# A network pharmacology-based study on Alzheimer disease prevention and treatment of Qiong Yu Gao

**DOI:** 10.1186/s13040-020-00212-z

**Published:** 2020-04-25

**Authors:** Jie-shu You, Chen-yue Li, Wei Chen, Xia-lin Wu, Li-jie Huang, Ren-kai Li, Fei Gao, Ming-yue Zhang, Huan-lan Liu, Wei-ling Qu

**Affiliations:** 1 School of Basic Medical Sciences, Guangzhou University of Chinese Medicine, Guangzhou, Guangdong Province China; 2 The First Affiliated Hospital of Guangzhou University of Chinese Medicine, Guangzhou, Guangdong Province China; 3 Department of Pharmacology and Pharmacy, The University of Hong Kong, Pok Fu Lam, Hong Kong; 4 College of Pharmacy, Chengdu University of Traditional Chinese Medicine, Chengdu, Sichuan Province China; 5 Division of Hematology, Renji Hospital, School of Medicine, Shanghai Jiaotong University, Shanghai, China

**Keywords:** Alzheimer disease, Gene regulatory networks, Genetic testing, Medicine, Chinese traditional, Molecular mechanisms of pharmacological action

## Abstract

**Background and objective:**

As the pathological mechanisms of AD are complex, increasing evidence have demonstrated Chinese Medicine with multi-ingredients and multi-targets may be more suitable for the treatment of diseases with complex pathogenesis. Therefore, the study was to preliminarily decipher the bioactive compounds and potential mechanisms of Qiong Yu Gao (QYG) for AD prevention and treatment by an integrated network pharmacology approach.

**Methods:**

Putative ingredients of QYG and significant genes of AD were retrieved from public database after screening. Then QYG ingredients target proteins/genes were obtained by target fishing. Compound-target-disease network was constructed using Cytoscape to decipher the mechanism of QYG for AD. KEGG pathway and GO enrichment analysis were performed to investigate the molecular mechanisms and pathways related to QYG for AD treatments.

**Results:**

Finally, 70 compounds and 511 relative drug targets were collected. In which, 17 representative direct targets were found. Gene ontology enrichment analysis revealed that the adenylate cyclase-inhibiting G-protein coupled acetylcholine receptor signaling pathway was the key biological processes and were regulated simultaneously by the 17 direct targets. The KEGG pathway enrichment analysis found that three signaling pathways were closely related to AD prevention and treatment by QYG, including PI3K-Akt signaling pathway, regulation of actin cytoskeleton pathway and insulin resistance pathway.

**Conclusion:**

This study demonstrated that QYG exerted the effect of preventing and treating AD by regulating multi-targets with multi-components. Furthermore, the study demonstrated that a network pharmacology-based approach was useful for elucidation of the interrelationship between complex diseases and interventions of Chinese herbal medicines.

## Introduction

Alzheimer disease (AD), is a chronic neurodegenerative disease associated with the gradual degeneration of various cortical areas and it is also the cause of 60–70% of cases of dementia [1, 2]. In 2015, approximately 29.8 million people worldwide suffered from AD and dementia led to about 1.9 million deaths [1, 3]. The cause of Alzheimer’s disease is complex and poorly understood, mainly including late age and the inheritance. Except that, a history of head injuries, depression and hypertension, smoking, obesity, diabetes and low level of education also increase the risks of AD. Although substantial progress has been made over the past few decades in exploring the pathophysiology and management of AD, unfortunately, no recognized and feasible treatments have been found to prevent and cure AD. A few drugs for AD therapy affirmed by the US Food and Drug Administration (FDA), such as acetylcholinesterase (AChE) inhibitors and *N*-methyl _D_-aspartate receptor (NMDAR) antagonists, only ameliorate some symptoms of AD, but they have no effect on the prevention and progression [4]. Therefore, it is urgent to systematically elucidate the mechanism of AD and search for safe and effective agents against AD.

Qiong Yu Gao (QYG), a classic anti-aging prescription, is consisted of Rehmanniae Radix (Sheng Dihuang), Poria Cocos (Fuling), Ginseng Radix et Rhizoma (Renshen) and MEL (Baimi). Rehmanniae Radix is the root tuber of *Rehmannia glutinosa Libosch* and is also one of the common and classic Chinese Medicine, which has been used in clinical practice for more than 3000 years in China. Traditionally, Rehmannia Radix is used for clearing heat and cooling blood, and nourishing Yin and promoting the production of body fluid. Modern researches further demonstrated the effects of Rehmannia Radix in treating fever, anemia, osteoporosis, hypertension, antioxidation and the disorder of autonomic nervous system [5–7]. Poria Cocos is the dried sclerotium of *Poria Cocos* (Schw.) Wolf and its traditional efficacy is eliminating dampness and diuresis, invigorating spleen and calming heart. Recent studies have shown that Poria Cocos exhibits anti-inflammatory, anti-cancer, antioxidant and antiviral activities [8–10]. Ginseng Radix et Rhizoma, the root and rhizome of *Panax ginseng* C. A. Mey, is very popular and well accepted all around the world. In china, Ginseng Radix et Rhizoma not only can be used alone for saving lives by reinforcing vital energy, but only plays an irreplaceable role in plenty of formulas such as Sijunzi decoction, Buzhongyiqi decoction, etc. [11] MEL is the honey of *Apis cerana* Fabricius and is commonly used for improving immune function, delaying aging and benefiting beauty [12, 13]. Actually, all of the four ingredients in QYG belong to medicinal and edible food/medicine. QYG has been clinically used for the treatment of cough with deficiency of yin and anti-aging in China for over 700 years. Besides, according to the record in Compendium of Materia Medica, QYG could increase intelligence, make hair black and delay aging. And the same efficacy of QYG is also recorded in the ancient and classis book “Hong’s Collection of Effective Recipes”. Modern pharmacological study further demonstrated its anti-aging effect and complex mechanism, that QYG plays an important role in protecting the central nervous system of aging animals by effectively removing free radicals from the brain and activating mitochondrial autophagy in order to promote the repair of damaged neurons [14–16]. As aging is the leading risk factor for sporadic AD and the mechanism of anti-aging of QYG is closed related to pathophysiology of AD [17], therefore, both the ancient classical and modern evidences demonstrate that QYG is an effective agent for AD prevention and treatment, and a holistic understanding of its molecular mechanisms needs to be further explored.

Network pharmacology, as one part of Bioinformatics, was first proposed by Andrew L Hopkins [18]. Network pharmacology has been used in the modern research of Chinese Medicine in recent years, as it not only integrates biological information and systematic medicine, but also is in accordance with the connotation of holistic theory, multi-components and multi-targets of Chinese Medicine. Network pharmacology can potentially reveal mechanisms of complex herbal formulae by discovering bioactive ingredients and biomarkers.

**Fig. 1:**
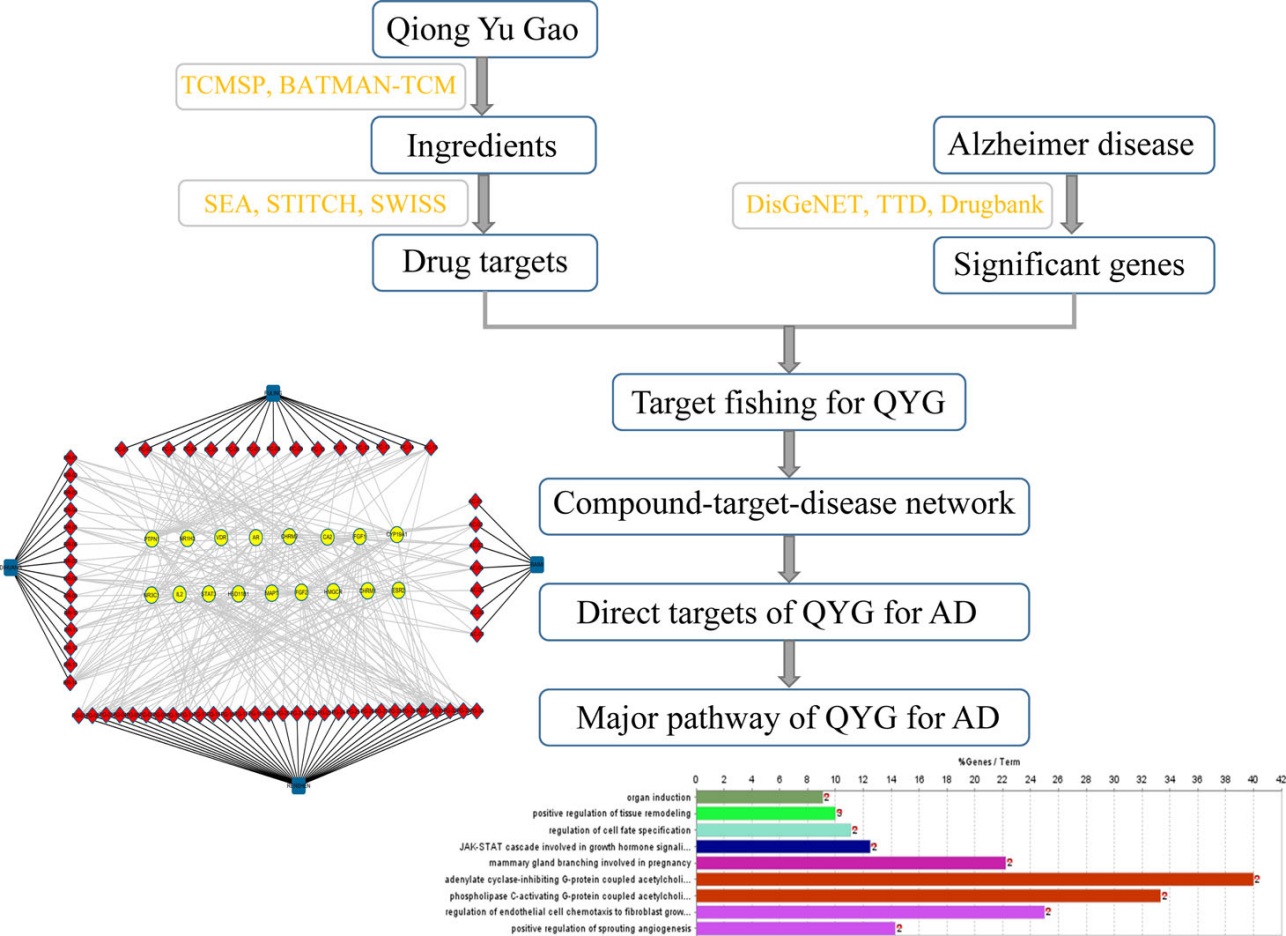
The flowchart of the whole study design

In this study, network pharmacology was employed to establish the compound-target-disease network to explore the potential mechanism of QYG in AD prevention and treatment. The flowchart of the whole study design was illustrated in Fig. 1, and the following was a brief description: firstly, we collected the chemical ingredients in QYG and significant genes for AD; then target fishing was used to predict the potential targets and the compound-target-disease network was constructed; Finally, we conducted gene ontology enrichment analysis. This study may also contribute to the new function discovery and product development of QYG.

## Materials and methods

### Collection of chemical ingredients in QYG

All of the chemical ingredients of QYG were collected from Traditional Chinese Medicine Systems Pharmacology (TCMSP) Database [19] (http://lsp.nwu.edu.cn/tcmsp.php, designed by Center for Bioinformatics, Northwest University, Xian, Shaanxi, China), a Bioinformatics Analysis Tool for Molecular Mechanism of Traditional Chinese Medicine [20] (BATMAN-TCM, http://bionet.ncpsb.org/batman-tcm/, designed by State Key Laboratory of Proteomics, Beijing Proteome Research Center, Beijing Institute of Radiation Medicine, Beijing, China) and Traditional Chinese Medicine Integrated Database [21] (TCMID, http://www.megabionet.org/tcmid/, designed by Center for Bioinformatics and Computational Biology, The Institute of Biomedical Sciences, College of Life Science, East China Normal University, Shanghai, China). According to a suggested criterion in TCMSP database, the ingredients whose drug-likeness (DL) values ≥0.18, oral bioavailability (OB) ≥ 30% and blood-brain barrier (BBB) ≥ − 0.3 were regarded as putative major ingredients and retained. In addition, those compounds with high contents and significant pharmacological activities but not meet the requirements were also retained.

### Significant genes collection for AD

AD significant genes were retrieved from 3 databases, DisGeNET [22] Therapeutic Target Database (TTD, http://www.disgenet.org/home/, designed by Integrative Biomedical Informatics Group, Research Unit on Biomedical Informatics, Barcelona Biomedical Research Park, Barcelona, Spain) and Drugbank Database [23] (https://www.drugbank.ca/, created by the scientists at the University of Alberta, Edmonton, Canada). The repetitive genes collected from the two databases were removed.

### Target fishing for QYG

The interaction between compounds and targets is the first principle for drug discovery. This target fishing was used to search for or predict the potential targets of small molecules. The targets of ingredients were predicted by Similarity Ensemble Approach [24] (SEA, http://sea.bkslab.org/, provided by the Shoichet Laboratory, Department of Pharmaceutical Chemistry, University of California, San Francisc, USA), STITCH [25] (http://stitch.embl.de/, supported by European Molecular Biology Laboratory, Swiss Institute of Bioinformatics, NNF Center for Protein Research) and Swiss Target Prediction [26] (Swiss, http://www.swisstargetprediction.ch/, provided by Molecular Modelling Group, the University of Lausanne, Lausanne, Switzerland). *Homo sapiens* were the only species for the targets. The common targets between AD and small molecules were retained.

### Network construction and analysis

The compound-target-disease network was constructed using Cytoscape software (Version 3.2.1, designed by Department of Bioengineering, University of California-San Diego, California, USA) [27]. The network was analyzed using Cytoscape plugin CentiScaPe to calculate topological parameters, mainly including the degree, betweenness centrality, closeness centrality and average shortest path length. The major ingredients and targets were represented by significant node, and the interactions were encoded by edges.

### Gene ontology enrichment analysis

The KEGG pathway enrichment was carried out by the Database for Annotation, Visualization and Integrated Discovery (DAVID) [28]. The direct targets of QYG for AD treatment were input in the DAVID gene list panel, and the most significant pathways were obtained by analyzing the corresponding parameter value (*p* value). Besides, Cytoscape plugin ClueGO [29] was applied to analyze the gene ontology (GO) enrichment. The terms with Expression Analysis Systematic Explorer scores of ≤0.05 were selected for functional annotation clustering and a *p* value of ≤0.05 for multiple hypothesis testing error was regarded as the significance cutoff [30]. Smaller *p* values indicated greater enrichment.

## Results

### Candidate genes associated with AD

A total of 1932 significant genes were collected from the TTD and Drugbank databases after deleting repetitions (Supplementary file, Table S1).

### Putative major chemical ingredients in the 4 herbs of QYG

Twenty-eight ingredients with OB ≥ 30%, DL ≥ 0.18 and BBB ≥ -0.3 from TCMSP, BATMAN-TCM and TCMID were selected. Besides, some compounds out of the condition but with pharmacological activities and high contents, including catalpol, rehmaglutin A, rehmaglutin B, rehmaglutin D, adenosine, acteoside, daucosterol, echinacoside, martynoside, rehmaionoside B and rehmaionoside C from Rehmanniae Radix (RR), polyporenic acid C, pachymic acid, poricoic acid A, poricoic acid B, poricoic acid C, poricoic acid D, poricoic acid DM, tumulosic acid and ergosterol from Poria Cocos (PC), ginsenoside Rh2, panaxynol, 20(S)-protopanaxadiol, ginsenoside La, ginsenoside Ro, ginsenoside Rc, ginsenoside Re, ginsenoside rf, gypenoside LXIX, sanchinoside C1, Panaxytriol, 20(R)-ginsenoside Rg2, 20-(S)-Ginsenoside Rg3, ginsenoside Rb1, and ginsenoside Rg1 from Ginseng Radix et Rhizoma *of Panax ginseng* C. A. Mey (PG), and fructose, glucose, maltose, pyridoxine, acetylcholine and sucrose from MEL of *Apis cerana* (AC), were also regarded as putative active ingredients. The final candidate compounds were shown in Supplementary file, Table S2.

### Network construction of the anti-AD targets of QYG

**Fig. 2:**
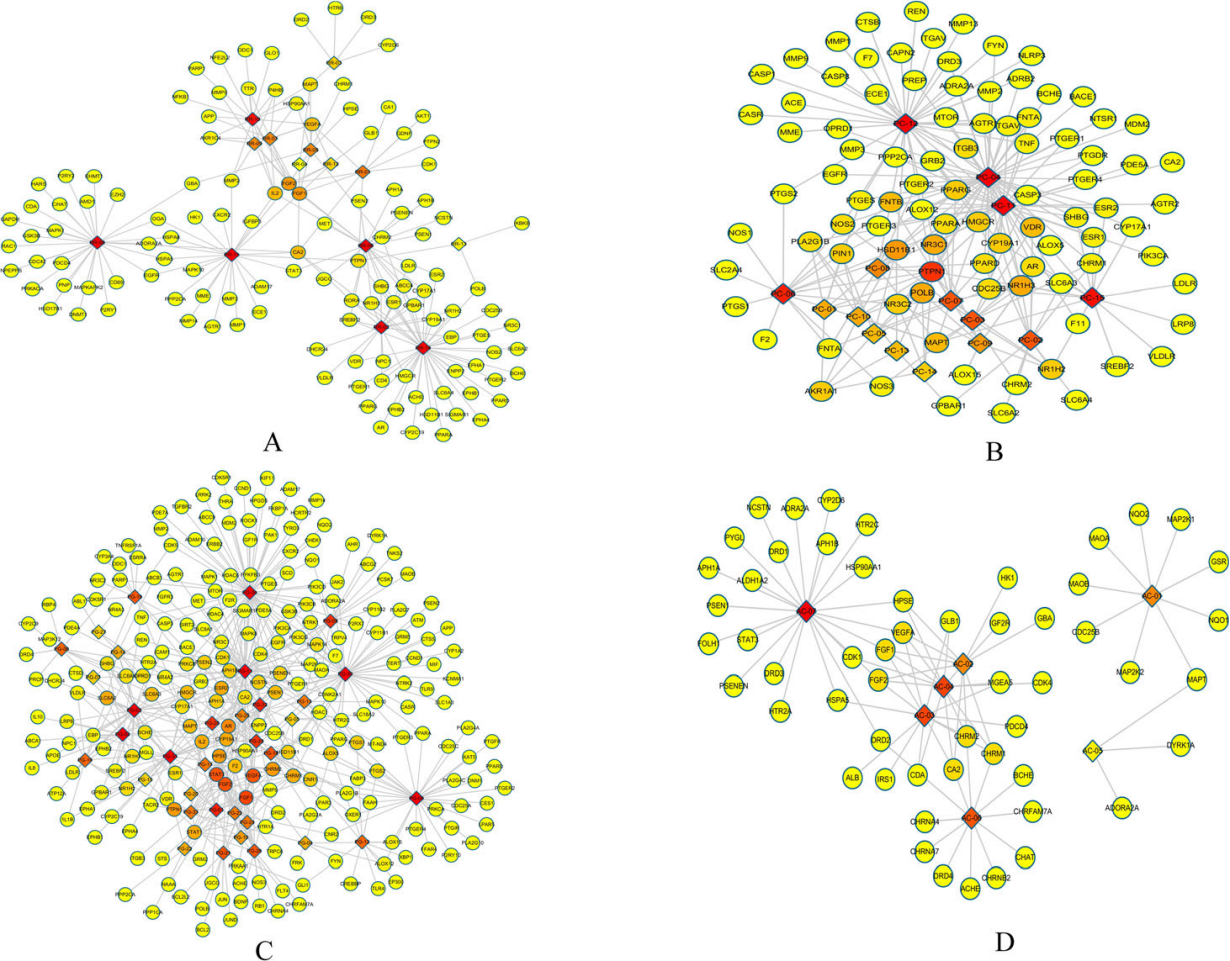
The compound-target-disease networks of **a** RR, **b** PC, **c** PG and **d** AC. The diamond nodes represent ingredients, and the circular nodes represent targets. The colors of the nodes are illustrated from red to yellow in descending order of degree values

According to pharmacophore matching, some statistical factors and similarity measures, 1270 targets of QYG for *Homo sapiens* were obtained. In which, RR contained 333, PC contained 184, PG contained 594, and AC contained 159 (Supplementary file, Table S3). And compound-target-disease network for the 4 herbs were constructed, respectively (RR-Targets, Fig. 2a; PC-Targets, Fig. 2b; PG-Targets, Fig. 2c and AC-Targets, Fig. 2d). The results revealed that 14 major ingredients in RR, 15 in PC, 34 in PG and 7 in AC may play important roles in anti-AD.

As shown in Fig. 2a, 123 AD-related targets were associated with 14 ingredients in RR and the analysis of network revealed that β-sitosterol (41 targets) was predicted as the major ingredient in RR. FGF1 and FGF2 were forecasted as the major targets of RR for the treatment of AD. As shown in Fig. 2b, the PC network included 93 targets. Among the ingredients of PC, polyporenic acid C (48 targets) was regarded as a major ingredient. PTPN1, HSD11B1, NR3C1, POLB and NR1H3 were predicted as major targets of PC for the treatment of AD. The PG network was shown in Fig. 2c, containing 240 targets. Gomisin A (61 targets) was predicted as the major ingredient of PG for the treatment of AD, and FGF1, FGF2, VEGFA and STAT3 were predicted as the major targets. For AC network, 7 bioactive ingredients in AC were validated to bind with 55 AD-related targets, in which sucrose (23 targets) was forecasted as the major ingredient and CA2, FGF1, CHRM2, FGF2, CHRM1 and VEGFA were the major target (Fig. 2d).

**Fig. 3:**
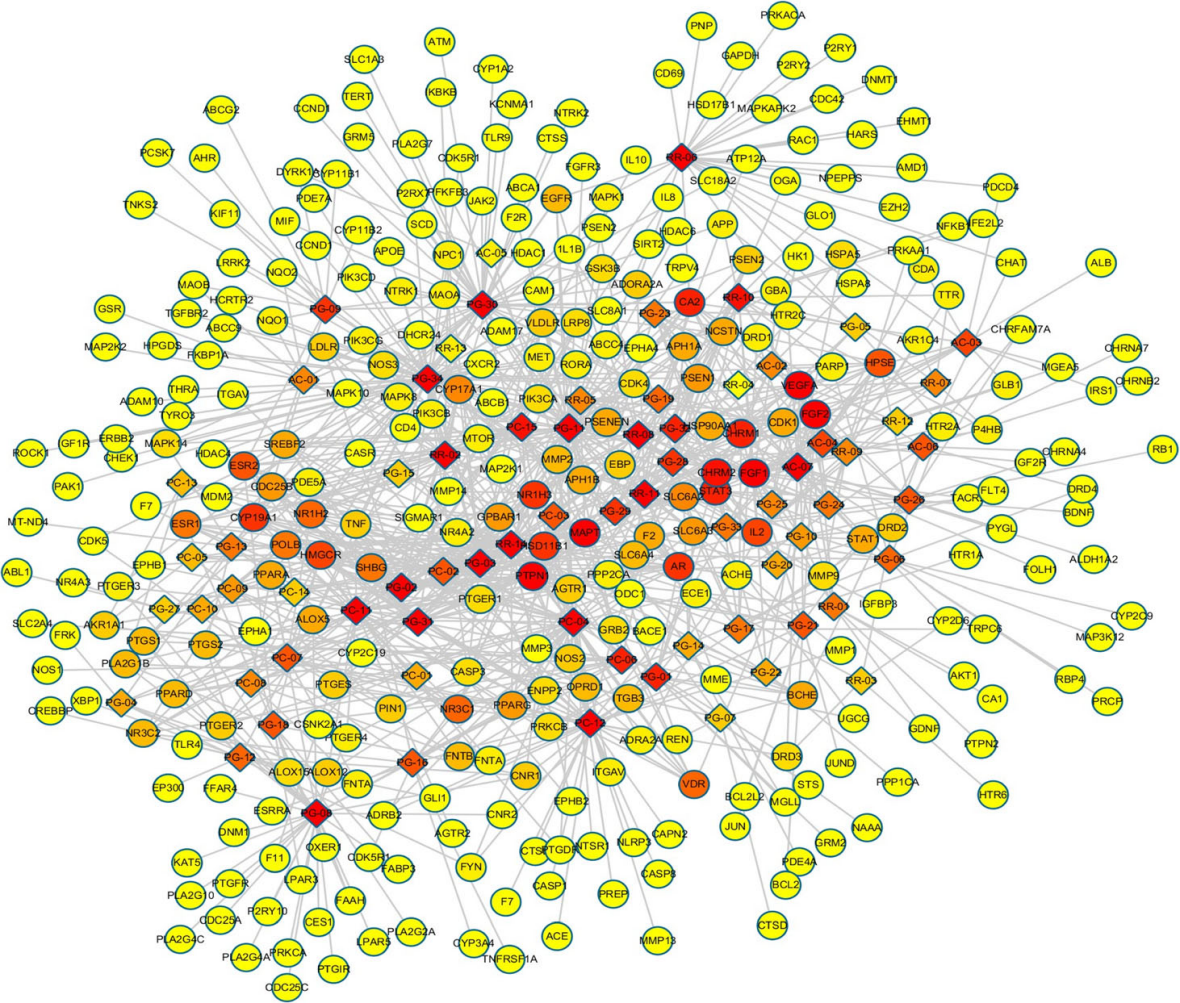
The compound-target-disease networks of QYG for AD treatment. The diamond nodes represent ingredients, and the circular nodes represent targets. The colors of the nodes are illustrated from red to yellow in descending order of degree values

As shown in Fig. 3, the compound-target-disease interaction network was constructed. The ingredients of QYG were described by diamond nodes, and the direct targets of QYG for AD treatment were encoded by circular nodes. Only the targets with higher values of “degree” (above two-fold of the median value) “betweenness centrality” and “closeness centrality” (above the median value), and “average shortest path length” (below the median value) were identified as the candidate targets of QYG for AD. Ultimately, 17 direct targets were screened (Table 1).

**Table 1:** The major targets of QYG for AD treatment and its relevant topological parameters

Uniprot ID	Protein name	Gene name	Degree	Betweenness centrality	Closeness centrality	Average shortest path length
P18031	Tyrosine-protein phosphatase non-receptor type 1	PTPN1	26	0.066106	0.337931	2.959184
P05230	Fibroblast growth factor 1	FGF1	25	0.041648	0.342657	2.918367
P09038	Fibroblast growth factor 2	FGF2	24	0.033146	0.334471	2.989796
P10636	Microtubule-associated protein tau	MAPT	18	0.038501	0.309637	3.229592
P08172	Muscarinic acetylcholine receptor M2	CHRM2	16	0.02588	0.319739	3.127551
P00918	Carbonic anhydrase 2	CA2	15	0.069146	0.345679	2.892857
P11229	Muscarinic acetylcholine receptor M1	CHRM1	15	0.026518	0.324503	3.081633
P40763	Signal transducer and activator of transcription 3	STAT3	15	0.011336	0.315113	3.173469
P10275	Androgen receptor	AR	14	0.037305	0.360958	2.770408
P11511	Cytochrome P450 19A1	CYP19A1	14	0.024461	0.332767	3.005102
P28845	Corticosteroid 11-beta-dehydrogenase isozyme 1	HSD11B1	14	0.029686	0.345679	2.892857
Q13133	Oxysterols receptor LXR-alpha	NR1H3	14	0.008421	0.311111	3.214286
P04035	3-hydroxy-3-methylglutaryl-coenzyme A reductase	HMGCR	13	0.030442	0.34087	2.933673
P60568	Interleukin-2	IL2	13	0.011053	0.316129	3.163265
Q92731	Estrogen receptor beta	ESR2	12	0.016322	0.329412	3.035714
P04150	Glucocorticoid receptor	NR3C1	11	0.019069	0.331641	3.015306
P11473	Vitamin D receptor	VDR	11	0.007378	0.311111	3.214286

### The characteristics of QYG in the treatment of AD

**Fig. 4:**
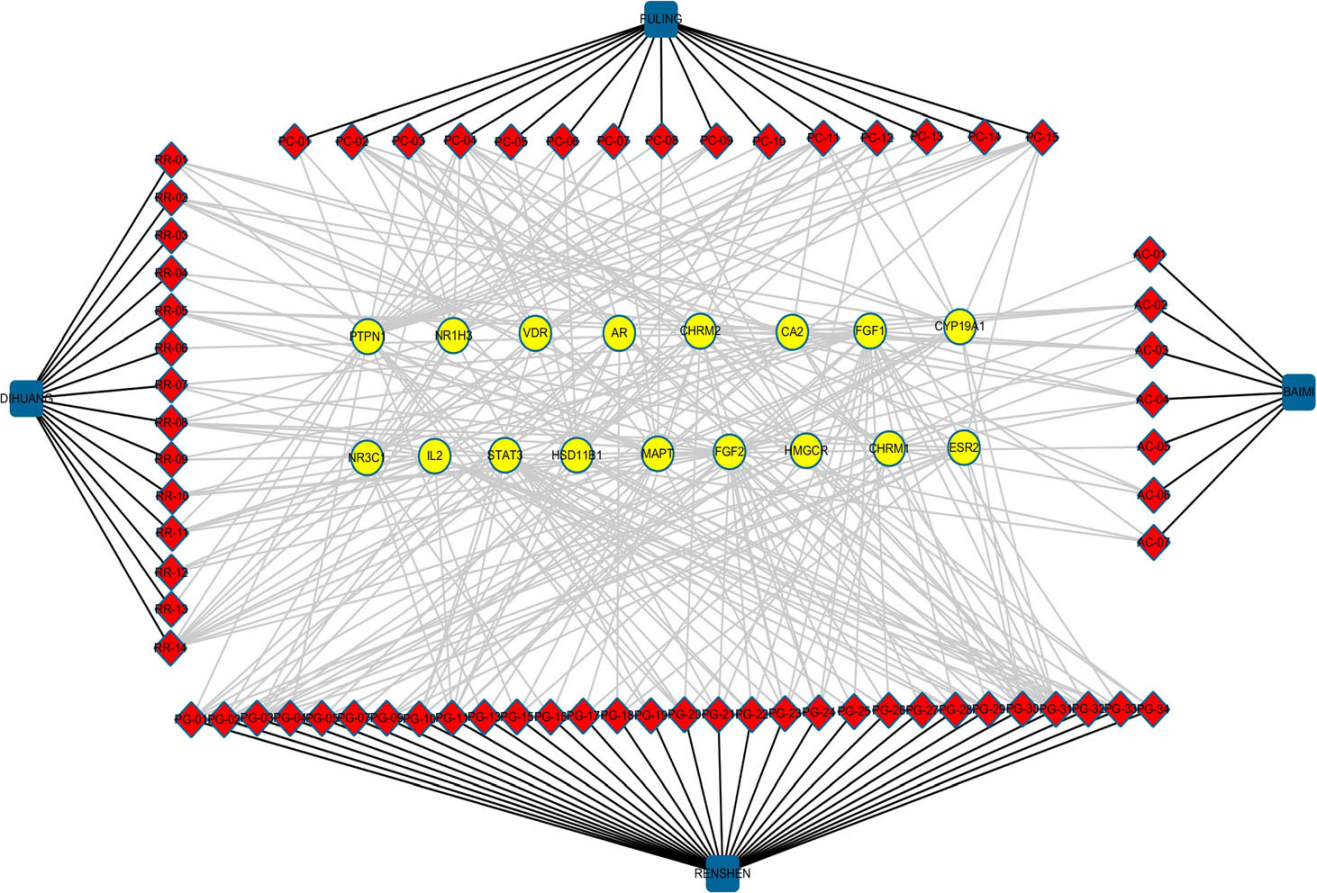
The compound-direct target-disease interaction network of QYG in treating AD. The diamond nodes represent ingredients; the circular nodes represent direct targets; and the blue rectangles represent herbs in QYG

As shown in Fig. 4, the ingredients directly to the targets were obtained via the compound-target-disease interaction network. Multi-ingredients from RR, PC, PG and AC regulated almost all direct targets. The results indicated that the anti-AD effect of QYG was played through regulating multi-targets by multi-ingredients.

### GO enrichment analysis for targets

**Fig. 5:**
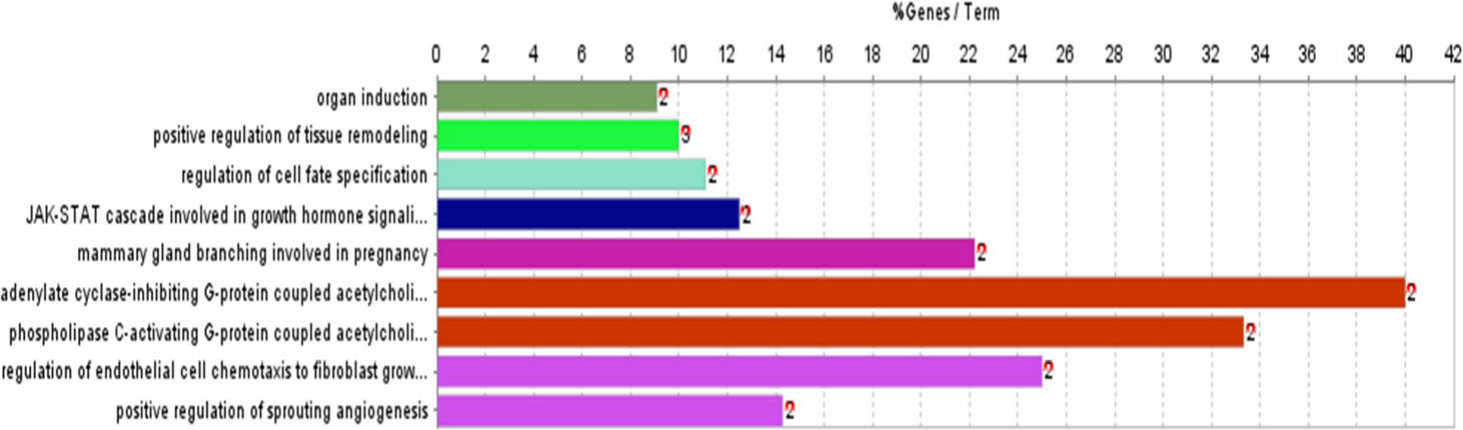
ClueGO analysis of the candidate direct targets of QYG for AD. Functionally grouped network of enriched categories was generated for the target genes. The x-axis represents the enrichment scores of these terms (*p* ≤ 0.05); the y-axis represents significantly enriched BP categories

To investigate the biological functions of the direct targets of QYG for AD, the gene GO biological process (BP) was performed by the Cytoscape plugin ClueGO. As shown in Fig. 5, 9 most significantly enriched GOBP terms (*p* ≤ 0.05) were listed. Among the 9 GOBP terms, adenylate cyclase-inhibiting G-protein coupled acetylcholine receptor signaling pathway was the most prominent.

### Target-pathway network and analysis

The functional annotation tool of DAVID was used for KEGG pathway enrichment analysis. Ultimately, three signaling pathways were obtained, including PI3K-Akt signaling pathway, regulation of actin cytoskeleton pathway and insulin resistance pathway (Table 2).

**Table 2:** The KEGG pathway annotation of the direct targets of QYG for AD treatment

Signaling pathway	Count	***p*** value	Gene
PI3K-Akt signaling pathway	5	0.0070	FGF2, IL2, CHRM2, CHRM1, FGF1
Regulation of actin cytoskeleton pathway	4	0.0117	FGF2, CHRM2, CHRM1, FGF1
Insulin resistance pathway	3	0.0254	NR1H3, PTPN1, STAT3

## Discussion

The etiology and pathogenesis of AD is complex. Chinese medicine, focus on systemic functional adjustments, is more suitable for the disease with complex pathogenesis. QYG, as a TCM formula, has the characteristics of multi-components and multi-targets therapy for AD. However, it is difficult to reveal its possible active ingredients and pharmacological mechanism. Network pharmacology may be an excellent method, which contributes to collecting active ingredients and pharmacological actions of herbs or TCM formula. In this study, the network pharmacology approach was used to identify the putative bioactive compounds in QYG and potential targets, and to explore the potential mechanisms of QYG for AD prevention and treatment. The results showed that QYG exerted the effect of treating AD by regulating multi-targets with multi-components.

Specifically, 70 ingredients of these 4 herbs were screened out. In which, several ingredients have been reported to improve the cognitive impairment and neurodegenerative disorders. For example, catalpol in RR is used for improving learning ability and memory for AD patients [31]. Trametenolic acid could significantly improve learning and memory ability of the cerebral ischemia injury rats [32]. Wu et al. found that Hederagenin could be used as a novel autophagic enhancer to play a role in neuroprotection by improving motor deficits in Parkinson’s disease mice model [33]. Stigmasterol could ameliorate memory impairment and promotes neuritogenesis and synaptogenesis [34]. Ginsenosides from ginseng showed the great therapeutic potential in AD [35]. The above evidence suggests the activities of these ingredients in QYG for the treatment of AD, but further experimental verification is still needed.

By target fishing for QYG and AD, the result showed that ingredients affected 123 AD-related targets in RR, 93 in PC, 240 in PG and 55 in AC. Furthermore, 17 representative targets including PTPN1, FGF1, FGF2, MAPT, CHRM2, CA2, CHRM1, STAT3, AR, CYP19A1, HSD11B1, NR1H3, HMGCR, IL2, ESR2, NR3C1 and VDR were regulated QYG. In which MAPT, CHRM1, CHRM2 and CA were the overlapped targets of the four herbs in QYG, reflecting that some active ingredients may interact with each other to play a synergistic effect. The relevant literatures also revealed that FGF1, FGF2, MAPT, CHRM2, CA2, CHRM1, STAT3, AR, CYP19A1, HSD11B1, NR1H3, HMGCR, ESR2, ESR1 and VDR may be involved in the pathogenesis of AD. The results of GO enrichment analysis revealed that the direct targets of QYG for AD were closely related to the adenylate cyclase-inhibiting G-protein coupled acetylcholine receptor signaling pathway.

Putative bioactive compounds in QYG associated with direct AD genes were placed in the DAVID for KEGG pathway analysis. According to the corresponding parameter (*p* value), some important pathways that may be regulated by QYG in the process of treating diseases were screened out, including PI3K-Akt signaling pathway, regulation of actin cytoskeleton pathway and insulin resistance pathway. The actin cytoskeleton is a dynamic network made up of actin polymers and associated actin binding proteins. Recent studies found that interference with the expression or function of upstream regulators of the actin cytoskeleton, including Rac-GEFs, Rac, and Rac targets, PAK could cause spine and synapse loss [36–38]. Interestingly, synapse degeneration is also demonstrated as a major component of AD pathology [39]. PI3K/Akt pathway is a signalling cascade centering on serine/threonine kinase Akt. Misregulation of the PI3K/AKT signaling pathway has been found to be closely associated with AD. The PI3K-Akt signaling pathway in AD is involved in regulating many basic cellular functions, such as cell cycle growth, cell migration, and apoptosis [40]. The insulin signal pathway mediating normal biological effects mainly include insulin receptor, insulin receptor substrates, phosphatidylinositol-3 kinase, phosphorylated PI3K, serine-threonine kinase, and phosphorylated AKT [41]. And emerging data have demonstrated that AD-associated abnormalities in energy metabolism is caused by brain insulin resistance and insulin deficiency. In turn, AD can further result in brain insulin signaling pathway injuries. In these pathways, genes CHRM2, CHRM1 are involved in three pathways. CHRM1 belongs to excitatory M1 class of muscarinic acetylcholine receptor and CHRM2 belongs to inhibitory M2 class. Increasing evidence suggest that CHRM1 may be a candidate gene for the pathogenesis of AD, with decreased CHRM1 or CHRM1 mRNA level, or unchanged central CHRM1 levels, but with the reduced radioligand-binding affinity [42, 43]. CHRM2 gene is demonstrated to be involved in synaptic plasticity, neuronal excitability, release of acetylcholine and cognitive function [44, 45]. And studies found that neuritic plaques, neurofibrillary tangles, soluble amyloid, neuronal death, loss of neurotransmitters, synapse loss, inflammation, and oxidative stress may be the targets for primary prevention in AD [46].

This study systematically explored the putative bioactive compounds in QYG and pharmacological mechanisms of QYG for AD prevention and treatment through network pharmacology. QYG was selected based on our previous study, which was much more reliable than those based on literature retrieval. All the ingredients of QYG belongs to medicinal and edible plant or diet, and they have a history of safe use. The potential advantage of “network targets, multi-component” strategy of network pharmacology is obvious, but it is generally as a guided theoretical science, which is also the limitation of this study. Therefore, the demonstration in the gene and protein levels of QYG treated Alzheimer disease will be provided in the future.

## Conclusion

This study demonstrated that the network pharmacological analysis was useful for elucidation of the interrelationship between complex diseases such as AD and interventions of Chinese medicines. Based on the study, we analyzed the active materials and the possible molecular mechanism of QYG on AD, which will also provide a theoretical basis for the future in-depth study of QYG for AD treatment. Besides, as QYG belongs to medicinal and edible formula, it will have a broader clinical practice and market.

## Supplementary information

**Additional file 1 **: **Table S1**. The 1981 significant genes associated with AD. **Table S2**. The putative major chemical ingredients and ADME parameters in QYG. **Table S3**. The detailed target information of the ingredients of herbs

## Data Availability

All data are available in the manuscript and they are showed in figures, tables and supplement file.

## Abbreviations

AD: Alzheimer disease
QYG: Qiong Yu Gao;
FDA: US Food and Drug Administration
AChE: Acetylcholinesterase
NMDAR: *N*-methyl _D_-aspartate receptor
TCMSP: Traditional Chinese Medicine Systems Pharmacology
BATMAN-TCM: Bioinformatics Analysis Tool for Molecular mechanism of Traditional Chinese Medicine
DL: Drug-likeness
OB: Oral bioavailability
DAVID: Annotation, Visualization and Integrated Discovery
GO: Gene ontology
BP: Biological process
RR: *Radix Rehmanniae*
PC: *Poria Cocos* (Schw.) Wolf
PG: *Panax ginseng* C. A. Mey
AC: *Apis cerana*
