# Supplementary material for: A network pharmacology-based study on Alzheimer disease prevention and treatment of Qiong Yu Gao
Source: BioData Min. 2020 Apr 25;13:2. doi: 10.1186/s13040-020-00212-z (PMC7183652; doi:10.1186/s13040-020-00212-z)
Supplement: Supplementary file 1 — Additional file 1 : Table S1. The 1981 significant genes associated with AD. Table S2. The putative major chemical ingredients and ADME parameters in QYG. Table S3. The detailed target information of the ingredients of herbs [file 13040_2020_212_MOESM1_ESM.docx]

**Table S1: The 1981 significant genes associated with AD**

| Uniprot | Symbol | Gene_name |
| --- | --- | --- |
| P05067 | APP | amyloid beta precursor protein |
| P02649 | APOE | apolipoprotein E |
| P49768 | PSEN1 | presenilin 1 |
| Q92673 | SORL1 | sortilin related receptor 1 |
| P10415 | BCL2 | BCL2, apoptosis regulator |
| P23560 | BDNF | brain derived neurotrophic factor |
| P12821 | ACE | angiotensin I converting enzyme |
| P49841 | GSK3B | glycogen synthase kinase 3 beta |
| P01584 | IL1B | interleukin 1 beta |
| P06213 | INSR | insulin receptor |
| P41159 | LEP | leptin |
| P00749 | PLAU | plasminogen activator, urokinase |
| P01344 | IGF2 | insulin like growth factor 2 |
| P01303 | NPY | neuropeptide Y |
| P08069 | IGF1R | insulin like growth factor 1 receptor |
| P01308 | INS | insulin |
| Q07812 | BAX | BCL2 associated X, apoptosis regulator |
| P10909 | CLU | clusterin |
| P49810 | PSEN2 | presenilin 2 |
| Q13492 | PICALM | phosphatidylinositol binding clathrin assembly protein |
| Q8IZY2 | ABCA7 | ATP binding cassette subfamily A member 7 |
| Q9Y5K6 | CD2AP | CD2 associated protein |
| Q9NZC2 | TREM2 | triggering receptor expressed on myeloid cells 2 |
| P17927 | CR1 | complement C3b/C4b receptor 1 (Knops blood group) |
| P62760 | VSNL1 | visinin like 1 |
| Q9BZA7 | PCDH11X | protocadherin 11 X-linked |
| Q96JQ5 | MS4A4A | membrane spanning 4-domains A4A |
| P01023 | A2M | alpha-2-macroglobulin |
| P22303 | ACHE | acetylcholinesterase (Cartwright blood group) |
| O00499 | BIN1 | bridging integrator 1 |
| P06276 | BCHE | butyrylcholinesterase |
| P0DP23;  P0DP24;  P0DP25 | CALM1 | calmodulin 1 |
| P42574 | CASP3 | caspase 3 |
| P20138 | CD33 | CD33 molecule |
| P36544;  Q494W8 | CHRNA7 | cholinergic receptor nicotinic alpha 7 subunit |
| P01034 | CST3 | cystatin C |
| P10635 | CYP2D6 | cytochrome P450 family 2 subfamily D member 6 |
| Q15392 | DHCR24 | 24-dehydrocholesterol reductase |
| Q16555 | DPYSL2 | dihydropyrimidinase like 2 |
| P21709 | EPHA1 | EPH receptor A1 |
| P03372 | ESR1 | estrogen receptor 1 |
| Q30201 | HFE | homeostatic iron regulator |
| P09601 | HMOX1 | heme oxygenase 1 |
| P14735 | IDE | insulin degrading enzyme |
| P05019 | IGF1 | insulin like growth factor 1 |
| P05231 | IL6 | interleukin 6 |
| P10636 | MAPT | microtubule associated protein tau |
| P05164 | MPO | myeloperoxidase |
| P42898 | MTHFR | methylenetetrahydrofolate reductase |
| P29474 | NOS3 | nitric oxide synthase 3 |
| P37231 | PPARG | peroxisome proliferator activated receptor gamma |
| F7VJQ1;  P04156 | PRNP | prion protein |
| P78509 | RELN | reelin |
| P01375 | TNF | tumor necrosis factor |
| P15692 | VEGFA | vascular endothelial growth factor A |
| Q9Y6A2 | CYP46A1 | cytochrome P450 family 46 subfamily A member 1 |
| Q92542 | NCSTN | nicastrin |
| P56817 | BACE1 | beta-secretase 1 |
|  | MIR146A | microRNA 146a |
| Q13867 | BLMH | bleomycin hydrolase |
| P27338 | MAOB | monoamine oxidase B |
| P04179 | SOD2 | superoxide dismutase 2 |
| P02787 | TF | transferrin |
| Q00059 | TFAM | transcription factor A, mitochondrial |
| Q92870 | APBB2 | amyloid beta precursor protein binding family B member 2 |
| P17787 | CHRNB2 | cholinergic receptor nicotinic beta 2 subunit |
| Q6NXT4 | SLC30A6 | solute carrier family 30 member 6 |
| P05198 | EIF2S1 | eukaryotic translation initiation factor 2 subunit alpha |
| P25705 | ATP5F1A | ATP synthase F1 subunit alpha |
| P06850 | CRH | corticotropin releasing hormone |
| P06733 | ENO1 | enolase 1 |
| P00734 | F2 | coagulation factor II, thrombin |
| P11717 | IGF2R | insulin like growth factor 2 receptor |
| Q6ZW49 | PAXIP1 | PAX interacting protein 1 |
| Q7LC44 | ARC | activity regulated cytoskeleton associated protein |
| Q30154 | HLA-DRB5 | major histocompatibility complex, class II, DR beta 5 |
| P60174 | TPI1 | triosephosphate isomerase 1 |
| O14863 | SLC30A4 | solute carrier family 30 member 4 |
| Q9P2A4 | ABI3 | ABI family member 3 |
| Q15109 | AGER | advanced glycosylation end-product specific receptor |
| Q9UKV5 | AMFR | autocrine motility factor receptor |
| P28329 | CHAT | choline O-acetyltransferase |
| O14773 | TPP1 | tripeptidyl peptidase 1 |
| Q07954 | LRP1 | LDL receptor related protein 1 |
| P08473 | MME | membrane metalloendopeptidase |
| P01138 | NGF | nerve growth factor |
| P08138 | NGFR | nerve growth factor receptor |
| Q13526 | PIN1 | peptidylprolyl cis/trans isomerase, NIMA-interacting 1 |
| P16885 | PLCG2 | phospholipase C gamma 2 |
| P10082 | PYY | peptide YY |
| P35354 | PTGS2 | prostaglandin-endoperoxide synthase 2 |
| P04271 | S100B | S100 calcium binding protein B |
| P14672 | SLC2A4 | solute carrier family 2 member 4 |
| P00441 | SOD1 | superoxide dismutase 1 |
| O14556 | GAPDHS | glyceraldehyde-3-phosphate dehydrogenase, spermatogenic |
| Q16236 | NFE2L2 | nuclear factor, erythroid 2 like 2 |
| P35228 | NOS2 | nitric oxide synthase 2 |
| P11137 | MAP2 | microtubule associated protein 2 |
| Q16620 | NTRK2 | neurotrophic receptor tyrosine kinase 2 |
| P35568 | IRS1 | insulin receptor substrate 1 |
| Q06481 | APLP2 | amyloid beta precursor like protein 2 |
| P23219 | PTGS1 | prostaglandin-endoperoxide synthase 1 |
| Q9UBK2 | PPARGC1A | PPARG coactivator 1 alpha |
| Q9NPG2 | NGB | neuroglobin |
| Q03135 | CAV1 | caveolin 1 |
| Q16539 | MAPK14 | mitogen-activated protein kinase 14 |
| P35222 | CTNNB1 | catenin beta 1 |
| P51681 | CCR5 | C-C motif chemokine receptor 5 (gene/pseudogene) |
| P04792 | HSPB1 | heat shock protein family B (small) member 1 |
| P10809 | HSPD1 | heat shock protein family D (Hsp60) member 1 |
| O95140 | MFN2 | mitofusin 2 |
| Q99828 | CIB1 | calcium and integrin binding 1 |
| Q04656 | ATP7A | ATPase copper transporting alpha |
| P00390 | GSR | glutathione-disulfide reductase |
| Q00613 | HSF1 | heat shock transcription factor 1 |
| O14920 | IKBKB | inhibitor of nuclear factor kappa B kinase subunit beta |
| F8WCM5 | INS-IGF2 | INS-IGF2 readthrough |
| P02795 | MT2A | metallothionein 2A |
| P36955 | SERPINF1 | serpin family F member 1 |
| Q9UQC2 | GAB2 | GRB2 associated binding protein 2 |
| O96008 | TOMM40 | translocase of outer mitochondrial membrane 40 |
| Q9NQ66 | PLCB1 | phospholipase C beta 1 |
| Q13501 | SQSTM1 | sequestosome 1 |
| Q96AC1 | FERMT2 | fermitin family member 2 |
| Q2M3D2 | EXOC3L2 | exocyst complex component 3 like 2 |
| Q96PG1 | MS4A4E | membrane spanning 4-domains A4E |
| P08651 | NFIC | nuclear factor I C |
| Q92879 | CELF1 | CUGBP Elav-like family member 1 |
| Q9H0M4 | ZCWPW1 | zinc finger CW-type and PWWP domain containing 1 |
| Q9P2Q2 | FRMD4A | FERM domain containing 4A |
| Q9H2W1 | MS4A6A | membrane spanning 4-domains A6A |
| Q14289 | PTK2B | protein tyrosine kinase 2 beta |
| P08887 | IL6R | interleukin 6 receptor |
| Q92835 | INPP5D | inositol polyphosphate-5-phosphatase D |
| Q15650 | TRIP4 | thyroid hormone receptor interactor 4 |
| P20749 | BCL3 | B cell CLL/lymphoma 3 |
| P08235 | NR3C2 | nuclear receptor subfamily 3 group C member 2 |
| Q96I99 | SUCLG2 | succinate-CoA ligase GDP-forming beta subunit |
| Q9HCB6 | SPON1 | spondin 1 |
| O95319 | CELF2 | CUGBP Elav-like family member 2 |
| Q6UB35 | MTHFD1L | methylenetetrahydrofolate dehydrogenase (NADP+ dependent) 1 like |
| Q6V1P9 | DCHS2 | dachsous cadherin-related 2 |
| Q9NQ75 | CASS4 | Cas scaffold protein family member 4 |
| Q96A65 | EXOC4 | exocyst complex component 4 |
| Q96KG7 | MEGF10 | multiple EGF like domains 10 |
| Q9HCM2 | PLXNA4 | plexin A4 |
| Q8NFF2 | SLC24A4 | solute carrier family 24 member 4 |
| Q8NEA6 | GLIS3 | GLIS family zinc finger 3 |
| P01011 | SERPINA3 | serpin family A member 3 |
| O95477 | ABCA1 | ATP binding cassette subfamily A member 1 |
| O14672 | ADAM10 | ADAM metallopeptidase domain 10 |
| O94973 | AP2A2 | adaptor related protein complex 2 subunit alpha 2 |
| P09917 | ALOX5 | arachidonate 5-lipoxygenase |
| O00213 | APBB1 | amyloid beta precursor protein binding family B member 1 |
| P02647 | APOA1 | apolipoprotein A1 |
| Q03001 | DST | dystonin |
| Q06455 | RUNX1T1 | RUNX1 translocation partner 1 |
| Q00535 | CDK5 | cyclin dependent kinase 5 |
| P51861 | CDR1 | cerebellar degeneration related protein 1 |
| P11597 | CETP | cholesteryl ester transfer protein |
| P43681 | CHRNA4 | cholinergic receptor nicotinic alpha 4 subunit |
| O96005 | CLPTM1 | CLPTM1, transmembrane protein |
| O00590 | ACKR2 | atypical chemokine receptor 2 |
| P00450 | CP | ceruloplasmin |
| P02741 | CRP | C-reactive protein |
| P04141 | CSF2 | colony stimulating factor 2 |
| P26232 | CTNNA2 | catenin alpha 2 |
| P07858 | CTSB | cathepsin B |
| P07339 | CTSD | cathepsin D |
| Q9Y485 | DMXL1 | Dmx like 1 |
| P78352 | DLG4 | discs large MAGUK scaffold protein 4 |
| P53805 | RCAN1 | regulator of calcineurin 1 |
| Q13627 | DYRK1A | dual specificity tyrosine phosphorylation regulated kinase 1A |
| P09104 | ENO2 | enolase 2 |
| Q92731 | ESR2 | estrogen receptor 2 |
| P42345 | MTOR | mechanistic target of rapamycin kinase |
| Q99928 | GABRG3 | gamma-aminobutyric acid type A receptor gamma3 subunit |
| P04406 | GAPDH | glyceraldehyde-3-phosphate dehydrogenase |
| P15976 | GATA1 | GATA binding protein 1 |
| P14136 | GFAP | glial fibrillary acidic protein |
| P28799 | GRN | granulin precursor |
| Q13224 | GRIN2B | glutamate ionotropic receptor NMDA type subunit 2B |
| Q99714 | HSD17B10 | hydroxysteroid 17-beta dehydrogenase 10 |
| P28223 | HTR2A | 5-hydroxytryptamine receptor 2A |
| P01583 | IL1A | interleukin 1 alpha |
| P22301 | IL10 | interleukin 10 |
| Q9H2S1 | KCNN2 | potassium calcium-activated channel subfamily N member 2 |
| Q13753 | LAMC2 | laminin subunit gamma 2 |
| P08865 | RPSA | ribosomal protein SA |
| P01130 | LDLR | low density lipoprotein receptor |
| Q14767 | LTBP2 | latent transforming growth factor beta binding protein 2 |
| P50895 | BCAM | basal cell adhesion molecule (Lutheran blood group) |
| P51825 | AFF1 | AF4/FMR2 family member 1 |
| P14780 | MMP9 | matrix metallopeptidase 9 |
| Q13875 | MOBP | myelin-associated oligodendrocyte basic protein |
| P00403 | COX2 | cytochrome c oxidase subunit II |
| Q8IVG9 | RNR2 | l-rRNA |
| P20783 | NTF3 | neurotrophin 3 |
| P08183 | ABCB1 | ATP binding cassette subfamily B member 1 |
| P27169 | PON1 | paraoxonase 1 |
| Q15257 | PTPA | protein phosphatase 2 phosphatase activator |
| P28482 | MAPK1 | mitogen-activated protein kinase 1 |
| P18433 | PTPRA | protein tyrosine phosphatase, receptor type A |
| P23470 | PTPRG | protein tyrosine phosphatase, receptor type G |
| Q92692 | NECTIN2 | nectin cell adhesion molecule 2 |
| Q9UQ07 | MOK | MOK protein kinase |
| P13500 | CCL2 | C-C motif chemokine ligand 2 |
| P15907 | ST6GAL1 | ST6 beta-galactoside alpha-2,6-sialyltransferase 1 |
| P43004 | SLC1A2 | solute carrier family 1 member 2 |
| P31645 | SLC6A4 | solute carrier family 6 member 4 |
| P37840 | SNCA | synuclein alpha |
| P61278 | SST | somatostatin |
| P08247 | SYP | synaptophysin |
| P01137 | TGFB1 | transforming growth factor beta 1 |
| P04637 | TP53 | tumor protein p53 |
| P02766 | TTR | transthyretin |
| P0CG47 | UBB | ubiquitin B |
| P09936 | UCHL1 | ubiquitin C-terminal hydrolase L1 |
| O00116 | AGPS | alkylglycerone phosphate synthase |
| P78560 | CRADD | CASP2 and RIPK1 domain containing adaptor with death domain |
| Q9P2R7 | SUCLA2 | succinate-CoA ligase ADP-forming beta subunit |
| Q15078 | CDK5R1 | cyclin dependent kinase 5 regulatory subunit 1 |
| O43497 | CACNA1G | calcium voltage-gated channel subunit alpha1 G |
| O00421 | CCRL2 | C-C motif chemokine receptor like 2 |
| O60669 | SLC16A7 | solute carrier family 16 member 7 |
| O95237 | LRAT | lecithin retinol acyltransferase |
| Q9Y287 | ITM2B | integral membrane protein 2B |
| Q2Y0W8 | SLC4A8 | solute carrier family 4 member 8 |
| O60284 | ST18 | ST18, C2H2C-type zinc finger |
| Q14764 | MVP | major vault protein |
| O00429 | DNM1L | dynamin 1 like |
| Q9Y625 | GPC6 | glypican 6 |
| Q9Y4F1 | FARP1 | FERM, ARH/RhoGEF and pleckstrin domain protein 1 |
| Q9Y6X6 | MYO16 | myosin XVI |
| O60281 | ZNF292 | zinc finger protein 292 |
| Q9UJT9 | FBXL7 | F-box and leucine rich repeat protein 7 |
| Q9Y520 | PRRC2C | proline rich coiled-coil 2C |
| Q2KHT3 | CLEC16A | C-type lectin domain containing 16A |
| Q8IX03 | WWC1 | WW and C2 domain containing 1 |
| Q9UKU0 | ACSL6 | acyl-CoA synthetase long chain family member 6 |
| Q96EB6 | SIRT1 | sirtuin 1 |
| P62166 | NCS1 | neuronal calcium sensor 1 |
| Q13148 | TARDBP | TAR DNA binding protein |
| Q53HV7 | SMUG1 | single-strand-selective monofunctional uracil-DNA glycosylase 1 |
| Q9Y5Z0 | BACE2 | beta-secretase 2 |
| Q8TEL6 | TRPC4AP | transient receptor potential cation channel subfamily C member 4 associated protein |
| Q9NP56 | PDE7B | phosphodiesterase 7B |
| Q8NDV7 | TNRC6A | trinucleotide repeat containing 6A |
| Q9Y6E2 | BZW2 | basic leucine zipper and W2 domains 2 |
| Q9HBI1 | PARVB | parvin beta |
| Q9UHD0 | IL19 | interleukin 19 |
| Q9UMX0 | UBQLN1 | ubiquilin 1 |
| Q96DB5 | RMDN1 | regulator of microtubule dynamics 1 |
| Q8TEU7 | RAPGEF6 | Rap guanine nucleotide exchange factor 6 |
| Q9H6U6 | BCAS3 | BCAS3, microtubule associated cell migration factor |
| Q5VV42 | CDKAL1 | CDK5 regulatory subunit associated protein 1 like 1 |
| Q96TC7 | RMDN3 | regulator of microtubule dynamics 3 |
| Q9NY57 | STK32B | serine/threonine kinase 32B |
| Q9NX95 | SYBU | syntabulin |
| Q9BV94 | EDEM2 | ER degradation enhancing alpha-mannosidase like protein 2 |
| Q9NZ56 | FMN2 | formin 2 |
| Q9NRR3 | CDC42SE2 | CDC42 small effector 2 |
| Q9P2F6 | ARHGAP20 | Rho GTPase activating protein 20 |
| Q7Z6J0 | SH3RF1 | SH3 domain containing ring finger 1 |
| Q9HCJ6 | VAT1L | vesicle amine transport 1 like |
| Q96PZ7 | CSMD1 | CUB and Sushi multiple domains 1 |
| Q86XI6 | PPP1R3B | protein phosphatase 1 regulatory subunit 3B |
| Q3KP44 | ANKRD55 | ankyrin repeat domain 55 |
| Q96JQ2 | CLMN | calmin |
| Q3SY56 | SP6 | Sp6 transcription factor |
| Q8TCT8 | SPPL2A | signal peptide peptidase like 2A |
| Q8TF40 | FNIP1 | folliculin interacting protein 1 |
| Q8IXS6 | PALM2 | paralemmin 2 |
| Q9BZF3 | OSBPL6 | oxysterol binding protein like 6 |
| Q5S007 | LRRK2 | leucine rich repeat kinase 2 |
| Q32M45 | ANO4 | anoctamin 4 |
| Q330K2 | NDUFAF6 | NADH:ubiquinone oxidoreductase complex assembly factor 6 |
| Q96LZ7 | RMDN2 | regulator of microtubule dynamics 2 |
| Q5VXU1 | NKAIN2 | sodium/potassium transporting ATPase interacting 2 |
| Q8N8U9 | BMPER | BMP binding endothelial regulator |
| Q5VZY2 | PLPP4 | phospholipid phosphatase 4 |
| Q96LT7 | C9orf72 | chromosome 9 open reading frame 72 |
| Q8IWF9 | CCDC83 | coiled-coil domain containing 83 |
| Q8IWL8 | STH | saitohin |
| Q8IU99 | CALHM1 | calcium homeostasis modulator 1 |
| O75864 | PPP1R37 | protein phosphatase 1 regulatory subunit 37 |
| Q17RQ9 | NKPD1 | NTPase KAP family P-loop domain containing 1 |
|  | EPHA1-AS1 | EPHA1 antisense RNA 1 |
| Q86TE4 | LUZP2 | leucine zipper protein 2 |
| O95932 | TGM6 | transglutaminase 6 |
| D6RGH6 | MCIDAS | multiciliate differentiation and DNA synthesis associated cell cycle protein |
| Q8NDZ2 | SIMC1 | SUMO interacting motifs containing 1 |
| Q6UWF3 | SCIMP | SLP adaptor and CSK interacting membrane protein |
|  | LINC01567 | long intergenic non-protein coding RNA 1567 |
| Q9Y2D5 | PALM2-AKAP2 | PALM2-AKAP2 readthrough |
|  | LINC01184 | long intergenic non-protein coding RNA 1184 |
| A0A087WXM9 | MEIKIN | meiotic kinetochore factor |
|  | SLC8A1-AS1 | SLC8A1 antisense RNA 1 |
| Q8IVW1 | ARL17B | ADP ribosylation factor like GTPase 17B |
|  | TSPOAP1-AS1 | TSPOAP1 antisense RNA 1 |
|  | LINC01725 | long intergenic non-protein coding RNA 1725 |
|  | MEF2C-AS1 | MEF2C antisense RNA 1 |
|  | LINC00972 | long intergenic non-protein coding RNA 972 |
| Q9BZC7 | ABCA2 | ATP binding cassette subfamily A member 2 |
| P05090 | APOD | apolipoprotein D |
| P25445 | FAS | Fas cell surface death receptor |
| P51587 | BRCA2 | BRCA2, DNA repair associated |
| P0DP23;  P0DP24;  P0DP25 | CALM2 | calmodulin 2 |
| P0DP23;  P0DP24;  P0DP25 | CALM3 | calmodulin 3 |
| P21964 | COMT | catechol-O-methyltransferase |
| P11511 | CYP19A1 | cytochrome P450 family 19 subfamily A member 1 |
| P29323 | EPHB2 | EPH receptor B2 |
| Q9BXW9 | FANCD2 | FA complementation group D2 |
| P01579 | IFNG | interferon gamma |
| P06858 | LPL | lipoprotein lipase |
| P19838 | NFKB1 | nuclear factor kappa B subunit 1 |
| O15118 | NPC1 | NPC intracellular cholesterol transporter 1 |
| Q02218 | OGDH | oxoglutarate dehydrogenase |
| Q01959 | SLC6A3 | solute carrier family 6 member 3 |
| P98155 | VLDLR | very low density lipoprotein receptor |
| P02768 | ALB | albumin |
| P02654 | APOC1 | apolipoprotein C1 |
| P16671 | CD36 | CD36 molecule |
| P25942 | CD40 | CD40 molecule |
| P36957 | DLST | dihydrolipoamide S-succinyltransferase |
| Q05639 | EEF1A2 | eukaryotic translation elongation factor 1 alpha 2 |
| P04150 | NR3C1 | nuclear receptor subfamily 3 group C member 1 |
| P08603 | CFH | complement factor H |
| P07196 | NEFL | neurofilament light |
| P00747 | PLG | plasminogen |
| P45983 | MAPK8 | mitogen-activated protein kinase 8 |
| P19525 | EIF2AK2 | eukaryotic translation initiation factor 2 alpha kinase 2 |
| P60880 | SNAP25 | synaptosome associated protein 25 |
| O00206 | TLR4 | toll like receptor 4 |
| Q6ZSZ6 | TSHZ1 | teashirt zinc finger homeobox 1 |
| P27348 | YWHAQ | tyrosine 3-monooxygenase/tryptophan 5-monooxygenase activation protein theta |
| Q9BXS0 | COL25A1 | collagen type XXV alpha 1 chain |
| P07384 | CAPN1 | calpain 1 |
| P06493 | CDK1 | cyclin dependent kinase 1 |
| P16220 | CREB1 | cAMP responsive element binding protein 1 |
| P53355 | DAPK1 | death associated protein kinase 1 |
| P17677 | GAP43 | growth associated protein 43 |
| P10997 | IAPP | islet amyloid polypeptide |
| P25713 | MT3 | metallothionein 3 |
| P42336 | PIK3CA | phosphatidylinositol-4,5-bisphosphate 3-kinase catalytic subunit alpha |
| P48736 | PIK3CG | phosphatidylinositol-4,5-bisphosphate 3-kinase catalytic subunit gamma |
| P04054 | PLA2G1B | phospholipase A2 group IB |
| Q12800 | TFCP2 | transcription factor CP2 |
| P63104 | YWHAZ | tyrosine 3-monooxygenase/tryptophan 5-monooxygenase activation protein zeta |
| O95197 | RTN3 | reticulon 3 |
| O60760 | HPGDS | hematopoietic prostaglandin D synthase |
| Q8WY21 | SORCS1 | sortilin related VPS10 domain containing receptor 1 |
| Q6UXR4 | SERPINA13P | serpin family A member 13, pseudogene |
|  | MIR132 | microRNA 132 |
| P24752 | ACAT1 | acetyl-CoA acetyltransferase 1 |
| P09874 | PARP1 | poly(ADP-ribose) polymerase 1 |
| Q02410 | APBA1 | amyloid beta precursor protein binding family A member 1 |
| P35670 | ATP7B | ATPase copper transporting beta |
| P55212 | CASP6 | caspase 6 |
| P41180 | CASR | calcium sensing receptor |
| P16070 | CD44 | CD44 molecule (Indian blood group) |
| Q12798 | CETN1 | centrin 1 |
| P10176 | COX8A | cytochrome c oxidase subunit 8A |
| Q92793 | CREBBP | CREB binding protein |
| P06241 | FYN | FYN proto-oncogene, Src family tyrosine kinase |
| P06396 | GSN | gelsolin |
| P18510 | IL1RN | interleukin 1 receptor antagonist |
| P29459 | IL12A | interleukin 12A |
| Q14116 | IL18 | interleukin 18 |
| P11215 | ITGAM | integrin subunit alpha M |
| P22001 | KCNA3 | potassium voltage-gated channel subfamily A member 3 |
| P21397 | MAOA | monoamine oxidase A |
| P29475 | NOS1 | nitric oxide synthase 1 |
| P78380 | OLR1 | oxidized low density lipoprotein receptor 1 |
| P05121 | SERPINE1 | serpin family E member 1 |
| P42338 | PIK3CB | phosphatidylinositol-4,5-bisphosphate 3-kinase catalytic subunit beta |
| O00329 | PIK3CD | phosphatidylinositol-4,5-bisphosphate 3-kinase catalytic subunit delta |
| P30613 | PKLR | pyruvate kinase L/R |
| P28340 | POLD1 | DNA polymerase delta 1, catalytic subunit |
| P20339 | RAB5A | RAB5A, member RAS oncogene family |
| Q99523 | SORT1 | sortilin 1 |
| P35610 | SOAT1 | sterol O-acyltransferase 1 |
| P04155 | TFF1 | trefoil factor 1 |
| P04216 | THY1 | Thy-1 cell surface antigen |
| O43583 | DENR | density regulated re-initiation and release factor |
| P20132 | SDS | serine dehydratase |
| Q8IV08 | PLD3 | phospholipase D family member 3 |
| Q16769 | QPCT | glutaminyl-peptide cyclotransferase |
| Q9BRK5 | SDF4 | stromal cell derived factor 4 |
| Q6P1J6 | PLB1 | phospholipase B1 |
| Q8NA03 | FSIP1 | fibrous sheath interacting protein 1 |
|  | TAS2R62P | taste 2 receptor member 62 pseudogene |
|  | TAS2R64P | taste 2 receptor member 64 pseudogene |
| P78363 | ABCA4 | ATP binding cassette subfamily A member 4 |
| P60709 | ACTB | actin beta |
| P01019 | AGT | angiotensinogen |
| P05091 | ALDH2 | aldehyde dehydrogenase 2 family member |
| P04083 | ANXA1 | annexin A1 |
| P51693 | APLP1 | amyloid beta precursor like protein 1 |
| P53004 | BLVRA | biliverdin reductase A |
| P0C0L4;  P0C0L5 | C4A | complement C4A (Rodgers blood group) |
| P0C0L4;  P0C0L5 | C4B | complement C4B (Chido blood group) |
| Q08345 | DDR1 | discoidin domain receptor tyrosine kinase 1 |
| P05937 | CALB1 | calbindin 1 |
| P20810 | CAST | calpastatin |
| P29965 | CD40LG | CD40 ligand |
| P34972 | CNR2 | cannabinoid receptor 2 |
| Q14194 | CRMP1 | collapsin response mediator protein 1 |
| Q16643 | DBN1 | drebrin 1 |
| Q16698 | DECR1 | 2,4-dienoyl-CoA reductase 1 |
| P42892 | ECE1 | endothelin converting enzyme 1 |
| P08246 | ELANE | elastase, neutrophil expressed |
| Q15485 | FCN2 | ficolin 2 |
| P30047 | GCHFR | GTP cyclohydrolase I feedback regulator |
| P41594 | GRM5 | glutamate metabotropic receptor 5 |
| P32780 | GTF2H1 | general transcription factor IIH subunit 1 |
| P10144 | GZMB | granzyme B |
| P04035 | HMGCR | 3-hydroxy-3-methylglutaryl-CoA reductase |
| P34932 | HSPA4 | heat shock protein family A (Hsp70) member 4 |
| P35858 | IGFALS | insulin like growth factor binding protein acid labile subunit |
| P17936 | IGFBP3 | insulin like growth factor binding protein 3 |
| P80188 | LCN2 | lipocalin 2 |
| P08519 | LPA | lipoprotein(a) |
| P08254 | MMP3 | matrix metallopeptidase 3 |
| P51948 | MNAT1 | MNAT1, CDK activating kinase assembly factor |
| P00395 | COX1 | cytochrome c oxidase subunit I |
| Q96IZ0 | PAWR | pro-apoptotic WT1 regulator |
| P01009 | SERPINA1 | serpin family A member 1 |
| P14555 | PLA2G2A | phospholipase A2 group IIA |
| P47712 | PLA2G4A | phospholipase A2 group IVA |
| P55058 | PLTP | phospholipid transfer protein |
| P27361 | MAPK3 | mitogen-activated protein kinase 3 |
| P08922 | ROS1 | ROS proto-oncogene 1, receptor tyrosine kinase |
| P40763 | STAT3 | signal transducer and activator of transcription 3 |
| P07101 | TH | tyrosine hydroxylase |
| O60603 | TLR2 | toll like receptor 2 |
| P19438 | TNFRSF1A | TNF receptor superfamily member 1A |
| P46939 | UTRN | utrophin |
| P21796 | VDAC1 | voltage dependent anion channel 1 |
| P11473 | VDR | vitamin D receptor |
| O95185 | UNC5C | unc-5 netrin receptor C |
| Q9UBN7 | HDAC6 | histone deacetylase 6 |
| Q07666 | KHDRBS1 | KH RNA binding domain containing, signal transduction associated 1 |
| O94811 | TPPP | tubulin polymerization promoting protein |
| O94907 | DKK1 | dickkopf WNT signaling pathway inhibitor 1 |
| Q9NTG7 | SIRT3 | sirtuin 3 |
| Q9UIK4 | DAPK2 | death associated protein kinase 2 |
| P37198 | NUP62 | nucleoporin 62 |
| Q9UI47 | CTNNA3 | catenin alpha 3 |
| P22466 | GAL | galanin and GMAP prepropeptide |
| Q9UJW0 | DCTN4 | dynactin subunit 4 |
| A4D1B5 | GSAP | gamma-secretase activating protein |
| Q9NUM4 | TMEM106B | transmembrane protein 106B |
| Q9H2E6 | SEMA6A | semaphorin 6A |
| Q9BXM7 | PINK1 | PTEN induced putative kinase 1 |
| Q9BZR6 | RTN4R | reticulon 4 receptor |
| Q96FF9 | CDCA5 | cell division cycle associated 5 |
| Q86UG4 | SLCO6A1 | solute carrier organic anion transporter family member 6A1 |
| P0C0L4;  P0C0L5 | C4B_2 | complement component 4B (Chido blood group), copy 2 |
|  | BACE1-AS | BACE1 antisense RNA |
| Q9BT76 | UPK3B | uroplakin 3B |
| P29274 | ADORA2A | adenosine A2a receptor |
| P04114 | APOB | apolipoprotein B |
| P55087 | AQP4 | aquaporin 4 |
| P10275 | AR | androgen receptor |
| P18848 | ATF4 | activating transcription factor 4 |
| B1AH88;  P30536 | TSPO | translocator protein |
| P01024 | C3 | complement C3 |
| P13987 | CD59 | CD59 molecule (CD59 blood group) |
| P34810 | CD68 | CD68 molecule |
| P02511 | CRYAB | crystallin alpha B |
| P50570 | DNM2 | dynamin 2 |
| P18146 | EGR1 | early growth response 1 |
| P06730 | EIF4E | eukaryotic translation initiation factor 4E |
| Q7KZI7 | MARK2 | microtubule affinity regulating kinase 2 |
| P15036 | ETS2 | ETS proto-oncogene 2, transcription factor |
| P05230 | FGF1 | fibroblast growth factor 1 |
| Q06546 | GABPA | GA binding protein transcription factor subunit alpha |
| P01275 | GCG | glucagon |
| P39905 | GDNF | glial cell derived neurotrophic factor |
| P01241 | GH1 | growth hormone 1 |
| P42261 | GRIA1 | glutamate ionotropic receptor AMPA type subunit 1 |
| P42262 | GRIA2 | glutamate ionotropic receptor AMPA type subunit 2 |
| Q14416 | GRM2 | glutamate metabotropic receptor 2 |
| O43612 | HCRT | hypocretin neuropeptide precursor |
| Q02297 | NRG1 | neuregulin 1 |
| P17096 | HMGA1 | high mobility group AT-hook 1 |
| P13985 | HRES1 | HTLV-1 related endogenous sequence |
| P28845 | HSD11B1 | hydroxysteroid 11-beta dehydrogenase 1 |
| P0DMV8;  P0DMV9 | HSPA1A | heat shock protein family A (Hsp70) member 1A |
| P0DMV8;  P0DMV9 | HSPA1B | heat shock protein family A (Hsp70) member 1B |
| P11021 | HSPA5 | heat shock protein family A (Hsp70) member 5 |
| P07900 | HSP90AA1 | heat shock protein 90 alpha family class A member 1 |
| P98160 | HSPG2 | heparan sulfate proteoglycan 2 |
| P05112 | IL4 | interleukin 4 |
| P10145 | CXCL8 | C-X-C motif chemokine ligand 8 |
| P35225 | IL13 | interleukin 13 |
| Q07866 | KLC1 | kinesin light chain 1 |
| P98164 | LRP2 | LDL receptor related protein 2 |
| Q09327 | MGAT3 | mannosyl (beta-1,4-)-glycoprotein beta-1,4-N-acetylglucosaminyltransferase |
| P04629 | NTRK1 | neurotrophic receptor tyrosine kinase 1 |
| P30989 | NTSR1 | neurotensin receptor 1 |
| O15527 | OGG1 | 8-oxoguanine DNA glycosylase |
| Q15165 | PON2 | paraoxonase 2 |
| Q07869 | PPARA | peroxisome proliferator activated receptor alpha |
| P41222 | PTGDS | prostaglandin D2 synthase |
| P08575 | PTPRC | protein tyrosine phosphatase, receptor type C |
| P63000 | RAC1 | Rac family small GTPase 1 |
| P04000 | OPN1LW | opsin 1, long wave sensitive |
| P00797 | REN | renin |
| P06702 | S100A9 | S100 calcium binding protein A9 |
| Q16143 | SNCB | synuclein beta |
| P08047 | SP1 | Sp1 transcription factor |
| P20226 | TBP | TATA-box binding protein |
| P32119 | PRDX2 | peroxiredoxin 2 |
| P21980 | TGM2 | transglutaminase 2 |
| P20333 | TNFRSF1B | TNF receptor superfamily member 1B |
| P19320 | VCAM1 | vascular cell adhesion molecule 1 |
| P08670 | VIM | vimentin |
| O60733 | PLA2G6 | phospholipase A2 group VI |
| O76074 | PDE5A | phosphodiesterase 5A |
| Q14457 | BECN1 | beclin 1 |
| Q9UBH6 | XPR1 | xenotropic and polytropic retrovirus receptor 1 |
| Q9UNQ0 | ABCG2 | ATP binding cassette subfamily G member 2 (Junior blood group) |
| P78417 | GSTO1 | glutathione S-transferase omega 1 |
| P45844 | ABCG1 | ATP binding cassette subfamily G member 1 |
| P68363 | TUBA1B | tubulin alpha 1b |
| Q92993 | KAT5 | lysine acetyltransferase 5 |
| O60502 | OGA | O-GlcNAcase |
| Q8IXJ6 | SIRT2 | sirtuin 2 |
| Q7Z3E1 | TIPARP | TCDD inducible poly(ADP-ribose) polymerase |
| Q96HU1 | SGSM3 | small G protein signaling modulator 3 |
| Q9NR96 | TLR9 | toll like receptor 9 |
| Q9BVK6 | TMED9 | transmembrane p24 trafficking protein 9 |
| Q96QK1 | VPS35 | VPS35, retromer complex component |
| Q9NWQ8 | PAG1 | phosphoprotein membrane anchor with glycosphingolipid microdomains 1 |
| Q9NZ42 | PSENEN | presenilin enhancer, gamma-secretase subunit |
| P57723 | PCBP4 | poly(rC) binding protein 4 |
| Q495T6 | MMEL1 | membrane metalloendopeptidase like 1 |
| Q5T2D2 | TREML2 | triggering receptor expressed on myeloid cells like 2 |
| Q8WW43 | APH1B | aph-1 homolog B, gamma-secretase subunit |
| Q9BTE1 | DCTN5 | dynactin subunit 5 |
| Q5TCY1 | TTBK1 | tau tubulin kinase 1 |
| Q86WG3 | ATCAY | ATCAY, caytaxin |
| Q86VH5 | LRRTM3 | leucine rich repeat transmembrane neuronal 3 |
| Q9Y2Q3 | GSTK1 | glutathione S-transferase kappa 1 |
| A5A3E0 | POTEF | POTE ankyrin domain family member F |
|  | PGR-AS1 | PGR antisense RNA 1 |
| P25098 | GRK2 | G protein-coupled receptor kinase 2 |
| P50052 | AGTR2 | angiotensin II receptor type 2 |
| P55008 | AIF1 | allograft inflammatory factor 1 |
| P16050 | ALOX15 | arachidonate 15-lipoxygenase |
| P16157 | ANK1 | ankyrin 1 |
| P02743 | APCS | amyloid P component, serum |
| P06727 | APOA4 | apolipoprotein A4 |
| P07741 | APRT | adenine phosphoribosyltransferase |
| P29972 | AQP1 | aquaporin 1 (Colton blood group) |
| O00327 | ARNTL | aryl hydrocarbon receptor nuclear translocator like |
| P36575 | ARR3 | arrestin 3 |
| P08842 | STS | steroid sulfatase |
| Q13315 | ATM | ATM serine/threonine kinase |
| P38398 | BRCA1 | BRCA1, DNA repair associated |
| P35613 | BSG | basigin (Ok blood group) |
| P06681 | C2 | complement C2 |
| P21730 | C5AR1 | complement C5a receptor 1 |
| Q9UQM7 | CAMK2A | calcium/calmodulin dependent protein kinase II alpha |
| P29466 | CASP1 | caspase 1 |
| P0DN79;  P35520 | CBS | cystathionine-beta-synthase |
| P11802 | CDK4 | cyclin dependent kinase 4 |
| P49716 | CEBPD | CCAAT enhancer binding protein delta |
| P36222 | CHI3L1 | chitinase 3 like 1 |
| P08172 | CHRM2 | cholinergic receptor muscarinic 2 |
| P26441 | CNTF | ciliary neurotrophic factor |
| P36551 | CPOX | coproporphyrinogen oxidase |
| P34998 | CRHR1 | corticotropin releasing hormone receptor 1 |
| P39880;  Q13948 | CUX1 | cut like homeobox 1 |
| P49238 | CX3CR1 | C-X3-C motif chemokine receptor 1 |
| P78310 | CXADR | CXADR, Ig-like cell adhesion molecule |
| P20813 | CYP2B6 | cytochrome P450 family 2 subfamily B member 6 |
| P08684 | CYP3A4 | cytochrome P450 family 3 subfamily A member 4 |
| O75553 | DAB1 | DAB1, reelin adaptor protein |
| P09172 | DBH | dopamine beta-hydroxylase |
| O43602 | DCX | doublecortin |
| Q05193 | DNM1 | dynamin 1 |
| P21917 | DRD4 | dopamine receptor D4 |
| Q12926 | ELAVL2 | ELAV like RNA binding protein 2 |
| P41970 | ELK3 | ELK3, ETS transcription factor |
| Q15303 | ERBB4 | erb-b2 receptor tyrosine kinase 4 |
| P25116 | F2R | coagulation factor II thrombin receptor |
| P14324 | FDPS | farnesyl diphosphate synthase |
| P09038 | FGF2 | fibroblast growth factor 2 |
| Q02790 | FKBP4 | FK506 binding protein 4 |
| P25090 | FPR2 | formyl peptide receptor 2 |
| P15104 | GLUL | glutamate-ammonia ligase |
| Q12879 | GRIN2A | glutamate ionotropic receptor NMDA type subunit 2A |
| P09488 | GSTM1 | glutathione S-transferase mu 1 |
| P21266 | GSTM3 | glutathione S-transferase mu 3 |
| P09211 | GSTP1 | glutathione S-transferase pi 1 |
| P42858 | HTT | huntingtin |
| P61086 | UBE2K | ubiquitin conjugating enzyme E2 K |
| P01891;  P01892;  P04439;  P05534;  P10314;  P10316;  P13746;  P16188;  P16189;  P16190;  P18462;  P30443;  P30447;  P30450;  P30453;  P30455;  P30456;  P30457;  P30459;  P30512;  Q09160 | HLA-A | major histocompatibility complex, class I, A |
| P01911;  P01912;  P04229;  P13760;  P13761;  P20039;  Q29974;  Q30134;  Q30167;  Q5Y7A7;  Q95IE3;  Q9GIY3;  Q9TQE0 | HLA-DRB1 | major histocompatibility complex, class II, DR beta 1 |
| P09651 | HNRNPA1 | heterogeneous nuclear ribonucleoprotein A1 |
| P38646 | HSPA9 | heat shock protein family A (Hsp70) member 9 |
| Q16082 | HSPB2 | heat shock protein family B (small) member 2 |
| Q13639 | HTR4 | 5-hydroxytryptamine receptor 4 |
| P50406 | HTR6 | 5-hydroxytryptamine receptor 6 |
| P05362 | ICAM1 | intercellular adhesion molecule 1 |
| P60568 | IL2 | interleukin 2 |
| P20702 | ITGAX | integrin subunit alpha X |
| P01042 | KNG1 | kininogen 1 |
| P22888 | LHCGR | luteinizing hormone/choriogonadotropin receptor |
| P38571 | LIPA | lipase A, lysosomal acid type |
| P02686 | MBP | myelin basic protein |
| P08582 | MELTF | melanotransferrin |
| P08253 | MMP2 | matrix metallopeptidase 2 |
| Q14511 | NEDD9 | neural precursor cell expressed, developmentally down-regulated 9 |
| Q13469 | NFATC2 | nuclear factor of activated T cells 2 |
| Q12857 | NFIA | nuclear factor I A |
| O00712 | NFIB | nuclear factor I B |
| Q14938 | NFIX | nuclear factor I X |
| P46531 | NOTCH1 | notch 1 |
| Q9UM47 | NOTCH3 | notch 3 |
| O60260 | PRKN | parkin RBR E3 ubiquitin protein ligase |
| P35558 | PCK1 | phosphoenolpyruvate carboxykinase 1 |
| P12004 | PCNA | proliferating cell nuclear antigen |
| P16284 | PECAM1 | platelet and endothelial cell adhesion molecule 1 |
| O43189 | PHF1 | PHD finger protein 1 |
| P01298 | PPY | pancreatic polypeptide |
| Q13131 | PRKAA1 | protein kinase AMP-activated catalytic subunit alpha 1 |
| P54646 | PRKAA2 | protein kinase AMP-activated catalytic subunit alpha 2 |
| Q9Y478 | PRKAB1 | protein kinase AMP-activated non-catalytic subunit beta 1 |
| P10644 | PRKAR1A | protein kinase cAMP-dependent type I regulatory subunit alpha |
| Q02750 | MAP2K1 | mitogen-activated protein kinase kinase 1 |
| Q92876 | KLK6 | kallikrein related peptidase 6 |
| P26599 | PTBP1 | polypyrimidine tract binding protein 1 |
| P23443 | RPS6KB1 | ribosomal protein S6 kinase B1 |
| P23297 | S100A1 | S100 calcium binding protein A1 |
| P13501 | CCL5 | C-C motif chemokine ligand 5 |
| P78423 | CX3CL1 | C-X3-C motif chemokine ligand 1 |
| Q01105 | SET | SET nuclear proto-oncogene |
| P23975 | SLC6A2 | solute carrier family 6 member 2 |
| O76070 | SNCG | synuclein gamma |
| Q9UQ90 | SPG7 | SPG7, paraplegin matrix AAA peptidase subunit |
| P78362 | SRPK2 | SRSF protein kinase 2 |
| P78536 | ADAM17 | ADAM metallopeptidase domain 17 |
| Q03519 | TAP2 | transporter 2, ATP binding cassette subfamily B member |
| P17735 | TAT | tyrosine aminotransferase |
| O15350 | TP73 | tumor protein p73 |
| P49815 | TSC2 | TSC complex subunit 2 |
| O43914 | TYROBP | TYRO protein tyrosine kinase binding protein |
| P55055 | NR1H2 | nuclear receptor subfamily 1 group H member 2 |
| P17861 | XBP1 | X-box binding protein 1 |
| Q9NQW7 | XPNPEP1 | X-prolyl aminopeptidase 1 |
| P25490 | YY1 | YY1 transcription factor |
| Q13093 | PLA2G7 | phospholipase A2 group VII |
| Q92570 | NR4A3 | nuclear receptor subfamily 4 group A member 3 |
| Q13310 | PABPC4 | poly(A) binding protein cytoplasmic 4 |
| O75469 | NR1I2 | nuclear receptor subfamily 1 group I member 2 |
| O43426 | SYNJ1 | synaptojanin 1 |
| Q96RI0 | F2RL3 | F2R like thrombin or trypsin receptor 3 |
| O95992 | CH25H | cholesterol 25-hydroxylase |
| Q13427 | PPIG | peptidylprolyl isomerase G |
| Q9UEF7 | KL | klotho |
| O15516 | CLOCK | clock circadian regulator |
| Q14994 | NR1I3 | nuclear receptor subfamily 1 group I member 3 |
| Q96S59 | RANBP9 | RAN binding protein 9 |
| Q9NP58 | ABCB6 | ATP binding cassette subfamily B member 6 (Langereis blood group) |
| O60706 | ABCC9 | ATP binding cassette subfamily C member 9 |
| O60858 | TRIM13 | tripartite motif containing 13 |
| Q99720 | SIGMAR1 | sigma non-opioid intracellular receptor 1 |
| P14550 | AKR1A1 | aldo-keto reductase family 1 member A1 |
| Q13901 | C1D | C1D nuclear receptor corepressor |
| O75689 | ADAP1 | ArfGAP with dual PH domains 1 |
| O96017 | CHEK2 | checkpoint kinase 2 |
| Q9C000 | NLRP1 | NLR family pyrin domain containing 1 |
| Q96T58 | SPEN | spen family transcriptional repressor |
| Q9NZ52 | GGA3 | golgi associated, gamma adaptin ear containing, ARF binding protein 3 |
| Q6XZF7 | DNMBP | dynamin binding protein |
| Q6UUV9 | CRTC1 | CREB regulated transcription coactivator 1 |
| Q9ULZ3 | PYCARD | PYD and CARD domain containing |
| Q9Y2W7 | KCNIP3 | potassium voltage-gated channel interacting protein 3 |
| Q96BI3 | APH1A | aph-1 homolog A, gamma-secretase subunit |
| Q8NBJ4 | GOLM1 | golgi membrane protein 1 |
| Q8N427 | NME8 | NME/NM23 family member 8 |
| Q9NZT1 | CALML5 | calmodulin like 5 |
| Q5K651 | SAMD9 | sterile alpha motif domain containing 9 |
| Q9HBW1 | LRRC4 | leucine rich repeat containing 4 |
| Q9C0B1 | FTO | FTO, alpha-ketoglutarate dependent dioxygenase |
| Q9HAB3 | SLC52A2 | solute carrier family 52 member 2 |
| Q96P20 | NLRP3 | NLR family pyrin domain containing 3 |
| Q5FWF5 | ESCO1 | establishment of sister chromatid cohesion N-acetyltransferase 1 |
| Q9H4Y5 | GSTO2 | glutathione S-transferase omega 2 |
|  | PWAR1 | Prader Willi/Angelman region RNA 1 |
| Q8N6N3 | C1orf52 | chromosome 1 open reading frame 52 |
| Q96DS6 | MS4A6E | membrane spanning 4-domains A6E |
|  | PWAR4 | Prader Willi/Angelman region RNA 4 |
|  | MIR107 | microRNA 107 |
|  | MIR29A | microRNA 29a |
|  | MIR342 | microRNA 342 |
|  | CXADRP1 | CXADR pseudogene 1 |
|  | HNRNPA1P10 | heterogeneous nuclear ribonucleoprotein A1 pseudogene 10 |
|  | C20orf181 | chromosome 20 open reading frame 181 |
|  | AD11 | Alzheimer disease-11 |
| P34998 | LINC02210-CRHR1 | LINC02210-CRHR1 readthrough |
| P11245 | NAT2 | N-acetyltransferase 2 |
| P16442 | ABO | ABO, alpha 1-3-N-acetylgalactosaminyltransferase and alpha 1-3-galactosyltransferase |
| P21399 | ACO1 | aconitase 1 |
| P08588 | ADRB1 | adrenoceptor beta 1 |
| P07550 | ADRB2 | adrenoceptor beta 2 |
| P30556 | AGTR1 | angiotensin II receptor type 1 |
| P31749 | AKT1 | AKT serine/threonine kinase 1 |
| P08758 | ANXA5 | annexin A5 |
| P08133 | ANXA6 | annexin A6 |
| P25054 | APC | APC, WNT signaling pathway regulator |
| P02655 | APOC2 | apolipoprotein C2 |
| P32121 | ARRB2 | arrestin beta 2 |
| O14867 | BACH1 | BTB domain and CNC homolog 1 |
| Q99933 | BAG1 | BCL2 associated athanogene 1 |
| P00751 | CFB | complement factor B |
| P12644 | BMP4 | bone morphogenetic protein 4 |
| P05155 | SERPING1 | serpin family G member 1 |
| P27797 | CALR | calreticulin |
| Q14790 | CASP8 | caspase 8 |
| P08571 | CD14 | CD14 molecule |
| Q07108 | CD69 | CD69 molecule |
| P12830 | CDH1 | cadherin 1 |
| P38936 | CDKN1A | cyclin dependent kinase inhibitor 1A |
| P46527 | CDKN1B | cyclin dependent kinase inhibitor 1B |
| P42771;  Q8N726 | CDKN2A | cyclin dependent kinase inhibitor 2A |
| P17676 | CEBPB | CCAAT enhancer binding protein beta |
| P23141 | CES1 | carboxylesterase 1 |
| P17516 | AKR1C4 | aldo-keto reductase family 1 member C4 |
| O14757 | CHEK1 | checkpoint kinase 1 |
| P24386 | CHM | CHM, Rab escort protein 1 |
| P32297 | CHRNA3 | cholinergic receptor nicotinic alpha 3 subunit |
| P12277 | CKB | creatine kinase B |
| P21554 | CNR1 | cannabinoid receptor 1 |
| P02461 | COL3A1 | collagen type III alpha 1 chain |
| P46108 | CRK | CRK proto-oncogene, adaptor protein |
| P09603 | CSF1 | colony stimulating factor 1 |
| P48730 | CSNK1D | casein kinase 1 delta |
| P29279 | CTGF | connective tissue growth factor |
| Q9UQB3 | CTNND2 | catenin delta 2 |
| P11712 | CYP2C9 | cytochrome P450 family 2 subfamily C member 9 |
| P24522 | GADD45A | growth arrest and DNA damage inducible alpha |
| P35638 | DDIT3 | DNA damage inducible transcript 3 |
| P15559 | NQO1 | NAD(P)H quinone dehydrogenase 1 |
| P09622 | DLD | dihydrolipoamide dehydrogenase |
| Q14195 | DPYSL3 | dihydropyrimidinase like 3 |
| P21728 | DRD1 | dopamine receptor D1 |
| Q01094 | E2F1 | E2F transcription factor 1 |
| P13639 | EEF2 | eukaryotic translation elongation factor 2 |
| P00533 | EGFR | epidermal growth factor receptor |
| P00488 | F13A1 | coagulation factor XIII A chain |
| O00519 | FAAH | fatty acid amide hydrolase |
| Q13451 | FKBP5 | FK506 binding protein 5 |
| P20930 | FLG | filaggrin |
| P21333 | FLNA | filamin A |
| P02751 | FN1 | fibronectin 1 |
| P01100 | FOS | Fos proto-oncogene, AP-1 transcription factor subunit |
| Q16595 | FXN | frataxin |
| Q99259 | GAD1 | glutamate decarboxylase 1 |
| P04062 | GBA | glucosylceramidase beta |
| P09681 | GIP | gastric inhibitory polypeptide |
| P17302 | GJA1 | gap junction protein alpha 1 |
| P16278 | GLB1 | galactosidase beta 1 |
| Q04760 | GLO1 | glyoxalase I |
| P43220 | GLP1R | glucagon like peptide 1 receptor |
| P35052 | GPC1 | glypican 1 |
| P62993 | GRB2 | growth factor receptor bound protein 2 |
| Q05586 | GRIN1 | glutamate ionotropic receptor NMDA type subunit 1 |
| P30711 | GSTT1 | glutathione S-transferase theta 1 |
| P14317 | HCLS1 | hematopoietic cell-specific Lyn substrate 1 |
| Q92769 | HDAC2 | histone deacetylase 2 |
| Q03014 | HHEX | hematopoietically expressed homeobox |
| P30519 | HMOX2 | heme oxygenase 2 |
| P00738 | HP | haptoglobin |
| Q92902 | HPS1 | HPS1, biogenesis of lysosomal organelles complex 3 subunit 1 |
| P11142 | HSPA8 | heat shock protein family A (Hsp70) member 8 |
| P08908 | HTR1A | 5-hydroxytryptamine receptor 1A |
| P28335 | HTR2C | 5-hydroxytryptamine receptor 2C |
| P14784 | IL2RB | interleukin 2 receptor subunit beta |
| P40189 | IL6ST | interleukin 6 signal transducer |
| P15248 | IL9 | interleukin 9 |
| P25025 | CXCR2 | C-X-C motif chemokine receptor 2 |
| Q01113 | IL9R | interleukin 9 receptor |
| Q16552 | IL17A | interleukin 17A |
| Q14573 | ITPR3 | inositol 1,4,5-trisphosphate receptor type 3 |
| O60674 | JAK2 | Janus kinase 2 |
| P11279 | LAMP1 | lysosomal associated membrane protein 1 |
| P16949 | STMN1 | stathmin 1 |
| P15018 | LIF | LIF, interleukin 6 family cytokine |
| P20700 | LMNB1 | lamin B1 |
| Q9UIQ6 | LNPEP | leucyl and cystinyl aminopeptidase |
| O75581 | LRP6 | LDL receptor related protein 6 |
| Q9P0L2 | MARK1 | microtubule affinity regulating kinase 1 |
| P15529 | CD46 | CD46 molecule |
| Q06413 | MEF2C | myocyte enhancer factor 2C |
| P50222 | MEOX2 | mesenchyme homeobox 2 |
| P14174 | MIF | macrophage migration inhibitory factor |
| P01106 | MYC | MYC proto-oncogene, bHLH transcription factor |
| P53384 | NUBP1 | nucleotide binding protein 1 |
| Q14934 | NFATC4 | nuclear factor of activated T cells 4 |
|  | NM | neutrophil migration |
| Q99466 | NOTCH4 | notch 4 |
| P01160 | NPPA | natriuretic peptide A |
| P49281 | SLC11A2 | solute carrier family 11 member 2 |
| P11926 | ODC1 | ornithine decarboxylase 1 |
| P41143 | OPRD1 | opioid receptor delta 1 |
| Q99572 | P2RX7 | purinergic receptor P2X 7 |
| P41231 | P2RY2 | purinergic receptor P2Y2 |
| P07237 | P4HB | prolyl 4-hydroxylase subunit beta |
| Q06830 | PRDX1 | peroxiredoxin 1 |
| Q13153 | PAK1 | p21 (RAC1) activated kinase 1 |
| P27815 | PDE4A | phosphodiesterase 4A |
| Q15118 | PDK1 | pyruvate dehydrogenase kinase 1 |
| O14939 | PLD2 | phospholipase D2 |
| P08697 | SERPINF2 | serpin family F member 2 |
| P06746 | POLB | DNA polymerase beta |
| P01189 | POMC | proopiomelanocortin |
| Q15166 | PON3 | paraoxonase 3 |
| P14859 | POU2F1 | POU class 2 homeobox 1 |
| Q08752 | PPID | peptidylprolyl isomerase D |
| P53779 | MAPK10 | mitogen-activated protein kinase 10 |
| P36507 | MAP2K2 | mitogen-activated protein kinase kinase 2 |
| Q92743 | HTRA1 | HtrA serine peptidase 1 |
| P43116 | PTGER2 | prostaglandin E receptor 2 |
| P20336 | RAB3A | RAB3A, member RAS oncogene family |
| P20338 | RAB4A | RAB4A, member RAS oncogene family |
| P51606 | RENBP | renin binding protein |
| Q13127 | REST | RE1 silencing transcription factor |
| P62753 | RPS6 | ribosomal protein S6 |
| P19793 | RXRA | retinoid X receptor alpha |
| Q15413 | RYR3 | ryanodine receptor 3 |
| O00767 | SCD | stearoyl-CoA desaturase |
| P10147 | CCL3 | C-C motif chemokine ligand 3 |
| P51671 | CCL11 | C-C motif chemokine ligand 11 |
| P48061 | CXCL12 | C-X-C motif chemokine ligand 12 |
| Q01130 | SRSF2 | serine and arginine rich splicing factor 2 |
| P04278 | SHBG | sex hormone binding globulin |
| Q92185 | ST8SIA1 | ST8 alpha-N-acetyl-neuraminide alpha-2,8-sialyltransferase 1 |
| P11166 | SLC2A1 | solute carrier family 2 member 1 |
| P17600 | SYN1 | synapsin I |
| P10600 | TGFB3 | transforming growth factor beta 3 |
| P37173 | TGFBR2 | transforming growth factor beta receptor 2 |
| P22735 | TGM1 | transglutaminase 1 |
| P01033 | TIMP1 | TIMP metallopeptidase inhibitor 1 |
| P16035 | TIMP2 | TIMP metallopeptidase inhibitor 2 |
| Q58FF3 | HSP90B2P | heat shock protein 90 beta family member 2, pseudogene |
| P49638 | TTPA | alpha tocopherol transfer protein |
| Q16881 | TXNRD1 | thioredoxin reductase 1 |
| P63165 | SUMO1 | small ubiquitin-like modifier 1 |
| Q16739 | UGCG | UDP-glucose ceramide glucosyltransferase |
| P22415 | USF1 | upstream transcription factor 1 |
| P55072 | VCP | valosin containing protein |
| P51811 | XK | X-linked Kx blood group |
| Q9Y6M5 | SLC30A1 | solute carrier family 30 member 1 |
| Q99726 | SLC30A3 | solute carrier family 30 member 3 |
| P61073 | CXCR4 | C-X-C motif chemokine receptor 4 |
|  | CP20 | lymphocyte cytosol polypeptide, 20 kD |
| Q13155 | AIMP2 | aminoacyl tRNA synthetase complex interacting multifunctional protein 2 |
| Q9ULV1 | FZD4 | frizzled class receptor 4 |
| O15294 | OGT | O-linked N-acetylglucosamine (GlcNAc) transferase |
| Q8WXG6 | MADD | MAP kinase activating death domain |
| Q92945 | KHSRP | KH-type splicing regulatory protein |
| Q9H3D4 | TP63 | tumor protein p63 |
| O14756 | HSD17B6 | hydroxysteroid 17-beta dehydrogenase 6 |
| Q15628 | TRADD | TNFRSF1A associated via death domain |
| P50591 | TNFSF10 | TNF superfamily member 10 |
| P54257 | HAP1 | huntingtin associated protein 1 |
| O60682 | MSC | musculin |
| P20674 | COX5A | cytochrome c oxidase subunit 5A |
| O75791 | GRAP2 | GRB2 related adaptor protein 2 |
| Q9ULK4 | MED23 | mediator complex subunit 23 |
| Q9NZJ5 | EIF2AK3 | eukaryotic translation initiation factor 2 alpha kinase 3 |
| Q9UQF2 | MAPK8IP1 | mitogen-activated protein kinase 8 interacting protein 1 |
| O14684 | PTGES | prostaglandin E synthase |
| O96018 | APBA3 | amyloid beta precursor protein binding family A member 3 |
| O75436 | VPS26A | VPS26, retromer complex component A |
| P30041 | PRDX6 | peroxiredoxin 6 |
| Q9Y5K2 | KLK4 | kallikrein related peptidase 4 |
| Q9UQ26 | RIMS2 | regulating synaptic membrane exocytosis 2 |
| P56524 | HDAC4 | histone deacetylase 4 |
| Q13133 | NR1H3 | nuclear receptor subfamily 1 group H member 3 |
| P42704 | LRPPRC | leucine rich pentatricopeptide repeat containing |
| Q96CV9 | OPTN | optineurin |
| Q99996 | AKAP9 | A-kinase anchoring protein 9 |
| O75955 | FLOT1 | flotillin 1 |
| Q08648;  Q6PDA7 | SPAG11B | sperm associated antigen 11B |
| Q99784 | OLFM1 | olfactomedin 1 |
| Q14956 | GPNMB | glycoprotein nmb |
| O95433 | AHSA1 | activator of HSP90 ATPase activity 1 |
| P48681 | NES | nestin |
| Q9NYY3 | PLK2 | polo like kinase 2 |
| O75475 | PSIP1 | PC4 and SFRS1 interacting protein 1 |
| Q9UPX8 | SHANK2 | SH3 and multiple ankyrin repeat domains 2 |
| O15061 | SYNM | synemin |
| Q9H2P0 | ADNP | activity dependent neuroprotector homeobox |
| Q9UQ35 | SRRM2 | serine/arginine repetitive matrix 2 |
| Q9UBY5 | LPAR3 | lysophosphatidic acid receptor 3 |
| Q13356 | PPIL2 | peptidylprolyl isomerase like 2 |
| Q9ULV3 | CIZ1 | CDKN1A interacting zinc finger protein 1 |
| Q9NV58 | RNF19A | ring finger protein 19A, RBR E3 ubiquitin protein ligase |
| Q9UKG1 | APPL1 | adaptor protein, phosphotyrosine interacting with PH domain and leucine zipper 1 |
| Q9Y2S7 | POLDIP2 | DNA polymerase delta interacting protein 2 |
| Q9UJY5 | GGA1 | golgi associated, gamma adaptin ear containing, ARF binding protein 1 |
| Q9Y286 | SIGLEC7 | sialic acid binding Ig like lectin 7 |
| Q9NRI5 | DISC1 | DISC1 scaffold protein |
| Q6XE24 | RBMS3 | RNA binding motif single stranded interacting protein 3 |
| Q9UHG2 | PCSK1N | proprotein convertase subtilisin/kexin type 1 inhibitor |
| O43464 | HTRA2 | HtrA serine peptidase 2 |
| O00548 | DLL1 | delta like canonical Notch ligand 1 |
| O00418 | EEF2K | eukaryotic elongation factor 2 kinase |
| Q9UQF0 | ERVW-1 | endogenous retrovirus group W member 1, envelope |
| Q9NZ20 | PLA2G3 | phospholipase A2 group III |
| Q0VDF9 | HSPA14 | heat shock protein family A (Hsp70) member 14 |
| Q99811 | PRRX2 | paired related homeobox 2 |
| P56937 | HSD17B7 | hydroxysteroid 17-beta dehydrogenase 7 |
| Q9NZC7 | WWOX | WW domain containing oxidoreductase |
| Q9NX09 | DDIT4 | DNA damage inducible transcript 4 |
| Q9NRX1 | PNO1 | partner of NOB1 homolog |
| Q9P2U7 | SLC17A7 | solute carrier family 17 member 7 |
| Q9GZU1 | MCOLN1 | mucolipin 1 |
| Q9UN36 | NDRG2 | NDRG family member 2 |
| Q9HBX8 | LGR6 | leucine rich repeat containing G protein-coupled receptor 6 |
| Q9H172 | ABCG4 | ATP binding cassette subfamily G member 4 |
| Q9H4A3 | WNK1 | WNK lysine deficient protein kinase 1 |
| Q8IX18 | DHX40 | DEAH-box helicase 40 |
| Q9H9B1 | EHMT1 | euchromatic histone lysine methyltransferase 1 |
| Q13057 | COASY | Coenzyme A synthase |
| P39060 | COL18A1 | collagen type XVIII alpha 1 chain |
| Q6PI77 | BHLHB9 | basic helix-loop-helix family member b9 |
| Q8TCT9 | HM13 | histocompatibility minor 13 |
| Q8N264 | ARHGAP24 | Rho GTPase activating protein 24 |
| Q9UD71 | PPP1R1B | protein phosphatase 1 regulatory inhibitor subunit 1B |
| Q86TM6 | SYVN1 | synoviolin 1 |
| Q9BY08 | EBPL | EBP like |
| Q8TF42 | UBASH3B | ubiquitin associated and SH3 domain containing B |
| Q8IVL0 | NAV3 | neuron navigator 3 |
| P36544;  Q494W8 | CHRFAM7A | CHRNA7 (exons 5-10) and FAM7A (exons A-E) fusion |
| O95760 | IL33 | interleukin 33 |
| Q9BT17 | MTG1 | mitochondrial ribosome associated GTPase 1 |
| Q7Z6L0 | PRRT2 | proline rich transmembrane protein 2 |
| Q96LB0 | MRGPRX3 | MAS related GPR family member X3 |
| Q96LA9 | MRGPRX4 | MAS related GPR family member X4 |
| O14558 | HSPB6 | heat shock protein family B (small) member 6 |
| Q8IUH3 | RBM45 | RNA binding motif protein 45 |
| Q8TDV0 | GPR151 | G protein-coupled receptor 151 |
| Q8TDB8 | SLC2A14 | solute carrier family 2 member 14 |
| A6NFN3 | RBFOX3 | RNA binding fox-1 homolog 3 |
| Q8N165 | PDIK1L | PDLIM1 interacting kinase 1 like |
| Q8TDU6 | GPBAR1 | G protein-coupled bile acid receptor 1 |
| Q7Z7J5 | DPPA2 | developmental pluripotency associated 2 |
| Q8TDS5 | OXER1 | oxoeicosanoid receptor 1 |
| Q5T6X5 | GPRC6A | G protein-coupled receptor class C group 6 member A |
| Q96LB2 | MRGPRX1 | MAS related GPR family member X1 |
| Q86VF5 | MOGAT3 | monoacylglycerol O-acyltransferase 3 |
| O00468 | AGRN | agrin |
| P0C7Q2 | ARMS2 | age-related maculopathy susceptibility 2 |
|  | MIR206 | microRNA 206 |
|  | MIR212 | microRNA 212 |
|  | MIR29B1 | microRNA 29b-1 |
|  | MIR29B2 | microRNA 29b-2 |
|  | MIR29C | microRNA 29c |
| Q9P2K8 | EIF2AK4 | eukaryotic translation initiation factor 2 alpha kinase 4 |
| Q8TDU5 | VN1R17P | vomeronasal 1 receptor 17 pseudogene |
|  | GPR166P | G protein-coupled receptor 166 pseudogene |
|  | MIR424 | microRNA 424 |
|  | LOC643387 | TAR DNA binding protein pseudogene |
| Q08648;  Q6PDA7 | SPAG11A | sperm associated antigen 11A |
| P0C6A0 | ZGLP1 | zinc finger, GATA-like protein 1 |
| Q6UXS9 | CASP12 | caspase 12 (gene/pseudogene) |
| P0DN79;  P35520 | CBSL | cystathionine-beta-synthase like |
|  | AAVS1 | adeno-associated virus integration site 1 |
| P00519 | ABL1 | ABL proto-oncogene 1, non-receptor tyrosine kinase |
| P49748 | ACADVL | acyl-CoA dehydrogenase very long chain |
| Q99798 | ACO2 | aconitase 2 |
| P78563 | ADARB1 | adenosine deaminase, RNA specific B1 |
| Q08462 | ADCY2 | adenylate cyclase 2 |
| P18509 | ADCYAP1 | adenylate cyclase activating polypeptide 1 |
| Q9UEY8 | ADD3 | adducin 3 |
| P35318 | ADM | adrenomedullin |
| P08913 | ADRA2A | adrenoceptor alpha 2A |
| P16112 | ACAN | aggrecan |
| P35869 | AHR | aryl hydrocarbon receptor |
| P02765 | AHSG | alpha 2-HS glycoprotein |
| P18054 | ALOX12 | arachidonate 12-lipoxygenase, 12S type |
| P17707 | AMD1 | adenosylmethionine decarboxylase 1 |
|  | AMD1P2 | adenosylmethionine decarboxylase 1 pseudogene 2 |
| P49418 | AMPH | amphiphysin |
| P03950 | ANG | angiogenin |
| O15123 | ANGPT2 | angiopoietin 2 |
| Q12955 | ANK3 | ankyrin 3 |
| P12235 | SLC25A4 | solute carrier family 25 member 4 |
| O75106 | AOC2 | amine oxidase, copper containing 2 |
| Q99767 | APBA2 | amyloid beta precursor protein binding family A member 2 |
| P13798 | APEH | acylaminoacyl-peptide hydrolase |
| Q13489 | BIRC3 | baculoviral IAP repeat containing 3 |
| P98170 | XIAP | X-linked inhibitor of apoptosis |
| O15392 | BIRC5 | baculoviral IAP repeat containing 5 |
| P02652 | APOA2 | apolipoprotein A2 |
| P02656 | APOC3 | apolipoprotein C3 |
| P07288 | KLK3 | kallikrein related peptidase 3 |
| P48023 | FASLG | Fas ligand |
| P78540 | ARG2 | arginase 2 |
| P61586 | RHOA | ras homolog family member A |
| P01008 | SERPINC1 | serpin family C member 1 |
| Q15911 | ZFHX3 | zinc finger homeobox 3 |
| P54707 | ATP12A | ATPase H+/K+ transporting non-gastric alpha2 subunit |
| P20648 | ATP4A | ATPase H+/K+ transporting subunit alpha |
| P05496 | ATP5MC1 | ATP synthase membrane subunit c locus 1 |
| Q06055 | ATP5MC2 | ATP synthase membrane subunit c locus 2 |
| P18859 | ATP5PF | ATP synthase peripheral stalk subunit F6 |
| P01185 | AVP | arginine vasopressin |
| P20160 | AZU1 | azurocidin 1 |
| P24385 | CCND1 | cyclin D1 |
| Q92843 | BCL2L2 | BCL2 like 2 |
| Q9Y276 | BCS1L | BCS1 homolog, ubiquinol-cytochrome c reductase complex chaperone |
|  | BCYRN1 | brain cytoplasmic RNA 1 |
| P30411 | BDKRB2 | bradykinin receptor B2 |
| P55957 | BID | BH3 interacting domain death agonist |
| P54132 | BLM | Bloom syndrome RecQ like helicase |
| P22004 | BMP6 | bone morphogenetic protein 6 |
| P32247 | BRS3 | bombesin receptor subtype 3 |
| Q10588 | BST1 | bone marrow stromal cell antigen 1 |
| Q16581 | C3AR1 | complement C3a receptor 1 |
| P01031 | C5 | complement C5 |
| P02748 | C9 | complement C9 |
| O43822 | C21orf2 | chromosome 21 open reading frame 2 |
| P00918 | CA2 | carbonic anhydrase 2 |
| O43772 | SLC25A20 | solute carrier family 25 member 20 |
| P22676 | CALB2 | calbindin 2 |
| P01258;  P06881 | CALCA | calcitonin related polypeptide alpha |
| P30988 | CALCR | calcitonin receptor |
| Q16566 | CAMK4 | calcium/calmodulin dependent protein kinase IV |
| P40121 | CAPG | capping actin protein, gelsolin like |
| P17655 | CAPN2 | calpain 2 |
| P49662 | CASP4 | caspase 4 |
| P55210 | CASP7 | caspase 7 |
| P55211 | CASP9 | caspase 9 |
| P04040 | CAT | catalase |
| P06307 | CCK | cholecystokinin |
| P24863 | CCNC | cyclin C |
| O43866 | CD5L | CD5 molecule like |
| P01732 | CD8A | CD8a molecule |
| P11836 | MS4A1 | membrane spanning 4-domains A1 |
| Q96HJ5 | MS4A3 | membrane spanning 4-domains A3 |
| P33681 | CD80 | CD80 molecule |
| P42081 | CD86 | CD86 molecule |
| P28906 | CD34 | CD34 molecule |
| P28907 | CD38 | CD38 molecule |
| Q08722 | CD47 | CD47 molecule |
| P19397 | CD53 | CD53 molecule |
| P04233 | CD74 | CD74 molecule |
| P32320 | CDA | cytidine deaminase |
| P30305 | CDC25B | cell division cycle 25B |
| P60953 | CDC42 | cell division cycle 42 |
| P19022 | CDH2 | cadherin 2 |
| P50750 | CDK9 | cyclin dependent kinase 9 |
| P06731 | CEACAM5 | carcinoembryonic antigen related cell adhesion molecule 5 |
|  | CECR | cat eye syndrome chromosome region |
| Q9Y281 | CFL2 | cofilin 2 |
| P13569 | CFTR | cystic fibrosis transmembrane conductance regulator |
| P10645 | CHGA | chromogranin A |
| P05060 | CHGB | chromogranin B |
| P11229 | CHRM1 | cholinergic receptor muscarinic 1 |
| Q9NSE2 | CISH | cytokine inducible SH2 containing protein |
| P51790 | CLCN3 | chloride voltage-gated channel 3 |
| P49759 | CLK1 | CDC like kinase 1 |
| P49760 | CLK2 | CDC like kinase 2 |
| P51684 | CCR6 | C-C motif chemokine receptor 6 |
| Q99788 | CMKLR1 | chemerin chemokine-like receptor 1 |
| Q92887 | ABCC2 | ATP binding cassette subfamily C member 2 |
| P09543 | CNP | 2',3'-cyclic nucleotide 3' phosphodiesterase |
| P13942 | COL11A2 | collagen type XI alpha 2 chain |
| O15431 | SLC31A1 | solute carrier family 31 member 1 |
| Q12887 | COX10 | COX10, heme A:farnesyltransferase cytochrome c oxidase assembly factor |
| Q7KZN9 | COX15 | COX15, cytochrome c oxidase assembly homolog |
| P15086 | CPB1 | carboxypeptidase B1 |
| P16870 | CPE | carboxypeptidase E |
| P15169 | CPN1 | carboxypeptidase N subunit 1 |
| P15336 | ATF2 | activating transcription factor 2 |
| P07333 | CSF1R | colony stimulating factor 1 receptor |
| P09919 | CSF3 | colony stimulating factor 3 |
| P68400 | CSNK2A1 | casein kinase 2 alpha 1 |
| P13611 | VCAN | versican |
| P28325 | CST5 | cystatin D |
| Q15828 | CST6 | cystatin E/M |
| Q16619 | CTF1 | cardiotrophin 1 |
| P43235 | CTSK | cathepsin K |
| P25774 | CTSS | cathepsin S |
| Q9UBR2 | CTSZ | cathepsin Z |
| P04839 | CYBB | cytochrome b-245 beta chain |
| P05177 | CYP1A2 | cytochrome P450 family 1 subfamily A member 2 |
| P33261 | CYP2C19 | cytochrome P450 family 2 subfamily C member 19 |
| P15538 | CYP11B1 | cytochrome P450 family 11 subfamily B member 1 |
| P19099 | CYP11B2 | cytochrome P450 family 11 subfamily B member 2 |
| P05093 | CYP17A1 | cytochrome P450 family 17 subfamily A member 1 |
| Q02318 | CYP27A1 | cytochrome P450 family 27 subfamily A member 1 |
| Q9UER7 | DAXX | death domain associated protein |
| P07585 | DCN | decorin |
| P52895 | AKR1C2 | aldo-keto reductase family 1 member C2 |
| Q8WYJ6 | SEPT1 | septin 1 |
| P49895 | DIO1 | iodothyronine deiodinase 1 |
| Q92813 | DIO2 | iodothyronine deiodinase 2 |
| Q12959 | DLG1 | discs large MAGUK scaffold protein 1 |
| Q92796 | DLG3 | discs large MAGUK scaffold protein 3 |
| P11532 | DMD | dystrophin |
| Q9Y5R6 | DMRT1 | doublesex and mab-3 related transcription factor 1 |
| P26358 | DNMT1 | DNA methyltransferase 1 |
| Q92608 | DOCK2 | dedicator of cytokinesis 2 |
| P27487 | DPP4 | dipeptidyl peptidase 4 |
| P14416 | DRD2 | dopamine receptor D2 |
| P35462 | DRD3 | dopamine receptor D3 |
| P54259 | ATN1 | atrophin 1 |
| Q02413 | DSG1 | desmoglein 1 |
| Q14126 | DSG2 | desmoglein 2 |
| Q16828 | DUSP6 | dual specificity phosphatase 6 |
| O14640 | DVL1 | dishevelled segment polarity protein 1 |
| P05305 | EDN1 | endothelin 1 |
| P01133 | EGF | epidermal growth factor |
| P11161 | EGR2 | early growth response 2 |
| P41091 | EIF2S3 | eukaryotic translation initiation factor 2 subunit gamma |
| P60842 | EIF4A1 | eukaryotic translation initiation factor 4A1 |
| Q14240 | EIF4A2 | eukaryotic translation initiation factor 4A2 |
| Q13541 | EIF4EBP1 | eukaryotic translation initiation factor 4E binding protein 1 |
| P78344 | EIF4G2 | eukaryotic translation initiation factor 4 gamma 2 |
| P26378 | ELAVL4 | ELAV like RNA binding protein 4 |
| P54849 | EMP1 | epithelial membrane protein 1 |
| Q14247 | CTTN | cortactin |
| Q07075 | ENPEP | glutamyl aminopeptidase |
| Q09472 | EP300 | E1A binding protein p300 |
| P54764 | EPHA4 | EPH receptor A4 |
| P54762 | EPHB1 | EPH receptor B1 |
| P01588 | EPO | erythropoietin |
| P19235 | EPOR | erythropoietin receptor |
| P04626 | ERBB2 | erb-b2 receptor tyrosine kinase 2 |
| P11308 | ERG | ERG, ETS transcription factor |
| Q15910 | EZH2 | enhancer of zeste 2 polycomb repressive complex 2 subunit |
| P55085 | F2RL1 | F2R like trypsin receptor 1 |
| P08709 | F7 | coagulation factor VII |
| P03951 | F11 | coagulation factor XI |
| P05160 | F13B | coagulation factor XIII B chain |
| P05413 | FABP3 | fatty acid binding protein 3 |
| Q12830 | BPTF | bromodomain PHD finger transcription factor |
| O15287 | FANCG | FA complementation group G |
| P49327 | FASN | fatty acid synthase |
| Q14517 | FAT1 | FAT atypical cadherin 1 |
| Q01362 | MS4A2 | membrane spanning 4-domains A2 |
| P08637 | FCGR3A | Fc fragment of IgG receptor IIIa |
| O75015 | FCGR3B | Fc fragment of IgG receptor IIIb |
| P07332 | FES | FES proto-oncogene, tyrosine kinase |
| Q92915 | FGF14 | fibroblast growth factor 14 |
| P22607 | FGFR3 | fibroblast growth factor receptor 3 |
| Q14192 | FHL2 | four and a half LIM domains 2 |
| P62942 | FKBP1A | FK506 binding protein 1A |
| Q08050 | FOXM1 | forkhead box M1 |
| Q12778 | FOXO1 | forkhead box O1 |
| O43524 | FOXO3 | forkhead box O3 |
| O75369 | FLNB | filamin B |
| P35916 | FLT4 | fms related tyrosine kinase 4 |
| Q06787 | FMR1 | fragile X mental retardation 1 |
| Q04609 | FOLH1 | folate hydrolase 1 |
| P53539 | FOSB | FosB proto-oncogene, AP-1 transcription factor subunit |
| P42685 | FRK | fyn related Src family tyrosine kinase |
| P23945 | FSHR | follicle stimulating hormone receptor |
| Q9UBS5 | GABBR1 | gamma-aminobutyric acid type B receptor subunit 1 |
| P22102 | GART | phosphoribosylglycinamide formyltransferase, phosphoribosylglycinamide synthetase, phosphoribosylaminoimidazole synthetase |
| P55789 | GFER | growth factor, augmenter of liver regeneration |
| P56159 | GFRA1 | GDNF family receptor alpha 1 |
| O60609 | GFRA3 | GDNF family receptor alpha 3 |
| P19440 | GGT1 | gamma-glutamyltransferase 1 |
| P48165 | GJA8 | gap junction protein alpha 8 |
| P08151 | GLI1 | GLI family zinc finger 1 |
| P60983 | GMFB | glia maturation factor beta |
| P63096 | GNAI1 | G protein subunit alpha i1 |
| P16520 | GNB3 | G protein subunit beta 3 |
| Q08379 | GOLGA2 | golgin A2 |
| P06744 | GPI | glucose-6-phosphate isomerase |
| Q9UKP6 | UTS2R | urotensin 2 receptor |
| P34947 | GRK5 | G protein-coupled receptor kinase 5 |
| P24298 | GPT | glutamic--pyruvic transaminase |
| P42263 | GRIA3 | glutamate ionotropic receptor AMPA type subunit 3 |
| Q16099 | GRIK4 | glutamate ionotropic receptor kainate type subunit 4 |
| P09341 | CXCL1 | C-X-C motif chemokine ligand 1 |
|  | GSM1 | geniospasm 1 |
| P02724 | GYPA | glycophorin A (MNS blood group) |
| P06028 | GYPB | glycophorin B (MNS blood group) |
| P15421 | GYPE | glycophorin E (MNS blood group) |
| Q16775 | HAGH | hydroxyacylglutathione hydrolase |
| Q16836 | HADH | hydroxyacyl-CoA dehydrogenase |
| P12081 | HARS | histidyl-tRNA synthetase |
| P69892 | HBG2 | hemoglobin subunit gamma 2 |
| O43614 | HCRTR2 | hypocretin receptor 2 |
| Q13547 | HDAC1 | histone deacetylase 1 |
| P19113 | HDC | histidine decarboxylase |
| P14210 | HGF | hepatocyte growth factor |
| Q16665 | HIF1A | hypoxia inducible factor 1 subunit alpha |
| O00291 | HIP1 | huntingtin interacting protein 1 |
| P19367 | HK1 | hexokinase 1 |
| P01889;  P03989;  P10319;  P18463;  P18464;  P18465;  P30460;  P30461;  P30462;  P30464;  P30466;  P30475;  P30479;  P30480;  P30481;  P30483;  P30484;  P30485;  P30486;  P30487;  P30488;  P30490;  P30491;  P30492;  P30493;  P30495;  P30498;  P30685;  Q04826;  Q29718;  Q29836;  Q29940;  Q31610;  Q31612;  Q95365 | HLA-B | major histocompatibility complex, class I, B |
| P01903 | HLA-DRA | major histocompatibility complex, class II, DR alpha |
| P13762 | HLA-DRB4 | major histocompatibility complex, class II, DR beta 4 |
| P17693 | HLA-G | major histocompatibility complex, class I, G |
| P09429 | HMGB1 | high mobility group box 1 |
| P54868 | HMGCS2 | 3-hydroxy-3-methylglutaryl-CoA synthase 2 |
| P55317 | FOXA1 | forkhead box A1 |
| Q9Y261 | FOXA2 | forkhead box A2 |
| P41235 | HNF4A | hepatocyte nuclear factor 4 alpha |
| P22626 | HNRNPA2B1 | heterogeneous nuclear ribonucleoprotein A2/B1 |
| P84074 | HPCA | hippocalcin |
| P14061 | HSD17B1 | hydroxysteroid 17-beta dehydrogenase 1 |
| P51659 | HSD17B4 | hydroxysteroid 17-beta dehydrogenase 4 |
| P35475 | IDUA | iduronidase, alpha-L- |
| P40305 | IFI27 | interferon alpha inducible protein 27 |
| O14879 | IFIT3 | interferon induced protein with tetratricopeptide repeats 3 |
| P01562 | IFNA1 | interferon alpha 1 |
| P01562 | IFNA13 | interferon alpha 13 |
| P01574 | IFNB1 | interferon beta 1 |
| P08833 | IGFBP1 | insulin like growth factor binding protein 1 |
| P24593 | IGFBP5 | insulin like growth factor binding protein 5 |
| Q16270 | IGFBP7 | insulin like growth factor binding protein 7 |
|  | IGL | immunoglobulin lambda locus |
| P14778 | IL1R1 | interleukin 1 receptor type 1 |
| Q9NPH3 | IL1RAP | interleukin 1 receptor accessory protein |
| P29460 | IL12B | interleukin 12B |
| Q13418 | ILK | integrin linked kinase |
| P14902 | IDO1 | indoleamine 2,3-dioxygenase 1 |
| Q9UK53 | ING1 | inhibitor of growth family member 1 |
| O15357 | INPPL1 | inositol polyphosphate phosphatase like 1 |
| P48200 | IREB2 | iron responsive element binding protein 2 |
| O14896 | IRF6 | interferon regulatory factor 6 |
| Q92985 | IRF7 | interferon regulatory factor 7 |
| P06756 | ITGAV | integrin subunit alpha V |
| P05556 | ITGB1 | integrin subunit beta 1 |
| P05106 | ITGB3 | integrin subunit beta 3 |
| Q14643 | ITPR1 | inositol 1,4,5-trisphosphate receptor type 1 |
| Q14571 | ITPR2 | inositol 1,4,5-trisphosphate receptor type 2 |
| P05412 | JUN | Jun proto-oncogene, AP-1 transcription factor subunit |
| P17275 | JUNB | JunB proto-oncogene, AP-1 transcription factor subunit |
| P17535 | JUND | JunD proto-oncogene, AP-1 transcription factor subunit |
| Q14721 | KCNB1 | potassium voltage-gated channel subfamily B member 1 |
| Q03721 | KCNC4 | potassium voltage-gated channel subfamily C member 4 |
| Q12791 | KCNMA1 | potassium calcium-activated channel subfamily M alpha 1 |
| P51787 | KCNQ1 | potassium voltage-gated channel subfamily Q member 1 |
| P26715 | KLRC1 | killer cell lectin like receptor C1 |
| P52732 | KIF11 | kinesin family member 11 |
| P02533 | KRT14 | keratin 14 |
| P11047 | LAMC1 | laminin subunit gamma 1 |
| P13473 | LAMP2 | lysosomal associated membrane protein 2 |
| P18428 | LBP | lipopolysaccharide binding protein |
| P00338 | LDHA | lactate dehydrogenase A |
| P42702 | LIFR | LIF receptor alpha |
| P48059 | LIMS1 | LIM zinc finger domain containing 1 |
| P11150 | LIPC | lipase C, hepatic type |
| P02545 | LMNA | lamin A/C |
| P28300 | LOX | lysyl oxidase |
| Q08397 | LOXL1 | lysyl oxidase like 1 |
| P30533 | LRPAP1 | LDL receptor related protein associated protein 1 |
| P61626 | LYZ | lysozyme |
| P29966 | MARCKS | myristoylated alanine rich protein kinase C substrate |
| Q15796 | SMAD2 | SMAD family member 2 |
| P78559 | MAP1A | microtubule associated protein 1A |
| P46821 | MAP1B | microtubule associated protein 1B |
| Q00266 | MAT1A | methionine adenosyltransferase 1A |
| P31153 | MAT2A | methionine adenosyltransferase 2A |
| P56270 | MAZ | MYC associated zinc finger protein |
| P11226 | MBL2 | mannose binding lectin 2 |
| P21741 | MDK | midkine |
| Q00987 | MDM2 | MDM2 proto-oncogene |
| Q02078 | MEF2A | myocyte enhancer factor 2A |
| Q14814 | MEF2D | myocyte enhancer factor 2D |
| O15553 | MEFV | MEFV, pyrin innate immunity regulator |
| P08581 | MET | MET proto-oncogene, receptor tyrosine kinase |
| Q08431 | MFGE8 | milk fat globule-EGF factor 8 protein |
|  | MICE | MHC class I polypeptide-related sequence E (pseudogene) |
| P54252 | ATXN3 | ataxin 3 |
| P42568 | MLLT3 | MLLT3, super elongation complex subunit |
| P03956 | MMP1 | matrix metallopeptidase 1 |
| P50281 | MMP14 | matrix metallopeptidase 14 |
| Q16653 | MOG | myelin oligodendrocyte glycoprotein |
| P41217 | CD200 | CD200 molecule |
| P34949 | MPI | mannose phosphate isomerase |
| P25189 | MPZ | myelin protein zero |
| P49959 | MRE11 | MRE11 homolog, double strand break repair nuclease |
| P33527 | ABCC1 | ATP binding cassette subfamily C member 1 |
|  | MSD | microcephaly with spastic diplegia (Paine syndrome) |
| O43347 | MSI1 | musashi RNA binding protein 1 |
| Q9UJ68 | MSRA | methionine sulfoxide reductase A |
| P00414 | COX3 | cytochrome c oxidase III |
| P11586 | MTHFD1 | methylenetetrahydrofolate dehydrogenase, cyclohydrolase and formyltetrahydrofolate synthetase 1 |
| P03886 | ND1 | NADH dehydrogenase, subunit 1 (complex I) |
| P03905 | ND4 | NADH dehydrogenase, subunit 4 (complex I) |
| P48039 | MTNR1A | melatonin receptor 1A |
| Q99707 | MTR | 5-methyltetrahydrofolate-homocysteine methyltransferase |
| Q9UBK8 | MTRR | 5-methyltetrahydrofolate-homocysteine methyltransferase reductase |
| P22033 | MUT | methylmalonyl-CoA mutase |
| P20591 | MX1 | MX dynamin like GTPase 1 |
| P12524 | MYCL | MYCL proto-oncogene, bHLH transcription factor |
| Q99836 | MYD88 | myeloid differentiation primary response 88 |
| P13533 | MYH6 | myosin heavy chain 6 |
| Q9UM54 | MYO6 | myosin VI |
| E9PAV3;  Q13765 | NACA | nascent polypeptide associated complex subunit alpha |
| O15394 | NCAM2 | neural cell adhesion molecule 2 |
| Q16718 | NDUFA5 | NADH:ubiquinone oxidoreductase subunit A5 |
| P56556 | NDUFA6 | NADH:ubiquinone oxidoreductase subunit A6 |
| O95169 | NDUFB8 | NADH:ubiquinone oxidoreductase subunit B8 |
| Q15019 | SEPT2 | septin 2 |
| P07197 | NEFM | neurofilament medium |
| P12036 | NEFH | neurofilament heavy |
| Q99519 | NEU1 | neuraminidase 1 |
| Q9NZG7 | NINJ2 | ninjurin 2 |
| P15531 | NME1 | NME/NM23 nucleoside diphosphate kinase 1 |
| P22392 | NME2 | NME/NM23 nucleoside diphosphate kinase 2 |
| P16083 | NQO2 | N-ribosyldihydronicotinamide:quinone reductase 2 |
| P00491 | PNP | purine nucleoside phosphorylase |
| O15259 | NPHP1 | nephrocystin 1 |
| Q15818 | NPTX1 | neuronal pentraxin 1 |
| O43847 | NRDC | nardilysin convertase |
| Q92686 | NRGN | neurogranin |
| P67809 | YBX1 | Y-box binding protein 1 |
| P30990 | NTS | neurotensin |
| P52948 | NUP98 | nucleoporin 98 |
| P43354 | NR4A2 | nuclear receptor subfamily 4 group A member 2 |
| P47874 | OMP | olfactory marker protein |
| P13725 | OSM | oncostatin M |
| P00480 | OTC | ornithine carbamoyltransferase |
| Q15070 | OXA1L | OXA1L, mitochondrial inner membrane protein |
| P01178 | OXT | oxytocin/neurophysin I prepropeptide |
| P47900 | P2RY1 | purinergic receptor P2Y1 |
| P51582 | P2RY4 | pyrimidinergic receptor P2Y4 |
| Q15077 | P2RY6 | pyrimidinergic receptor P2Y6 |
| P30086 | PEBP1 | phosphatidylethanolamine binding protein 1 |
| P26367 | PAX6 | paired box 6 |
| P11498 | PC | pyruvate carboxylase |
| P48539 | PCP4 | Purkinje cell protein 4 |
| Q07002 | CDK18 | cyclin dependent kinase 18 |
| Q13946 | PDE7A | phosphodiesterase 7A |
| P01127 | PDGFB | platelet derived growth factor subunit B |
| P09619 | PDGFRB | platelet derived growth factor receptor beta |
| Q13822 | ENPP2 | ectonucleotide pyrophosphatase/phosphodiesterase 2 |
| P01213 | PDYN | prodynorphin |
| P01210 | PENK | proenkephalin |
| O15534 | PER1 | period circadian regulator 1 |
| Q16875 | PFKFB3 | 6-phosphofructo-2-kinase/fructose-2,6-biphosphatase 3 |
| P07093 | SERPINE2 | serpin family E member 2 |
| Q99574 | SERPINI1 | serpin family I member 1 |
| Q8NEB9 | PIK3C3 | phosphatidylinositol 3-kinase catalytic subunit type 3 |
| P00750 | PLAT | plasminogen activator, tissue type |
| Q03405 | PLAUR | plasminogen activator, urokinase receptor |
| Q15111 | PLCL1 | phospholipase C like 1 (inactive) |
| Q13393 | PLD1 | phospholipase D1 |
| P08567 | PLEK | pleckstrin |
| P53350 | PLK1 | polo like kinase 1 |
| P29590 | PML | promyelocytic leukemia |
| O15305 | PMM2 | phosphomannomutase 2 |
| P54821 | PRRX1 | paired related homeobox 1 |
| P54098 | POLG | DNA polymerase gamma, catalytic subunit |
| P49335 | POU3F4 | POU class 3 homeobox 4 |
| Q01860 | POU5F1 | POU class 5 homeobox 1 |
| Q03181 | PPARD | peroxisome proliferator activated receptor delta |
| P62937 | PPIA | peptidylprolyl isomerase A |
| P23284 | PPIB | peptidylprolyl isomerase B |
| P62136 | PPP1CA | protein phosphatase 1 catalytic subunit alpha |
| Q13522 | PPP1R1A | protein phosphatase 1 regulatory inhibitor subunit 1A |
| Q96QC0 | PPP1R10 | protein phosphatase 1 regulatory subunit 10 |
| P67775 | PPP2CA | protein phosphatase 2 catalytic subunit alpha |
| Q00005 | PPP2R2B | protein phosphatase 2 regulatory subunit Bbeta |
| Q08209 | PPP3CA | protein phosphatase 3 catalytic subunit alpha |
| P63098 | PPP3R1 | protein phosphatase 3 regulatory subunit B, alpha |
| P50897 | PPT1 | palmitoyl-protein thioesterase 1 |
| P04280 | PRB1 | proline rich protein BstNI subfamily 1 |
| P17612 | PRKACA | protein kinase cAMP-activated catalytic subunit alpha |
| P22694 | PRKACB | protein kinase cAMP-activated catalytic subunit beta |
| P31321 | PRKAR1B | protein kinase cAMP-dependent type I regulatory subunit beta |
| P05771 | PRKCB | protein kinase C beta |
| Q02156 | PRKCE | protein kinase C epsilon |
| P01236 | PRL | prolactin |
|  | PROS2P | protein S (beta) pseudogene |
| P35030 | PRSS3 | serine protease 3 |
| P48740 | MASP1 | mannan binding lectin serine peptidase 1 |
| A5PKW4 | PSD | pleckstrin and Sec7 domain containing |
| P28072 | PSMB6 | proteasome subunit beta 6 |
| P28065 | PSMB9 | proteasome subunit beta 9 |
| Q13200 | PSMD2 | proteasome 26S subunit, non-ATPase 2 |
| O00233 | PSMD9 | proteasome 26S subunit, non-ATPase 9 |
| Q13635 | PTCH1 | patched 1 |
| P60484 | PTEN | phosphatase and tensin homolog |
| Q13258 | PTGDR | prostaglandin D2 receptor |
| P34995 | PTGER1 | prostaglandin E receptor 1 |
| P43115 | PTGER3 | prostaglandin E receptor 3 |
| P35408 | PTGER4 | prostaglandin E receptor 4 |
| P18031 | PTPN1 | protein tyrosine phosphatase, non-receptor type 1 |
| Q00577 | PURA | purine rich element binding protein A |
| P20472 | PVALB | parvalbumin |
| P20742 | PZP | PZP, alpha-2-macroglobulin like |
| P20340 | RAB6A | RAB6A, member RAS oncogene family |
| P54727 | RAD23B | RAD23 homolog B, nucleotide excision repair protein |
| P04049 | RAF1 | Raf-1 proto-oncogene, serine/threonine kinase |
| P62826 | RAN | RAN, member RAS oncogene family |
| P06400 | RB1 | RB transcriptional corepressor 1 |
| Q7Z6E9 | RBBP6 | RB binding protein 6, ubiquitin ligase |
| P02753 | RBP4 | retinol binding protein 4 |
| P05451 | REG1A | regenerating family member 1 alpha |
| Q04206 | RELA | RELA proto-oncogene, NF-kB subunit |
| P07949 | RET | ret proto-oncogene |
| P35251 | RFC1 | replication factor C subunit 1 |
| P41220 | RGS2 | regulator of G protein signaling 2 |
| P49798 | RGS4 | regulator of G protein signaling 4 |
| Q02161 | RHD | Rh blood group D antigen |
| Q13464 | ROCK1 | Rho associated coiled-coil containing protein kinase 1 |
| P26373 | RPL13 | ribosomal protein L13 |
| P61247 | RPS3A | ribosomal protein S3A |
| Q9UBS0 | RPS6KB2 | ribosomal protein S6 kinase B2 |
| P62266 | RPS23 | ribosomal protein S23 |
| P42677 | RPS27 | ribosomal protein S27 |
| P10301 | RRAS | RAS related |
| Q92736 | RYR2 | ryanodine receptor 2 |
| P06703 | S100A6 | S100 calcium binding protein A6 |
| P05109 | S100A8 | S100 calcium binding protein A8 |
| P80511 | S100A12 | S100 calcium binding protein A12 |
| P54253 | ATXN1 | ataxin 1 |
| Q99700 | ATXN2 | ataxin 2 |
| P22307 | SCP2 | sterol carrier protein 2 |
| P80075 | CCL8 | C-C motif chemokine ligand 8 |
| Q99731 | CCL19 | C-C motif chemokine ligand 19 |
| O00585 | CCL21 | C-C motif chemokine ligand 21 |
| P34741 | SDC2 | syndecan 2 |
| Q9UBV2 | SEL1L | SEL1L, ERAD E3 ligase adaptor subunit |
| P45985 | MAP2K4 | mitogen-activated protein kinase kinase 4 |
| P23246 | SFPQ | splicing factor proline and glutamine rich |
| P84103 | SRSF3 | serine and arginine rich splicing factor 3 |
| Q13243 | SRSF5 | serine and arginine rich splicing factor 5 |
| P62995 | TRA2B | transformer 2 beta homolog |
| Q16586 | SGCA | sarcoglycan alpha |
| P51688 | SGSH | N-sulfoglucosamine sulfohydrolase |
| Q15811 | ITSN1 | intersectin 1 |
| Q99962 | SH3GL2 | SH3 domain containing GRB2 like 2, endophilin A1 |
| Q14190 | SIM2 | SIM bHLH transcription factor 2 |
| P12757 | SKIL | SKI like proto-oncogene |
| P43005 | SLC1A1 | solute carrier family 1 member 1 |
| P43003 | SLC1A3 | solute carrier family 1 member 3 |
| P11169 | SLC2A3 | solute carrier family 2 member 3 |
| P32418 | SLC8A1 | solute carrier family 8 member A1 |
| P57103 | SLC8A3 | solute carrier family 8 member A3 |
| P54219 | SLC18A1 | solute carrier family 18 member A1 |
| Q05940 | SLC18A2 | solute carrier family 18 member A2 |
| O75094 | SLIT3 | slit guidance ligand 3 |
| P17405 | SMPD1 | sphingomyelin phosphodiesterase 1 |
| O60906 | SMPD2 | sphingomyelin phosphodiesterase 2 |
| Q07890 | SOS2 | SOS Ras/Rho guanine nucleotide exchange factor 2 |
| P48431 | SOX2 | SRY-box 2 |
| Q02446 | SP4 | Sp4 transcription factor |
| Q9UBP0 | SPAST | spastin |
| Q01082 | SPTBN1 | spectrin beta, non-erythrocytic 1 |
| Q12772 | SREBF2 | sterol regulatory element binding transcription factor 2 |
| P11831 | SRF | serum response factor |
| P30874 | SSTR2 | somatostatin receptor 2 |
|  | ST2 | suppression of tumorigenicity 2 |
| P42224 | STAT1 | signal transducer and activator of transcription 1 |
| P52823 | STC1 | stanniocalcin 1 |
| P61764 | STXBP1 | syntaxin binding protein 1 |
| Q06520 | SULT2A1 | sulfotransferase family 2A member 1 |
| P23763 | VAMP1 | vesicle associated membrane protein 1 |
| Q92777 | SYN2 | synapsin II |
| P21579 | SYT1 | synaptotagmin 1 |
| P21452 | TACR2 | tachykinin receptor 2 |
| P29371 | TACR3 | tachykinin receptor 3 |
|  | TRBV20OR9-2 | T cell receptor beta variable 20/OR9-2 (non-functional) |
| P48775 | TDO2 | tryptophan 2,3-dioxygenase |
| O14746 | TERT | telomerase reverse transcriptase |
| Q14186 | TFDP1 | transcription factor Dp-1 |
| Q07654 | TFF3 | trefoil factor 3 |
| P02786 | TFRC | transferrin receptor |
| P61812 | TGFB2 | transforming growth factor beta 2 |
|  | THAS | thoracoabdominal syndrome |
| P35443 | THBS4 | thrombospondin 4 |
| P52888 | THOP1 | thimet oligopeptidase 1 |
| P10827 | THRA | thyroid hormone receptor alpha |
| P31483 | TIA1 | TIA1 cytotoxic granule associated RNA binding protein |
| P35625 | TIMP3 | TIMP metallopeptidase inhibitor 3 |
| Q99727 | TIMP4 | TIMP metallopeptidase inhibitor 4 |
| Q04724 | TLE1 | transducin like enhancer of split 1 |
| P41732 | TSPAN7 | tetraspanin 7 |
| O00501 | CLDN5 | claudin 5 |
| Q13829 | TNFAIP1 | TNF alpha induced protein 1 |
| P19429 | TNNI3 | troponin I3, cardiac type |
| Q13625 | TP53BP2 | tumor protein p53 binding protein 2 |
| P17752 | TPH1 | tryptophan hydroxylase 1 |
| P13693 | TPT1 | tumor protein, translationally-controlled 1 |
| Q12933 | TRAF2 | TNF receptor associated factor 2 |
| Q9Y210 | TRPC6 | transient receptor potential cation channel subfamily C member 6 |
| O94759 | TRPM2 | transient receptor potential cation channel subfamily M member 2 |
| P16473 | TSHR | thyroid stimulating hormone receptor |
| P0CV98;  P0CW01;  Q01534 | TSPY1 | testis specific protein Y-linked 1 |
| P33981 | TTK | TTK protein kinase |
| Q15672 | TWIST1 | twist family bHLH transcription factor 1 |
| P29597 | TYK2 | tyrosine kinase 2 |
| Q06418 | TYRO3 | TYRO3 protein tyrosine kinase |
| P17643 | TYRP1 | tyrosinase related protein 1 |
| Q05086 | UBE3A | ubiquitin protein ligase E3A |
| P78381 | SLC35A2 | solute carrier family 35 member A2 |
|  | UGT1A | UDP glucuronosyltransferase family 1 member A complex locus |
| P13051 | UNG | uracil DNA glycosylase |
| P31930 | UQCRC1 | ubiquinol-cytochrome c reductase core protein 1 |
| Q15853 | USF2 | upstream transcription factor 2, c-fos interacting |
| P01282 | VIP | vasoactive intestinal peptide |
| O76090 | BEST1 | bestrophin 1 |
| P04275 | VWF | von Willebrand factor |
| P23381 | WARS | tryptophanyl-tRNA synthetase |
| P42768 | WAS | Wiskott-Aldrich syndrome |
| P30291 | WEE1 | WEE1 G2 checkpoint kinase |
| P04628 | WNT1 | Wnt family member 1 |
| P41221 | WNT5A | Wnt family member 5A |
| Q93097 | WNT2B | Wnt family member 2B |
| P19544 | WT1 | Wilms tumor 1 |
|  | XBP1P1 | X-box binding protein 1 pseudogene 1 |
| P18887 | XRCC1 | X-ray repair cross complementing 1 |
| P28698 | MZF1 | myeloid zinc finger 1 |
| Q9ULX5 | RNF112 | ring finger protein 112 |
| Q9NZL3 | ZNF224 | zinc finger protein 224 |
| Q9UL36 | ZNF236 | zinc finger protein 236 |
| Q12852 | MAP3K12 | mitogen-activated protein kinase kinase kinase 12 |
| Q14114 | LRP8 | LDL receptor related protein 8 |
| Q13467 | FZD5 | frizzled class receptor 5 |
| P51149 | RAB7A | RAB7A, member RAS oncogene family |
| Q00765 | REEP5 | receptor accessory protein 5 |
| Q13838 | DDX39B | DExD-box helicase 39B |
| P19484 | TFEB | transcription factor EB |
| P48307 | TFPI2 | tissue factor pathway inhibitor 2 |
| Q12774 | ARHGEF5 | Rho guanine nucleotide exchange factor 5 |
| O43653 | PSCA | prostate stem cell antigen |
| P35658 | NUP214 | nucleoporin 214 |
|  | AD5 | Alzheimer disease 5 |
| Q9NRG9 | AAAS | aladin WD repeat nucleoporin |
| Q86U42 | PABPN1 | poly(A) binding protein nuclear 1 |
| P39687 | ANP32A | acidic nuclear phosphoprotein 32 family member A |
|  | CDR3 | Cerebellar degeneration-related autoantigen-3 |
| Q15849 | SLC14A2 | solute carrier family 14 member 2 |
| Q13112 | CHAF1B | chromatin assembly factor 1 subunit B |
| Q93008 | USP9X | ubiquitin specific peptidase 9 X-linked |
| O75923 | DYSF | dysferlin |
| Q92560 | BAP1 | BRCA1 associated protein 1 |
| O95936 | EOMES | eomesodermin |
| O60231 | DHX16 | DEAH-box helicase 16 |
| O75344 | FKBP6 | FK506 binding protein 6 |
| O14654 | IRS4 | insulin receptor substrate 4 |
| Q99569 | PKP4 | plakophilin 4 |
| Q92569 | PIK3R3 | phosphoinositide-3-kinase regulatory subunit 3 |
| P61968 | LMO4 | LIM domain only 4 |
| Q8IW41 | MAPKAPK5 | mitogen-activated protein kinase-activated protein kinase 5 |
| O75569 | PRKRA | protein activator of interferon induced protein kinase EIF2AK2 |
| O75716 | STK16 | serine/threonine kinase 16 |
| O60763 | USO1 | USO1 vesicle transport factor |
| O95263 | PDE8B | phosphodiesterase 8B |
| Q16853 | AOC3 | amine oxidase, copper containing 3 |
| P49757 | NUMB | NUMB, endocytic adaptor protein |
| O15524 | SOCS1 | suppressor of cytokine signaling 1 |
| Q9Y4H2 | IRS2 | insulin receptor substrate 2 |
| Q13470 | TNK1 | tyrosine kinase non receptor 1 |
| Q13546 | RIPK1 | receptor interacting serine/threonine kinase 1 |
| Q13443 | ADAM9 | ADAM metallopeptidase domain 9 |
| P62491 | RAB11A | RAB11A, member RAS oncogene family |
| P17152 | TMEM11 | transmembrane protein 11 |
| O14508 | SOCS2 | suppressor of cytokine signaling 2 |
| O15379 | HDAC3 | histone deacetylase 3 |
| O43490 | PROM1 | prominin 1 |
| P49019 | HCAR3 | hydroxycarboxylic acid receptor 3 |
| O94788 | ALDH1A2 | aldehyde dehydrogenase 1 family member A2 |
| P56645 | PER3 | period circadian regulator 3 |
| O15055 | PER2 | period circadian regulator 2 |
| Q14155 | ARHGEF7 | Rho guanine nucleotide exchange factor 7 |
| Q13564 | NAE1 | NEDD8 activating enzyme E1 subunit 1 |
| P20042 | EIF2S2 | eukaryotic translation initiation factor 2 subunit beta |
| Q92558 | WASF1 | WAS protein family member 1 |
| Q13319 | CDK5R2 | cyclin dependent kinase 5 regulatory subunit 2 |
| Q12988 | HSPB3 | heat shock protein family B (small) member 3 |
| O60229 | KALRN | kalirin RhoGEF kinase |
| O14543 | SOCS3 | suppressor of cytokine signaling 3 |
| Q8IWQ3 | BRSK2 | BR serine/threonine kinase 2 |
| Q9UHE5 | NAT8 | N-acetyltransferase 8 (putative) |
| O60271 | SPAG9 | sperm associated antigen 9 |
| Q5T4W7 | ARTN | artemin |
| O95832 | CLDN1 | claudin 1 |
| O43439 | CBFA2T2 | CBFA2/RUNX1 translocation partner 2 |
| O14964 | HGS | hepatocyte growth factor-regulated tyrosine kinase substrate |
| Q01638 | IL1RL1 | interleukin 1 receptor like 1 |
| O00400 | SLC33A1 | solute carrier family 33 member 1 |
| Q14202 | ZMYM3 | zinc finger MYM-type containing 3 |
| O95411 | TIAF1 | TGFB1-induced anti-apoptotic factor 1 |
| Q15907 | RAB11B | RAB11B, member RAS oncogene family |
| Q13253 | NOG | noggin |
| P49137 | MAPKAPK2 | mitogen-activated protein kinase-activated protein kinase 2 |
| O14544 | SOCS6 | suppressor of cytokine signaling 6 |
| Q01151 | CD83 | CD83 molecule |
| Q9UL54 | TAOK2 | TAO kinase 2 |
| O94813 | SLIT2 | slit guidance ligand 2 |
| O75881 | CYP7B1 | cytochrome P450 family 7 subfamily B member 1 |
| O95631 | NTN1 | netrin 1 |
| Q9NSB8 | HOMER2 | homer scaffold protein 2 |
| Q86YM7 | HOMER1 | homer scaffold protein 1 |
| Q6UWB1 | IL27RA | interleukin 27 receptor subunit alpha |
| O60239 | SH3BP5 | SH3 domain binding protein 5 |
| O75116 | ROCK2 | Rho associated coiled-coil containing protein kinase 2 |
| O95847 | SLC25A27 | solute carrier family 25 member 27 |
| Q9UNK0 | STX8 | syntaxin 8 |
| O95450 | ADAMTS2 | ADAM metallopeptidase with thrombospondin type 1 motif 2 |
| Q99999 | GAL3ST1 | galactose-3-O-sulfotransferase 1 |
| Q99988 | GDF15 | growth differentiation factor 15 |
| P55786 | NPEPPS | aminopeptidase puromycin sensitive |
| O95817 | BAG3 | BCL2 associated athanogene 3 |
| Q16568 | CARTPT | CART prepropeptide |
| O95988 | TCL1B | T cell leukemia/lymphoma 1B |
| Q9Y6H5 | SNCAIP | synuclein alpha interacting protein |
|  | ECE2 | endothelin converting enzyme 2 |
| Q9UKV0 | HDAC9 | histone deacetylase 9 |
| Q15027 | ACAP1 | ArfGAP with coiled-coil, ankyrin repeat and PH domains 1 |
| Q14145 | KEAP1 | kelch like ECH associated protein 1 |
| Q7L014 | DDX46 | DEAD-box helicase 46 |
| O60641 | SNAP91 | synaptosome associated protein 91 |
| Q7L0J3 | SV2A | synaptic vesicle glycoprotein 2A |
| Q15021 | NCAPD2 | non-SMC condensin I complex subunit D2 |
| O14792 | HS3ST1 | heparan sulfate-glucosamine 3-sulfotransferase 1 |
| Q93074 | MED12 | mediator complex subunit 12 |
| Q9Y6H6 | KCNE3 | potassium voltage-gated channel subfamily E regulatory subunit 3 |
| Q92844 | TANK | TRAF family member associated NFKB activator |
| Q9HD15 | SRA1 | steroid receptor RNA activator 1 |
| O43521 | BCL2L11 | BCL2 like 11 |
| Q9UQQ2 | SH2B3 | SH2B adaptor protein 3 |
| O95998 | IL18BP | interleukin 18 binding protein |
| Q9Y5P4 | COL4A3BP | collagen type IV alpha 3 binding protein |
| P30405 | PPIF | peptidylprolyl isomerase F |
| O14595 | CTDSP2 | CTD small phosphatase 2 |
| Q99547 | MPHOSPH6 | M-phase phosphoprotein 6 |
| O00148 | DDX39A | DExD-box helicase 39A |
| O95390 | GDF11 | growth differentiation factor 11 |
| Q13137 | CALCOCO2 | calcium binding and coiled-coil domain 2 |
| Q9Y691 | KCNMB2 | potassium calcium-activated channel subfamily M regulatory beta subunit 2 |
| Q9NQX3 | GPHN | gephyrin |
| O15439 | ABCC4 | ATP binding cassette subfamily C member 4 |
| O60895 | RAMP2 | receptor activity modifying protein 2 |
| Q9UNE7 | STUB1 | STIP1 homology and U-box containing protein 1 |
| Q8N423 | LILRB2 | leukocyte immunoglobulin like receptor B2 |
| O95996 | APC2 | APC2, WNT signaling pathway regulator |
| Q9Y6N6 | LAMC3 | laminin subunit gamma 3 |
| Q9Y258 | CCL26 | C-C motif chemokine ligand 26 |
| Q9UGM6 | WARS2 | tryptophanyl tRNA synthetase 2, mitochondrial |
| Q00978 | IRF9 | interferon regulatory factor 9 |
| Q9BX26 | SYCP2 | synaptonemal complex protein 2 |
| P63244 | RACK1 | receptor for activated C kinase 1 |
| Q9Y646 | CPQ | carboxypeptidase Q |
| O95398 | RAPGEF3 | Rap guanine nucleotide exchange factor 3 |
| O14744 | PRMT5 | protein arginine methyltransferase 5 |
| Q9UEE9 | CFDP1 | craniofacial development protein 1 |
| Q9UP83 | COG5 | component of oligomeric golgi complex 5 |
| O75947 | ATP5PD | ATP synthase peripheral stalk subunit d |
| Q92581 | SLC9A6 | solute carrier family 9 member A6 |
| Q9UBU9 | NXF1 | nuclear RNA export factor 1 |
| Q5JRX3 | PITRM1 | pitrilysin metallopeptidase 1 |
| O60232 | SSSCA1 | Sjogren syndrome/scleroderma autoantigen 1 |
| P78382 | SLC35A1 | solute carrier family 35 member A1 |
| P61916 | NPC2 | NPC intracellular cholesterol transporter 2 |
| P52954 | LBX1 | ladybird homeobox 1 |
| O00399 | DCTN6 | dynactin subunit 6 |
| Q15125 | EBP | EBP, cholestenol delta-isomerase |
| Q53GS9 | USP39 | ubiquitin specific peptidase 39 |
| P56975 | NRG3 | neuregulin 3 |
| Q15185 | PTGES3 | prostaglandin E synthase 3 |
| O00533 | CHL1 | cell adhesion molecule L1 like |
| P56211 | ARPP19 | cAMP regulated phosphoprotein 19 |
| Q92598 | HSPH1 | heat shock protein family H (Hsp110) member 1 |
| Q6PUV4 | CPLX2 | complexin 2 |
| O14810 | CPLX1 | complexin 1 |
| Q9Y251 | HPSE | heparanase |
| O00264 | PGRMC1 | progesterone receptor membrane component 1 |
| O75973 | C1QL1 | complement C1q like 1 |
| Q9Y5R2 | MMP24 | matrix metallopeptidase 24 |
| P30048 | PRDX3 | peroxiredoxin 3 |
| Q9Y6M0 | PRSS21 | serine protease 21 |
| P31948 | STIP1 | stress induced phosphoprotein 1 |
| P49755 | TMED10 | transmembrane p24 trafficking protein 10 |
| P50579 | METAP2 | methionyl aminopeptidase 2 |
| P60981 | DSTN | destrin, actin depolymerizing factor |
| Q93045 | STMN2 | stathmin 2 |
| Q9UNN5 | FAF1 | Fas associated factor 1 |
| P82251 | SLC7A9 | solute carrier family 7 member 9 |
| O95759 | TBC1D8 | TBC1 domain family member 8 |
| Q9Y484 | WDR45 | WD repeat domain 45 |
| O75717 | WDHD1 | WD repeat and HMG-box DNA binding protein 1 |
| Q9Y5N1 | HRH3 | histamine receptor H3 |
| Q99623 | PHB2 | prohibitin 2 |
| Q99685 | MGLL | monoglyceride lipase |
| Q8N2Q7 | NLGN1 | neuroligin 1 |
| O94989 | ARHGEF15 | Rho guanine nucleotide exchange factor 15 |
| Q9Y2G2 | CARD8 | caspase recruitment domain family member 8 |
| Q9Y2G9 | SBNO2 | strawberry notch homolog 2 |
| Q9UPU3 | SORCS3 | sortilin related VPS10 domain containing receptor 3 |
| Q14686 | NCOA6 | nuclear receptor coactivator 6 |
| Q9BZQ4 | NMNAT2 | nicotinamide nucleotide adenylyltransferase 2 |
| Q6IC98 | GRAMD4 | GRAM domain containing 4 |
| Q9UKL0 | RCOR1 | REST corepressor 1 |
| Q92575 | UBXN4 | UBX domain protein 4 |
| O75129 | ASTN2 | astrotactin 2 |
| O75153 | CLUH | clustered mitochondria homolog |
| Q12769 | NUP160 | nucleoporin 160 |
| Q2M389 | WASHC4 | WASH complex subunit 4 |
| Q8NF91 | SYNE1 | spectrin repeat containing nuclear envelope protein 1 |
| Q5T4S7 | UBR4 | ubiquitin protein ligase E3 component n-recognin 4 |
| Q9NXA8 | SIRT5 | sirtuin 5 |
| Q9Y3R0 | GRIP1 | glutamate receptor interacting protein 1 |
| O95502 | NPTXR | neuronal pentraxin receptor |
| Q14681 | KCTD2 | potassium channel tetramerization domain containing 2 |
| Q9UBD9 | CLCF1 | cardiotrophin like cytokine factor 1 |
| O76054 | SEC14L2 | SEC14 like lipid binding 2 |
| Q9BZE4 | GTPBP4 | GTP binding protein 4 |
| Q9UM07 | PADI4 | peptidyl arginine deiminase 4 |
| O94760 | DDAH1 | dimethylarginine dimethylaminohydrolase 1 |
| O95665 | NTSR2 | neurotensin receptor 2 |
| Q9UKY0 | PRND | prion like protein doppel |
| O75807 | PPP1R15A | protein phosphatase 1 regulatory subunit 15A |
| Q9UPY5 | SLC7A11 | solute carrier family 7 member 11 |
| Q9UIK5 | TMEFF2 | transmembrane protein with EGF like and two follistatin like domains 2 |
| P57729 | RAB38 | RAB38, member RAS oncogene family |
| Q9Y6B2 | EID1 | EP300 interacting inhibitor of differentiation 1 |
| O94906 | PRPF6 | pre-mRNA processing factor 6 |
| Q9Y3I1 | FBXO7 | F-box protein 7 |
| O95415 | BRI3 | brain protein I3 |
| Q9UKC9 | FBXL2 | F-box and leucine rich repeat protein 2 |
| Q9UQN3 | CHMP2B | charged multivesicular body protein 2B |
| Q7Z4F1 | LRP10 | LDL receptor related protein 10 |
| Q8WWY3 | PRPF31 | pre-mRNA processing factor 31 |
| Q52LR7 | EPC2 | enhancer of polycomb homolog 2 |
| Q9UK22 | FBXO2 | F-box protein 2 |
| P56750 | CLDN17 | claudin 17 |
| Q9UJY1 | HSPB8 | heat shock protein family B (small) member 8 |
| Q6UXD5 | SEZ6L2 | seizure related 6 homolog like 2 |
| Q9H190 | SDCBP2 | syndecan binding protein 2 |
| Q02083 | NAAA | N-acylethanolamine acid amidase |
| Q9Y3P8 | SIT1 | signaling threshold regulating transmembrane adaptor 1 |
| O75509 | TNFRSF21 | TNF receptor superfamily member 21 |
| Q53EL6 | PDCD4 | programmed cell death 4 |
| Q9UQ52 | CNTN6 | contactin 6 |
| Q92730 | RND1 | Rho family GTPase 1 |
| Q16763 | UBE2S | ubiquitin conjugating enzyme E2 S |
| Q3YBM2 | TMEM176B | transmembrane protein 176B |
| Q8WXD2 | SCG3 | secretogranin III |
| Q6IBW4 | NCAPH2 | non-SMC condensin II complex subunit H2 |
| Q96ED9 | HOOK2 | hook microtubule tethering protein 2 |
| Q9UMY4 | SNX12 | sorting nexin 12 |
| Q9NY64 | SLC2A8 | solute carrier family 2 member 8 |
| Q9NZM5 | NOP53 | NOP53 ribosome biogenesis factor |
| Q9NP59 | SLC40A1 | solute carrier family 40 member 1 |
| O14512 | SOCS7 | suppressor of cytokine signaling 7 |
| Q9GZX6 | IL22 | interleukin 22 |
| P57678 | GEMIN4 | gem nuclear organelle associated protein 4 |
|  | MBL3P | mannose-binding lectin family member 3, pseudogene |
|  | PCA3 | prostate cancer associated 3 |
| Q9Y566 | SHANK1 | SH3 and multiple ankyrin repeat domains 1 |
| Q8N9N2 | ASCC1 | activating signal cointegrator 1 complex subunit 1 |
| Q86XR7;  Q9Y3B3 | TMED7 | transmembrane p24 trafficking protein 7 |
| Q9Y3D6 | FIS1 | fission, mitochondrial 1 |
| Q9Y2S2 | CRYL1 | crystallin lambda 1 |
| Q9Y3A2 | UTP11 | UTP11, small subunit processome component |
| Q9NWZ3 | IRAK4 | interleukin 1 receptor associated kinase 4 |
| Q8WU39 | MZB1 | marginal zone B and B1 cell specific protein |
| Q9NPF0 | CD320 | CD320 molecule |
| Q9BQ95 | ECSIT | ECSIT signalling integrator |
| Q9NPD7 | NRN1 | neuritin 1 |
| Q9UJC3 | HOOK1 | hook microtubule tethering protein 1 |
| O95071 | UBR5 | ubiquitin protein ligase E3 component n-recognin 5 |
| Q9UBW5 | BIN2 | bridging integrator 2 |
| Q9UIC8 | LCMT1 | leucine carboxyl methyltransferase 1 |
| Q9UHF3 | NAT8B | N-acetyltransferase 8B (putative, gene/pseudogene) |
| Q9NZC3 | GDE1 | glycerophosphodiester phosphodiesterase 1 |
| Q9UBU3 | GHRL | ghrelin and obestatin prepropeptide |
| Q8TAD2 | IL17D | interleukin 17D |
| Q9NZR2 | LRP1B | LDL receptor related protein 1B |
| Q9H0Q3 | FXYD6 | FXYD domain containing ion transport regulator 6 |
| O94779 | CNTN5 | contactin 5 |
| Q9NRF9 | POLE3 | DNA polymerase epsilon 3, accessory subunit |
| Q9NP99 | TREM1 | triggering receptor expressed on myeloid cells 1 |
| Q9P0J1 | PDP1 | pyruvate dehyrogenase phosphatase catalytic subunit 1 |
| Q86V15 | CASZ1 | castor zinc finger 1 |
| Q5VW36 | FOCAD | focadhesin |
| Q96DW6 | SLC25A38 | solute carrier family 25 member 38 |
| Q9BX59 | TAPBPL | TAP binding protein like |
| Q96G61 | NUDT11 | nudix hydrolase 11 |
| A3KN83 | SBNO1 | strawberry notch homolog 1 |
| Q9BV57 | ADI1 | acireductone dioxygenase 1 |
| Q969H0 | FBXW7 | F-box and WD repeat domain containing 7 |
| Q6XR72 | SLC30A10 | solute carrier family 30 member 10 |
| P51805 | PLXNA3 | plexin A3 |
| Q6VMQ6 | ATF7IP | activating transcription factor 7 interacting protein |
| Q5VTY9 | HHAT | hedgehog acyltransferase |
| Q8TAP4 | LMO3 | LIM domain only 3 |
| Q9NQX4 | MYO5C | myosin VC |
| O95445 | APOM | apolipoprotein M |
| P0CG37 | CFC1 | cripto, FRL-1, cryptic family 1 |
| Q5T5P2 | KIAA1217 | KIAA1217 |
| Q9UIR0 | BTNL2 | butyrophilin like 2 |
| Q8WYA6 | CTNNBL1 | catenin beta like 1 |
| Q9NRM0 | SLC2A9 | solute carrier family 2 member 9 |
| Q7Z6G8 | ANKS1B | ankyrin repeat and sterile alpha motif domain containing 1B |
| Q8TCT7 | SPPL2B | signal peptide peptidase like 2B |
| Q9NRW4 | DUSP22 | dual specificity phosphatase 22 |
| Q86YH6 | PDSS2 | decaprenyl diphosphate synthase subunit 2 |
| Q9HDD0 | HRASLS | HRAS like suppressor |
| Q8IU85 | CAMK1D | calcium/calmodulin dependent protein kinase ID |
| Q8N6Q3 | CD177 | CD177 molecule |
| Q9NQC3 | RTN4 | reticulon 4 |
| Q9GZX7 | AICDA | activation induced cytidine deaminase |
| Q86WK6 | AMIGO1 | adhesion molecule with Ig like domain 1 |
| Q9ULH0 | KIDINS220 | kinase D interacting substrate 220 |
| Q9P2P5 | HECW2 | HECT, C2 and WW domain containing E3 ubiquitin protein ligase 2 |
| Q96PQ0 | SORCS2 | sortilin related VPS10 domain containing receptor 2 |
| Q9P246 | STIM2 | stromal interaction molecule 2 |
| Q96Q42 | ALS2 | ALS2, alsin Rho guanine nucleotide exchange factor |
| Q9Y333 | LSM2 | LSM2 homolog, U6 small nuclear RNA and mRNA degradation associated |
| Q8TE85 | GRHL3 | grainyhead like transcription factor 3 |
| Q9UKA9 | PTBP2 | polypyrimidine tract binding protein 2 |
| Q9NPP4 | NLRC4 | NLR family CARD domain containing 4 |
| P57087 | JAM2 | junctional adhesion molecule 2 |
| Q9HB63 | NTN4 | netrin 4 |
| Q9HBA0 | TRPV4 | transient receptor potential cation channel subfamily V member 4 |
| Q9GZT4 | SRR | serine racemase |
| Q96GW7 | BCAN | brevican |
| Q96NK8 | NEUROD6 | neuronal differentiation 6 |
| Q9GZZ7 | GFRA4 | GDNF family receptor alpha 4 |
| Q9H2S6 | TNMD | tenomodulin |
| Q96BY2 | MOAP1 | modulator of apoptosis 1 |
| Q8WTT2 | NOC3L | NOC3 like DNA replication regulator |
| Q9H173 | SIL1 | SIL1 nucleotide exchange factor |
| Q9H772 | GREM2 | gremlin 2, DAN family BMP antagonist |
| Q9BQQ3 | GORASP1 | golgi reassembly stacking protein 1 |
|  | AD6 | Alzheimer disease 6 |
| Q6ZS30 | NBEAL1 | neurobeachin like 1 |
| Q96C19 | EFHD2 | EF-hand domain family member D2 |
| Q9H9Z2 | LIN28A | lin-28 homolog A |
| Q96DC8 | ECHDC3 | enoyl-CoA hydratase domain containing 3 |
| Q8WV60 | PTCD2 | pentatricopeptide repeat domain 2 |
| Q6PJT7 | ZC3H14 | zinc finger CCCH-type containing 14 |
| Q8TB24 | RIN3 | Ras and Rab interactor 3 |
| Q9H9S0 | NANOG | Nanog homeobox |
| Q86SK9 | SCD5 | stearoyl-CoA desaturase 5 |
| Q9BZ23 | PANK2 | pantothenate kinase 2 |
| Q9H7Z7 | PTGES2 | prostaglandin E synthase 2 |
| Q8NFU7 | TET1 | tet methylcytosine dioxygenase 1 |
| Q9H3Z4 | DNAJC5 | DnaJ heat shock protein family (Hsp40) member C5 |
| Q16609 | LPAL2 | lipoprotein(a) like 2, pseudogene |
| Q9BZV2 | SLC19A3 | solute carrier family 19 member 3 |
| Q9GZQ8 | MAP1LC3B | microtubule associated protein 1 light chain 3 beta |
| Q8TDF5 | NETO1 | neuropilin and tolloid like 1 |
| Q9NTX7 | RNF146 | ring finger protein 146 |
| Q96P65 | QRFPR | pyroglutamylated RFamide peptide receptor |
| Q86VS8 | HOOK3 | hook microtubule tethering protein 3 |
| Q8TDC3 | BRSK1 | BR serine/threonine kinase 1 |
| Q9BXY0 | MAK16 | MAK16 homolog |
| Q9H492 | MAP1LC3A | microtubule associated protein 1 light chain 3 alpha |
| Q9BXI3 | NT5C1A | 5'-nucleotidase, cytosolic IA |
| Q96SB3 | PPP1R9B | protein phosphatase 1 regulatory subunit 9B |
| Q8TDB4 | MGARP | mitochondria localized glutamic acid rich protein |
| P54829 | PTPN5 | protein tyrosine phosphatase, non-receptor type 5 |
| Q96DT6 | ATG4C | autophagy related 4C cysteine peptidase |
| Q9BYB0 | SHANK3 | SH3 and multiple ankyrin repeat domains 3 |
| P56704 | WNT3A | Wnt family member 3A |
| Q8IVL1 | NAV2 | neuron navigator 2 |
| Q969F0 | FATE1 | fetal and adult testis expressed 1 |
| Q5EBL2 | ZNF628 | zinc finger protein 628 |
| Q8TAE8 | GADD45GIP1 | GADD45G interacting protein 1 |
| Q9BU23 | LMF2 | lipase maturation factor 2 |
| Q5M775 | SPECC1 | sperm antigen with calponin homology and coiled-coil domains 1 |
| Q8IZQ8 | MYOCD | myocardin |
| Q86UW7 | CADPS2 | calcium dependent secretion activator 2 |
| Q8N4E7 | FTMT | ferritin mitochondrial |
| Q9C009 | FOXQ1 | forkhead box Q1 |
| Q96A70 | AZIN2 | antizyme inhibitor 2 |
|  | DBA2 | Diamond-Blackfan anemia 2 |
| Q8TE54 | SLC26A7 | solute carrier family 26 member 7 |
| Q8TCU5 | GRIN3A | glutamate ionotropic receptor NMDA type subunit 3A |
| O60391 | GRIN3B | glutamate ionotropic receptor NMDA type subunit 3B |
| Q6Q788 | APOA5 | apolipoprotein A5 |
| Q8IWU9 | TPH2 | tryptophan hydroxylase 2 |
| Q96NE9 | FRMD6 | FERM domain containing 6 |
| Q8WXH5 | SOCS4 | suppressor of cytokine signaling 4 |
| Q8IYS5 | OSCAR | osteoclast associated, immunoglobulin-like receptor |
| Q8TD46 | CD200R1 | CD200 receptor 1 |
| Q8TF66 | LRRC15 | leucine rich repeat containing 15 |
| Q56VL3 | OCIAD2 | OCIA domain containing 2 |
| Q9H598 | SLC32A1 | solute carrier family 32 member 1 |
| Q9NTU7 | CBLN4 | cerebellin 4 precursor |
| Q8WXA9 | SREK1 | splicing regulatory glutamic acid and lysine rich protein 1 |
| Q86YI8 | PHF13 | PHD finger protein 13 |
| Q5VWK5 | IL23R | interleukin 23 receptor |
| Q8N271 | PROM2 | prominin 2 |
| Q96MV8 | ZDHHC15 | zinc finger DHHC-type containing 15 |
| Q6ISU1 | PTCRA | pre T cell antigen receptor alpha |
| Q8IZD4 | DCP1B | decapping mRNA 1B |
| Q5VWX1 | KHDRBS2 | KH RNA binding domain containing, signal transduction associated 2 |
| Q2M385 | MPEG1 | macrophage expressed 1 |
| Q6ZMV9 | KIF6 | kinesin family member 6 |
| Q9H4W6 | EBF3 | early B cell factor 3 |
| Q5SRE7 | PHYHD1 | phytanoyl-CoA dioxygenase domain containing 1 |
| Q8NFD2 | ANKK1 | ankyrin repeat and kinase domain containing 1 |
| Q8IZI9 | IFNL3 | interferon lambda 3 |
|  | DAOA-AS1 | DAOA antisense RNA 1 |
| Q8N9W4 | GOLGA6L2 | golgin A6 family-like 2 |
| P25391 | LAMA1 | laminin subunit alpha 1 |
| Q8NBB4 | ZSCAN1 | zinc finger and SCAN domain containing 1 |
| Q5VXT5 | SYPL2 | synaptophysin like 2 |
| Q8NBC4 | C20orf203 | chromosome 20 open reading frame 203 |
| Q8N9Q2 | SREK1IP1 | SREK1 interacting protein 1 |
| Q96AH8 | RAB7B | RAB7B, member RAS oncogene family |
| Q86YW5 | TREML1 | triggering receptor expressed on myeloid cells like 1 |
| P60323 | NANOS3 | nanos C2HC-type zinc finger 3 |
| P61366 | OSTN | osteocrin |
| P83859 | QRFP | pyroglutamylated RFamide peptide |
| Q86SJ2 | AMIGO2 | adhesion molecule with Ig like domain 2 |
| O60741 | HCN1 | hyperpolarization activated cyclic nucleotide gated potassium channel 1 |
| Q86XR7;  Q9Y3B3 | TICAM2 | toll like receptor adaptor molecule 2 |
| O43861 | ATP9B | ATPase phospholipid transporting 9B (putative) |
| Q8WYQ3 | CHCHD10 | coiled-coil-helix-coiled-coil-helix domain containing 10 |
|  | MIRLET7D | microRNA let-7d |
|  | MIR100 | microRNA 100 |
|  | MIR137 | microRNA 137 |
|  | MIR144 | microRNA 144 |
|  | MIR155 | microRNA 155 |
|  | MIR15A | microRNA 15a |
|  | MIR188 | microRNA 188 |
|  | MIR195 | microRNA 195 |
|  | MIR19B1 | microRNA 19b-1 |
|  | MIR20A | microRNA 20a |
|  | MIR200B | microRNA 200b |
|  | MIR214 | microRNA 214 |
|  | MIR219A1 | microRNA 219a-1 |
|  | MIR22 | microRNA 22 |
|  | MIR222 | microRNA 222 |
|  | MIR23B | microRNA 23b |
|  | MIR26B | microRNA 26b |
|  | MIR296 | microRNA 296 |
|  | MIR30E | microRNA 30e |
|  | ASS1P1 | argininosuccinate synthetase 1 pseudogene 1 |
|  | MIR339 | microRNA 339 |
|  | MIR375 | microRNA 375 |
|  | MIR451A | microRNA 451a |
|  | MIR485 | microRNA 485 |
| P0C0P6 | NPS | neuropeptide S |
|  | MIR455 | microRNA 455 |
|  | LOC646506 | lysosomal associated membrane protein 1 pseudogene |
| A6NCL1 | GMNC | geminin coiled-coil domain containing |
|  | SCFV | single-chain Fv fragment |
| P14598 | NCF1 | neutrophil cytosolic factor 1 |
|  | GGTLC5P | gamma-glutamyltransferase light chain 5 pseudogene |
| Q86X60 | FAM72B | family with sequence similarity 72 member B |
|  | MIR545 | microRNA 545 |
|  | MIR590 | microRNA 590 |
|  | MIR603 | microRNA 603 |
|  | MIR616 | microRNA 616 |
|  | SNORD118 | small nucleolar RNA, C/D box 118 |
| P0CV98;  P0CW01;  Q01534 | TSPY3 | testis specific protein Y-linked 3 |
| B5MD39 | GGTLC3 | gamma-glutamyltransferase light chain family member 3 |
| P0CV99 | TSPY4 | testis specific protein Y-linked 4 |
| P36268 | GGT2 | gamma-glutamyltransferase 2 |
| P41597 | CCR2 | C-C motif chemokine receptor 2 |
|  | GGTLC4P | gamma-glutamyltransferase light chain 4 pseudogene |
|  | AD10 | Alzheimer disease-10 |
|  | CDKN2B-AS1 | CDKN2B antisense RNA 1 |
|  | MIR937 | microRNA 937 |
|  | AD14 | Alzheimer disease 14 |
|  | AD12 | Alzheimer disease 12 |
| P0CV98;  P0CW01;  Q01534 | TSPY10 | testis specific protein Y-linked 10 |
|  | MIR1185-1 | microRNA 1185-1 |
| Q86XR7;  Q9Y3B3 | TMED7-TICAM2 | TMED7-TICAM2 readthrough |
|  | FAS-AS1 | FAS antisense RNA 1 |
|  | PARK16 | Parkinson disease 16 (susceptibility) |
| P0DMP1 | MTRNR2L12 | MT-RNR2 like 12 |
|  | MIR4504 | microRNA 4504 |
|  | GDNF-AS1 | GDNF antisense RNA 1 |
|  | THRA1/BTR | uncharacterized LOC105371807 |
|  | CST12P | cystatin 12, pseudogene |
|  | LOC107987479 | cytochrome P450 2D6 |

**Table S2 The putative major chemical ingredients and ADME parameters in QYG**

| **Serial Number** | **Herb** | **Molecule Name** | **OB** | **DL** | **BBB** | **Canonical SMILES** |
| --- | --- | --- | --- | --- | --- | --- |
| RR-01 | *Radix Rehmanniae* | Catalpol | 5.07 | 0.44 | -2.33 | C1=COC(C2C1C(C3C2(O3)CO)O)OC4C(C(C(C(O4)CO)O)O)O |
| RR-02 | *Radix Rehmanniae* | Campesterol | 37.58 | 0.71 | 0.95 | CC(C)C(C)CCC(C)C1CCC2C1(CCC3C2CC=C4C3(CCC(C4)O)C)C |
| RR-03 | *Radix Rehmanniae* | Rehmaglutin D | 57.03 | 0.10 | -0.61 | C1COC2C3C1C(C(C3(CO2)O)Cl)O |
| RR-04 | *Radix Rehmanniae* | Rehmaglutin B | 64.62 | 0.11 | -1.11 | C1C2C3C(OCC3(C(C2O)Cl)O)OC1O |
| RR-05 | *Radix Rehmanniae* | Rehmaglutin A | 29.70 | 0.10 | -1.25 | C1COC2C3C1C(C(C3(CO2)O)O)O |
| RR-06 | *Radix Rehmanniae* | Adenosine | 15.98 | 0.18 | -2.22 | C1=NC(=C2C(=N1)N(C=N2)C3C(C(C(O3)CO)O)O)N |
| RR-07 | *Radix Rehmanniae* | Acteoside | 2.94 | 0.62 | -2.97 | CC1C(C(C(C(O1)OC2C(C(OC(C2OC(=O)C=CC3=CC(=C(C=C3)O)O)CO)OCCC4=CC(=C(C=C4)O)O)O)O)O)O |
| RR-08 | *Radix Rehmanniae* | Daucosterol | 20.63 | 0.63 | -0.61 | CCC(CCC(C)C1CCC2C1(CCC3C2CC=C4C3(CCC(C4)OC5C(C(C(C(O5)CO)O)O)O)C)C)C(C)C |
| RR-09 | *Radix Rehmanniae* | Echinacoside | 3.14 | 0.38 | -4.05 | CC1C(C(C(C(O1)OC2C(C(OC(C2OC(=O)C=CC3=CC(=C(C=C3)O)O)COC4C(C(C(C(O4)CO)O)O)O)OCCC5=CC(=C(C=C5)O)O)O)O)O)O |
| RR-10 | *Radix Rehmanniae* | Martynoside | 12.91 | 0.58 | -2.52 | CC1C(C(C(C(O1)OC2C(C(OC(C2OC(=O)C=CC3=CC(=C(C=C3)O)OC)CO)OCCC4=CC(=C(C=C4)OC)O)O)O)O)O |
| RR-11 | *Radix Rehmanniae* | Rehmaionoside B | 3.24 | 0.33 | -2.06 | CC(C=CC1(C(CCCC1(C)OC2C(C(C(C(O2)CO)O)O)O)(C)C)O)O |
| RR-12 | *Radix Rehmanniae* | Rehmaionoside C | 12.89 | 0.34 | -1.42 | CC(=O)C=CC1(C(CCCC1(C)OC2C(C(C(C(O2)CO)O)O)O)(C)C)O |
| RR-13 | *Radix Rehmanniae* | Rehmannin | 38.68 | 0.57 | -0.3 | CC=C(C)C(=O)OC1CC(CC2C1(CCC3(C2=CCC4C3(CCC5C4(CCC(=O)C5(C)C)C)C)C)C(=O)O)(C)C |
| RR-14 | *Radix Rehmanniae* | β-sitosterol | 36.91 | 0.75 | 0.99 | CCC(CCC(C)C1CCC2C1(CCC3C2CC=C4C3(CCC(C4)O)C)C)C(C)C |
| PC-01 | *Poria Cocos* (Schw.) Wolf | Trametenolic acid | 38.71 | 0.80 | -0.14 | CC(=CCCC(C1CCC2(C1(CCC3=C2CCC4C3(CCC(C4(C)C)O)C)C)C)C(=O)O)C |
| PC-02 | *Poria Cocos* (Schw.) Wolf | Ergosta-7,22E-dien-3beta-ol | 43.51 | 0.72 | 0.91 | CC(C)C(C)C=CC(C)C1CCC2C1(CCC3C2=CCC4C3(CCC(C4)O)C)C |
| PC-03 | *Poria Cocos* (Schw.) Wolf | Ergosterol peroxide | 40.36 | 0.81 | 0.34 | CC(C)C(C)C=CC(C)C1CCC2C1(CCC3C24C=CC5(C3(CCC(C5)O)C)OO4)C |
| PC-04 | *Poria Cocos* (Schw.) Wolf | Polyporenic acid C | 38.26 | 0.82 | -0.57 | CC(C)C(=C)CCC(C1C(CC2(C1(CC=C3C2=CCC4C3(CCC(=O)C4(C)C)C)C)C)O)C(=O)O |
| PC-05 | *Poria Cocos* (Schw.) Wolf | Eburicoic acid | 38.70 | 0.81 | -0.04 | CC(C)C(=C)CCC(C1CCC2(C1(CCC3=C2CCC4C3(CCC(C4(C)C)O)C)C)C)C(=O)O |
| PC-06 | *Poria Cocos* (Schw.) Wolf | Pachymic acid | 33.63 | 0.81 | -0.57 | CC(C)C(=C)CCC(C1C(CC2(C1(CCC3=C2CCC4C3(CCC(C4(C)C)OC(=O)C)C)C)C)O)C(=O)O |
| PC-07 | *Poria Cocos* (Schw.) Wolf | Poricoic acid A | 30.61 | 0.76 | -0.93 | CC(C)C(=C)CCC(C1C(CC2(C1(CC=C3C2=CCC(C3(C)CCC(=O)O)C(=C)C)C)C)O)C(=O)O |
| PC-08 | *Poria Cocos* (Schw.) Wolf | Poricoic acid B | 30.52 | 0.75 | -0.87 | CC(=CCCC(C1C(CC2(C1(CC=C3C2=CCC(C3(C)CCC(=O)O)C(=C)C)C)C)O)C(=O)O)C |
| PC-09 | *Poria Cocos* (Schw.) Wolf | Poricoic acid C | 38.15 | 0.75 | -0.41 | CC(C)C(=C)CCC(C1CCC2(C1(CC=C3C2=CCC(C3(C)CCC(=O)O)C(=C)C)C)C)C(=O)O |
| PC-10 | *Poria Cocos* (Schw.) Wolf | Tumulosic acid | 29.88 | 0.81 | -0.47 | CC(C)C(=C)CCC(C1C(CC2(C1(CCC3=C2CCC4C3(CCC(C4(C)C)O)C)C)C)O)C(=O)O |
| PC-11 | *Poria Cocos* (Schw.) Wolf | Poricoic acid D | 22.38 | 0.78 | -1.29 | CC(=C)C1CC=C2C(=CCC3(C2(CC(C3C(CCC(=C)C(C)(C)O)C(=O)O)O)C)C)C1(C)CCC(=O)O |
| PC-12 | *Poria Cocos* (Schw.) Wolf | Poricoic acid DM | 29.32 | 0.78 | -0.94 | CC(=C)C1CC=C2C(=CCC3(C2(CC(C3C(CCC(=C)C(C)(C)O)C(=O)O)O)C)C)C1(C)CCC(=O)OC |
| PC-13 | *Poria Cocos* (Schw.) Wolf | Hederagenin | 36.91 | 0.75 | 0.96 | CC1(CCC2(CCC3(C(=CCC4C3(CCC5C4(CCC(C5(C)CO)O)C)C)C2C1)C)C(=O)O)C |
| PC-14 | *Poria Cocos* (Schw.) Wolf | Dehydroeburicoic acid | 44.17 | 0.83 | -0.16 | CC(C)C(=C)CCC(C1CCC2(C1(CC=C3C2=CCC4C3(CCC(C4(C)C)O)C)C)C)C(=O)O |
| PC-15 | *Poria Cocos* (Schw.) Wolf | Ergosterol | 14.29 | 0.72 | 1.04 | CC(C)C(C)C=CC(C)C1CCC2C1(CCC3C2=CC=C4C3(CCC(C4)O)C)C |
| PG-01 | *Panax Ginseng* C. A. Mey | Diop | 43.59 | 0.39 | 0.26 | CC(C)CCCCCOC(=O)C1=CC=CC=C1C(=O)OCCCCCC(C)C |
| PG-02 | *Panax Ginseng* C. A. Mey | Stigmasterol | 43.83 | 0.76 | 1.00 | CCC(C=CC(C)C1CCC2C1(CCC3C2CC=C4C3(CCC(C4)O)C)C)C(C)C |
| PG-03 | *Panax Ginseng* C. A. Mey | beta-Sitosterol | 36.91 | 0.75 | 0.99 | CCC(CCC(C)C1CCC2C1(CCC3C2CC=C4C3(CCC(C4)O)C)C)C(C)C |
| PG-04 | *Panax Ginseng* C. A. Mey | Inermin | 65.83 | 0.54 | 0.36 | C1C2C(C3=C(O1)C=C(C=C3)O)OC4=CC5=C(C=C24)OCO5 |
| PG-05 | *Panax Ginseng* C. A. Mey | Aposiopolamine | 66.65 | 0.22 | 0.40 | CN1C2CC(CC1C3C2O3)OC(=O)C(=C)C4=CC=CC=C4 |
| PG-06 | *Panax Ginseng* C. A. Mey | Celabenzine | 101.88 | 0.49 | 0.05 | C1CCN(CCCNC(=O)CC(NC1)C2=CC=CC=C2)C(=O)C3=CC=CC=C3 |
| PG-07 | *Panax Ginseng* C. A. Mey | Deoxyharringtonine | 39.27 | 0.81 | -0.25 | CC(C)CCC(CC(=O)OC)(C(=O)OC1C2C3=CC4=C(C=C3CCN5C2(CCC5)C=C1OC)OCO4)O |
| PG-08 | *Panax Ginseng* C. A. Mey | Arachidonate | 45.57 | 0.20 | 0.58 | CCCCCC=CCC=CCC=CCC=CCCCC(=O)O |
| PG-09 | *Panax Ginseng* C. A. Mey | Frutinone A | 65.90 | 0.34 | 0.46 | C1=CC=C2C(=C1)C3=C(C(=O)C4=CC=CC=C4O3)C(=O)O2 |
| PG-10 | *Panax Ginseng* C. A. Mey | Ginsenoside Rh2 | 36.32 | 0.56 | -1.38 | CC(=CCCC(C)(C1CCC2(C1C(CC3C2(CCC4C3(CCC(C4(C)C)OC5C(C(C(C(O5)CO)O)O)O)C)C)O)C)O)C |
| PG-11 | *Panax Ginseng* C. A. Mey | Ginsenoside Rh4 | 31.11 | 0.78 | -0.18 | CC(=CCC=C(C)C1CCC2(C1C(CC3C2(CC(C4C3(CCC(C4(C)C)O)C)OC5C(C(C(C(O5)CO)O)O)O)C)O)C)C |
| PG-12 | *Panax Ginseng* C. A. Mey | Girinimbin | 61.22 | 0.31 | 1.22 | CC1=C2C(=C3C(=C1)C4=CC=CC=C4N3)C=CC(O2)(C)C |
| PG-13 | *Panax Ginseng* C. A. Mey | Gomisin B | 31.99 | 0.83 | 0.18 | CC=C(C)C(=O)OC1C2=CC(=C(C(=C2C3=C(C4=C(C=C3CC(C1(C)O)C)OCO4)OC)OC)OC)OC |
| PG-14 | *Panax Ginseng* C. A. Mey | Malkangunin | 57.71 | 0.63 | -0.17 | CC1CCC(C2(C13CC(C(C2OC(=O)C4=CC=CC=C4)OC(=O)C)C(O3)(C)C)CO)O |
| PG-15 | *Panax Ginseng* C. A. Mey | Panaxadiol | 33.09 | 0.79 | 0.23 | CC1(CCCC(O1)(C)C2CCC3(C2C(CC4C3(CCC5C4(CCC(C5(C)C)O)C)C)O)C)C |
| PG-16 | *Panax Ginseng* C. A. Mey | Suchilactone | 57.52 | 0.56 | 0.28 | COC1=C(C=C(C=C1)CC2COC(=O)C2=CC3=CC4=C(C=C3)OCO4)OC |
| PG-17 | *Panax Ginseng* C. A. Mey | Fumarine | 59.26 | 0.83 | -0.13 | CN1CCC2=CC3=C(C=C2C(=O)CC4=C(C1)C5=C(C=C4)OCO5)OCO3 |
| PG-18 | *Panax Ginseng* C. A. Mey | Panaxynol | 42.44 | 0.10 | 1.03 | CCCCCCCC=CCC#CC#CC(C=C)O |
| PG-19 | *Panax Ginseng* C. A. Mey | 20(S)-Protopanaxadiol | 29.69 | 0.77 | -0.52 | CC(=CCCC(C)(C1CCC2(C1C(CC3C2(CCC4C3(CCC(C4(C)C)O)C)C)O)C)O)C |
| PG-20 | *Panax Ginseng* C. A. Mey | Ginsenoside La | 17.74 | 0.14 | -3.25 | CC(=CC1CC(C2CCC3(C2C(O1)CC4C3CCC5C4(CCC(C5(C)C)OC6C(C(C(C(O6)CO)O)O)O)C)C)(C)OC7C(C(C(C(O7)CO)O)O)O)C |
| PG-21 | *Panax Ginseng* C. A. Mey | Ginsenoside Ro | 1.98 | 0.05 | -3.92 | CC1(CCC2(CCC3(C(=CCC4C3(CCC5C4(CCC(C5(C)C)OC6C(C(C(C(O6)C(=O)O)O)O)OC7C(C(C(C(O7)CO)O)O)O)C)C)C2C1)C)C(=O)OC8C(C(C(C(O8)CO)O)O)O)C |
| PG-22 | *Panax Ginseng* C. A. Mey | Gypenoside LXIX | 7.73 | 0.04 | -5.38 | CC1(C2CCC3(C(C2(CCC1OC4C(C(C(C(O4)CO)O)O)OC5C(C(C(C(O5)CO)O)O)O)C)CC(C6C3(CCC6C(C)(CC=CC(C)(C)O)OC7C(C(C(C(O7)COC8C(C(C(CO8)O)O)O)O)O)O)C)O)C)C |
| PG-23 | *Panax Ginseng* C. A. Mey | Ginsenoside Rc | 8.16 | 0.04 | -5.69 | CC(=CCCC(C)(C1CCC2(C1C(CC3C2(CCC4C3(CCC(C4(C)C)OC5C(C(C(C(O5)CO)O)O)OC6C(C(C(C(O6)CO)O)O)O)C)C)O)C)OC7C(C(C(C(O7)COC8C(C(C(O8)CO)O)O)O)O)O)C |
| PG-24 | *Panax Ginseng* C. A. Mey | Ginsenoside Re | 4.27 | 0.12 | -4.39 | CC1C(C(C(C(O1)OC2C(C(C(OC2OC3CC4(C(CC(C5C4(CCC5C(C)(CCC=C(C)C)OC6C(C(C(C(O6)CO)O)O)O)C)O)C7(C3C(C(CC7)O)(C)C)C)C)CO)O)O)O)O)O |
| PG-25 | *Panax Ginseng* C. A. Mey | Ginsenoside rf | 17.74 | 0.24 | -3.35 | CC(=CCCC(C)(C1CCC2(C1C(CC3C2(CC(C4C3(CCC(C4(C)C)O)C)OC5C(C(C(C(O5)CO)O)O)OC6C(C(C(C(O6)CO)O)O)O)C)O)C)O)C |
| PG-26 | *Panax Ginseng* C. A. Mey | Sanchinoside C1 | 10.04 | 0.28 | -3.50 | CC(=CCCC(C)(C1CCC2(C1C(CC3C2(CC(C4C3(CCC(C4(C)C)O)C)OC5C(C(C(C(O5)CO)O)O)O)C)O)C)OC6C(C(C(C(O6)CO)O)O)O)C |
| PG-27 | *Panax Ginseng* C. A. Mey | Panaxytriol | 33.76 | 0.13 | -0.98 | CCCCCCCC(C(CC#CC#CC(C=C)O)O)O |
| PG-28 | *Panax Ginseng* C. A. Mey | 20(R)-Ginsenoside Rg2 | 10.09 | 0.26 | -2.95 | CC1C(C(C(C(O1)OC2C(C(C(OC2OC3CC4(C(CC(C5C4(CCC5C(C)(CCC=C(C)C)O)C)O)C6(C3C(C(CC6)O)(C)C)C)C)CO)O)O)O)O)O |
| PG-29 | *Panax Ginseng* C. A. Mey | 20-(S)-Ginsenoside Rg3 | 13.69 | 0.22 | -2.47 | CC(=CCCC(C)(C1CCC2(C1C(CC3C2(CCC4C3(CCC(C4(C)C)OC5C(C(C(C(O5)CO)O)O)OC6C(C(C(C(O6)CO)O)O)O)C)C)O)C)O)C |
| PG-30 | *Panax Ginseng* C. A. Mey | Aposcopolamine | 59.68 | 0.25 | 0.68 | CN1C2CC(CC1C3C2O3)OC(=O)C(=C)C4=CC=CC=C4 |
| PG-31 | *Panax Ginseng* C. A. Mey | Campesterol | 37.58 | 0.71 | 0.93 | CC(C)C(C)CCC(C)C1CCC2C1(CCC3C2CC=C4C3(CCC(C4)O)C)C |
| PG-32 | *Panax Ginseng* C. A. Mey | Ginsenoside Rb1 | 6.24 | 0.04 | -5.60 | CC(=CCCC(C)(C1CCC2(C1C(CC3C2(CCC4C3(CCC(C4(C)C)OC5C(C(C(C(O5)CO)O)O)OC6C(C(C(C(O6)CO)O)O)O)C)C)O)C)OC7C(C(C(C(O7)COC8C(C(C(C(O8)CO)O)O)O)O)O)O)C |
| PG-33 | *Panax Ginseng* C. A. Mey | Ginsenoside Rg1 | 9.03 | 0.28 | -3.41 | CC(=CCCC(C)(C1CCC2(C1C(CC3C2(CC(C4C3(CCC(C4(C)C)O)C)OC5C(C(C(C(O5)CO)O)O)O)C)O)C)OC6C(C(C(C(O6)CO)O)O)O)C |
| PG-34 | *Panax Ginseng* C. A. Mey | Gomisin A | 30.69 | 0.78 | -0.02 | CC1CC2=CC3=C(C(=C2C4=C(C(=C(C=C4CC1(C)O)OC)OC)OC)OC)OCO3 |
| AC-01 | *Apis Cerana* Fabricius. | PQN | 47.60 | 0.66 | 0.81 | CC1=C(C(=O)C2=CC=CC=C2C1=O)CC=C(C)CCCC(C)CCCC(C)CCCC(C)C |
| AC-02 | *Apis Cerana* Fabricius. | [Fructose](http://www.megabionet.org/tcmid/ingredient/7970/) | 1.68 | 0.03 | -4.58 | C1C(C(C(C(O1)(CO)O)O)O)O |
| AC-03 | *Apis Cerana* Fabricius. | [Glucose](http://www.megabionet.org/tcmid/ingredient/23487/) | 24.44 | 0.03 | -4.65 | C(C1C(C(C(C(O1)O)O)O)O)O |
| AC-04 | *Apis Cerana* Fabricius. | Maltose | 1.80 | 0.24 | -6.56 | C(C1C(C(C(C(O1)OC2C(OC(C(C2O)O)O)CO)O)O)O)O |
| AC-05 | *Apis Cerana* Fabricius. | [Pyridoxine](http://www.megabionet.org/tcmid/ingredient/18247/) | 61.54 | 0.04 | -0.81 | CC1=NC=C(C(=C1O)CO)CO |
| AC-06 | *Apis Cerana* Fabricius. | Acetylcholine | 27.80 | 0.02 | 1.16 | CC(=O)OCC[N+](C)(C)C |
| AC-07 | *Apis Cerana* Fabricius. | Sucrose | 7.17 | 0.23 | -6.67 | C(C1C(C(C(C(O1)OC2(C(C(C(O2)CO)O)O)CO)O)O)O)O |

**Table S3 The detailed target information of the ingredients of herbs**

| **Ingredients** | **Targets** | **Uniprot ID** | **Description** |
| --- | --- | --- | --- |
| RR-01 | AMY2A | P04746 | Pancreatic alpha-amylase |
| RR-01 | FGF1 | P05230 | Fibroblast growth factor 1 |
| RR-01 | FGF2 | P09038 | Fibroblast growth factor 2 |
| RR-01 | LGALS4 | P56470 | Galectin-4 |
| RR-01 | LGALS3 | P17931 | Galectin-3 |
| RR-01 | LGALS8 | O00214 | Galectin-8 |
| RR-01 | LGALS9 | O00182 | Galectin-9 |
| RR-01 | SLC5A4 | Q9NY91 | Low affinity sodium-glucose cotransporter |
| RR-01 | TYR | P14679 | Tyrosinase |
| RR-01 | VEGFA | P15692 | Vascular endothelial growth factor A |
| RR-01 | MAPT | Q9NUW8 | Microtubule-associated protein tau |
| RR-01 | TDP1 | Q9NR56 | Tyrosyl-DNA phosphodiesterase 1 |
| RR-01 | MBNL1 | Q5VZF2 | Muscleblind-like protein 1 |
| RR-01 | MBNL2 | Q9NUK0 | Muscleblind-like protein 2 |
| RR-01 | MBNL3 | P17706 | Muscleblind-like protein 3 |
| RR-01 | PTPN2 | P18031 | Tyrosine-protein phosphatase non-receptor type 2 |
| RR-01 | PTPN1 | P06493 | Tyrosine-protein phosphatase non-receptor type 1 |
| RR-01 | CDK1 | P11802 | Cyclin-dependent kinase 1 |
| RR-01 | CDK4 | P24941 | Cyclin- dependent kinase 4 |
| RR-01 | CDK2 | Q00526 | Cyclin-dependent kinase 2 |
| RR-01 | CDK3 | Q00534 | Cyclin-dependent kinase 3 |
| RR-01 | CDK6 | O43570 | Cyclin-dependent kinase 6 |
| RR-01 | CA12 | P00915 | Carbonic anhydrase 12 |
| RR-01 | CA1 | P00918 | Carbonic anhydrase 1 |
| RR-01 | CA2 | P10636 | Carbonic anhydrase 2 |
| RR-01 | AKT1 | P31749 | v-akt Murine thymoma viral oncogene homolog 1 |
| RR-01 | GDNF | P39905 | Glial cell derived neurotrophic factor |
| RR-01 | RPS13 | P62277 | Ribosomal protein S13 |
| RR-02 | ABCB11 | O95342 | Bile salt export pump |
| RR-02 | AR | P10275 | Androgen receptor |
| RR-02 | CA2 | P00918 | Carbonic anhydrase 2 |
| RR-02 | CA1 | P00915 | Carbonic anhydrase 1 |
| RR-02 | SERPINA6 | P08185 | Corticosteroid-binding globulin |
| RR-02 | CD4 | P01730 | T-cell surface glycoprotein CD4 |
| RR-02 | CDC45 | O75419 | Cell division control protein 45 homolog |
| RR-02 | CYP17A1 | P05093 | Steroid 17-alpha-hydroxylase/17,20 lyase |
| RR-02 | CYP19A1 | P11511 | Aromatase |
| RR-02 | CYP24A1 | Q07973 | 1,25-dihydroxyvitamin D(3) 24-hydroxylase, mitochondrial |
| RR-02 | CYP27B1 | O15528 | 25-hydroxyvitamin D-1 alpha hydroxylase, mitochondrial |
| RR-02 | POLA1 | P09884 | DNA polymerase alpha catalytic subunit |
| RR-02 | EBP | Q15125 | 3-beta-hydroxysteroid-Delta(8),Delta(7)-isomerase |
| RR-02 | ESR1 | P03372 | Estrogen receptor |
| RR-02 | ESR2 | Q92731 | Estrogen receptor beta |
| RR-02 | FGF1 | P05230 | Fibroblast growth factor 1 |
| RR-02 | G6PD | P11413 | Glucose-6-phosphate 1-dehydrogenase |
| RR-02 | GPBAR1 | Q8TDU6 | G-protein coupled bile acid receptor 1 |
| RR-02 | CDC25A | P30304 | M-phase inducer phosphatase 1 |
| RR-02 | ABCC4 | O15439 | Multidrug resistance-associated protein 4 |
| RR-02 | NPC1L1 | Q9UHC9 | Niemann-Pick C1-like protein 1 |
| RR-02 | NPC1 | O15118 | Niemann-Pick C1 protein |
| RR-02 | NR1H3 | Q13133 | Oxysterols receptor LXR-alpha |
| RR-02 | NR1H4 | Q96RI1 | Bile acid receptor |
| RR-02 | SLC10A2 | Q12908 | Ileal sodium/bile acid cotransporter |
| RR-02 | SLC10A1 | Q14973 | Sodium/bile acid cotransporter |
| RR-02 | ATIC | P31939 | Bifunctional purine biosynthesis protein PURH |
| RR-02 | RORA | P35398 | Nuclear receptor ROR-alpha |
| RR-02 | RORC | P51449 | Nuclear receptor ROR-gamma |
| RR-02 | SLC22A3 | O75751 | Solute carrier family 22 member 3 |
| RR-02 | SRD5A1 | P18405 | 3-oxo-5-alpha-steroid 4-dehydrogenase 1 |
| RR-02 | SRD5A2 | P31213 | 3-oxo-5-alpha-steroid 4-dehydrogenase 2 |
| RR-02 | SHBG | P04278 | Sex hormone-binding globulin |
| RR-02 | SHH | Q15465 | Sonic hedgehog protein |
| RR-02 | ST3GAL1 | Q11201 | CMP-N-acetylneuraminate-beta-galactosamide-alpha-2,3-sialyltransferase 1 |
| RR-02 | SREBF2 | Q12772 | Sterol regulatory element-binding protein 2 |
| RR-02 | VDR | P11473 | Vitamin D3 receptor |
| RR-02 | GC | P02774 | Vitamin D-binding protein |
| RR-02 | TDP1 | Q9NUW8 | Tyrosyl-DNA phosphodiesterase 1 |
| RR-02 | NR1H2 | P55055 | Oxysterols receptor LXR-beta |
| RR-02 | HMGCR | P04035 | 3-hydroxy-3-methylglutaryl-coenzyme A reductase |
| RR-02 | CYP51A1 | Q16850 | Lanosterol 14-alpha demethylase |
| RR-02 | SREBF1 | P36956 | Sterol regulatory element-binding protein 1 |
| RR-02 | LDLR | P01130 | Low-density lipoprotein receptor |
| RR-02 | VLDLR | P98155 | Very low-density lipoprotein receptor |
| RR-02 | LRP8 | Q14114 | Low-density lipoprotein receptor-related protein 8 |
| RR-02 | HSD3B2 | P26439 | 3 beta-hydroxysteroid dehydrogenase/Delta 5-->4-isomerase type 2 |
| RR-02 | DHCR24 | Q15392 | Delta(24)-sterol reductase |
| RR-02 | CYP7A1 | P22680 | Cholesterol 7-alpha-monooxygenase |
| RR-02 | ABCG8 | Q9H221 | ATP-binding cassette sub-family G member 8 |
| RR-02 | CSN1S1 | P47710 | Alpha-S1-casein |
| RR-02 | ABCG5 | Q9H222 | ATP-binding cassette sub-family G member 5 |
| RR-03 | TDP1 | Q9NUW8 | Tyrosyl-DNA phosphodiesterase 1 |
| RR-03 | MAPT | P10636 | Microtubule-associated protein tau |
| RR-03 | FUCA1 | P04066 | Tissue alpha-L-fucosidase |
| RR-03 | FUCA2 | Q9BTY2 | Plasma alpha-L-fucosidase |
| RR-03 | MBNL1 | Q9NR56 | Muscleblind-like protein 1 |
| RR-03 | MBNL2 | Q5VZF2 | Muscleblind-like protein 2 |
| RR-03 | MBNL3 | Q9NUK0 | Muscleblind-like protein 3 |
| RR-03 | ADRA2A | P08913 | Alpha-2A adrenergic receptor |
| RR-03 | CYP2D6 | P10635 | Cytochrome P450 2D6 |
| RR-03 | DRD2 | P14416 | D(2) dopamine receptor |
| RR-03 | ADRA2B | P18089 | Alpha-2B adrenergic receptor |
| RR-03 | ADRA2C | P18825 | Alpha-2C adrenergic receptor |
| RR-03 | DRD3 | P35462 | D(3) dopamine receptor |
| RR-03 | HTR6 | P50406 | 5-hydroxytryptamine receptor 6 |
| RR-03 | CYP2J2 | P51589 | Cytochrome P450 2J2 |
| RR-04 | FUCA1 | P04066 | Tissue alpha-L-fucosidase |
| RR-04 | FUCA2 | Q9BTY2 | Plasma alpha-L-fucosidase |
| RR-04 | TDP1 | Q9NUW8 | Tyrosyl-DNA phosphodiesterase 1 |
| RR-04 | MAPT | P10636 | Microtubule-associated protein tau |
| RR-04 | CA9 | Q16790 | Carbonic anhydrase 9 |
| RR-04 | CA1 | P00915 | Carbonic anhydrase 1 |
| RR-04 | CA2 | P00918 | Carbonic anhydrase 2 |
| RR-04 | CA3 | P07451 | Carbonic anhydrase 3 |
| RR-04 | CA5A | P35218 | Carbonic anhydrase 5A |
| RR-04 | CA7 | P43166 | Carbonic anhydrase 7 |
| RR-04 | CA13 | Q8N1Q1 | Carbonic anhydrase 13 |
| RR-04 | CA5B | Q9Y2D0 | Carbonic anhydrase 5B |
| RR-04 | ADRA1D | P25100 | Alpha-1D adrenergic receptor |
| RR-04 | ADRA1A | P35348 | Alpha-1A adrenergic receptor |
| RR-04 | ADRA1B | P35368 | Alpha-1B adrenergic receptor |
| RR-05 | [HSP90AA1](http://zinc15.docking.org/genes/HSP90AA1) | P07900 | Heat shock protein HSP 90-alpha |
| RR-05 | MAPT | P10636 | Microtubule-associated protein tau |
| RR-05 | TDP1 | Q9NUW8 | Tyrosyl-DNA phosphodiesterase 1 |
| RR-05 | MBNL1 | Q9NR56 | Muscleblind-like protein 1 |
| RR-05 | MBNL2 | Q5VZF2 | Muscleblind-like protein 2 |
| RR-05 | MBNL3 | Q9NUK0 | Muscleblind-like protein 3 |
| RR-05 | FUCA1 | P04066 | Tissue alpha-L-fucosidase |
| RR-05 | FUCA2 | Q9BTY2 | Plasma alpha-L-fucosidase |
| RR-05 | GBA | P04062 | Glucosylceramidase |
| RR-05 | FGF1 | P05230 | Fibroblast growth factor 1 |
| RR-05 | FGF2 | P09038 | Fibroblast growth factor 2 |
| RR-05 | VEGFA | P15692 | Vascular endothelial growth factor A |
| RR-05 | HPSE | Q9Y251 | Heparanase 8 kDa subunit |
| RR-05 | HPSE2 | Q8WWQ2 | Inactive heparanase-2 |
| RR-05 | CHRM1 | P11229 | Muscarinic acetylcholine receptor M1 |
| RR-05 | CHRM2 | P08172 | Muscarinic acetylcholine receptor M2 |
| RR-06 | DCK | P27707 | Deoxycytidine kinase |
| RR-06 | DGUOK | Q16854 | Deoxyguanosine kinase, mitochondrial |
| RR-06 | MAP3K7 | [O43318](https://www.uniprot.org/uniprot/O43318) | Mitogen-activated protein kinase kinase kinase 7 |
| RR-06 | DNPH1 | [O43598](https://www.uniprot.org/uniprot/O43598) | 2'-deoxynucleoside 5'-phosphate N-hydrolase 1 |
| RR-06 | IMPDH1 | [P20839](https://www.uniprot.org/uniprot/P20839) | Inosine-5'-monophosphate dehydrogenase 1 |
| RR-06 | ADCY5 | [O95622](https://www.uniprot.org/uniprot/O95622) | Adenylate cyclase type 5 |
| RR-06 | DTYMK | [P23919](https://www.uniprot.org/uniprot/P23919) | Thymidylate kinase |
| RR-06 | KMT5C | [Q86Y97](https://www.uniprot.org/uniprot/Q86Y97) | Histone-lysine N-methyltransferase KMT5C |
| RR-06 | TARS | [P26639](https://www.uniprot.org/uniprot/P26639) | Threonine--tRNA ligase, cytoplasmic |
| RR-06 | RNASEL | [Q05823](https://www.uniprot.org/uniprot/Q05823) | 2-5A-dependent ribonuclease |
| RR-06 | AHCYL1 | [O43865](https://www.uniprot.org/uniprot/O43865) | S-adenosylhomocysteine hydrolase-like protein 1 |
| RR-06 | HARS | [P12081](https://www.uniprot.org/uniprot/P12081) | Histidine--tRNA ligase, cytoplasmic |
| RR-06 | RNASE1 | [P07998](https://www.uniprot.org/uniprot/P07998) | Ribonuclease pancreatic |
| RR-06 | RNASE2 | [P10153](https://www.uniprot.org/uniprot/P10153) | Non-secretory ribonuclease |
| RR-06 | IMPDH2 | [P12268](https://www.uniprot.org/uniprot/P12268) | Inosine-5'-monophosphate dehydrogenase 2 |
| RR-06 | HLCS | [P50747](https://www.uniprot.org/uniprot/P50747) | Biotin--protein ligase |
| RR-06 | GPR17 | [Q13304](https://www.uniprot.org/uniprot/Q13304) | Uracil nucleotide/cysteinyl leukotriene receptor |
| RR-06 | P2RX4 | [Q99571](https://www.uniprot.org/uniprot/Q99571) | P2X purinoceptor 4 |
| RR-06 | PLCG1 | [P19174](https://www.uniprot.org/uniprot/P19174) | 1-phosphatidylinositol 4,5-bisphosphate phosphodiesterase gamma-1 |
| RR-06 | MGMT | [P16455](https://www.uniprot.org/uniprot/P16455) | Methylated-DNA--protein-cysteine methyltransferase |
| RR-06 | TPMT | [P51580](https://www.uniprot.org/uniprot/P51580) | Thiopurine S-methyltransferase |
| RR-06 | ADCY10 | [Q96PN6](https://www.uniprot.org/uniprot/Q96PN6) | Adenylate cyclase type 10 |
| RR-06 | P2RY2 | [P41231](https://www.uniprot.org/uniprot/P41231) | P2Y purinoceptor 2 |
| RR-06 | ADH1A | [P07327](https://www.uniprot.org/uniprot/P07327) | Alcohol dehydrogenase 1A |
| RR-06 | RAC1 | P63000 | Ras-related C3 botulinum toxin substrate 1 |
| RR-06 | HEXA | P06865 | Beta-hexosaminidase subunit alpha |
| RR-06 | HEXB | [P07686](https://www.uniprot.org/uniprot/P07686) | Beta-hexosaminidase subunit beta |
| RR-06 | ATIC | [P31939](https://www.uniprot.org/uniprot/P31939) | Bifunctional purine biosynthesis protein PURH |
| RR-06 | TRPM2 | [O94759](https://www.uniprot.org/uniprot/O94759) | Transient receptor potential cation channel subfamily M member 2 |
| RR-06 | PRKACA | [P17612](https://www.uniprot.org/uniprot/P17612) | cAMP-dependent protein kinase catalytic subunit alpha |
| RR-06 | CD69 | Q07108 | Early activation antigen CD69 |
| RR-06 | P2RY14 | [Q15391](https://www.uniprot.org/uniprot/Q15391) | P2Y purinoceptor 14 |
| RR-06 | TK1 | [P04183](https://www.uniprot.org/uniprot/P04183) | Thymidine kinase, cytosolic |
| RR-06 | CDC42 | P60953 | Cell division control protein 42 homolog |
| RR-06 | NPEPPS | [P55786](https://www.uniprot.org/uniprot/P55786) | Puromycin-sensitive aminopeptidase |
| RR-06 | PAX8 | [Q06710](https://www.uniprot.org/uniprot/Q06710) | Paired box protein Pax-8 |
| RR-06 | P2RY12 | [Q9H244](https://www.uniprot.org/uniprot/Q9H244) | P2Y purinoceptor 12 |
| RR-06 | TYMP | P19971 | Thymidine phosphorylase |
| RR-06 | HPRT1 | [P00492](https://www.uniprot.org/uniprot/P00492) | Hypoxanthine-guanine phosphoribosyltransferase |
| RR-06 | TMIGD3 | [P0DMS9](https://www.uniprot.org/uniprot/P0DMS9) | Transmembrane domain-containing protein TMIGD3 |
| RR-06 | CHAT | P28329 | Choline O-acetyltransferase |
| RR-06 | GALR3 | [O60755](https://www.uniprot.org/uniprot/O60755) | Galanin receptor type 3 |
| RR-06 | ADORA1 | P30542 | Adenosine A1 receptor |
| RR-06 | ADORA2A | P29274 | Adenosine A2a receptor |
| RR-06 | ADK | P55263 | Adenosine kinase |
| RR-06 | ADORA3 | P0DMS8 | Adenosine A3 receptor |
| RR-06 | HSPA8 | P11142 | Heat shock cognate 71 kDa protein |
| RR-06 | HSPA5 | P11021 | 78 kDa glucose-regulated protein |
| RR-06 | AHCY | P23526 | Adenosylhomocysteinase |
| RR-06 | ADA | P00813 | Adenosine deaminase |
| RR-06 | GAPDH | P04406 | Glyceraldehyde-3-phosphate dehydrogenase liver |
| RR-06 | EHMT1 | Q9H9B1 | Histone-lysine N-methyltransferase, H3 lysine-9 specific 5 |
| RR-06 | EHMT2 | Q96KQ7 | Histone-lysine N-methyltransferase, H3 lysine-9 specific 3 |
| RR-06 | MCL1 | Q07820 | Induced myeloid leukemia cell differentiation protein Mcl-1 |
| RR-06 | SETD7 | Q8WTS6 | Histone-lysine N-methyltransferase SETD7 |
| RR-06 | EGFR | P00533 | Epidermal growth factor receptor erbB1 |
| RR-06 | SRM | P19623 | Spermidine synthase |
| RR-06 | AMD1 | P17707 | S-adenosylmethionine decarboxylase 1 |
| RR-06 | PDCD4 | Q53EL6 | Programmed cell death protein 4 |
| RR-06 | SRC | P12931 | Tyrosine-protein kinase SRC |
| RR-06 | ADORA2B | P29275 | Adenosine A2b receptor |
| RR-06 | MAPK1 | P28482 | MAP kinase ERK2 |
| RR-06 | SLC29A1 | Q99808 | Equilibrative nucleoside transporter 1 |
| RR-06 | PNP | P00491 | Purine nucleoside phosphorylase |
| RR-06 | SETD2 | Q9BYW2 | Histone-lysine N-methyltransferase SETD2 |
| RR-06 | CARM1 | Q86X55 | Histone-arginine methyltransferase CARM1 |
| RR-06 | PRMT1 | Q99873 | Protein-arginine N-methyltransferase 1 |
| RR-06 | DOT1L | Q8TEK3 | Histone-lysine N-methyltransferase, H3 lysine-79 specific |
| RR-06 | P2RY1 | P47900 | Purinergic receptor P2Y1 |
| RR-06 | P2RY11 | Q96G91 | Purinergic receptor P2Y11 |
| RR-06 | GRK1 | Q15835 | Rhodopsin kinase |
| RR-06 | SMS | P52788 | Spermine synthase |
| RR-06 | MTAP | Q13126 | S-methyl-5-thioadenosine phosphorylase (by homology) |
| RR-06 | FBP1 | P09467 | Fructose-1,6-bisphosphatase |
| RR-06 | FHIT | P49789 | Bis(5'-adenosyl)-triphosphatase |
| RR-06 | QARS | P47897 | Glutaminyl-tRNA synthetase |
| RR-06 | KMT2A | Q03164 | Histone-lysine N-methyltransferase MLL |
| RR-06 | SUV39H1 | O43463 | Histone-lysine N-methyltransferase SUV39H1 |
| RR-06 | DNMT1 | P26358 | DNA (cytosine-5)-methyltransferase 1 |
| RR-06 | INMT | O95050 | Indolethylamine N-methyltransferase |
| RR-06 | SMYD2 | Q9NRG4 | N-lysine methyltransferase SMYD2 |
| RR-06 | EZH2 | Q15910 | EZH2/SUZ12/EED/RBBP7/RBBP4 |
| RR-06 | EZH1 | Q92800 | Histone-lysine N-methyltransferase EZH1 |
| RR-06 | SETDB1 | Q15047 | Histone-lysine N-methyltransferase SETDB1 |
| RR-06 | PNMT | P11086 | Phenylethanolamine N-methyltransferase |
| RR-06 | DNMT3B | Q9UBC3 | DNA (cytosine-5)-methyltransferase 3B |
| RR-06 | CDA | P32320 | Cytidine deaminase |
| RR-06 | MAPKAPK2 | P49137 | MAP kinase-activated protein kinase 2 |
| RR-06 | HSPA1A | P0DMV8 | Heat shock 70 kDa protein 1 |
| RR-06 | CCND1 CDK4 | P24385 P11802 | Cyclin-dependent kinase 4/cyclin D1 |
| RR-06 | RARS | P54136 | Arginyl-tRNA synthetase |
| RR-06 | CA2 | P00918 | Carbonic anhydrase II |
| RR-06 | CA1 | P00915 | Carbonic anhydrase I |
| RR-06 | CA12 | O43570 | Carbonic anhydrase XII |
| RR-06 | CA9 | Q16790 | Carbonic anhydrase IX |
| RR-06 | PIM1 | P11309 | Serine/threonine-protein kinase PIM1 |
| RR-06 | P2RX1 | P51575 | P2X purinoceptor 1 |
| RR-06 | HSD17B1 | P14061 | Estradiol 17-beta-dehydrogenase 1 |
| RR-06 | GBA | P04062 | Beta-glucocerebrosidase |
| RR-06 | OGA | O60502 | Bifunctional protein NCOAT |
| RR-06 | MARS | P56192 | Methionyl-tRNA synthetase |
| RR-06 | CA7 | P43166 | Carbonic anhydrase VII |
| RR-06 | CA13 | Q8N1Q1 | Carbonic anhydrase XIII |
| RR-06 | PRMT7 | Q9NVM4 | Protein arginine N-methyltransferase 7 |
| RR-06 | GAA | P10253 | Lysosomal alpha-glucosidase |
| RR-06 | PARG | Q86W56 | Poly(ADP-ribose) glycohydrolase |
| RR-06 | GSK3B | P49841 | Glycogen synthase kinase-3 beta |
| RR-06 | FUCA1 | P04066 | Alpha-L-fucosidase I |
| RR-06 | IARS | P41252 | Isoleucyl-tRNA synthetase |
| RR-06 | CA14 | Q9ULX7 | Carbonic anhydrase XIV |
| RR-06 | SLC5A2 | P31639 | Sodium/glucose cotransporter 2 |
| RR-06 | SLC28A2 | O43868 | Sodium/nucleoside cotransporter 2 |
| RR-06 | DAO | P14920 | D-amino-acid oxidase |
| RR-07 | PRKCA | P17252 | Protein kinase C alpha |
| RR-07 | MMP2 | P08253 | Matrix metalloproteinase 2 |
| RR-07 | MMP12 | P39900 | Matrix metalloproteinase 12 |
| RR-07 | HSP90AA1 | P07900 | Heat shock protein HSP 90-alpha |
| RR-07 | APP | P05067 | Beta amyloid A4 protein |
| RR-07 | AKR1B1 | P15121 | Aldose reductase |
| RR-07 | AKR1B10 | O60218 | Aldo-keto reductase family 1 member B10 |
| RR-07 | AKR1C4 | [P17516](https://www.uniprot.org/uniprot/P17516) | Aldo-keto reductase family 1 member C4 |
| RR-07 | AMY1A; AMY1B; AMY1C | [P04745](https://www.uniprot.org/uniprot/P04745) | Alpha-amylase 1 |
| RR-07 | AMY2A | [P04746](https://www.uniprot.org/uniprot/P04746) | Pancreatic alpha-amylase |
| RR-07 | CA12 | O43570 | Carbonic anhydrase 12 |
| RR-07 | CA14 | [Q9ULX7](https://www.uniprot.org/uniprot/Q9ULX7) | Carbonic anhydrase 14 |
| RR-07 | CA5A | [P35218](https://www.uniprot.org/uniprot/P35218) | Carbonic anhydrase 5A, mitochondrial |
| RR-07 | CA5B | [Q9Y2D0](https://www.uniprot.org/uniprot/Q9Y2D0) | Carbonic anhydrase 5B, mitochondrial |
| RR-07 | CA6 | [P23280](https://www.uniprot.org/uniprot/P23280) | Carbonic anhydrase 6 |
| RR-07 | CA7 | P43166 | Carbonic anhydrase 7 |
| RR-07 | CA9 | [Q16790](https://www.uniprot.org/uniprot/Q16790) | Carbonic anhydrase 9 |
| RR-07 | FGF2 | [P09038](https://www.uniprot.org/uniprot/P09038) | Fibroblast growth factor 2 |
| RR-07 | FGF1 | [P05230](https://www.uniprot.org/uniprot/P05230) | Fibroblast growth factor 1 |
| RR-07 | SLC37A4 | [O43826](https://www.uniprot.org/uniprot/O43826) | Glucose-6-phosphate exchanger SLC37A4 |
| RR-07 | IL2 | [P60568](https://www.uniprot.org/uniprot/P60568) | Interleukin-2 |
| RR-07 | PRKCA | [P17252](https://www.uniprot.org/uniprot/P17252) | Protein kinase C alpha type |
| RR-07 | LGALS3 | [P17931](https://www.uniprot.org/uniprot/P17931) | Galectin-3 |
| RR-07 | LGALS4 | P56470 | Galectin-4 |
| RR-07 | LGALS8 | [O00214](https://www.uniprot.org/uniprot/O00214) | Galectin-8 |
| RR-07 | LGALS9 | [O00182](https://www.uniprot.org/uniprot/O00182) | Galectin-9 |
| RR-07 | NMUR2 | [Q9GZQ4](https://www.uniprot.org/uniprot/Q9GZQ4) | Neuromedin-U receptor 2 |
| RR-07 | P4HB | [P07237](https://www.uniprot.org/uniprot/P07237) | Protein disulfide-isomerase |
| RR-07 | HRAS | [P01112](https://www.uniprot.org/uniprot/P01112) | GTPase HRas |
| RR-07 | SLC28A3 | [Q9HAS3](https://www.uniprot.org/uniprot/Q9HAS3) | Solute carrier family 28 member 3 |
| RR-07 | SLC5A1 | [P13866](https://www.uniprot.org/uniprot/P13866) | Sodium/glucose cotransporter 1 |
| RR-07 | SLC5A2 | [P31639](https://www.uniprot.org/uniprot/P31639) | Sodium/glucose cotransporter 2 |
| RR-07 | SLC5A4 | [Q9NY91](https://www.uniprot.org/uniprot/Q9NY91) | Solute carrier family 5 member 4 |
| RR-07 | TTR | P02766 | Transthyretin |
| RR-07 | TYR | [P14679](https://www.uniprot.org/uniprot/P14679) | Tyrosinase |
| RR-07 | VEGFA | [P15692](https://www.uniprot.org/uniprot/P15692) | Vascular endothelial growth factor A |
| RR-08 | IL2 | P60568 | Interleukin-2 |
| RR-08 | STAT3 | P40763 | Signal transducer and activator of transcription 3 |
| RR-08 | PSEN2 | P49810 | Presenilin-2 |
| RR-08 | PSENEN | Q9NZ42 | Gamma-secretase subunit PEN-2 |
| RR-08 | NCSTN | Q92542 | Nicastrin |
| RR-08 | APH1A | Q96BI3 | Gamma-secretase subunit APH-1A |
| RR-08 | PSEN1 | P49768 | Presenilin-1 |
| RR-08 | APH1B | Q8WW43 | Gamma-secretase subunit APH-1B |
| RR-08 | PTAFR | P25105 | Platelet activating factor receptor |
| RR-08 | MET | P08581 | Hepatocyte growth factor receptor |
| RR-08 | S1PR3 | Q99500 | Sphingosine 1-phosphate receptor Edg-3 |
| RR-08 | S1PR1 | P21453 | Sphingosine 1-phosphate receptor Edg-1 |
| RR-08 | FLT1 | P17948 | Vascular endothelial growth factor receptor 1 |
| RR-08 | RBP4 | P02753 | Plasma retinol-binding protein |
| RR-08 | PPM1B | O75688 | Protein phosphatase 2C beta |
| RR-08 | PPP1CC | P36873 | Serine/threonine protein phosphatase PP1-gamma catalytic subunit |
| RR-08 | PPP2CA | P67775 | Serine/threonine protein phosphatase 2A, catalytic subunit, alpha isoform |
| RR-08 | PPP2R5A | Q15172 | Serine/threonine protein phosphatase 2A, 56 kDa regulatory subunit, alpha isoform |
| RR-08 | HSD11B2 | P80365 | 11-beta-hydroxysteroid dehydrogenase 2 |
| RR-08 | S1PR5 | Q9H228 | Sphingosine 1-phosphate receptor Edg-8 |
| RR-08 | S1PR4 | O95977 | Sphingosine 1-phosphate receptor Edg-6 |
| RR-08 | AMY2A | [P04746](https://www.uniprot.org/uniprot/P04746) | Pancreatic alpha-amylase |
| RR-08 | AR | [P10275](https://www.uniprot.org/uniprot/P10275) | Androgen receptor |
| RR-08 | SERPINA6 | [P08185](https://www.uniprot.org/uniprot/P08185) | Corticosteroid-binding globulin |
| RR-08 | UGCG | Q16739 | Ceramide glucosyltransferase |
| RR-08 | CYP17A1 | [P05093](https://www.uniprot.org/uniprot/P05093) | Steroid 17-alpha-hydroxylase/17,20 lyase |
| RR-08 | POLA1 | P09884 | DNA polymerase alpha catalytic subunit |
| RR-08 | EBP | [Q15392](https://www.uniprot.org/uniprot/Q15392) | 3-beta-hydroxysteroid-Delta(8),Delta(7)-isomerase |
| RR-08 | EPHA2 | P29317 | Ephrin type-A receptor 2 |
| RR-08 | EPHA5 | [P54756](https://www.uniprot.org/uniprot/P54756) | Ephrin type-A receptor 5 |
| RR-08 | EPHA7 | [Q15375](https://www.uniprot.org/uniprot/Q15375) | Ephrin type-A receptor 7 |
| RR-08 | ESR1 | [P03372](https://www.uniprot.org/uniprot/P03372) | Estrogen receptor |
| RR-08 | ESR2 | [Q92731](https://www.uniprot.org/uniprot/Q92731) | Estrogen receptor beta |
| RR-08 | FGF1 | [P05230](https://www.uniprot.org/uniprot/P05230) | Fibroblast growth factor 1 |
| RR-08 | FGF2 | [P09038](https://www.uniprot.org/uniprot/P09038) | Fibroblast growth factor 2 |
| RR-08 | G6PD | P11413 | Glucose-6-phosphate 1-dehydrogenase |
| RR-08 | GBA2 | [Q9HCG7](https://www.uniprot.org/uniprot/Q9HCG7) | Non-lysosomal glucosylceramidase |
| RR-08 | GPBAR1 | [Q8TDU6](https://www.uniprot.org/uniprot/Q8TDU6) | G-protein coupled bile acid receptor 1 |
| RR-08 | CDC25A | [P30304](https://www.uniprot.org/uniprot/P30304) | M-phase inducer phosphatase 1 |
| RR-08 | ABCC4 | [O15439](https://www.uniprot.org/uniprot/O15439) | Multidrug resistance-associated protein 4 |
| RR-08 | NPC1L1 | [Q9UHC9](https://www.uniprot.org/uniprot/Q9UHC9) | NPC1-like intracellular cholesterol transporter 1 |
| RR-08 | NR1H3 | [Q13133](https://www.uniprot.org/uniprot/Q13133) | Oxysterols receptor LXR-alpha |
| RR-08 | SLC10A1 | [Q14973](https://www.uniprot.org/uniprot/Q14973) | Sodium/bile acid cotransporter |
| RR-08 | RORA | [P35398](https://www.uniprot.org/uniprot/P35398) | Nuclear receptor ROR-alpha |
| RR-08 | SRD5A2 | [P31213](https://www.uniprot.org/uniprot/P31213) | 3-oxo-5-alpha-steroid 4-dehydrogenase 2 |
| RR-08 | SHBG | [P04278](https://www.uniprot.org/uniprot/P04278) | Sex hormone-binding globulin |
| RR-08 | ST3GAL1 | [Q11201](https://www.uniprot.org/uniprot/Q11201) | CMP-N-acetylneuraminate-beta-galactosamide-alpha-2,3-sialyltransferase 1 |
| RR-08 | SREBF2 | [Q12772](https://www.uniprot.org/uniprot/Q12772) | Sterol regulatory element-binding protein 2 |
| RR-08 | TYR | [P14679](https://www.uniprot.org/uniprot/P14679) | Tyrosinase |
| RR-09 | MMP2 | P08253 | Matrix metalloproteinase 2 |
| RR-09 | MMP12 | P39900 | Matrix metalloproteinase 12 |
| RR-09 | PRKCA | P17252 | Protein kinase C alpha |
| RR-09 | MMP13 | P45452 | Matrix metalloproteinase 13 |
| RR-09 | AKR1B1 | P15121 | Aldose reductase |
| RR-09 | MMP9 | P14780 | Matrix metalloproteinase 9 |
| RR-09 | AKR1B10 | [O60218](https://www.uniprot.org/uniprot/O60218) | Aldo-keto reductase family 1 member B10 |
| RR-09 | AKR1C4 | [P17516](https://www.uniprot.org/uniprot/P17516) | Aldo-keto reductase family 1 member C4 |
| RR-09 | AMY1A; AMY1B; AMY1C | [P04745](https://www.uniprot.org/uniprot/P04745) | Alpha-amylase 1 |
| RR-09 | AMY2A | [P04746](https://www.uniprot.org/uniprot/P04746) | Pancreatic alpha-amylase |
| RR-09 | CA14 | [Q9ULX7](https://www.uniprot.org/uniprot/Q9ULX7) | Carbonic anhydrase 14 |
| RR-09 | CA12 | O43570 | Carbonic anhydrase 12 |
| RR-09 | CA1 | [P00915](https://www.uniprot.org/uniprot/P00915) | Carbonic anhydrase 1 |
| RR-09 | CA5A | [P35218](https://www.uniprot.org/uniprot/P35218) | Carbonic anhydrase 5A, mitochondrial |
| RR-09 | CA5B | [Q9Y2D0](https://www.uniprot.org/uniprot/Q9Y2D0) | Carbonic anhydrase 5B, mitochondrial |
| RR-09 | CA6 | [P23280](https://www.uniprot.org/uniprot/P23280) | Carbonic anhydrase 6 |
| RR-09 | CA7 | P43166 | Carbonic anhydrase 7 |
| RR-09 | CA9 | [Q16790](https://www.uniprot.org/uniprot/Q16790) | Carbonic anhydrase 9 |
| RR-09 | ERAP1 | [Q9NZ08](https://www.uniprot.org/uniprot/Q9NZ08) | Endoplasmic reticulum aminopeptidase 1 |
| RR-09 | FGF1 | [P05230](https://www.uniprot.org/uniprot/P05230) | Fibroblast growth factor 1 |
| RR-09 | FGF2 | [P09038](https://www.uniprot.org/uniprot/P09038) | Fibroblast growth factor 2 |
| RR-09 | IL2 | P60568 | Interleukin-2 |
| RR-09 | LGALS3 | [P17931](https://www.uniprot.org/uniprot/P17931) | Galectin-3 |
| RR-09 | LGALS4 | P56470 | Galectin-4 |
| RR-09 | LGALS8 | [O00214](https://www.uniprot.org/uniprot/O00214) | Galectin-8 |
| RR-09 | LGALS9 | [O00182](https://www.uniprot.org/uniprot/O00182) | Galectin-9 |
| RR-09 | NMUR2 | [Q9GZQ4](https://www.uniprot.org/uniprot/Q9GZQ4) | Neuromedin-U receptor 2 |
| RR-09 | P4HB | [P07237](https://www.uniprot.org/uniprot/P07237) | Protein disulfide-isomerase |
| RR-09 | HRAS | [P01112](https://www.uniprot.org/uniprot/P01112) | GTPase HRas |
| RR-09 | SLC28A3 | [Q9HAS3](https://www.uniprot.org/uniprot/Q9HAS3) | Solute carrier family 28 member 3 |
| RR-09 | SLC5A1 | [P13866](https://www.uniprot.org/uniprot/P13866) | Sodium/glucose cotransporter 1 |
| RR-09 | SLC5A2 | [P31639](https://www.uniprot.org/uniprot/P31639) | Sodium/glucose cotransporter 2 |
| RR-09 | SLC5A4 | [Q9NY91](https://www.uniprot.org/uniprot/Q9NY91) | Solute carrier family 5 member 4 |
| RR-09 | TTR | P02766 | Transthyretin |
| RR-09 | TYR | [P14679](https://www.uniprot.org/uniprot/P14679) | Tyrosinase |
| RR-09 | VEGFA | [P15692](https://www.uniprot.org/uniprot/P15692) | Vascular endothelial growth factor A |
| RR-10 | PRKCA | P17252 | Protein kinase C alpha |
| RR-10 | MMP2 | P08253 | Matrix metalloproteinase 2 |
| RR-10 | MMP12 | P39900 | Matrix metalloproteinase 12 |
| RR-10 | CHIA | Q9BZP6 | Acidic mammalian chitinase |
| RR-10 | IMPDH1 | P20839 | Inosine-5'-monophosphate dehydrogenase 1 |
| RR-10 | HRAS | P01112 | Transforming protein p21/H-Ras-1 |
| RR-10 | HSP90AA1 | P07900 | Heat shock protein HSP 90-alpha |
| RR-10 | ADORA3 | P0DMS8 | Adenosine A3 receptor |
| RR-10 | LGALS7 | P47929 | P47929 |
| RR-10 | SLC29A1 | Q99808 | Equilibrative nucleoside transporter 1 |
| RR-10 | PARP1 | P09874 | Poly [ADP-ribose] polymerase-1 |
| RR-10 | APP | [P05067](https://www.uniprot.org/uniprot/P05067) | Amyloid-beta A4 protein |
| RR-10 | AKR1B10 | [O60218](https://www.uniprot.org/uniprot/O60218) | Aldo-keto reductase family 1 member B10 |
| RR-10 | AKR1C4 | [P17516](https://www.uniprot.org/uniprot/P17516) | Aldo-keto reductase family 1 member C4 |
| RR-10 | AMY1A; AMY1B; AMY1C | [P04745](https://www.uniprot.org/uniprot/P04745) | Alpha-amylase 1 |
| RR-10 | AMY2A | [P04746](https://www.uniprot.org/uniprot/P04746) | Pancreatic alpha-amylase |
| RR-10 | CA13 | Q8N1Q1 | Carbonic anhydrase 13 |
| RR-10 | CA12 | O43570 | Carbonic anhydrase 12 |
| RR-10 | CA14 | [Q9ULX7](https://www.uniprot.org/uniprot/Q9ULX7) | Carbonic anhydrase 14 |
| RR-10 | CA5A | [P35218](https://www.uniprot.org/uniprot/P35218) | Carbonic anhydrase 5A, mitochondrial |
| RR-10 | CA5B | [Q9Y2D0](https://www.uniprot.org/uniprot/Q9Y2D0) | Carbonic anhydrase 5B, mitochondrial |
| RR-10 | CA6 | [P23280](https://www.uniprot.org/uniprot/P23280) | Carbonic anhydrase 6 |
| RR-10 | CA7 | P43166 | Carbonic anhydrase 7 |
| RR-10 | CA9 | [Q16790](https://www.uniprot.org/uniprot/Q16790) | Carbonic anhydrase 9 |
| RR-10 | ODC1 | P11926 | Ornithine decarboxylase |
| RR-10 | ERAP1 | [Q9NZ08](https://www.uniprot.org/uniprot/Q9NZ08) | Endoplasmic reticulum aminopeptidase 1 |
| RR-10 | FGF1 | [P05230](https://www.uniprot.org/uniprot/P05230) | Fibroblast growth factor 1 |
| RR-10 | FGF2 | [P09038](https://www.uniprot.org/uniprot/P09038) | Fibroblast growth factor 2 |
| RR-10 | IL2 | P60568 | Interleukin-2 |
| RR-10 | LGALS3 | [P17931](https://www.uniprot.org/uniprot/P17931) | Galectin-3 |
| RR-10 | LGALS4 | P56470 | Galectin-4 |
| RR-10 | LGALS8 | [O00214](https://www.uniprot.org/uniprot/O00214) | Galectin-8 |
| RR-10 | LGALS9 | [O00182](https://www.uniprot.org/uniprot/O00182) | Galectin-9 |
| RR-10 | GLO1 | [Q04760](https://www.uniprot.org/uniprot/Q04760) | Lactoylglutathione lyase |
| RR-10 | SELL | [P14151](https://www.uniprot.org/uniprot/P14151) | L-selectin |
| RR-10 | SELP | [P16109](https://www.uniprot.org/uniprot/P16109) | P-selectin |
| RR-10 | IKBKG | [Q9Y6K9](https://www.uniprot.org/uniprot/Q9Y6K9) | NF-kappa-B essential modulator |
| RR-10 | NFE2L2 | [Q16236](https://www.uniprot.org/uniprot/Q16236) | Nuclear factor erythroid 2-related factor 2 |
| RR-10 | NFKB1 | [P19838](https://www.uniprot.org/uniprot/P19838) | Nuclear factor NF-kappa-B p105 subunit |
| RR-10 | NMUR2 | Q9GZQ4 | Neuromedin-U receptor 2 |
| RR-10 | P4HB | [P07237](https://www.uniprot.org/uniprot/P07237) | Protein disulfide-isomerase |
| RR-10 | SLC28A3 | [Q9HAS3](https://www.uniprot.org/uniprot/Q9HAS3) | Solute carrier family 28 member 3 |
| RR-10 | SLC5A1 | [P13866](https://www.uniprot.org/uniprot/P13866) | Sodium/glucose cotransporter 1 |
| RR-10 | SLC5A2 | [P31639](https://www.uniprot.org/uniprot/P31639) | Sodium/glucose cotransporter 2 |
| RR-10 | SLC5A4 | [Q9NY91](https://www.uniprot.org/uniprot/Q9NY91) | Solute carrier family 5 member 4 |
| RR-10 | MAPT | [P10636](https://www.uniprot.org/uniprot/P10636) | Microtubule-associated protein tau |
| RR-10 | TUBB1 | [Q9H4B7](https://www.uniprot.org/uniprot/Q9H4B7) | Tubulin beta-1 chain |
| RR-10 | TUBB3 | [Q13509](https://www.uniprot.org/uniprot/Q13509) | Tubulin beta-3 chain |
| RR-10 | TTR | P02766 | Transthyretin |
| RR-10 | TDP1 | [Q9NUW8](https://www.uniprot.org/uniprot/Q9NUW8) | Tyrosyl-DNA phosphodiesterase 1 |
| RR-10 | TYR | [P14679](https://www.uniprot.org/uniprot/P14679) | Tyrosinase |
| RR-10 | VEGFA | [P15692](https://www.uniprot.org/uniprot/P15692) | Vascular endothelial growth factor A |
| RR-11 | LGALS4 | P56470 | Galectin-4 |
| RR-11 | LGALS8 | [O00214](https://www.uniprot.org/uniprot/O00214) | Galectin-8 |
| RR-11 | FGF1 | [P05230](https://www.uniprot.org/uniprot/P05230) | Fibroblast growth factor 1 |
| RR-11 | FGF2 | [P09038](https://www.uniprot.org/uniprot/P09038) | Fibroblast growth factor 2 |
| RR-11 | IL2 | P60568 | Interleukin-2 |
| RR-11 | STAT3 | P40763 | Signal transducer and activator of transcription 3 |
| RR-11 | TYR | P14679 | Tyrosinase |
| RR-11 | SLC5A2 | P31639 | Sodium/glucose cotransporter 2 |
| RR-11 | SLC5A1 | P13866 | Sodium/glucose cotransporter 1 |
| RR-11 | ADORA1 | P30542 | Adenosine A1 receptor (by homology) |
| RR-11 | SLC29A1 | Q99808 | Equilibrative nucleoside transporter 1 |
| RR-11 | ADA | P00813 | Adenosine deaminase |
| RR-11 | MMP13 | P45452 | Matrix metalloproteinase 13 |
| RR-11 | MMP1 | P03956 | Matrix metalloproteinase 1 |
| RR-11 | MMP7 | P09237 | Matrix metalloproteinase 7 |
| RR-11 | MMP12 | P39900 | Matrix metalloproteinase 12 |
| RR-11 | MMP8 | P22894 | Matrix metalloproteinase 8 |
| RR-11 | CA2 | P00918 | Carbonic anhydrase II |
| RR-11 | CA1 | P00915 | Carbonic anhydrase I |
| RR-11 | CA12 | O43570 | Carbonic anhydrase XII |
| RR-11 | CA9 | Q16790 | Carbonic anhydrase IX |
| RR-11 | ADORA3 | P0DMS8 | Adenosine A3 receptor |
| RR-11 | HRAS | P01112 | Transforming protein p21/H-Ras-1 |
| RR-11 | CA14 | Q9ULX7 | Carbonic anhydrase XIV |
| RR-11 | PPM1B | O75688 | Protein phosphatase 2C beta |
| RR-11 | PPP1CC | P36873 | Serine/threonine protein phosphatase PP1-gamma catalytic subunit |
| RR-11 | PPP2CA | P67775 | Serine/threonine protein phosphatase 2A, catalytic subunit, alpha isoform |
| RR-11 | PPP2R5A | Q15172 | Serine/threonine protein phosphatase 2A, 56 kDa regulatory subunit, alpha isoform |
| RR-11 | ADORA2A | P29274 | Adenosine A2a receptor |
| RR-11 | PTPN1 | P18031 | Protein-tyrosine phosphatase 1B |
| RR-11 | OGA | O60502 | Bifunctional protein NCOAT |
| RR-11 | ADK | P55263 | Adenosine kinase |
| RR-11 | KDM3A | Q9Y4C1 | Lysine-specific demethylase 3A |
| RR-11 | KDM5B | Q9UGL1 | Lysine-specific demethylase 5B |
| RR-11 | IGFBP3 | P17936 | Insulin-like growth factor binding protein 3 |
| RR-11 | KDM4D | Q6B0I6 | Lysine-specific demethylase 4D |
| RR-11 | KDM4C | Q9H3R0 | Lysine-specific demethylase 4C |
| RR-11 | MAPK10 | P53779 | c-Jun N-terminal kinase 3 |
| RR-11 | MME | P08473 | Neprilysin |
| RR-11 | HSPA8 | P11142 | Heat shock cognate 71 kDa protein |
| RR-11 | SLC5A4 | Q9NY91 | Low affinity sodium-glucose cotransporter |
| RR-11 | FUCA1 | P04066 | Alpha-L-fucosidase I |
| RR-11 | HSPA5 | P11021 | 78 kDa glucose-regulated protein |
| RR-11 | HSP90AB1 | P08238 | Heat shock protein HSP 90-beta |
| RR-11 | SLC28A2 | O43868 | Sodium/nucleoside cotransporter 2 |
| RR-11 | EGFR | P00533 | Epidermal growth factor receptor erbB1 |
| RR-11 | MANBA | O00462 | Beta-mannosidase (by homology) |
| RR-11 | MAN2B1 | O00754 | Lysosomal alpha-mannosidase |
| RR-11 | EPHX2 | P34913 | Epoxide hydratase |
| RR-11 | PIM1 | P11309 | Serine/threonine-protein kinase PIM1 |
| RR-11 | JAK3 | P52333 | Tyrosine-protein kinase JAK3 |
| RR-11 | ADORA2B | P29275 | Adenosine A2b receptor |
| RR-11 | ITGB1 ITGA4 | P05556 P13612 | Integrin alpha-4/beta-1 |
| RR-11 | NOX4 | Q9NPH5 | NADPH oxidase 4 |
| RR-11 | NPC1L1 | Q9UHC9 | Niemann-Pick C1-like protein 1 |
| RR-11 | ANPEP | P15144 | Aminopeptidase N |
| RR-11 | MMP2 | P08253 | Matrix metalloproteinase 2 |
| RR-11 | MMP14 | P50281 | Matrix metalloproteinase 14 |
| RR-11 | LTA4H | P09960 | Leukotriene A4 hydrolase |
| RR-11 | ECE1 | P42892 | Endothelin-converting enzyme 1 |
| RR-11 | AKR1C3 | P42330 | Aldo-keto-reductase family 1 member C3 |
| RR-11 | CDK2 CCNA1 CCNA2 | P24941 P78396 P20248 | Cyclin-dependent kinase 2/cyclin A |
| RR-11 | HK2 | P52789 | Hexokinase type II |
| RR-11 | HK1 | P19367 | Hexokinase type I |
| RR-11 | AMPD3 | Q01432 | AMP deaminase 3 |
| RR-11 | ERN1 | O75460 | Serine/threonine-protein kinase/endoribonuclease IRE1 |
| RR-11 | FLT1 | P17948 | Vascular endothelial growth factor receptor 1 |
| RR-11 | KIT | P10721 | Stem cell growth factor receptor |
| RR-11 | CA7 | P43166 | Carbonic anhydrase VII |
| RR-11 | MMP3 | P08254 | Matrix metalloproteinase 3 |
| RR-11 | CA6 | P23280 | Carbonic anhydrase VI |
| RR-11 | ADAM17 | P78536 | ADAM17 |
| RR-11 | CA4 | P22748 | Carbonic anhydrase IV |
| RR-11 | CA13 | Q8N1Q1 | Carbonic anhydrase XIII |
| RR-11 | CA5A | P35218 | Carbonic anhydrase VA |
| RR-11 | CXCR2 | P25025 | Interleukin-8 receptor B |
| RR-11 | AGTR1 | P30556 | Type-1 angiotensin II receptor |
| RR-12 | LGALS4 | P56470 | Galectin-4 |
| RR-12 | LGALS8 | [O00214](https://www.uniprot.org/uniprot/O00214) | Galectin-8 |
| RR-12 | LANCL2 | Q9NS86 | LanC-like protein 2 |
| RR-12 | FGF1 | [P05230](https://www.uniprot.org/uniprot/P05230) | Fibroblast growth factor 1 |
| RR-12 | FGF2 | [P09038](https://www.uniprot.org/uniprot/P09038) | Fibroblast growth factor 2 |
| RR-12 | LGALS3 | [P17931](https://www.uniprot.org/uniprot/P17931) | Galectin-3 |
| RR-12 | VEGFA | [P15692](https://www.uniprot.org/uniprot/P15692) | Vascular endothelial growth factor A |
| RR-12 | IL2 | P60568 | Interleukin-2 |
| RR-12 | LGALS9 | [O00182](https://www.uniprot.org/uniprot/O00182) | Galectin-9 |
| RR-12 | SELP | [P16109](https://www.uniprot.org/uniprot/P16109) | P-selectin |
| RR-12 | GLB1 | P16278 | Beta-galactosidase |
| RR-13 | AKR1B10 | O60218 | Aldo-keto reductase family 1 member B10 |
| RR-13 | CD81 | P60033 | CD81 antigen |
| RR-13 | HSD11B2 | P80365 | Corticosteroid 11-beta-dehydrogenase isozyme 2 |
| RR-13 | POLB | P06746 | DNA polymerase beta |
| RR-13 | CES2 | O00748 | Cocaine esterase |
| RR-13 | PLCG1 | P19174 | 1-phosphatidylinositol 4,5-bisphosphate phosphodiesterase gamma-1 |
| RR-13 | ACP1 | P24666 | Low molecular weight phosphotyrosine protein phosphatase |
| RR-13 | PTPN1 | P18031 | Tyrosine-protein phosphatase non-receptor type 1 |
| RR-13 | PTPN2 | P17706 | Tyrosine-protein phosphatase non-receptor type 2 |
| RR-13 | PTPN6 | [P29350](https://www.uniprot.org/uniprot/P29350) | Tyrosine-protein phosphatase non-receptor type 6 |
| RR-13 | F3 | [P13726](https://www.uniprot.org/uniprot/P13726) | Tissue factor |
| RR-13 | BCL2L1 | Q07817 | Apoptosis regulator Bcl-X |
| RR-13 | IKBKB | O14920 | Inhibitor of nuclear factor kappa B kinase beta subunit |
| RR-14 | ABCB11 | O95342 | Bile salt export pump |
| RR-14 | CA1 | P00915 | Carbonic anhydrase 1 |
| RR-14 | CA2 | P00918 | Carbonic anhydrase 2 |
| RR-14 | CD4 | P01730 | T-cell surface glycoprotein CD4 |
| RR-14 | CDC45 | O75419 | Cell division control protein 45 homolog |
| RR-14 | CYP24A1 | Q07973 | 1,25-dihydroxyvitamin D(3) 24-hydroxylase, mitochondrial |
| RR-14 | CYP27B1 | O15528 | 25-hydroxyvitamin D-1 alpha hydroxylase, mitochondrial |
| RR-14 | POLA1 | P09884 | DNA polymerase alpha catalytic subunit |
| RR-14 | EBP | Q15125 | 3-beta-hydroxysteroid-Delta(8),Delta(7)-isomerase |
| RR-14 | ENPP2 | Q13822 | Ectonucleotide pyrophosphatase/phosphodiesterase family member 2 |
| RR-14 | EPHA1 | P21709 | Ephrin type-A receptor 1 |
| RR-14 | EPHA2 | P29317 | Ephrin type-A receptor 2 |
| RR-14 | EPHA4 | P54764 | Ephrin type-A receptor 4 |
| RR-14 | EPHA5 | P54756 | Ephrin type-A receptor 5 |
| RR-14 | EPHA6 | Q9UF33 | Ephrin type-A receptor 6 |
| RR-14 | EPHA7 | Q15375 | Ephrin type-A receptor 7 |
| RR-14 | EPHA8 | P29322 | Ephrin type-A receptor 8 |
| RR-14 | EPHB1 | P54762 | Ephrin type-B receptor 1 |
| RR-14 | EPHB2 | P29323 | Ephrin type-B receptor 2 |
| RR-14 | EPHB3 | P54753 | Ephrin type-B receptor 3 |
| RR-14 | GBA2 | Q9HCG7 | Non-lysosomal glucosylceramidase |
| RR-14 | GPBAR1 | Q8TDU6 | G-protein coupled bile acid receptor 1 |
| RR-14 | ABCC4 | O15439 | Multidrug resistance-associated protein 4 |
| RR-14 | NR1H4 | Q96RI1 | Bile acid receptor |
| RR-14 | SLC10A2 | Q12908 | Ileal sodium/bile acid cotransporter |
| RR-14 | SLC10A1 | Q14973 | Sodium/bile acid cotransporter |
| RR-14 | ATIC | P31939 | Bifunctional purine biosynthesis protein PURH |
| RR-14 | SRD5A1 | P18405 | 3-oxo-5-alpha-steroid 4-dehydrogenase 1 |
| RR-14 | SRD5A2 | P31213 | 3-oxo-5-alpha-steroid 4-dehydrogenase 2 |
| RR-14 | SHH | Q15465 | Sonic hedgehog protein |
| RR-14 | ST3GAL1 | Q11201 | CMP-N-acetylneuraminate-beta-galactosamide-alpha-2,3-sialyltransferase 1 |
| RR-14 | GC | P02774 | Vitamin D-binding protein |
| RR-14 | AR | P10275 | Androgen Receptor |
| RR-14 | HMGCR | P04035 | HMG-CoA reductase |
| RR-14 | CYP51A1 | Q16850 | Cytochrome P450 51 (by homology) |
| RR-14 | NPC1L1 | Q9UHC9 | Niemann-Pick C1-like protein 1 |
| RR-14 | NR1H3 | Q13133 | LXR-alpha |
| RR-14 | CYP19A1 | P11511 | Cytochrome P450 19A1 |
| RR-14 | CYP17A1 | P05093 | Cytochrome P450 17A1 |
| RR-14 | RORC | P51449 | Nuclear receptor ROR-gamma |
| RR-14 | ESR1 | P03372 | Estrogen receptor alpha |
| RR-14 | ESR2 | Q92731 | Estrogen receptor beta |
| RR-14 | SREBF2 | Q12772 | Sterol regulatory element-binding protein 2 |
| RR-14 | SHBG | P04278 | Testis-specific androgen-binding protein |
| RR-14 | SLC6A2 | P23975 | Norepinephrine transporter |
| RR-14 | CYP2C19 | P33261 | Cytochrome P450 2C19 |
| RR-14 | RORA | P35398 | Nuclear receptor ROR-alpha |
| RR-14 | PTPN1 | P18031 | Protein-tyrosine phosphatase 1B |
| RR-14 | BCHE | P06276 | Butyrylcholinesterase |
| RR-14 | SERPINA6 | P08185 | Corticosteroid binding globulin |
| RR-14 | SLC6A4 | P31645 | Serotonin transporter |
| RR-14 | CHRM2 | P08172 | Muscarinic acetylcholine receptor M2 |
| RR-14 | VDR | P11473 | Vitamin D receptor |
| RR-14 | ACHE | P22303 | Acetylcholinesterase |
| RR-14 | G6PD | P11413 | Glucose-6-phosphate 1-dehydrogenase |
| RR-14 | NR1H2 | P55055 | LXR-beta |
| RR-14 | GLRA1 | P23415 | Glycine receptor subunit alpha-1 |
| RR-14 | CES2 | O00748 | Carboxylesterase 2 |
| RR-14 | PTGER1 | P34995 | Prostanoid EP1 receptor (by homology) |
| RR-14 | PTGER2 | P43116 | Prostanoid EP2 receptor (by homology) |
| RR-14 | HSD11B1 | P28845 | 11-beta-hydroxysteroid dehydrogenase 1 |
| RR-14 | PTGES | O14684 | Prostaglandin E synthase |
| RR-14 | CDC25A | P30304 | Dual specificity phosphatase Cdc25A |
| RR-14 | PPARA | Q07869 | Peroxisome proliferator-activated receptor alpha |
| RR-14 | PPARD | Q03181 | Peroxisome proliferator-activated receptor delta |
| RR-14 | DHCR7 | Q9UBM7 | Anti-estrogen binding site (AEBS) (by homology) |
| RR-14 | SQLE | Q14534 | Squalene monooxygenase |
| RR-14 | PTPN6 | P29350 | Protein-tyrosine phosphatase 1C |
| RR-14 | NR1I3 | Q14994 | Nuclear receptor subfamily 1 group I member 3 |
| RR-14 | FDFT1 | P37268 | Squalene synthetase (by homology) |
| RR-14 | SIGMAR1 | Q99720 | Sigma opioid receptor |
| RR-14 | NOS2 | P35228 | Nitric oxide synthase, inducible (by homology) |
| RR-14 | NR3C1 | P04150 | Glucocorticoid receptor |
| RR-14 | PPARG | P37231 | Peroxisome proliferator-activated receptor gamma |
| RR-14 | CDC25B | P30305 | Dual specificity phosphatase Cdc25B |
| RR-14 | UGT2B7 | P16662 | UDP-glucuronosyltransferase 2B7 |
| RR-14 | HSD11B2 | P80365 | 11-beta-hydroxysteroid dehydrogenase 2 |
| RR-14 | POLB | P06746 | DNA polymerase beta (by homology) |
| PC-01 | POLB | P06746 | DNA polymerase beta |
| PC-01 | PTPN2 | P17706 | Tyrosine-protein phosphatase non-receptor type 2 |
| PC-01 | PTPN1 | P18031 | Tyrosine-protein phosphatase non-receptor type 1 |
| PC-01 | AKR1B10 | O60218 | Aldo-keto reductase family 1 member B10 |
| PC-01 | AKR1B1 | P15121 | Aldose reductase |
| PC-01 | AKR1B15 | C9JRZ8 | Aldo-keto reductase family 1 member B15 |
| PC-01 | AKR1A1 | P14550 | Alcohol dehydrogenase [NADP(+)] |
| PC-01 | AKR1E2 | Q96JD6 | 1,5-anhydro-D-fructose reductase |
| PC-01 | HSD11B1 | P28845 | Corticosteroid 11-beta-dehydrogenase isozyme 1 |
| PC-01 | HSD11B1L | Q7Z5J1 | Hydroxysteroid 11-beta-dehydrogenase 1-like protein |
| PC-01 | PLA2G1B | P04054 | Phospholipase A2 |
| PC-01 | PTPRF | P10586 | Receptor-type tyrosine-protein phosphatase F |
| PC-01 | ACP1 | P24666 | Low molecular weight phosphotyrosine protein phosphatase |
| PC-01 | PTPRD | P23468 | Receptor-type tyrosine-protein phosphatase delta |
| PC-01 | PTPRS | Q13332 | Receptor-type tyrosine-protein phosphatase S |
| PC-01 | TYR | P14679 | Tyrosinase |
| PC-01 | [PTGES](http://zinc15.docking.org/genes/PTGES) | O14684 | Prostaglandin E synthase |
| PC-01 | [PIN1](http://zinc15.docking.org/genes/PIN1) | Q13526 | Peptidyl-prolyl cis-trans isomerase NIMA-interacting 1 |
| PC-01 | [POLA1](http://zinc15.docking.org/genes/POLA1) | P09884 | DNA polymerase alpha catalytic subunit |
| PC-02 | AR | P10275 | Androgen receptor |
| PC-02 | TDP1 | Q9NUW8 | Tyrosyl-DNA phosphodiesterase 1 |
| PC-02 | NR1H2 | P55055 | Oxysterols receptor LXR-beta |
| PC-02 | NR1H3 | Q13133 | Oxysterols receptor LXR-alpha |
| PC-02 | HMGCR | P04035 | 3-hydroxy-3-methylglutaryl-coenzyme A reductase |
| PC-02 | CYP51A1 | Q16850 | Lanosterol 14-alpha demethylase |
| PC-02 | SLC6A2 | P23975 | Sodium-dependent noradrenaline transporter |
| PC-02 | SLC6A3 | Q01959 | Sodium-dependent dopamine transporter |
| PC-02 | SLC6A4 | P31645 | Sodium-dependent serotonin transporter |
| PC-02 | CYP19A1 | P11511 | Cytochrome P450 19A1 |
| PC-02 | CHRM2 | P08172 | Muscarinic acetylcholine receptor M2 |
| PC-02 | CHRM1 | P11229 | Muscarinic acetylcholine receptor M1 |
| PC-02 | CHRM4 | P08173 | Muscarinic acetylcholine receptor M4 |
| PC-02 | CHRM5 | P08912 | Muscarinic acetylcholine receptor M5 |
| PC-02 | CHRM3 | P20309 | Muscarinic acetylcholine receptor M3 |
| PC-02 | [GPBAR1](http://zinc15.docking.org/genes/GPBAR1) | Q8TDU6 | G-protein coupled bile acid receptor 1 |
| PC-02 | [SLC10A2](http://zinc15.docking.org/genes/SLC10A2) | Q12908 | Ileal sodium/bile acid cotransporter |
| PC-02 | [VDR](http://zinc15.docking.org/genes/VDR) | P11473 | Vitamin D3 receptor |
| PC-02 | [ABCB11](http://zinc15.docking.org/genes/ABCB11) | O95342 | Bile salt export pump |
| PC-02 | [CYP24A1](http://zinc15.docking.org/genes/CYP24A1) | Q07973 | 1,25-dihydroxyvitamin D(3) 24-hydroxylase, mitochondrial |
| PC-02 | [NR1H4](http://zinc15.docking.org/genes/NR1H4) | Q96RI1 | Bile acid receptor |
| PC-02 | [NPC1L1](http://zinc15.docking.org/genes/NPC1L1) | Q9UHC9 | Niemann-Pick C1-like protein 1 |
| PC-02 | [SLC10A1](http://zinc15.docking.org/genes/SLC10A1) | Q14973 | Sodium/bile acid cotransporter |
| PC-03 | NOS3 | P29474 | Nitric oxide synthase, endothelial |
| PC-03 | NOS3 | P29475 | Nitric oxide synthase, brain |
| PC-03 | NOS2 | P35228 | Nitric oxide synthase, inducible |
| PC-03 | AR | P10275 | Androgen receptor |
| PC-03 | NR1H2 | P55055 | Oxysterols receptor LXR-beta |
| PC-03 | NR1H3 | Q13133 | Oxysterols receptor LXR-alpha |
| PC-03 | PTPN1 | P18031 | Tyrosine-protein phosphatase non-receptor type 1 |
| PC-03 | PTPN2 | P17706 | Tyrosine-protein phosphatase non-receptor type 2 |
| PC-03 | MAPT | P10636 | Microtubule-associated protein tau |
| PC-03 | VDR | P11473 | Vitamin D3 receptor |
| PC-03 | CHRM2 | P08172 | Muscarinic acetylcholine receptor M2 |
| PC-03 | CHRM4 | P08173 | Muscarinic acetylcholine receptor M4 |
| PC-03 | CHRM5 | P08912 | Muscarinic acetylcholine receptor M5 |
| PC-03 | CHRM1 | P11229 | Muscarinic acetylcholine receptor M1 |
| PC-03 | CHRM3 | P20309 | Muscarinic acetylcholine receptor M3 |
| PC-03 | CASP3 | P42574 | Caspase-3 |
| PC-04 | ACP1 | P24666 | Low molecular weight phosphotyrosine protein phosphatase |
| PC-04 | ADORA3 | P0DMS8 | Adenosine A3 receptor |
| PC-04 | ADRA2A | P08913 | Alpha-2a adrenergic receptor |
| PC-04 | ADRA2C | P18825 | Adrenergic receptor alpha-2 |
| PC-04 | ADRB2 | P07550 | Adrenergic receptor beta |
| PC-04 | ADRB3 | P13945 | Beta-3 adrenergic receptor |
| PC-04 | AGTR1 | P30556 | Type-1 angiotensin II receptor |
| PC-04 | AKR1B10 | O60218 | Aldo-keto reductase family 1 member B10 |
| PC-04 | ALOX12 | P18054 | Arachidonate 12-lipoxygenase |
| PC-04 | ALOX5 | P09917 | Arachidonate 5-lipoxygenase |
| PC-04 | ALOX5AP | P20292 | 5-lipoxygenase activating protein |
| PC-04 | AR | P10275 | Androgen Receptor |
| PC-04 | BACE1 | P56817 | Beta-secretase 1 |
| PC-04 | BCHE | P06276 | Butyrylcholinesterase |
| PC-04 | CCKBR | P32239 | Cholecystokinin B receptor |
| PC-04 | CCR1 | P32246 | C-C chemokine receptor type 1 |
| PC-04 | CD81 | P60033 | CD81 antigen |
| PC-04 | CDC25A | P30304 | Dual specificity phosphatase Cdc25A |
| PC-04 | CDC25B | P30305 | Dual specificity phosphatase Cdc25B |
| PC-04 | CES2 | O00748 | Carboxylesterase 2 |
| PC-04 | CHRM1 | P11229 | Muscarinic acetylcholine receptor M1 |
| PC-04 | CHRM3 | P20309 | Muscarinic acetylcholine receptor M3 |
| PC-04 | CYP17A1 | P05093 | Cytochrome P450 17A1 |
| PC-04 | CYP19A1 | P11511 | Cytochrome P450 19A1 |
| PC-04 | CYP51A1 | Q16850 | Cytochrome P450 51 (by homology) |
| PC-04 | CYSLTR1 | Q9Y271 | Cysteinyl leukotriene receptor 1 |
| PC-04 | DRD3 | P35462 | Dopamine D3 receptor |
| PC-04 | ECE1 | P42892 | Endothelin-converting enzyme 1 |
| PC-04 | EDNRA | P25101 | Endothelin receptor ET-A |
| PC-04 | EDNRB | P24530 | Endothelin receptor ET-B |
| PC-04 | EGFR | P00533 | Epidermal growth factor receptor erbB1 |
| PC-04 | ESR1 | P03372 | Estrogen receptor alpha |
| PC-04 | ESR2 | Q92731 | Estrogen receptor beta |
| PC-04 | FABP1 | P07148 | Fatty acid-binding protein, liver |
| PC-04 | FNTA FNTB | P49354 P49356 | Protein farnesyltransferase |
| PC-04 | FYN | P06241 | Tyrosine-protein kinase FYN |
| PC-04 | G6PD | P11413 | Glucose-6-phosphate 1-dehydrogenase |
| PC-04 | GPR17 | Q13304 | Uracil nucleotide/cysteinyl leukotriene receptor |
| PC-04 | HAO1 | Q9UJM8 | Hydroxyacid oxidase 1 |
| PC-04 | HMGCR | P04035 | HMG-CoA reductase |
| PC-04 | HSD11B1 | P28845 | 11-beta-hydroxysteroid dehydrogenase 1 |
| PC-04 | ITGA2B ITGB3 | P08514 P05106 | Integrin alpha-IIb/beta-3 |
| PC-04 | ITGAV ITGB3 | P06756 P05106 | Integrin alpha-V/beta-3 |
| PC-04 | ITGB1 ITGA4 | P05556 P13612 | Integrin alpha-4/beta-1 |
| PC-04 | ITGB5 ITGAV | P18084 P06756 | Integrin alpha-V/beta-5 |
| PC-04 | MDM2 | Q00987 | p53-binding protein Mdm-2 |
| PC-04 | MMP2 | P08253 | Matrix metalloproteinase 2 |
| PC-04 | MMP3 | P08254 | Matrix metalloproteinase 3 |
| PC-04 | NOS2 | P35228 | Nitric oxide synthase, inducible |
| PC-04 | NPC1L1 | Q9UHC9 | Niemann-Pick C1-like protein 1 |
| PC-04 | NR1H3 | Q13133 | LXR-alpha |
| PC-04 | NR3C1 | P04150 | Glucocorticoid receptor |
| PC-04 | NTSR1 | P30989 | Neurotensin receptor 1 |
| PC-04 | OGFRL1 | Q5TC84 | Opioid growth factor receptor-like protein 1 |
| PC-04 | OPRD1 | P41143 | Delta opioid receptor |
| PC-04 | PGC | P20142 | Pepsinogen C (by homology) |
| PC-04 | PGR | P06401 | Progesterone receptor |
| PC-04 | PIM1 | P11309 | Serine/threonine-protein kinase PIM1 |
| PC-04 | POLB | P06746 | DNA polymerase beta (by homology) |
| PC-04 | PPARA | Q07869 | Peroxisome proliferator-activated receptor alpha |
| PC-04 | PPARD | Q03181 | Peroxisome proliferator-activated receptor delta |
| PC-04 | PPARG | P37231 | Peroxisome proliferator-activated receptor gamma |
| PC-04 | PREP | P48147 | Prolyl endopeptidase |
| PC-04 | PTGDR | Q13258 | Prostanoid DP receptor |
| PC-04 | PTGDR2 | Q9Y5Y4 | G protein-coupled receptor 44 |
| PC-04 | PTGER1 | P34995 | Prostanoid EP1 receptor |
| PC-04 | PTGER2 | P43116 | Prostanoid EP2 receptor (by homology) |
| PC-04 | PTGER4 | P35408 | Prostanoid EP4 receptor (by homology) |
| PC-04 | PTGES | O14684 | Prostaglandin E synthase |
| PC-04 | PTPN1 | P18031 | Protein-tyrosine phosphatase 1B |
| PC-04 | PTPN2 | P17706 | T-cell protein-tyrosine phosphatase |
| PC-04 | PTPN6 | P29350 | Protein-tyrosine phosphatase 1C |
| PC-04 | PTPRF | P10586 | Receptor-type tyrosine-protein phosphatase F (LAR) |
| PC-04 | RORC | P51449 | Nuclear receptor ROR-gamma |
| PC-04 | S1PR2 | O95136 | Sphingosine 1-phosphate receptor Edg-5 |
| PC-04 | SERPINA6 | P08185 | Corticosteroid binding globulin |
| PC-04 | SHBG | P04278 | Testis-specific androgen-binding protein |
| PC-04 | SLC10A2 | Q12908 | Ileal bile acid transporter |
| PC-04 | SLC6A3 | Q01959 | Dopamine transporter |
| PC-04 | SRD5A2 | P31213 | Steroid 5-alpha-reductase 2 |
| PC-04 | TNF | P01375 | TNF-alpha |
| PC-04 | TOP2A | P11388 | DNA topoisomerase II alpha |
| PC-04 | VDR | P11473 | Vitamin D receptor |
| PC-04 | TOP2A | P11388 | DNA topoisomerase 2-alpha |
| PC-04 | PIN1 | Q13526 | Peptidyl-prolyl cis-trans isomerase NIMA-interacting 1 |
| PC-05 | POLB | P06746 | DNA polymerase beta |
| PC-05 | PTPN2 | P17706 | Tyrosine-protein phosphatase non-receptor type 2 |
| PC-05 | PTPN1 | P18031 | Tyrosine-protein phosphatase non-receptor type 1 |
| PC-05 | AKR1B10 | O60218 | Aldo-keto reductase family 1 member B10 |
| PC-05 | AKR1B1 | P15121 | Aldose reductase |
| PC-05 | AKR1B15 | C9JRZ8 | Aldo-keto reductase family 1 member B15 |
| PC-05 | AKR1A1 | P14550 | Alcohol dehydrogenase [NADP(+)] |
| PC-05 | AKR1E2 | Q96JD6 | 1,5-anhydro-D-fructose reductase |
| PC-05 | HSD11B1 | P28845 | Corticosteroid 11-beta-dehydrogenase isozyme 1 |
| PC-05 | HSD11B1L | Q7Z5J1 | Hydroxysteroid 11-beta-dehydrogenase 1-like protein |
| PC-05 | PTPN6 | P29350 | Tyrosine-protein phosphatase non-receptor type 6 |
| PC-05 | PTPN11 | Q06124 | Tyrosine-protein phosphatase non-receptor type 11 |
| PC-05 | TDP1 | Q9NUW8 | Tyrosyl-DNA phosphodiesterase 1 |
| PC-05 | CDC25A | P30304 | M-phase inducer phosphatase 1 |
| PC-05 | CDC25B | P30305 | M-phase inducer phosphatase 2 |
| PC-05 | [PIN1](http://zinc15.docking.org/genes/PIN1) | Q13526 | Peptidyl-prolyl cis-trans isomerase NIMA-interacting 1 |
| PC-05 | [POLA1](http://zinc15.docking.org/genes/POLA1) | P09884 | DNA polymerase alpha catalytic subunit |
| PC-06 | PTPN2 | P17706 | Tyrosine-protein phosphatase non-receptor type 2 |
| PC-06 | PTPN1 | P18031 | Tyrosine-protein phosphatase non-receptor type 1 |
| PC-06 | FNTA&FNTB | P49354&P49356 | Complex |
| PC-06 | HSD11B1 | P28845 | Corticosteroid 11-beta-dehydrogenase isozyme 1 |
| PC-06 | MAPT | P10636 | Microtubule-associated protein tau |
| PC-06 | F2 | P00734 | Activation peptide fragment 1 |
| PC-06 | NR3C1 | P04150 | Glucocorticoid receptor |
| PC-06 | NR3C2 | P08235 | Mineralocorticoid receptor |
| PC-06 | PTGS1 | P23219 | Prostaglandin G/H synthase 1 |
| PC-06 | PTGS2 | P35354 | Prostaglandin G/H synthase 2 |
| PC-06 | NOS2 | P35228 | Nitric oxide synthase , inducible |
| PC-06 | NOS3 | P29474 | Nitric oxide synthase , endothelial |
| PC-06 | NOS1 | P29475 | Nitric oxide synthase , brain |
| PC-06 | PTAFR | P25105 | Platelet-activating factor receptor |
| PC-06 | HSD17B2 | P37059 | Estradiol 17-beta-dehydrogenase 2 |
| PC-06 | SLC2A4 | P14672 | Solute carrier family 2, facilitated glucose transporter member 4 |
| PC-06 | [TAS2R31](http://zinc15.docking.org/genes/TAS2R31) | P59538 | Taste receptor type 2 member 31 |
| PC-06 | [HSD11B2](http://zinc15.docking.org/genes/HSD11B2) | P80365 | Corticosteroid 11-beta-dehydrogenase isozyme 2 |
| PC-06 | PIN1 | Q13526 | Peptidyl-prolyl cis-trans isomerase NIMA-interacting 1 |
| PC-07 | PTPN2 | P17706 | Tyrosine-protein phosphatase non-receptor type 2 |
| PC-07 | PTPN1 | P18031 | Tyrosine-protein phosphatase non-receptor type 1 |
| PC-07 | TDP1 | Q9NUW8 | Tyrosyl-DNA phosphodiesterase 1 |
| PC-07 | MAPT | P10636 | Microtubule-associated protein tau |
| PC-07 | NR3C1 | P04150 | Glucocorticoid receptor |
| PC-07 | NR3C2 | P08235 | Mineralocorticoid receptor |
| PC-07 | NR1H2 | P55055 | Oxysterols receptor LXR-beta |
| PC-07 | NR1H3 | Q13133 | Oxysterols receptor LXR-alpha |
| PC-07 | FNTA&FNTB | P49354&P49356 | Complex |
| PC-07 | TOP2A | P11388 | DNA topoisomerase 2-alpha |
| PC-07 | TOP2B | Q02880 | DNA topoisomerase 2-beta |
| PC-07 | PPARG | P37231 | Peroxisome proliferator-activated receptor gamma |
| PC-07 | PPARD | Q03181 | Peroxisome proliferator-activated receptor delta |
| PC-07 | PPARA | Q07869 | Peroxisome proliferator-activated receptor alpha |
| PC-07 | HMGCR | P04035 | 3-hydroxy-3-methylglutaryl-coenzyme A reductase |
| PC-08 | PTPN2 | P17706 | Tyrosine-protein phosphatase non-receptor type 2 |
| PC-08 | PTPN1 | P18031 | Tyrosine-protein phosphatase non-receptor type 1 |
| PC-08 | TOP2A | P11388 | DNA topoisomerase 2-alpha |
| PC-08 | TOP2B | Q02880 | DNA topoisomerase 2-beta |
| PC-08 | FNTA&FNTB | P49354&P49356 | Complex |
| PC-08 | MAPT | P10636 | Microtubule-associated protein tau |
| PC-08 | HMGCR | P04035 | 3-hydroxy-3-methylglutaryl-coenzyme A reductase |
| PC-08 | TDP1 | Q9NUW8 | Tyrosyl-DNA phosphodiesterase 1 |
| PC-08 | PTPRF | P10586 | Receptor-type tyrosine-protein phosphatase F |
| PC-08 | PTPRD | P23468 | Receptor-type tyrosine-protein phosphatase delta |
| PC-08 | PTPRS | Q13332 | Receptor-type tyrosine-protein phosphatase S |
| PC-08 | PTGER2 | P43116 | Prostaglandin E2 receptor EP2 subtype |
| PC-08 | PPARG | P37231 | Peroxisome proliferator-activated receptor gamma |
| PC-08 | PPARD | Q03181 | Peroxisome proliferator-activated receptor delta |
| PC-08 | PPARA | Q07869 | Peroxisome proliferator-activated receptor alpha |
| PC-09 | TOP2A | P11388 | DNA topoisomerase 2-alpha |
| PC-09 | TOP2B | Q02880 | DNA topoisomerase 2-beta |
| PC-09 | NR1H2 | P55055 | Oxysterols receptor LXR-beta |
| PC-09 | NR1H3 | Q13133 | Oxysterols receptor LXR-alpha |
| PC-09 | NR3C1 | P04150 | Glucocorticoid receptor |
| PC-09 | NR3C2 | P08235 | Mineralocorticoid receptor |
| PC-09 | PTPN2 | P17706 | Tyrosine-protein phosphatase non-receptor type 2 |
| PC-09 | PTPN1 | P18031 | Tyrosine-protein phosphatase non-receptor type 1 |
| PC-09 | POLB | P06746 | DNA polymerase beta |
| PC-09 | TDP1 | Q9NUW8 | Tyrosyl-DNA phosphodiesterase 1 |
| PC-09 | ALOX5 | P09917 | Arachidonate 5-lipoxygenase |
| PC-09 | ALOX15 | P16050 | Arachidonate 15-lipoxygenase |
| PC-09 | ALOX12 | P18054 | Arachidonate 12-lipoxygenase |
| PC-09 | ALOX15B | O15296 | Arachidonate 15-lipoxygenase B |
| PC-09 | ALOX12B | O75342 | Arachidonate 12-lipoxygenase |
| PC-10 | MAPT | P10636 | Microtubule-associated protein tau |
| PC-10 | TDP1 | Q9NUW8 | Tyrosyl-DNA phosphodiesterase 1 |
| PC-10 | POLB | P06746 | DNA polymerase beta |
| PC-10 | AKR1B10 | O60218 | Aldo-keto reductase family 1 member B10 |
| PC-10 | PLA2G1B | P04054 | Phospholipase A2 |
| PC-10 | PTPN2 | P17706 | Tyrosine-protein phosphatase non-receptor type 2 |
| PC-10 | PTPN1 | P18031 | Tyrosine-protein phosphatase non-receptor type 1 |
| PC-10 | AKR1B15 | C9JRZ8 | Aldo-keto reductase family 1 member B15 |
| PC-10 | AKR1A1 | P14550 | Alcohol dehydrogenase [NADP(+)] |
| PC-10 | AKR1B1 | P15121 | Aldose reductase |
| PC-10 | AKR1E2 | Q96JD6 | 1,5-anhydro-D-fructose reductase |
| PC-10 | HSD11B1 | P28845 | Corticosteroid 11-beta-dehydrogenase isozyme 1 |
| PC-10 | HSD11B1L | Q7Z5J1 | Hydroxysteroid 11-beta-dehydrogenase 1-like protein |
| PC-10 | PTPRF | P10586 | Receptor-type tyrosine-protein phosphatase F |
| PC-10 | ACP1 | P24666 | Low molecular weight phosphotyrosine protein phosphatase |
| PC-10 | [CD81](http://zinc15.docking.org/genes/CD81) | P60033 | CD81 antigen |
| PC-10 | [PIN1](http://zinc15.docking.org/genes/PIN1) | Q13526 | Peptidyl-prolyl cis-trans isomerase NIMA-interacting 1 |
| PC-10 | [PTPN6](http://zinc15.docking.org/genes/PTPN6) | P29350 | Tyrosine-protein phosphatase non-receptor type 6 |
| PC-10 | [POLA1](http://zinc15.docking.org/genes/POLA1) | P09884 | DNA polymerase alpha catalytic subunit |
| PC-11 | PTPN1 | P18031 | Protein-tyrosine phosphatase 1B |
| PC-11 | TOP2A | P11388 | DNA topoisomerase II alpha |
| PC-11 | LTB4R | Q15722 | Leukotriene B4 receptor 1 |
| PC-11 | TNF | P01375 | TNF-alpha |
| PC-11 | PPARG | P37231 | Peroxisome proliferator-activated receptor gamma |
| PC-11 | PPARA | Q07869 | Peroxisome proliferator-activated receptor alpha |
| PC-11 | PPARD | Q03181 | Peroxisome proliferator-activated receptor delta |
| PC-11 | NR3C1 | P04150 | Glucocorticoid receptor |
| PC-11 | CYP19A1 | P11511 | Cytochrome P450 19A1 |
| PC-11 | PTGER1 | P34995 | Prostanoid EP1 receptor |
| PC-11 | PTGER4 | P35408 | Prostanoid EP4 receptor |
| PC-11 | PTGFR | P43088 | Prostanoid FP receptor |
| PC-11 | PTGER3 | P43115 | Prostanoid EP3 receptor |
| PC-11 | PTGER2 | P43116 | Prostanoid EP2 receptor |
| PC-11 | VDR | P11473 | Vitamin D receptor |
| PC-11 | PDE2A | O00408 | Phosphodiesterase 2A |
| PC-11 | FNTA FNTB | P49354 P49356 | Protein farnesyltransferase |
| PC-11 | TYMS | P04818 | Thymidylate synthase (by homology) |
| PC-11 | HSD11B1 | P28845 | 11-beta-hydroxysteroid dehydrogenase 1 |
| PC-11 | ESR1 | P03372 | Estrogen receptor alpha |
| PC-11 | PTGDR | Q13258 | Prostanoid DP receptor |
| PC-11 | CA2 | P00918 | Carbonic anhydrase II |
| PC-11 | ESR2 | Q92731 | Estrogen receptor beta |
| PC-11 | F11 | P03951 | Coagulation factor XI |
| PC-11 | MTOR | P42345 | Serine/threonine-protein kinase mTOR |
| PC-11 | PIK3CA | P42336 | PI3-kinase p110-alpha subunit |
| PC-11 | PDE5A | O76074 | Phosphodiesterase 5A |
| PC-11 | TYR | P14679 | Tyrosinase |
| PC-11 | AGTR1 | P30556 | Type-1 angiotensin II receptor |
| PC-11 | AGTR2 | P50052 | Angiotensin II receptor |
| PC-11 | SLC10A2 | Q12908 | Ileal bile acid transporter |
| PC-11 | GRB2 | P62993 | Growth factor receptor-bound protein 2 |
| PC-11 | PPP2CA | P67775 | Serine/threonine protein phosphatase 2A, catalytic subunit, alpha isoform |
| PC-11 | PTGIR | P43119 | Prostanoid IP receptor |
| PC-11 | SLC22A6 | Q4U2R8 | Solute carrier family 22 member 6 (by homology) |
| PC-11 | ALOX12 | P18054 | Arachidonate 12-lipoxygenase |
| PC-11 | NR1H3 | Q13133 | LXR-alpha |
| PC-11 | RORC | P51449 | Nuclear receptor ROR-gamma |
| PC-11 | PLA2G1B | P04054 | Phospholipase A2 group 1B |
| PC-11 | CDC25B | P30305 | Dual specificity phosphatase Cdc25B |
| PC-11 | EDNRB | P24530 | Endothelin receptor ET-B |
| PC-11 | ITGAV ITGB3 | P06756 P05106 | Integrin alpha-V/beta-3 |
| PC-11 | CCR1 | P32246 | C-C chemokine receptor type 1 |
| PC-11 | EDNRA | P25101 | Endothelin receptor ET-A |
| PC-11 | HDAC11 | Q96DB2 | Histone deacetylase 11 |
| PC-11 | HDAC10 | Q969S8 | Histone deacetylase 10 |
| PC-11 | NR3C2 | P08235 | Mineralocorticoid receptor |
| PC-11 | PGR | P06401 | Progesterone receptor |
| PC-11 | SHBG | P04278 | Testis-specific androgen-binding protein |
| PC-12 | PTPN1 | P18031 | Protein-tyrosine phosphatase 1B |
| PC-12 | PTGDR2 | Q9Y5Y4 | G protein-coupled receptor 44 |
| PC-12 | ITGB1 ITGA4 | P05556 P13612 | Integrin alpha-4/beta-1 |
| PC-12 | ACE | P12821 | Angiotensin-converting enzyme (by homology) |
| PC-12 | CYSLTR1 | Q9Y271 | Cysteinyl leukotriene receptor 1 |
| PC-12 | MME | P08473 | Neprilysin (by homology) |
| PC-12 | EDNRB | P24530 | Endothelin receptor ET-B |
| PC-12 | AGTR1 | P30556 | Type-1 angiotensin II receptor |
| PC-12 | EDNRA | P25101 | Endothelin receptor ET-A |
| PC-12 | FNTA FNTB | P49354 P49356 | Protein farnesyltransferase |
| PC-12 | VDR | P11473 | Vitamin D receptor |
| PC-12 | ALOX5AP | P20292 | 5-lipoxygenase activating protein |
| PC-12 | ITGB7 ITGA4 | P26010 P13612 | Integrin alpha-4/beta-7 |
| PC-12 | TNFRSF10A | O00220 | TRAIL receptor-1 |
| PC-12 | F3 F7 | P13726 P08709 | Coagulation factor VII/tissue factor |
| PC-12 | S1PR2 | O95136 | Sphingosine 1-phosphate receptor Edg-5 |
| PC-12 | NOS2 | P35228 | Nitric oxide synthase, inducible (by homology) |
| PC-12 | PGR | P06401 | Progesterone receptor |
| PC-12 | NR3C1 | P04150 | Glucocorticoid receptor |
| PC-12 | TNF | P01375 | TNF-alpha |
| PC-12 | PTGES | O14684 | Prostaglandin E synthase |
| PC-12 | TYR | P14679 | Tyrosinase |
| PC-12 | GRB2 | P62993 | Growth factor receptor-bound protein 2 |
| PC-12 | CTSA | P10619 | Lysosomal protective protein |
| PC-12 | CAPN2 | P17655 | Calpain 2 |
| PC-12 | AMPD2 | Q01433 | AMP deaminase 2 |
| PC-12 | CTSB | P07858 | Cathepsin (B and K) |
| PC-12 | ITGAV ITGB3 | P06756 P05106 | Integrin alpha-V/beta-3 |
| PC-12 | PYGL | P06737 | Liver glycogen phosphorylase |
| PC-12 | HCAR2 | Q8TDS4 | Hydroxycarboxylic acid receptor 2 |
| PC-12 | HSD11B1 | P28845 | 11-beta-hydroxysteroid dehydrogenase 1 |
| PC-12 | REN | P00797 | Renin |
| PC-12 | OPRD1 | P41143 | Delta opioid receptor |
| PC-12 | MMP13 | P45452 | Matrix metalloproteinase 13 |
| PC-12 | MMP1 | P03956 | Matrix metalloproteinase 1 |
| PC-12 | CASP1 | P29466 | Caspase-1 |
| PC-12 | PGC | P20142 | Pepsinogen C (by homology) |
| PC-12 | LTB4R | Q15722 | Leukotriene B4 receptor 1 |
| PC-12 | TOP2A | P11388 | DNA topoisomerase II alpha |
| PC-12 | CASP3 | P42574 | Caspase-3 |
| PC-12 | CASP8 | Q14790 | Caspase-8 |
| PC-12 | ECE1 | P42892 | Endothelin-converting enzyme 1 |
| PC-12 | CYP19A1 | P11511 | Cytochrome P450 19A1 |
| PC-12 | CASR | P41180 | Calcium sensing receptor |
| PC-12 | PPARG | P37231 | Peroxisome proliferator-activated receptor gamma |
| PC-12 | PTGS2 | P35354 | Cyclooxygenase-2 |
| PC-12 | ITGA2B ITGB3 | P08514 P05106 | Integrin alpha-IIb/beta-3 |
| PC-12 | ITGB5 ITGAV | P18084 P06756 | Integrin alpha-V/beta-5 |
| PC-12 | TBXA2R | P21731 | Thromboxane A2 receptor |
| PC-12 | HMGCR | P04035 | HMG-CoA reductase |
| PC-12 | NLRP3 | Q96P20 | NACHT, LRR and PYD domains-containing protein 3 |
| PC-12 | GPR17 | Q13304 | Uracil nucleotide/cysteinyl leukotriene receptor |
| PC-12 | TBXAS1 | P24557 | Thromboxane-A synthase |
| PC-12 | FYN | P06241 | Tyrosine-protein kinase FYN |
| PC-12 | ADRA2A | P08913 | Alpha-2a adrenergic receptor |
| PC-12 | ADRA2C | P18825 | Adrenergic receptor alpha-2 |
| PC-12 | ADRB2 | P07550 | Adrenergic receptor beta |
| PC-12 | DRD3 | P35462 | Dopamine D3 receptor |
| PC-12 | ADRB3 | P13945 | Beta-3 adrenergic receptor |
| PC-12 | PTGIR | P43119 | Prostanoid IP receptor |
| PC-12 | MMP9 | P14780 | Matrix metalloproteinase 9 |
| PC-12 | MMP2 | P08253 | Matrix metalloproteinase 2 |
| PC-12 | MMP10 | P09238 | Matrix metalloproteinase 10 |
| PC-12 | MMP12 | P39900 | Matrix metalloproteinase 12 |
| PC-12 | MMP8 | P22894 | Matrix metalloproteinase 8 |
| PC-13 | PTPN2 | P17706 | Tyrosine-protein phosphatase non-receptor type 2 |
| PC-13 | PTPN1 | P18031 | Tyrosine-protein phosphatase non-receptor type 1 |
| PC-13 | HSD11B1 | P28845 | Corticosteroid 11-beta-dehydrogenase isozyme 1 |
| PC-13 | HSD11B1L | Q7Z5J1 | Hydroxysteroid 11-beta-dehydrogenase 1-like protein |
| PC-13 | TDP1 | Q9NUW8 | Tyrosyl-DNA phosphodiesterase 1 |
| PC-13 | POLB | P06746 | DNA polymerase beta |
| PC-13 | AKR1B10 | O60218 | Aldo-keto reductase family 1 member B10 |
| PC-13 | AKR1B1 | P15121 | Aldose reductase |
| PC-13 | AKR1B15 | C9JRZ8 | Aldo-keto reductase family 1 member B15 |
| PC-13 | AKR1A1 | P14550 | Alcohol dehydrogenase [NADP(+)] |
| PC-13 | AKR1E2 | Q96JD6 | 1,5-anhydro-D-fructose reductase |
| PC-13 | PLA2G1B | P04054 | Phospholipase A2 |
| PC-13 | PTPRF | P10586 | Receptor-type tyrosine-protein phosphatase F |
| PC-13 | ACP1 | P24666 | Low molecular weight phosphotyrosine protein phosphatase |
| PC-13 | PTPRD | P23468 | Receptor-type tyrosine-protein phosphatase delta |
| PC-13 | RNASE3 | P12724 | Eosinophil cationic protein |
| PC-13 | [CD81](http://zinc15.docking.org/genes/CD81) | P60033 | CD81 antigen |
| PC-13 | [HSD11B2](http://zinc15.docking.org/genes/HSD11B2) | P80365 | Corticosteroid 11-beta-dehydrogenase isozyme 2 |
| PC-13 | [PTPN6](http://zinc15.docking.org/genes/PTPN6) | P29350 | Tyrosine-protein phosphatase non-receptor type 6 |
| PC-13 | [PLCG1](http://zinc15.docking.org/genes/PLCG1) | P19174 | 1-phosphatidylinositol 4,5-bisphosphate phosphodiesterase gamma-1 |
| PC-13 | [POLA1](http://zinc15.docking.org/genes/POLA1) | P09884 | DNA polymerase alpha catalytic subunit |
| PC-13 | [GPBAR1](http://zinc15.docking.org/genes/GPBAR1) | Q8TDU6 | G-protein coupled bile acid receptor 1 |
| PC-14 | TDP1 | Q9NUW8 | Tyrosyl-DNA phosphodiesterase 1 |
| PC-14 | POLB | P06746 | DNA polymerase beta |
| PC-14 | TOP2A | P11388 | DNA topoisomerase 2-alpha |
| PC-14 | TOP2B | Q02880 | DNA topoisomerase 2-beta |
| PC-14 | PTPN2 | P17706 | Tyrosine-protein phosphatase non-receptor type 2 |
| PC-14 | PTPN1 | P18031 | Tyrosine-protein phosphatase non-receptor type 1 |
| PC-14 | NR3C1 | P04150 | Glucocorticoid receptor |
| PC-14 | NR3C2 | P08235 | Mineralocorticoid receptor |
| PC-14 | AKR1B10 | O60218 | Aldo-keto reductase family 1 member B10 |
| PC-14 | AKR1B15 | C9JRZ8 | Aldo-keto reductase family 1 member B15 |
| PC-14 | AKR1B1 | P15121 | Aldose reductase |
| PC-14 | AKR1A1 | P14550 | Alcohol dehydrogenase [NADP(+)] |
| PC-14 | AKR1E2 | Q96JD6 | 1,5-anhydro-D-fructose reductase |
| PC-14 | PTPN6 | P29350 | Tyrosine-protein phosphatase non-receptor type 6 |
| PC-14 | PTPN11 | Q06124 | Tyrosine-protein phosphatase non-receptor type 11 |
| PC-14 | SPTAN1 | Q13813 | Spectrin alpha chain, non-erythrocytic 1 |
| PC-14 | [CD81](http://zinc15.docking.org/genes/CD81) | P60033 | CD81 antigen |
| PC-15 | CYP19A1 | P11511 | Cytochrome P450 19A1 |
| PC-15 | CDC25A | P30304 | M-phase inducer phosphatase 1 |
| PC-15 | CDC25B | P30305 | M-phase inducer phosphatase 2 |
| PC-15 | PTPN2 | P17706 | Tyrosine-protein phosphatase non-receptor type 2 |
| PC-15 | PTPN1 | P18031 | Tyrosine-protein phosphatase non-receptor type 1 |
| PC-15 | LDLR | P01130 | Low-density lipoprotein receptor |
| PC-15 | VLDLR | P98155 | Very low-density lipoprotein receptor |
| PC-15 | LRP8 | Q14114 | Low-density lipoprotein receptor-related protein 8 |
| PC-15 | MAPT | P10636 | Microtubule-associated protein tau |
| PC-15 | NR1H2 | P55055 | Oxysterols receptor LXR-beta |
| PC-15 | NR1H3 | Q13133 | Oxysterols receptor LXR-alpha |
| PC-15 | AR | P10275 | Androgen receptor |
| PC-15 | ESR1 | P03372 | Estrogen receptor |
| PC-15 | ESR2 | Q92731 | Estrogen receptor beta |
| PC-15 | TDP1 | Q9NUW8 | Tyrosyl-DNA phosphodiesterase 1 |
| PC-15 | FDFT1 | P37268 | Squalene synthase |
| PC-15 | CYP51A1 | Q16850 | Lanosterol 14-alpha demethylase |
| PC-15 | MSMO1 | Q15800 | Methylsterol monooxygenase 1 |
| PC-15 | OSBPL9 | Q96SU4 | Oxysterol-binding protein-related protein 9 |
| PC-15 | OSBPL10 | Q9BXB5 | Oxysterol-binding protein-related protein 10 |
| PC-15 | OSBPL11 | Q9BXB4 | Oxysterol-binding protein-related protein 11 |
| PC-15 | OSBP | P22059 | Oxysterol-binding protein 1 |
| PC-15 | OSBP2 | Q969R2 | Oxysterol-binding protein 2 |
| PC-15 | OSBPL5 | Q9H0X9 | Oxysterol-binding protein-related protein 5 |
| PC-15 | OSBPL8 | Q9BZF1 | Oxysterol-binding protein-related protein 8 |
| PC-15 | [CYP17A1](http://zinc15.docking.org/genes/CYP17A1) | P05093 | Steroid 17-alpha-hydroxylase/17,20 lyase |
| PC-15 | [VDR](http://zinc15.docking.org/genes/VDR) | P11473 | Vitamin D3 receptor |
| PC-15 | [CYP24A1](http://zinc15.docking.org/genes/CYP24A1) | Q07973 | 1,25-dihydroxyvitamin D(3) 24-hydroxylase, mitochondrial |
| PC-15 | [CYP27B1](http://zinc15.docking.org/genes/CYP24A1) | O15528 | 25-hydroxyvitamin D-1 alpha hydroxylase, mitochondrial |
| PC-15 | [NPC1L1](http://zinc15.docking.org/genes/NPC1L1) | Q9UHC9 | Niemann-Pick C1-like protein 1 |
| PC-15 | [RORA](http://zinc15.docking.org/genes/RORA) | P35398 | Nuclear receptor ROR-alpha |
| PC-15 | [G6PD](http://zinc15.docking.org/genes/G6PD) | P11413 | Glucose-6-phosphate 1-dehydrogenase |
| PC-15 | [SERPINA6](http://zinc15.docking.org/genes/SERPINA6) | P08185 | Corticosteroid-binding globulin |
| PC-15 | [SHBG](http://zinc15.docking.org/genes/SHBG) | P04278 | Sex hormone-binding globulin |
| PC-15 | [SREBF2](http://zinc15.docking.org/genes/SREBF2) | Q12772 | Sterol regulatory element-binding protein 2 |
| PG-01 | MAPT | P10636 | Microtubule-associated protein tau |
| PG-01 | PRKCG | P05129 | Protein kinase C gamma type |
| PG-01 | PRKCB | P05771 | Protein kinase C beta type |
| PG-01 | PRKCA | P17252 | Protein kinase C alpha type |
| PG-01 | PRKCD | Q05655 | Protein kinase C delta type regulatory subunit |
| PG-01 | PRKCQ | Q04759 | Protein kinase C theta type |
| PG-01 | EPHX2 | P34913 | Lipid-phosphate phosphatase |
| PG-01 | PTPN2 | P17706 | Tyrosine-protein phosphatase non-receptor type 2 |
| PG-01 | PTPN1 | P18031 | Tyrosine-protein phosphatase non-receptor type 1 |
| PG-01 | AR | P10275 | Androgen receptor |
| PG-01 | MGLL | Q99685 | Monoglyceride lipase |
| PG-01 | CNR1 | P21554 | Cannabinoid receptor 1 |
| PG-01 | CNR2 | P34972 | Cannabinoid receptor 2 |
| PG-01 | BCHE | P06276 | Cholinesterase |
| PG-01 | ACHE | P22303 | Acetylcholinesterase |
| PG-01 | LCT | P09848 | Lactase-phlorizin hydrolase |
| PG-01 | DNPEP | Q9ULA0 | Aspartyl aminopeptidase |
| PG-01 | RNPEP | Q9H4A4 | Aminopeptidase B |
| PG-01 | MDH2 | P40926 | Malate dehydrogenase, mitochondrial |
| PG-01 | CHRM4 | P08173 | Muscarinic acetylcholine receptor M4 |
| PG-01 | FUT7 | [Q11130](https://www.uniprot.org/uniprot/Q11130) | Alpha-(1,3)-fucosyltransferase 7 |
| PG-01 | PRTN3 | P24158 | Myeloblastin |
| PG-01 | CHRM5 | P08912 | Muscarinic acetylcholine receptor M5 |
| PG-01 | LAP3 | P28838 | Cytosol aminopeptidase |
| PG-01 | LTB4R | Q15722 | Leukotriene B4 receptor 1 |
| PG-01 | KYNU | Q16719 | Kynureninase |
| PG-01 | CHRM1 | P11229 | Muscarinic acetylcholine receptor M1 |
| PG-01 | CHRM2 | P08172 | Muscarinic acetylcholine receptor M2 |
| PG-01 | PTPN6 | P29350 | Tyrosine-protein phosphatase non-receptor type 6 |
| PG-01 | CHRM3 | P20309 | Muscarinic acetylcholine receptor M3 |
| PG-01 | NAAA | Q02083 | N-acylethanolamine-hydrolyzing acid amidase |
| PG-01 | GBA2 | Q9HCG7 | Non-lysosomal glucosylceramidase |
| PG-01 | KCNA5 | P22460 | Potassium voltage-gated channel subfamily A member 5 |
| PG-01 | STS | P08842 | Steryl-sulfatase |
| PG-01 | PHLPP2 | Q6ZVD8 | PH domain leucine-rich repeat-containing protein phosphatase 2 |
| PG-01 | UGCG | Q16739 | Ceramide glucosyltransferase |
| PG-01 | LTA4H | P09960 | Leukotriene A-4 hydrolase |
| PG-01 | GRM2 | Q14416 | Metabotropic glutamate receptor 2 |
| PG-02 | AR | P10275 | Androgen receptor |
| PG-02 | TDP1 | Q9NUW8 | Tyrosyl-DNA phosphodiesterase 1 |
| PG-02 | CYP19A1 | P11511 | Cytochrome P450 19A1 |
| PG-02 | HMGCR | P04035 | 3-hydroxy-3-methylglutaryl-coenzyme A reductase |
| PG-02 | CYP51A1 | Q16850 | Lanosterol 14-alpha demethylase |
| PG-02 | NR1H2 | P55055 | Oxysterols receptor LXR-beta |
| PG-02 | NR1H3 | Q13133 | Oxysterols receptor LXR-alpha |
| PG-02 | LDLR | P01130 | Low-density lipoprotein receptor |
| PG-02 | VLDLR | P98155 | Very low-density lipoprotein receptor |
| PG-02 | LRP8 | Q14114 | Low-density lipoprotein receptor-related protein 8 |
| PG-02 | CYP17A1 | P05093 | Steroid 17-alpha-hydroxylase/17, 20 lyase |
| PG-02 | ESR1 | P03372 | Estrogen receptor |
| PG-02 | ESR2 | Q92731 | Estrogen receptor beta |
| PG-02 | SLC6A2 | P23975 | Sodium-dependent noradrenaline transporter |
| PG-02 | SLC6A3 | Q01959 | Sodium-dependent dopamine transporter |
| PG-02 | [NPC1L1](http://zinc15.docking.org/genes/NPC1L1) | Q9UHC9 | Niemann-Pick C1-like protein 1 |
| PG-02 | [NPC1](http://zinc15.docking.org/genes/NPC1) | O15118 | Niemann-Pick C1 protein |
| PG-02 | SHBG | P04278 | Sex hormone-binding globulin |
| PG-02 | [GPBAR1](http://zinc15.docking.org/genes/GPBAR1) | Q8TDU6 | G-protein coupled bile acid receptor 1 |
| PG-02 | [SERPINA6](http://zinc15.docking.org/genes/SERPINA6) | P08185 | Corticosteroid-binding globulin |
| PG-02 | SRD5A2 | P31213 | 3-oxo-5-alpha-steroid 4-dehydrogenase 2 |
| PG-02 | G6PD | P11413 | Glucose-6-phosphate 1-dehydrogenase |
| PG-02 | [ST3GAL1](http://zinc15.docking.org/genes/ST3GAL1) | Q11201 | CMP-N-acetylneuraminate-beta-galactosamide-alpha-2,3-sialyltransferase 1 |
| PG-02 | [RORA](http://zinc15.docking.org/genes/RORA) | P35398 | Nuclear receptor ROR-alpha |
| PG-02 | [POLA1](http://zinc15.docking.org/genes/POLA1) | P09884 | DNA polymerase alpha catalytic subunit |
| PG-02 | SRD5A1 | P18405 | 3-oxo-5-alpha-steroid 4-dehydrogenase 1 |
| PG-02 | SHH | Q15465 | Sonic hedgehog protein |
| PG-02 | [SREBF2](http://zinc15.docking.org/genes/SREBF2) | Q12772 | Sterol regulatory element-binding protein 2 |
| PG-02 | EBP | Q15125 | 3-beta-hydroxysteroid-Delta(8),Delta(7)-isomerase |
| PG-02 | ABCC4 | O15439 | Multidrug resistance-associated protein 4 |
| PG-02 | [NR1H4](http://zinc15.docking.org/genes/NR1H4) | Q96RI1 | Bile acid receptor |
| PG-02 | [ABCB11](http://zinc15.docking.org/genes/ABCB11) | O95342 | Bile salt export pump |
| PG-02 | RORC | P51449 | Nuclear receptor ROR-gamma |
| PG-02 | [SLC10A1](http://zinc15.docking.org/genes/SLC10A1) | Q14973 | Sodium/bile acid cotransporter |
| PG-02 | [SLC22A3](http://zinc15.docking.org/genes/SLC22A3) | O75751 | Solute carrier family 22 member 3 |
| PG-02 | [UGT2B7](http://zinc15.docking.org/genes/UGT2B7) | P16662 | UDP-glucuronosyltransferase 2B7 |
| PG-02 | SLCO1B1 | Q9Y6L6 | Solute carrier organic anion transporter family member 1B1 |
| PG-02 | ABCG5 | Q9H222 | ATP-binding cassette sub-family G member 5 |
| PG-02 | ABCG8 | Q9H221 | ATP-binding cassette sub-family G member 8 |
| PG-02 | ABCA1 | O95477 | ATP-binding cassette sub-family A member 1 |
| PG-02 | IL8 | P10145 | Interleukin-8 |
| PG-02 | IL10 | P22301 | Interleukin-10 |
| PG-02 | TNF | P01375 | Tumor necrosis factor |
| PG-03 | TDP1 | Q9NUW8 | Tyrosyl-DNA phosphodiesterase 1 |
| PG-03 | AR | P10275 | Androgen receptor |
| PG-03 | HMGCR | P04035 | 3-hydroxy-3-methylglutaryl-coenzyme A reductase |
| PG-03 | CYP51A1 | Q16850 | Lanosterol 14-alpha demethylase |
| PG-03 | NR1H2 | P55055 | Oxysterols receptor LXR-beta |
| PG-03 | NR1H3 | Q13133 | Oxysterols receptor LXR-alpha |
| PG-03 | CYP19A1 | P11511 | Cytochrome P450 19A1 |
| PG-03 | CYP17A1 | P05093 | Steroid 17-alpha-hydroxylase/17,20 lyase |
| PG-03 | LDLR | P01130 | Low-density lipoprotein receptor |
| PG-03 | VLDLR | P98155 | Very low-density lipoprotein receptor |
| PG-03 | LRP8 | Q14114 | Low-density lipoprotein receptor-related protein 8 |
| PG-03 | ESR1 | P03372 | Estrogen receptor |
| PG-03 | ESR2 | Q92731 | Estrogen receptor beta |
| PG-03 | SLC6A2 | P23975 | Sodium-dependent noradrenaline transporter |
| PG-03 | SLC6A4 | P31645 | Sodium-dependent serotonin transporter |
| PG-03 | [NPC1L1](http://zinc15.docking.org/genes/NPC1L1) | Q9UHC9 | Niemann-Pick C1-like protein 1 |
| PG-03 | [NPC1](http://zinc15.docking.org/genes/NPC1) | O15118 | Niemann-Pick C1 protein |
| PG-03 | SHBG | P04278 | Sex hormone-binding globulin |
| PG-03 | [SLC10A1](http://zinc15.docking.org/genes/SLC10A1) | Q14973 | Sodium/bile acid cotransporter |
| PG-03 | SRD5A2 | P31213 | 3-oxo-5-alpha-steroid 4-dehydrogenase 2 |
| PG-03 | ABCC4 | O15439 | Multidrug resistance-associated protein 4 |
| PG-03 | GC | P02774 | Vitamin D-binding protein |
| PG-03 | [SLC10A2](http://zinc15.docking.org/genes/SLC10A2) | Q12908 | Ileal sodium/bile acid cotransporter |
| PG-03 | CDC45 | O75419 | Cell division control protein 45 homolog |
| PG-03 | [SERPINA6](http://zinc15.docking.org/genes/SERPINA6) | P08185 | Corticosteroid-binding globulin |
| PG-03 | SRD5A1 | P18405 | 3-oxo-5-alpha-steroid 4-dehydrogenase 1 |
| PG-03 | VDR | P11473 | Vitamin D3 receptor |
| PG-03 | [CDC25A](http://zinc15.docking.org/genes/CDC25A) | P30304 | M-phase inducer phosphatase 1 |
| PG-03 | CD4 | P01730 | T-cell surface glycoprotein CD4 |
| PG-03 | NR1H4 | Q96RI1 | Bile acid receptor |
| PG-03 | G6PD | P11413 | Glucose-6-phosphate 1-dehydrogenase |
| PG-03 | [CYP24A1](http://zinc15.docking.org/genes/CYP24A1) | Q07973 | 1,25-dihydroxyvitamin D(3) 24-hydroxylase, mitochondrial |
| PG-03 | [CYP27B1](http://zinc15.docking.org/genes/CYP27B1) | O15528 | 25-hydroxyvitamin D-1 alpha hydroxylase, mitochondrial |
| PG-03 | [ST3GAL1](http://zinc15.docking.org/genes/ST3GAL1) | Q11201 | CMP-N-acetylneuraminate-beta-galactosamide-alpha-2,3-sialyltransferase 1 |
| PG-03 | ESR2 | Q92731 | Estrogen receptor beta |
| PG-03 | [RORA](http://zinc15.docking.org/genes/RORA) | P35398 | Nuclear receptor ROR-alpha |
| PG-03 | [POLA1](http://zinc15.docking.org/genes/POLA1) | P09884 | DNA polymerase alpha catalytic subunit |
| PG-03 | EBP | Q15125 | 3-beta-hydroxysteroid-Delta(8),Delta(7)-isomerase |
| PG-03 | [SREBF2](http://zinc15.docking.org/genes/SREBF2) | Q12772 | Sterol regulatory element-binding protein 2 |
| PG-03 | CA2 | P00918 | Carbonic anhydrase 2 |
| PG-03 | CA1 | P00915 | Carbonic anhydrase 1 |
| PG-03 | [ABCB11](http://zinc15.docking.org/genes/ABCB11) | O95342 | Bile salt export pump |
| PG-03 | RORC | P51449 | Nuclear receptor ROR-gamma |
| PG-03 | [ATIC](http://zinc15.docking.org/genes/ATIC) | P31939 | Bifunctional purine biosynthesis protein PURH |
| PG-03 | [SLC22A3](http://zinc15.docking.org/genes/SLC22A3) | O75751 | Solute carrier family 22 member 3 |
| PG-03 | SHH | Q15465 | Sonic hedgehog protein |
| PG-03 | CYP7A1 | P22680 | Cholesterol 7-alpha-monooxygenase |
| PG-03 | [SREBF1](http://zinc15.docking.org/genes/SREBF2) | P36956 | Sterol regulatory element-binding protein 1 |
| PG-03 | DHCR24 | Q15392 | Delta(24)-sterol reductase |
| PG-03 | ABCG5 | Q9H222 | ATP-binding cassette sub-family G member 5 |
| PG-03 | CASP3 | P42574 | Caspase-3 |
| PG-03 | ABCG8 | Q9H221 | ATP-binding cassette sub-family G member 8 |
| PG-03 | ICAM1 | P05362 | Intercellular adhesion molecule 1 |
| PG-03 | APOE | P02649 | Apolipoprotein E |
| PG-04 | CRYZ | Q08257 | Quinone oxidoreductase |
| PG-04 | ALOX15B | O15296 | Arachidonate 15-lipoxygenase B |
| PG-04 | ALOX5 | P09917 | Arachidonate 5-lipoxygenase |
| PG-04 | ALOX15 | P16050 | Arachidonate 15-lipoxygenase |
| PG-04 | ALOX12 | P18054 | Arachidonate 12-lipoxygenase, 12S-type |
| PG-04 | ALOXE3 | Q9BYJ1 | Epidermis-type lipoxygenase 3 |
| PG-04 | PTPN1 | P18031 | Tyrosine-protein phosphatase non-receptor type 1 |
| PG-04 | PTPN2 | P17706 | Tyrosine-protein phosphatase non-receptor type 2 |
| PG-04 | ALOX12B | O75342 | Arachidonate 12-lipoxygenase, 12R-type |
| PG-04 | SRC | P12931 | Proto-oncogene tyrosine-protein kinase Src |
| PG-04 | FYN | P06241 | Tyrosine-protein kinase Fyn |
| PG-04 | YES1 | P07947 | Tyrosine-protein kinase Yes |
| PG-04 | FGR | P09769 | Tyrosine-protein kinase Fgr |
| PG-04 | FRK | P42685 | Tyrosine-protein kinase FRK |
| PG-04 | CYP19A1 | P11511 | Cytochrome P450 19A1 |
| PG-04 | FFAR1 | O14842 | Free fatty acid receptor 1 |
| PG-05 | MBNL1 | Q9NR56 | Muscleblind-like protein 1 |
| PG-05 | MBNL2 | Q5VZF2 | Muscleblind-like protein 2 |
| PG-05 | MBNL3 | Q9NUK0 | Muscleblind-like protein 3 |
| PG-05 | CHRM2 | P08172 | Muscarinic acetylcholine receptor M2 |
| PG-05 | CHRM4 | P08173 | Muscarinic acetylcholine receptor M4 |
| PG-05 | CHRM5 | P08912 | Muscarinic acetylcholine receptor M5 |
| PG-05 | CHRM1 | P11229 | Muscarinic acetylcholine receptor M1 |
| PG-05 | CHRM3 | P20309 | Muscarinic acetylcholine receptor M3 |
| PG-05 | TDP1 | Q9NUW8 | Tyrosyl-DNA phosphodiesterase 1 |
| PG-05 | HTR2A | P28223 | 5-hydroxytryptamine receptor 2A |
| PG-05 | HTR2C | P28335 | 5-hydroxytryptamine receptor 2C |
| PG-05 | HTR2B | P41595 | 5-hydroxytryptamine receptor 2B |
| PG-05 | ADRA1D | P25100 | Alpha-1D adrenergic receptor |
| PG-05 | ADRA1A | P35348 | Alpha-1A adrenergic receptor |
| PG-05 | ADRA1B | P35368 | Alpha-1B adrenergic receptor |
| PG-05 | [SLC18A2](http://zinc15.docking.org/genes/SLC18A2) | Q05940 | Synaptic vesicular amine transporter |
| PG-05 | [LGALS1](http://zinc15.docking.org/genes/LGALS1) | P09382 | Galectin-1 |
| PG-05 | [PABPC1](http://zinc15.docking.org/genes/PABPC1) | [P11940](https://www.uniprot.org/uniprot/P11940) | Polyadenylate-binding protein 1 |
| PG-05 | LGALS9 | O00182 | Galectin-9 |
| PG-06 | OPRM1 | P35372 | Mu-type opioid receptor |
| PG-06 | OPRD1 | P41143 | Delta-type opioid receptor |
| PG-06 | OPRK1 | P41145 | Kappa-type opioid receptor |
| PG-06 | OPRL1 | P41146 | Nociceptin receptor |
| PG-06 | DRD4 | P21917 | D(4) dopamine receptor |
| PG-06 | HTR2A | P28223 | 5-hydroxytryptamine receptor 2A |
| PG-06 | CCR1 | P32246 | C-C chemokine receptor type 1 |
| PG-06 | CCR2 | P41597 | C-C chemokine receptor type 2 |
| PG-06 | CCR3 | P51677 | C-C chemokine receptor type 3 |
| PG-06 | CCR5 | P51681 | C-C chemokine receptor type 5 |
| PG-06 | CCRL2 | C9JP23 | C-C chemokine receptor-like 2 |
| PG-06 | SLC6A2 | P23975 | Sodium-dependent noradrenaline transporter |
| PG-06 | SLC6A4 | P31645 | Sodium-dependent serotonin transporter |
| PG-06 | SLC6A3 | Q01959 | Sodium-dependent dopamine transporter |
| PG-06 | PRCP | P42785 | Lysosomal Pro-X carboxypeptidase |
| PG-06 | CYP2C9 | P11712 | Cytochrome P450 2C9 |
| PG-06 | KDM2A | Q9Y2K7 | Lysine-specific demethylase 2A |
| PG-06 | KDM5C | P41229 | Lysine-specific demethylase 5C |
| PG-06 | L3MBTL1 | Q9Y468 | Lethal(3)malignant brain tumor-like protein 1 |
| PG-06 | L3MBTL3 | Q96JM7 | Lethal(3)malignant brain tumor-like protein 3 |
| PG-06 | L3MBTL4 | Q8NA19 | Lethal(3)malignant brain tumor-like protein 4 |
| PG-06 | MAP3K12 | Q12852 | Mitogen-activated protein kinase kinase kinase 12 |
| PG-06 | MBTD1 | Q05BQ5 | MBT domain-containing protein 1 |
| PG-06 | IKBKG | Q9Y6K9 | NF-kappa-B essential modulator |
| PG-06 | NPC1L1 | Q9UHC9 | Niemann-Pick C1-like protein 1 |
| PG-06 | NPSR1 | Q6W5P4 | Neuropeptide S receptor |
| PG-06 | NPY2R | P49146 | Neuropeptide Y receptor type 2 |
| PG-06 | PARP1 | P09874 | Poly [ADP-ribose] polymerase 1 |
| PG-06 | PREP | P48147 | Prolyl endopeptidase |
| PG-06 | RBP4 | P02753 | Retinol-binding protein 4 |
| PG-06 | SLC5A1 | P13866 | Sodium/glucose cotransporter 1 |
| PG-06 | SLC6A7 | Q99884 | Sodium-dependent proline transporter |
| PG-06 | TP53BP1 | Q12888 | Tumor suppressor p53-binding protein 1 |
| PG-06 | SLC18A3 | Q16572 | Vesicular acetylcholine transporter |
| PG-07 | BCHE | P06276 | Cholinesterase |
| PG-07 | ACHE | P22303 | Acetylcholinesterase |
| PG-07 | OPRM1 | P35372 | Mu-type opioid receptor |
| PG-07 | OPRD1 | P41143 | Delta-type opioid receptor |
| PG-07 | OPRK1 | P41145 | Kappa-type opioid receptor |
| PG-07 | OPRL1 | P41146 | Nociceptin receptor |
| PG-07 | EDNRB | P24530 | Endothelin B receptor |
| PG-07 | EDNRA | P25101 | Endothelin-1 receptor |
| PG-07 | TRPV1 | Q8NER1 | Transient receptor potential cation channel subfamily V member 1 |
| PG-07 | MBNL1 | Q9NR56 | Muscleblind-like protein 1 |
| PG-07 | MBNL2 | Q5VZF2 | Muscleblind-like protein 2 |
| PG-07 | MBNL3 | Q9NUK0 | Muscleblind-like protein 3 |
| PG-07 | MAPT | P10636 | Microtubule-associated protein tau |
| PG-07 | REN | P00797 | Renin |
| PG-07 | CTSD | P07339 | Cathepsin D |
| PG-07 | F3 | P13726 | Tissue factor |
| PG-08 | FABP3 | P05413 | Fatty acid-binding protein , heart |
| PG-08 | FABP4 | P15090 | Fatty acid-binding protein, adipocyte |
| PG-08 | PTGS1 | P23219 | Prostaglandin G/H synthase 1 |
| PG-08 | PTGS2 | P35354 | Prostaglandin G/H synthase 2 |
| PG-08 | FABP5 | Q01469 | Fatty acid-binding protein 5 |
| PG-08 | FABP12 | A6NFH5 | Fatty acid-binding protein 12 |
| PG-08 | FABP7 | O15540 | Fatty acid-binding protein, brain |
| PG-08 | PMP2 | P02689 | Myelin P2 protein |
| PG-08 | FABP9 | Q0Z7S8 | Fatty acid-binding protein 9 |
| PG-08 | PPARG | P37231 | Peroxisome proliferator-activated receptor gamma |
| PG-08 | PPARD | Q03181 | Peroxisome proliferator-activated receptor delta |
| PG-08 | PPARA | Q07869 | Peroxisome proliferator-activated receptor alpha |
| PG-08 | ALOX5 | P09917 | Arachidonate 5-lipoxygenase |
| PG-08 | ALOX15 | P16050 | Arachidonate 15-lipoxygenase |
| PG-08 | ALOX12 | P18054 | Arachidonate 12-lipoxygenase, 12S-type |
| PG-08 | ACER2 | Q5QJU3 | Alkaline ceramidase 2 |
| PG-08 | PAM | P19021 | Peptidyl-glycine alpha-amidating monooxygenase |
| PG-08 | ASAH1 | Q13510 | Acid ceramidase |
| PG-08 | BBOX1 | O75936 | Gamma-butyrobetaine dioxygenase |
| PG-08 | COL4A3BP | Q9Y5P4 | Collagen type IV alpha-3-binding protein |
| PG-08 | CNR1 | P21554 | Cannabinoid receptor 1 |
| PG-08 | CNR2 | P34972 | Cannabinoid receptor 2 |
| PG-08 | CYP4F2 | P78329 | Phylloquinone omega-hydroxylase CYP4F2 |
| PG-08 | HSD17B3 | P37058 | Testosterone 17-beta-dehydrogenase 3 |
| PG-08 | DNM1 | Q05193 | Dynamin-1 |
| PG-08 | ENPP2 | Q13822 | Ectonucleotide pyrophosphatase/phosphodiesterase family member 2 |
| PG-08 | CES1 | P02795 | Liver carboxylesterase 1 |
| PG-08 | CES2 | O00748 | Cocaine esterase |
| PG-08 | FAAH | P23141 | Fatty-acid amide hydrolase 1 |
| PG-08 | FFAR4 | O00519 | Free fatty acid receptor 4 |
| PG-08 | GABBR2 | Q7L5A8 | Gamma-aminobutyric acid type B receptor subunit 2 |
| PG-08 | GPR174 | Q5NUL3 | Probable G-protein coupled receptor 174 |
| PG-08 | GPR34 | O75899 | Probable G-protein coupled receptor 34 |
| PG-08 | HAO1 | Q9BXC1 | Hydroxyacid oxidase 1 |
| PG-08 | HMGCR | Q9UPC5 | 3-hydroxy-3-methylglutaryl-coenzyme A reductase |
| PG-08 | EPHX2 | Q9UJM8 | Bifunctional epoxide hydrolase 2 |
| PG-08 | KAT5 | P04035 | Histone acetyltransferase KAT5 |
| PG-08 | KDM5A | P34913 | Lysine-specific demethylase 5A |
| PG-08 | PRKCA | Q92993 | Protein kinase C alpha type |
| PG-08 | LPAR1 | P29375 | Lysophosphatidic acid receptor 1 |
| PG-08 | LPAR2 | P17252 | Lysophosphatidic acid receptor 2 |
| PG-08 | LPAR3 | Q92633 | Lysophosphatidic acid receptor 3 |
| PG-08 | LPAR4 | Q9HBW0 | Lysophosphatidic acid receptor 4 |
| PG-08 | LPAR5 | Q9UBY5 | Lysophosphatidic acid receptor 5 |
| PG-08 | LPAR6 | Q99677 | Lysophosphatidic acid receptor 6 |
| PG-08 | LTB4R | Q9H1C0 | Leukotriene B4 receptor 1 |
| PG-08 | SLC25A20 | P43657 | Mitochondrial carnitine/acylcarnitine carrier protein |
| PG-08 | MGLL | Q15722 | Monoglyceride lipase |
| PG-08 | CDC25A | O43772 | M-phase inducer phosphatase 1 |
| PG-08 | CDC25C | Q99685 | M-phase inducer phosphatase 3 |
| PG-08 | ABCC2 | P30304 | Canalicular multispecific organic anion transporter 1 |
| PG-08 | NAAA | P30307 | N-acylethanolamine-hydrolyzing acid amidase |
| PG-08 | OXER1 | Q92887 | Oxoeicosanoid receptor 1 |
| PG-08 | P2RY10 | Q02083 | Putative P2Y purinoceptor 10 |
| PG-08 | PLA2G4A | Q8TDS5 | Cytosolic phospholipase A2 |
| PG-08 | PLA2G4B | O00398 | Cytosolic phospholipase A2 beta |
| PG-08 | PLA2G4C | P47712 | Cytosolic phospholipase A2 gamma |
| PG-08 | PLA2G2A | P0C869 | Phospholipase A2, membrane associated |
| PG-08 | PLA2G2C | Q9UP65 | Putative inactive group IIC secretory phospholipase A2 |
| PG-08 | PLA2G10 | P14555 | Group 10 secretory phospholipase A2 |
| PG-08 | PTGDR | Q5R387 | Prostaglandin D2 receptor |
| PG-08 | PDCD4 | O15496 | Programmed cell death protein 4 |
| PG-08 | PTGER2 | Q13258 | Prostaglandin E2 receptor EP2 subtype |
| PG-08 | PTGER3 | Q53EL6 | Prostaglandin E2 receptor EP3 subtype |
| PG-08 | PTGER4 | P43116 | Prostaglandin E2 receptor EP4 subtype |
| PG-08 | PTGFR | P43115 | Prostaglandin F2-alpha receptor |
| PG-08 | PTGIR | P35408 | Prostacyclin receptor |
| PG-08 | POLH | P43088 | DNA polymerase eta |
| PG-08 | POLK | P43119 | DNA polymerase kappa |
| PG-08 | S1PR2 | O75417 | Sphingosine 1-phosphate receptor 2 |
| PG-08 | SLC22A6 | Q9Y253 | Solute carrier family 22 member 6 |
| PG-08 | SLC22A8 | Q9UBT6 | Solute carrier family 22 member 8 |
| PG-08 | SLC6A11 | O95136 | Sodium- and chloride-dependent GABA transporter 3 |
| PG-08 | SLCO2A1 | Q4U2R8 | Solute carrier organic anion transporter family member 2A1 |
| PG-08 | TBXA2R | Q8TCC7 | Thromboxane A2 receptor |
| PG-08 | TBXAS1 | P48066 | Thromboxane-A synthase |
| PG-08 | TLR2 | Q92959 | Toll-like receptor 2 |
| PG-08 | TRPV1 | P21731 | Transient receptor potential cation channel subfamily V member 1 |
| PG-08 | PRKCA | P17252 | Protein kinase C alpha type |
| PG-08 | PRKCG | P05129 | Protein kinase C gamma type |
| PG-08 | ALOX15B | O15296 | Arachidonate 15-lipoxygenase B |
| PG-08 | PTGS1 | P23219 | Prostaglandin G/H synthase 1 |
| PG-08 | PTGS2 | P35354 | Prostaglandin G/H synthase 2 |
| PG-08 | PLA2G1B | P04054 | Phospholipase A2 |
| PG-09 | MBNL1 | Q9NR56 | Muscleblind-like protein 1 |
| PG-09 | MBNL2 | Q5VZF2 | Muscleblind-like protein 2 |
| PG-09 | MBNL3 | Q9NUK0 | Muscleblind-like protein 3 |
| PG-09 | DYRK1A | Q13627 | Dual specificity tyrosine-phosphorylation-regulated kinase 1A |
| PG-09 | CA12 | O43570 | Carbonic anhydrase 12 |
| PG-09 | CA1 | P00915 | Carbonic anhydrase 1 |
| PG-09 | CA2 | P00918 | Carbonic anhydrase 2 |
| PG-09 | CA3 | P07451 | Carbonic anhydrase 3 |
| PG-09 | CA5A | P35218 | Carbonic anhydrase 5A , mitochondrial |
| PG-09 | CA7 | P43166 | Carbonic anhydrase 7 |
| PG-09 | CA9 | Q16790 | Carbonic anhydrase 9 |
| PG-09 | CA13 | Q8N1Q1 | Carbonic anhydrase 13 |
| PG-09 | CA14 | Q9ULX7 | Carbonic anhydrase 14 |
| PG-09 | CA5B | Q9Y2D0 | Carbonic anhydrase 5B , mitochondrial |
| PG-09 | CA4 | P22748 | Carbonic anhydrase 4 |
| PG-09 | ABCG2 | Q9UNQ0 | ATP-binding cassette sub-family G member 2 |
| PG-09 | AHR | P35869 | Aryl hydrocarbon receptor |
| PG-09 | MAOA | P21397 | Amine oxidase [flavin-containing] A |
| PG-09 | MAOB | P27338 | Amine oxidase [flavin-containing] B |
| PG-09 | CYP11B1 | P15538 | Cytochrome P450 11B1, mitochondrial |
| PG-09 | CYP11B2 | P19099 | Cytochrome P450 11B2, mitochondrial |
| PG-09 | CBR1 | P16152 | Carbonyl reductase [NADPH] 1 |
| PG-09 | CYP17A1 | P05093 | Steroid 17-alpha-hydroxylase/17,20 lyase |
| PG-09 | CYP19A1 | P11511 | Aromatase |
| PG-09 | CYP1A1 | P04798 | Cytochrome P450 1A1 |
| PG-09 | CYP1B1 | Q16678 | Cytochrome P450 1B1 |
| PG-09 | ESR2 | Q92731 | Estrogen receptor beta |
| PG-09 | NQO1 | P15559 | NAD(P)H dehydrogenase [quinone] 1 |
| PG-09 | NR2E3 | Q9Y5X4 | Photoreceptor-specific nuclear receptor |
| PG-09 | NR2E3 | Q16549 | Photoreceptor-specific nuclear receptor |
| PG-09 | PCSK7 | P15907 | Proprotein convertase subtilisin/kexin type 7 |
| PG-09 | ST6GAL1 | O95271 | Beta-galactoside alpha-2,6-sialyltransferase 1 |
| PG-09 | TNKS | Q9H2K2 | Tankyrase-1 |
| PG-09 | TNKS2 | Q9UNQ0 | Tankyrase-2 |
| PG-10 | STAT3 | P40763 | Signal transducer and activator of transcription 3 |
| PG-10 | STAT1 | P42224 | Signal transducer and activator of transcription 1-alpha/beta |
| PG-10 | STAT2 | P52630 | Signal transducer and activator of transcription 2 |
| PG-10 | STAT4 | Q14765 | Signal transducer and activator of transcription 4 |
| PG-10 | PTAFR | P25105 | Platelet-activating factor receptor |
| PG-10 | KDR | P35968 | Vascular endothelial growth factor receptor 2 |
| PG-10 | FLT1 | P17948 | Vascular endothelial growth factor receptor 1 |
| PG-10 | FLT4 | P35916 | Vascular endothelial growth factor receptor 3 |
| PG-10 | MBNL1 | Q9NR56 | Muscleblind-like protein 1 |
| PG-10 | MBNL2 | Q5VZF2 | Muscleblind-like protein 2 |
| PG-10 | MBNL3 | Q9NUK0 | Muscleblind-like protein 3 |
| PG-10 | AR | P10275 | Androgen receptor |
| PG-10 | VDR | P11473 | Vitamin D3 receptor |
| PG-10 | DRD2 | P14416 | D(2) dopamine receptor |
| PG-10 | ADRA1D | P25100 | Alpha-1D adrenergic receptor |
| PG-10 | BCL2A1 | Q16548 | Bcl-2-related protein A1 |
| PG-10 | RHAG | Q02094 | Ammonium transporter Rh type A |
| PG-10 | LGALS4 | P56470 | Galectin-4 |
| PG-10 | LGALS8 | O00214 | Galectin-8 |
| PG-10 | FGF1 | P05230 | Fibroblast growth factor 1 |
| PG-10 | FGF2 | P09038 | Fibroblast growth factor 2 |
| PG-10 | LGALS3 | P17931 | Galectin-3 |
| PG-11 | STAT3 | P40763 | Signal transducer and activator of transcription 3 |
| PG-11 | IL2 | P60568 | Interleukin-2 |
| PG-11 | BCL2L1 | Q07817 | Apoptosis regulator Bcl-X |
| PG-11 | PSEN2 | P49810 | Presenilin-2 |
| PG-11 | PSENEN | Q9NZ42 | Gamma-secretase subunit PEN-2 |
| PG-11 | NCSTN | Q92542 | Nicastrin |
| PG-11 | APH1A | Q96BI3 | Gamma-secretase subunit APH-1A |
| PG-11 | PSEN1 | P49768 | Presenilin-1 |
| PG-11 | APH1B | Q8WW43 | Gamma-secretase subunit APH-1B |
| PG-11 | MMP9 | P14780 | Matrix metalloproteinase 9 |
| PG-11 | PTAFR | P25105 | Platelet activating factor receptor |
| PG-11 | MET | P08581 | Hepatocyte growth factor receptor |
| PG-11 | ALK | Q9UM73 | ALK tyrosine kinase receptor |
| PG-11 | PRF1 | P14222 | Perforin-1 |
| PG-11 | NR3C1 | P04150 | Glucocorticoid receptor |
| PG-11 | HSP90AA1 | P07900 | Heat shock protein HSP 90-alpha |
| PG-11 | SIRT2 | Q8IXJ6 | NAD-dependent deacetylase sirtuin 2 |
| PG-11 | AR | P10275 | Androgen Receptor |
| PG-11 | HDAC6 | Q9UBN7 | Histone deacetylase 6 (by homology) |
| PG-11 | HDAC1 | Q13547 | Histone deacetylase 1 |
| PG-11 | HDAC4 | P56524 | Histone deacetylase 4 |
| PG-11 | MAOA | P21397 | Monoamine oxidase A |
| PG-11 | MCHR1 | Q99705 | Melanin-concentrating hormone receptor 1 |
| PG-11 | MTOR | P42345 | Serine/threonine-protein kinase mTOR |
| PG-11 | PIK3CA | P42336 | PI3-kinase p110-alpha subunit |
| PG-11 | TRPV4 | Q9HBA0 | Transient receptor potential cation channel subfamily V member 4 (by homology) |
| PG-11 | SCD | O00767 | Acyl-CoA desaturase |
| PG-11 | EGFR | P00533 | Epidermal growth factor receptor erbB1 |
| PG-11 | ADORA1 | P30542 | Adenosine A1 receptor (by homology) |
| PG-11 | PIK3CB | P42338 | PI3-kinase p110-beta subunit |
| PG-11 | CDK2 | P24941 | Cyclin-dependent kinase 2 |
| PG-11 | CCNA1 | P78396 | Cyclin-A1 |
| PG-11 | CCNA2 | P20248 | Cyclin-A2 |
| PG-11 | PIM1 | P11309 | Serine/threonine-protein kinase PIM1 |
| PG-11 | MAPK14 | Q16539 | MAP kinase p38 alpha |
| PG-11 | PIM2 | Q9P1W9 | Serine/threonine-protein kinase PIM2 |
| PG-11 | ADORA3 | P0DMS8 | Adenosine A3 receptor |
| PG-11 | SLC5A2 | P31639 | Sodium/glucose cotransporter 2 |
| PG-11 | PDE5A | O76074 | Phosphodiesterase 5A |
| PG-11 | TYMS | P04818 | Thymidylate synthase (by homology) |
| PG-11 | PDE6A | P16499 | Phosphodiesterase 6A |
| PG-11 | PGK1 | P00558 | Phosphoglycerate kinase 1 |
| PG-11 | PTGER1 | P34995 | Prostanoid EP1 receptor (by homology) |
| PG-11 | CCR1 | P32246 | C-C chemokine receptor type 1 |
| PG-11 | SIGMAR1 | Q99720 | Sigma opioid receptor |
| PG-11 | ICAM1 | P05362 | Intercellular adhesion molecule-1 |
| PG-11 | SELE | P16581 | Selectin E |
| PG-11 | MAPK1 | P28482 | MAP kinase ERK2 |
| PG-11 | S1PR1 | P21453 | Sphingosine 1-phosphate receptor Edg-1 |
| PG-11 | CDK2 | P24941 | Cyclin-dependent kinase 2 |
| PG-11 | ITGAL | P20701 | Leukocyte adhesion glycoprotein LFA-1 alpha |
| PG-11 | SYK | P43405 | Tyrosine-protein kinase SYK |
| PG-11 | RASGRP3 | Q8IV61 | RAS guanyl releasing protein 3 |
| PG-11 | REN | P00797 | Renin |
| PG-11 | ADK | P55263 | Adenosine kinase |
| PG-11 | SLC8A1 | P32418 | Sodium/calcium exchanger 1 |
| PG-11 | AURKA | O14965 | Serine/threonine-protein kinase Aurora-A |
| PG-11 | SLC5A1 | P13866 | Sodium/glucose cotransporter 1 |
| PG-11 | LIMK2 | P53671 | LIM domain kinase 2 |
| PG-11 | SMO | Q99835 | Smoothened homolog |
| PG-11 | UPP1 | Q16831 | Uridine phosphorylase 1 (by homology) |
| PG-11 | ATP1A1 | P05023 | Sodium/potassium-transporting ATPase alpha-1 chain |
| PG-11 | CSNK2A1 | P68400 | Casein kinase II alpha |
| PG-11 | CSNK2A2 | P19784 | Casein kinase II alpha (prime) |
| PG-11 | PDE2A | O00408 | Phosphodiesterase 2A |
| PG-11 | PDE4B | Q07343 | Phosphodiesterase 4B |
| PG-11 | MAP2K1 | Q02750 | Dual specificity mitogen-activated protein kinase kinase 1 |
| PG-11 | GYS1 | P13807 | Muscle glycogen synthase |
| PG-11 | HDAC3 | O15379 | Histone deacetylase 3 |
| PG-11 | PDPK1 | O15530 | 3-phosphoinositide dependent protein kinase-1 |
| PG-11 | SLC6A4 | P31645 | Serotonin transporter (by homology) |
| PG-11 | SLC6A3 | Q01959 | Dopamine transporter (by homology) |
| PG-11 | GRB2 | P62993 | Growth factor receptor-bound protein 2 |
| PG-11 | HSD11B1 | P28845 | 11-beta-hydroxysteroid dehydrogenase 1 |
| PG-11 | AGTR1 | P30556 | Type-1 angiotensin II receptor (by homology) |
| PG-11 | NTRK1 | P04629 | Nerve growth factor receptor Trk-A |
| PG-11 | VHL | P40337 | Von Hippel-Lindau disease tumor suppressor/Elongin B/Elongin C |
| PG-11 | S1PR3 | Q99500 | Sphingosine 1-phosphate receptor Edg-3 |
| PG-11 | F7 | P08709 | Coagulation factor VII |
| PG-11 | NR4A2 | P43354 | Nuclear receptor subfamily 4 group A member 2 |
| PG-11 | PTGES | O14684 | Prostaglandin E synthase |
| PG-11 | BCL2A1 | Q16548 | Bcl-2-related protein A1 |
| PG-11 | MAPK8 | P45983 | c-Jun N-terminal kinase 1 |
| PG-11 | MAP3K14 | Q99558 | Mitogen-activated protein kinase kinase kinase 14 |
| PG-11 | ITGB7 | P26010 | Integrin beta-7 |
| PG-11 | ITGA4 | P13612 | Integrin alpha-4 |
| PG-11 | CCNT1 | O60563 | Cyclin T1 |
| PG-11 | PFKFB3 | Q16875 | 6-phosphofructo-2-kinase/fructose-2,6-bisphosphatase 3 |
| PG-11 | SGK1 | O00141 | Serine/threonine-protein kinase Sgk1 |
| PG-11 | F10 | P00742 | Thrombin and coagulation factor X |
| PG-11 | PIK3CG | P48736 | PI3-kinase p110-gamma subunit |
| PG-11 | GSK3B | P49841 | Glycogen synthase kinase-3 beta |
| PG-11 | CCR5 | P51681 | C-C chemokine receptor type 5 |
| PG-11 | F2R | P25116 | Proteinase-activated receptor 1 |
| PG-11 | ALOX5AP | P20292 | 5-lipoxygenase activating protein |
| PG-11 | BACE1 | P56817 | Beta-secretase 1 |
| PG-11 | ACVRL1 | P37023 | Serine/threonine-protein kinase receptor R3 |
| PG-11 | RORC | P51449 | Nuclear receptor ROR-gamma |
| PG-11 | VEGFA | P15692 | Vascular endothelial growth factor A |
| PG-11 | FGF1 | P05230 | Acidic fibroblast growth factor |
| PG-11 | FGF2 | P09038 | Basic fibroblast growth factor |
| PG-11 | DHFR | P00374 | Dihydrofolate reductase |
| PG-11 | HCAR2 | Q8TDS4 | Hydroxycarboxylic acid receptor 2 |
| PG-11 | PDE10A | Q9Y233 | Phosphodiesterase 10A |
| PG-11 | CNR1 | P21554 | Cannabinoid receptor 1 |
| PG-11 | SLC29A1 | Q99808 | Equilibrative nucleoside transporter 1 |
| PG-11 | PPARG | P37231 | Peroxisome proliferator-activated receptor gamma |
| PG-11 | FGFR3 | P22607 | Fibroblast growth factor receptor 3 |
| PG-12 | PTGS1 | P23219 | Prostaglandin G/H synthase 1 |
| PG-12 | PTGS2 | P35354 | Prostaglandin G/H synthase 2 |
| PG-12 | ALOX5 | P09917 | Arachidonate 5-lipoxygenase |
| PG-12 | ALOX15 | P16050 | Arachidonate 15-lipoxygenase |
| PG-12 | ALOX12 | P18054 | Arachidonate 12-lipoxygenase, 12S-type |
| PG-12 | ALOX15B | O15296 | Arachidonate 15-lipoxygenase B |
| PG-12 | ALOX12B | O75342 | Arachidonate 12-lipoxygenase, 12S-type |
| PG-12 | ALOXE3 | Q9BYJ1 | Epidermis-type lipoxygenase 3 |
| PG-12 | MBNL1 | Q9NR56 | Muscleblind-like protein 1 |
| PG-12 | MBNL2 | Q5VZF2 | Muscleblind-like protein 2 |
| PG-12 | MBNL3 | Q9NUK0 | Muscleblind-like protein 3 |
| PG-12 | EP300 | Q09472 | Histone acetyltransferase p300 |
| PG-12 | CREBBP | Q92793 | CREB-binding protein |
| PG-12 | CNR1 | P21554 | Cannabinoid receptor 1 |
| PG-12 | TDP1 | Q9NUW8 | Tyrosyl-DNA phosphodiesterase 1 |
| PG-12 | XBP1 | P17861 | X-box-binding protein 1 |
| PG-12 | [SMARCA2](http://zinc15.docking.org/genes/SMARCA2) | P51531 | Probable global transcription activator SNF2L2 |
| PG-12 | GLI2 | P10070 | Zinc finger protein GLI2 |
| PG-12 | [GABRA6](http://zinc15.docking.org/genes/GABRA6) | Q16445 | Gamma-aminobutyric acid receptor subunit alpha-6 |
| PG-12 | TLR4 | O00206 | Toll-like receptor 4 |
| PG-12 | GLI1 | P08151 | Zinc finger protein GLI1 |
| PG-13 | PTGS1 | P23219 | Prostaglandin G/H synthase 1 |
| PG-13 | PTGS2 | P35354 | Prostaglandin G/H synthase 2 |
| PG-13 | TDP1 | Q9NUW8 | Tyrosyl-DNA phosphodiesterase 1 |
| PG-13 | MAPK11 | Q15759 | Mitogen-activated protein kinase 11 |
| PG-13 | MAPK14 | Q16539 | Mitogen-activated protein kinase 14 |
| PG-13 | F9 | P00740 | Coagulation factor IXa heavy chain |
| PG-13 | F10 | P00742 | Factor X light chain |
| PG-13 | F7 | P08709 | Coagulation factor VII |
| PG-13 | ATR | Q13535 | Serine/threonine-protein kinase ATR |
| PG-13 | F2 | P00734 | Activation peptide fragment 1 |
| PG-13 | MAPK8 | P45983 | Mitogen-activated protein kinase 8 |
| PG-13 | MAPK9 | P45984 | Mitogen-activated protein kinase 9 |
| PG-13 | MAPK10 | P53779 | Mitogen-activated protein kinase 10 |
| PG-13 | MAPT | P10636 | Microtubule-associated protein tau |
| PG-13 | NR3C1 | P04150 | Glucocorticoid receptor |
| PG-13 | MT-ND4 | P03905 | NADH-ubiquinone oxidoreductase chain 4 |
| PG-13 | NR0B1 | [P51843](https://www.uniprot.org/uniprot/P51843) | Nuclear receptor subfamily 0 group B member 1 |
| PG-13 | [SLC22A3](http://zinc15.docking.org/genes/SLC22A3) | O75751 | Solute carrier family 22 member 3 |
| PG-14 | ABCB1 | P08183 | Multidrug resistance protein 1 |
| PG-14 | ABCB11 | O95342 | Bile salt export pump |
| PG-14 | ABCB4 | P21439 | Multidrug resistance protein 3 |
| PG-14 | ABCB5 | Q2M3G0 | ATP-binding cassette sub-family B member 5 |
| PG-14 | PDE4B | Q07343 | cAMP-specific 3', 5'-cyclic phosphodiesterase 4B |
| PG-14 | PDE4D | Q08499 | cAMP-specific 3', 5'-cyclic phosphodiesterase 4D |
| PG-14 | PDE4A | P27815 | cAMP-specific 3', 5'-cyclic phosphodiesterase 4A |
| PG-14 | PDE4C | Q08493 | cAMP-specific 3', 5'-cyclic phosphodiesterase 4C |
| PG-14 | SLC6A2 | P23975 | Sodium-dependent noradrenaline transporter |
| PG-14 | SLC6A3 | Q01959 | Sodium-dependent dopamine transporter |
| PG-14 | MGLL | Q99685 | Monoglyceride lipase |
| PG-14 | BCHE | P06276 | Cholinesterase |
| PG-14 | ACHE | P22303 | Acetylcholinesterase |
| PG-14 | PRKCG | P05129 | Protein kinase C gamma type |
| PG-14 | PRKCB | P05771 | Protein kinase C beta type |
| PG-14 | [LGALS1](http://zinc15.docking.org/genes/LGALS1) | P09382 | Galectin-1 |
| PG-14 | [TUBB3](http://zinc15.docking.org/genes/TUBB3) | Q13509 | Tubulin beta-3 chain |
| PG-14 | [LGALS9](http://zinc15.docking.org/genes/LGALS1) | [O00182](https://www.uniprot.org/uniprot/O00182) | Galectin-9 |
| PG-15 | MAPT | P10636 | Microtubule-associated protein tau |
| PG-15 | CYP51A1 | Q16850 | Lanosterol 14-alpha demethylase |
| PG-15 | DHH | O43323 | Desert hedgehog protein C-product |
| PG-15 | IHH | Q14623 | Indian hedgehog protein N-product |
| PG-15 | SHH | Q15465 | Sonic hedgehog protein C-product |
| PG-15 | UGT1A4 | P22310 | UDP-glucuronosyltransferase 1-4 |
| PG-15 | UGT1A9 | O60656 | UDP-glucuronosyltransferase 1-9 |
| PG-15 | UGT1A6 | P19224 | UDP-glucuronosyltransferase 1-6 |
| PG-15 | UGT1A1 | P22309 | UDP-glucuronosyltransferase 1-1 |
| PG-15 | UGT1A3 | P35503 | UDP-glucuronosyltransferase 1-3 |
| PG-15 | UGT1A5 | P35504 | UDP-glucuronosyltransferase 1-5 |
| PG-15 | UGT1A7 | Q9HAW7 | UDP-glucuronosyltransferase 1-7 |
| PG-15 | UGT1A10 | Q9HAW8 | UDP-glucuronosyltransferase 1-10 |
| PG-15 | UGT1A8 | Q9HAW9 | UDP-glucuronosyltransferase 1-8 |
| PG-15 | CYP19A1 | P11511 | Cytochrome P450 19A1 |
| PG-15 | CD81 | P60033 | CD81 antigen |
| PG-15 | 1L1B | P01584 | Interleukin-1 beta |
| PG-15 | [AKR1B10](http://zinc15.docking.org/genes/AKR1B10) | O60218 | Aldo-keto reductase family 1 member B10 |
| PG-15 | PTPN1 | P18031 | Tyrosine-protein phosphatase non-receptor type 1 |
| PG-15 | [HSD11B2](http://zinc15.docking.org/genes/HSD11B2) | P80365 | Corticosteroid 11-beta-dehydrogenase isozyme 2 |
| PG-15 | [PTPN2](http://zinc15.docking.org/genes/PTPN2) | P17706 | Tyrosine-protein phosphatase non-receptor type 2 |
| PG-15 | GPBAR1 | Q8TDU6 | G-protein coupled bile acid receptor 1 |
| PG-16 | DYRK1A | Q13627 | Dual specificity tyrosine-phosphorylation-regulated kinase 1A |
| PG-16 | MAPT | P10636 | Microtubule-associated protein tau |
| PG-16 | ODC1 | P11926 | Ornithine decarboxylase |
| PG-16 | EDNRB | P24530 | Endothelin B receptor |
| PG-16 | EDNRA | P25101 | Endothelin-1 receptor |
| PG-16 | PRKCG | P05129 | Protein kinase C gamma type |
| PG-16 | PRKCB | P05771 | Protein kinase C beta type |
| PG-16 | PRKCA | P17252 | Protein kinase C alpha type |
| PG-16 | PRKCQ | Q04759 | Protein kinase C theta type |
| PG-16 | PRKCD | Q05655 | Protein kinase C delta type regulatory subunit |
| PG-16 | MBNL1 | Q9NR56 | Muscleblind-like protein 1 |
| PG-16 | MBNL2 | Q5VZF2 | Muscleblind-like protein 2 |
| PG-16 | MBNL3 | Q9NUK0 | Muscleblind-like protein 3 |
| PG-16 | NR3C1 | P04150 | Glucocorticoid receptor |
| PG-16 | NR3C2 | P08235 | Mineralocorticoid receptor |
| PG-16 | ACHE | P22303 | Acetylcholinesterase |
| PG-16 | CDK5R1 | Q15078 | Cyclin-dependent kinase 5 activator 1 |
| PG-16 | CDK5 | Q00535 | Cyclin-dependent-like kinase 5 |
| PG-16 | CYP1A1 | P04798 | Cytochrome P450 1A1 |
| PG-16 | CYP1B1 | Q16678 | Cytochrome P450 1B1 |
| PG-16 | CYP3A4 | P08684 | Cytochrome P450 3A4 |
| PG-16 | DYRK2 | Q92630 | Dual specificity tyrosine-phosphorylation-regulated kinase 2 |
| PG-16 | DYRK3 | O43781 | Dual specificity tyrosine-phosphorylation-regulated kinase 3 |
| PG-16 | ENPP2 | Q13822 | Ectonucleotide pyrophosphatase/phosphodiesterase family member 2 |
| PG-16 | ESRRA | P11474 | Steroid hormone receptor ERR1 |
| PG-16 | F5 | P12259 | Coagulation factor V |
| PG-16 | HCN4 | Q9Y3Q4 | Potassium/sodium hyperpolarization-activated cyclic nucleotide-gated channel 4 |
| PG-16 | LYPLA1 | O75608 | Acyl-protein thioesterase 1 |
| PG-16 | LYPLA2 | O95372 | Acyl-protein thioesterase 2 |
| PG-16 | NR0B1 | P51843 | Nuclear receptor subfamily 0 group B member 1 |
| PG-16 | HPGD | P15428 | 15-hydroxyprostaglandin dehydrogenase [NAD(+)] |
| PG-16 | PIM1 | P11309 | Serine/threonine-protein kinase pim-1 |
| PG-16 | SHBG | P04278 | Sex hormone-binding globulin |
| PG-16 | TAAR1 | Q96RJ0 | Trace amine-associated receptor 1 |
| PG-16 | F3 | P13726 | Tissue factor |
| PG-16 | TNFRSF1A | P19438 | Tumor necrosis factor receptor superfamily member 1A |
| PG-17 | HTR7 | P34969 | 5-hydroxytryptamine receptor 7 |
| PG-17 | DRD2 | P14416 | D(2) dopamine receptor |
| PG-17 | DRD1 | P21728 | D(1A) dopamine receptor |
| PG-17 | DRD5 | P21918 | D(1B) dopamine receptor |
| PG-17 | CHRM2 | P08172 | Muscarinic acetylcholine receptor M2 |
| PG-17 | CHRM4 | P08173 | Muscarinic acetylcholine receptor M4 |
| PG-17 | CHRM5 | P08912 | Muscarinic acetylcholine receptor M5 |
| PG-17 | CHRM1 | P11229 | Muscarinic acetylcholine receptor M1 |
| PG-17 | CHRM3 | P20309 | Muscarinic acetylcholine receptor M3 |
| PG-17 | SLC6A2 | P23975 | Sodium-dependent noradrenaline transporter |
| PG-17 | SLC6A3 | Q01959 | Sodium-dependent dopamine transporter |
| PG-17 | HTR1A | P08908 | 5-hydroxytryptamine receptor 1A |
| PG-17 | HTR1B | P28222 | 5-hydroxytryptamine receptor 1B |
| PG-17 | MBNL1 | Q9NR56 | Muscleblind-like protein 1 |
| PG-17 | MBNL2 | Q5VZF2 | Muscleblind-like protein 2 |
| PG-17 | F2 | P00734 | Prothrombin |
| PG-17 | KIAA010 | Q15004 | PCNA-associated factor |
| PG-17 | HRH1 | P35367 | Histamine H1 receptor |
| PG-17 | TRPC3 | Q13507 | Short transient receptor potential channel 3 |
| PG-17 | TRPC6 | [Q9Y210](https://www.uniprot.org/uniprot/Q9Y210) | Short transient receptor potential channel 6 |
| PG-17 | F3 | P13726 | Tissue factor |
| PG-17 | NR4A2 | P43354 | Nuclear receptor subfamily 4 group A member 2 |
| PG-17 | NR0B1 | [P51843](https://www.uniprot.org/uniprot/P51843) | Nuclear receptor subfamily 0 group B member 1 |
| PG-18 | OPRM1 | P35372 | Mu-type opioid receptor |
| PG-18 | OPRD1 | P41143 | Delta-type opioid receptor |
| PG-18 | OPRK1 | P41145 | Kappa-type opioid receptor |
| PG-18 | OPRL1 | P41146 | Nociceptin receptor |
| PG-18 | AR | P10275 | Androgen receptor |
| PG-18 | ESR1 | P03372 | Estrogen receptor |
| PG-18 | ESR2 | Q92731 | Estrogen receptor beta |
| PG-18 | PGR | P06401 | Progesterone receptor |
| PG-18 | PLA2G1B | P04054 | Phospholipase A2 |
| PG-18 | PLA2G2A | P14555 | Phospholipase A2, membrane associated |
| PG-18 | PLA2G5 | P39877 | Calcium-dependent phospholipase A2 |
| PG-18 | PLA2G2F | Q9BZM2 | Group IIF secretory phospholipase A2 |
| PG-18 | PLA2G2E | Q9NZK7 | Group IIE secretory phospholipase A2 |
| PG-18 | PLA2G2D | Q9UNK4 | Group IID secretory phospholipase A2 |
| PG-18 | CNR1 | P21554 | Cannabinoid receptor 1 |
| PG-18 | FAAH | O00519 | Fatty-acid amide hydrolase 1 |
| PG-18 | FABP3 | P05413 | Fatty acid-binding protein, heart |
| PG-18 | HMGCR | P04035 | 3-hydroxy-3-methylglutaryl-coenzyme A reductase |
| PG-18 | LPAR1 | Q92633 | Lysophosphatidic acid receptor 1 |
| PG-18 | LPAR2 | Q9HBW0 | Lysophosphatidic acid receptor 2 |
| PG-18 | LPAR3 | Q9UBY5 | Lysophosphatidic acid receptor 3 |
| PG-18 | LPAR4 | Q99677 | Lysophosphatidic acid receptor 4 |
| PG-18 | LPAR5 | Q9H1C0 | Lysophosphatidic acid receptor 5 |
| PG-18 | LPAR6 | P43657 | Lysophosphatidic acid receptor 6 |
| PG-18 | OXER1 | Q8TDS5 | Oxoeicosanoid receptor 1 |
| PG-18 | PLA2G4C | Q9UP65 | Cytosolic phospholipase A2 gamma |
| PG-19 | NR1H2 | P55055 | Oxysterols receptor LXR-beta |
| PG-19 | NR1H3 | Q13133 | Oxysterols receptor LXR-alpha |
| PG-19 | SLC6A2 | P23975 | Sodium-dependent noradrenaline transporter |
| PG-19 | SLC6A4 | P31645 | Sodium-dependent serotonin transporter |
| PG-19 | SLC6A9 | P48067 | Sodium- and chloride-dependent glycine transporter 1 |
| PG-19 | SLC6A3 | Q01959 | Sodium-dependent dopamine transporter |
| PG-19 | SLC6A5 | Q9Y345 | Sodium- and chloride-dependent glycine transporter 2 |
| PG-19 | SLC6A7 | Q99884 | Sodium-dependent proline transporter |
| PG-19 | SLC6A14 | Q9UN76 | Sodium- and chloride-dependent neutral and basic amino acid transporter B(0+) |
| PG-19 | LDLR | P01130 | Low-density lipoprotein receptor |
| PG-19 | VLDLR | P98155 | Very low-density lipoprotein receptor |
| PG-19 | LRP8 | Q14114 | Low-density lipoprotein receptor-related protein 8 |
| PG-19 | ATP12A | P54707 | Potassium-transporting ATPase alpha chain 2 |
| PG-19 | ATP1A1 | P05023 | Sodium/potassium-transporting ATPase subunit alpha-1 |
| PG-19 | ATP1A3 | P13637 | Sodium/potassium-transporting ATPase subunit alpha-3 |
| PG-19 | CD81 | P60033 | CD81 antigen |
| PG-19 | PLCG1 | P19174 | 1-phosphatidylinositol 4,5-bisphosphate phosphodiesterase gamma-1 |
| PG-19 | 1L1B | P01584 | Interleukin-1 beta |
| PG-19 | [AKR1B10](http://zinc15.docking.org/genes/AKR1B10) | O60218 | Aldo-keto reductase family 1 member B10 |
| PG-19 | PTPN1 | P18031 | Tyrosine-protein phosphatase non-receptor type 1 |
| PG-19 | PTPN2 | P17706 | Tyrosine-protein phosphatase non-receptor type 2 |
| PG-19 | [HSD11B2](http://zinc15.docking.org/genes/HSD11B2) | P80365 | Corticosteroid 11-beta-dehydrogenase isozyme 2 |
| PG-19 | RBL2 | Q08999 | Retinoblastoma-like protein 2 |
| PG-19 | RHAG | Q02094 | Ammonium transporter Rh type A |
| PG-19 | MVD | P53602 | Diphosphomevalonate decarboxylase |
| PG-20 | MBNL1 | Q9NR56 | Muscleblind-like protein 1 |
| PG-20 | MBNL2 | Q5VZF2 | Muscleblind-like protein 2 |
| PG-20 | MBNL3 | Q9NUK0 | Muscleblind-like protein 3 |
| PG-20 | STAT3 | P40763 | Signal transducer and activator of transcription 3 |
| PG-20 | STAT1 | P42224 | Signal transducer and activator of transcription 1-alpha/beta |
| PG-20 | STAT2 | P52630 | Signal transducer and activator of transcription 2 |
| PG-20 | STAT4 | Q14765 | Signal transducer and activator of transcription 4 |
| PG-20 | CHRM5 | P08912 | Muscarinic acetylcholine receptor M5 |
| PG-20 | CHRM1 | P11229 | Muscarinic acetylcholine receptor M1 |
| PG-20 | CHRM3 | P20309 | Muscarinic acetylcholine receptor M3 |
| PG-20 | CHRM2 | P08172 | Muscarinic acetylcholine receptor M2 |
| PG-20 | CHRM4 | P08173 | Muscarinic acetylcholine receptor M4 |
| PG-20 | F2 | P00734 | Activation peptide fragment 1 |
| PG-20 | PTPN2 | P17706 | Tyrosine-protein phosphatase non-receptor type 2 |
| PG-20 | PTPN1 | P18031 | Tyrosine-protein phosphatase non-receptor type 1 |
| PG-20 | LGALS4 | P56470 | Galectin-4 |
| PG-20 | LGALS8 | O00214 | Galectin-8 |
| PG-20 | IL2 | P60568 | Interleukin-2 |
| PG-20 | LGALS3 | P17931 | Galectin-3 |
| PG-21 | HSD11B1 | P28845 | Corticosteroid 11-beta-dehydrogenase isozyme 1 |
| PG-21 | HSD11B2 | P80365 | Corticosteroid 11-beta-dehydrogenase isozyme 2 |
| PG-21 | HSD11B1L | Q7Z5J1 | Hydroxysteroid 11-beta-dehydrogenase 1-like protein |
| PG-21 | F2 | P00734 | Activation peptide fragment 1 |
| PG-21 | BCL2L1 | Q07817 | Bcl-2-like protein 1 |
| PG-21 | BCL2 | P10415 | Apoptosis regulator Bcl-2 |
| PG-21 | BCL2L2 | Q92843 | Bcl-2-like protein 2 |
| PG-21 | GLI1 | P08151 | Zinc finger protein GLI1 |
| PG-21 | GLI2 | P10070 | Zinc finger protein GLI2 |
| PG-21 | GLI3 | P10071 | Transcriptional repressor GLI3R |
| PG-21 | PTAFR | P25105 | Platelet-activating factor receptor |
| PG-21 | JUN | P05412 | Transcription factor AP-1 |
| PG-21 | JUNB | P17275 | Transcription factor jun-B |
| PG-21 | JUND | P17535 | Transcription factor jun-D |
| PG-21 | PTPN2 | P17706 | Tyrosine-protein phosphatase non-receptor type 2 |
| PG-21 | CD81 | P60033 | CD81 antigen |
| PG-21 | PTPN1 | P18031 | Tyrosine-protein phosphatase non-receptor type 1 |
| PG-21 | FGF1 | P05230 | Fibroblast growth factor 1 |
| PG-21 | FGF2 | P09038 | Fibroblast growth factor 2 |
| PG-21 | PTPN6 | P29350 | Tyrosine-protein phosphatase non-receptor type 6 |
| PG-21 | PLCG1 | P19174 | 1-phosphatidylinositol 4,5-bisphosphate phosphodiesterase gamma-1 |
| PG-21 | VEGFA | P15692 | Vascular endothelial growth factor A |
| PG-21 | [AKR1B10](http://zinc15.docking.org/genes/AKR1B10) | O60218 | Aldo-keto reductase family 1 member B10 |
| PG-21 | ACP1 | P24666 | Low molecular weight phosphotyrosine protein phosphatase |
| PG-21 | [SLCO1B1](http://zinc15.docking.org/genes/SLCO1B1) | Q9Y6L6 | Solute carrier organic anion transporter family member 1B1 |
| PG-21 | POLB | P06746 | DNA polymerase beta |
| PG-21 | [LGALS3](http://zinc15.docking.org/genes/LGALS3) | P17931 | Galectin-3 |
| PG-22 | STAT3 | P40763 | Signal transducer and activator of transcription 3 |
| PG-22 | STAT1 | P42224 | Signal transducer and activator of transcription 1-alpha/beta |
| PG-22 | STAT2 | P52630 | Signal transducer and activator of transcription 2 |
| PG-22 | STAT4 | Q14765 | Signal transducer and activator of transcription 4 |
| PG-22 | PPM1B | O75688 | Protein phosphatase 1B |
| PG-22 | PTPN1 | P18031 | Tyrosine-protein phosphatase non-receptor type 1 |
| PG-22 | PPM1A | P35813 | Protein phosphatase 1A |
| PG-22 | PPP1CC | P36873 | Serine/threonine-protein phosphatase PP1-gamma catalytic subunit |
| PG-22 | PPP2CA | P67775 | Serine/threonine-protein phosphatase 2A catalytic subunit alpha isoform |
| PG-22 | PPP2R5A | Q15172 | Serine/threonine-protein phosphatase 2A 56 kDa regulatory subunit alpha isoform |
| PG-22 | PTPN2 | P17706 | Tyrosine-protein phosphatase non-receptor type 2 |
| PG-22 | PPP1CA | P62136 | Serine/threonine-protein phosphatase PP1-alpha catalytic subunit |
| PG-22 | PPP1CB | P62140 | Serine/threonine-protein phosphatase PP1-beta catalytic subunit |
| PG-22 | PPP4C | P60510 | Serine/threonine-protein phosphatase 4 catalytic subunit |
| PG-22 | PPP2CB | P62714 | Serine/threonine-protein phosphatase 2A catalytic subunit beta isoform |
| PG-22 | AMY2A | P04746 | Pancreatic alpha-amylase |
| PG-22 | FGF1 | P05230 | Fibroblast growth factor 1 |
| PG-22 | FGF2 | P09038 | Fibroblast growth factor 2 |
| PG-23 | STAT3 | P40763 | Signal transducer and activator of transcription 3 |
| PG-23 | STAT1 | P42224 | Signal transducer and activator of transcription 1-alpha/beta |
| PG-23 | STAT2 | P52630 | Signal transducer and activator of transcription 2 |
| PG-23 | STAT4 | Q14765 | Signal transducer and activator of transcription 4 |
| PG-23 | FGF1 | P05230 | Fibroblast growth factor 1 |
| PG-23 | FGF2 | P09038 | Fibroblast growth factor 2 |
| PG-23 | VEGFA | P15692 | Vascular endothelial growth factor A |
| PG-23 | HPSE | Q9Y251 | Heparanase 8 kDa subunit |
| PG-23 | HPSE2 | Q8WWQ2 | Inactive heparanase-2 |
| PG-23 | CHRM5 | P08912 | Muscarinic acetylcholine receptor M5 |
| PG-23 | CHRM1 | P11229 | Muscarinic acetylcholine receptor M1 |
| PG-23 | CHRM3 | P20309 | Muscarinic acetylcholine receptor M3 |
| PG-23 | CHRM2 | P08172 | Muscarinic acetylcholine receptor M2 |
| PG-23 | CHRM4 | P08173 | Muscarinic acetylcholine receptor M4 |
| PG-23 | MAPT | P10636 | Microtubule-associated protein tau |
| PG-23 | AMY2A | P04746 | Pancreatic alpha-amylase |
| PG-23 | LGALS4 | P56470 | Galectin-4 |
| PG-23 | LGALS8 | O00214 | Galectin-8 |
| PG-23 | VEGFA | P15692 | Vascular endothelial growth factor A |
| PG-24 | STAT3 | P40763 | Signal transducer and activator of transcription 3 |
| PG-24 | STAT1 | P42224 | Signal transducer and activator of transcription 1-alpha/beta |
| PG-24 | STAT2 | P52630 | Signal transducer and activator of transcription 2 |
| PG-24 | STAT4 | Q14765 | Signal transducer and activator of transcription 4 |
| PG-24 | CHRM5 | P08912 | Muscarinic acetylcholine receptor M5 |
| PG-24 | CHRM1 | P11229 | Muscarinic acetylcholine receptor M1 |
| PG-24 | CHRM3 | P20309 | Muscarinic acetylcholine receptor M3 |
| PG-24 | CHRM2 | P08172 | Muscarinic acetylcholine receptor M2 |
| PG-24 | CHRM4 | P08173 | Muscarinic acetylcholine receptor M4 |
| PG-24 | FGF1 | P05230 | Fibroblast growth factor 1 |
| PG-24 | FGF2 | P09038 | Fibroblast growth factor 2 |
| PG-24 | VEGFA | P15692 | Vascular endothelial growth factor A |
| PG-24 | HPSE | Q9Y251 | Heparanase 8 kDa subunit |
| PG-24 | HPSE2 | Q8WWQ2 | Inactive heparanase-2 |
| PG-24 | FLT1 | P17948 | Vascular endothelial growth factor receptor 1 |
| PG-24 | AMY2A | P04746 | Pancreatic alpha-amylase |
| PG-24 | PRKAA1 | Q13131 | 5'-AMP-activated protein kinase catalytic subunit alpha-1 |
| PG-24 | STK11 | Q15831 | Serine/threonine-protein kinase STK11 |
| PG-24 | NOS3 | P29474 | Nitric oxide synthase, endothelial |
| PG-25 | STAT3 | P40763 | Signal transducer and activator of transcription 3 |
| PG-25 | STAT1 | P42224 | Signal transducer and activator of transcription 1-alpha/beta |
| PG-25 | STAT2 | P52630 | Signal transducer and activator of transcription 2 |
| PG-25 | STAT4 | Q14765 | Signal transducer and activator of transcription 4 |
| PG-25 | CDK1 | P06493 | Cyclin-dependent kinase 1 |
| PG-25 | CDK2 | P24941 | Cyclin-dependent kinase 2 |
| PG-25 | CDK4 | P11802 | Cyclin-dependent kinase 4 |
| PG-25 | CDK3 | Q00526 | Cyclin-dependent kinase 3 |
| PG-25 | CDK6 | Q00534 | Cyclin-dependent kinase 6 |
| PG-25 | FGF1 | P05230 | Fibroblast growth factor 1 |
| PG-25 | FGF2 | P09038 | Fibroblast growth factor 2 |
| PG-25 | VEGFA | P15692 | Vascular endothelial growth factor A |
| PG-25 | HPSE | Q9Y251 | Heparanase 8 kDa subunit |
| PG-25 | HPSE2 | Q8WWQ2 | Inactive heparanase-2 |
| PG-25 | CHRM5 | P08912 | Muscarinic acetylcholine receptor M5 |
| PG-25 | VEGFA | P15692 | Vascular endothelial growth factor A |
| PG-26 | STAT3 | P40763 | Signal transducer and activator of transcription 3 |
| PG-26 | STAT1 | P42224 | Signal transducer and activator of transcription 1-alpha/beta |
| PG-26 | STAT2 | P52630 | Signal transducer and activator of transcription 2 |
| PG-26 | STAT4 | Q14765 | Signal transducer and activator of transcription 4 |
| PG-26 | MBNL1 | Q9NR56 | Muscleblind-like protein 1 |
| PG-26 | MBNL2 | Q5VZF2 | Muscleblind-like protein 2 |
| PG-26 | MBNL3 | Q9NUK0 | Muscleblind-like protein 3 |
| PG-26 | PTAFR | P25105 | Platelet-activating factor receptor |
| PG-26 | FGF1 | P05230 | Fibroblast growth factor 1 |
| PG-26 | FGF2 | P09038 | Fibroblast growth factor 2 |
| PG-26 | VEGFA | P15692 | Vascular endothelial growth factor A |
| PG-26 | HPSE | Q9Y251 | Heparanase 8 kDa subunit |
| PG-26 | HPSE2 | Q8WWQ2 | Inactive heparanase-2 |
| PG-26 | F2 | P00734 | Activation peptide fragment 1 |
| PG-26 | CHRM5 | P08912 | Muscarinic acetylcholine receptor M5 |
| PG-26 | MMP9 | [P14780](https://www.uniprot.org/uniprot/P14780) | Matrix metalloproteinase-9 |
| PG-26 | BDNF | [P23560](https://www.uniprot.org/uniprot/P23560) | Brain-derived neurotrophic factor |
| PG-26 | CHRNA4 | P43681 | Neuronal acetylcholine receptor subunit alpha-4 |
| PG-26 | CHRNA2 | Q15822 | Neuronal acetylcholine receptor subunit alpha-2 |
| PG-26 | CHRNA7 | P36544 | Neuronal acetylcholine receptor subunit alpha-7 |
| PG-26 | CHRFAM7A | Q494W8 | CHRNA7-FAM7A fusion protein |
| PG-26 | RB1 | P06400 | Retinoblastoma-associated protein |
| PG-26 | RBL2 | [Q08999](https://www.uniprot.org/uniprot/Q08999) | Retinoblastoma-like protein 2 |
| PG-26 | RHAG | [Q02094](https://www.uniprot.org/uniprot/Q02094) | Ammonium transporter Rh type A |
| PG-27 | HMGCR | P04035 | 3-hydroxy-3-methylglutaryl-coenzyme A reductase |
| PG-27 | OPRM1 | P35372 | Mu-type opioid receptor |
| PG-27 | OPRD1 | P41143 | Delta-type opioid receptor |
| PG-27 | OPRK1 | P41145 | Kappa-type opioid receptor |
| PG-27 | OPRL1 | P41146 | Nociceptin receptor |
| PG-27 | UPP2 | O95045 | Uridine phosphorylase 2 |
| PG-27 | UPP1 | Q16831 | Uridine phosphorylase 1 |
| PG-27 | NR4A2 | P43354 | Nuclear receptor subfamily 4 group A member 2 |
| PG-27 | NR4A1 | P22736 | Nuclear receptor subfamily 4 group A member 1 |
| PG-27 | NR4A3 | Q92570 | Nuclear receptor subfamily 4 group A member 3 |
| PG-27 | ABL1 | P00519 | Tyrosine-protein kinase ABL1 |
| PG-27 | ABL2 | P42684 | Abelson tyrosine-protein kinase 2 |
| PG-27 | SLC5A1 | P13866 | Sodium/glucose cotransporter 1 |
| PG-27 | SLC5A2 | P31639 | Sodium/glucose cotransporter 2 |
| PG-27 | SLC5A4 | Q9NY91 | Low affinity sodium-glucose cotransporter |
| PG-28 | PTAFR | P25105 | Platelet activating factor receptor |
| PG-28 | STAT3 | P40763 | Signal transducer and activator of transcription 3 |
| PG-28 | IL2 | P60568 | Interleukin-2 |
| PG-28 | VEGFA | P15692 | Vascular endothelial growth factor A |
| PG-28 | FGF1 | P05230 | Acidic fibroblast growth factor |
| PG-28 | FGF2 | P09038 | Basic fibroblast growth factor |
| PG-28 | HPSE | Q9Y251 | Heparanase |
| PG-28 | ATP1A1 | P05023 | Sodium/potassium-transporting ATPase alpha-1 chain |
| PG-28 | BCL2L1 | Q07817 | Apoptosis regulator Bcl-X |
| PG-28 | HSD11B2 | P80365 | 11-beta-hydroxysteroid dehydrogenase 2 |
| PG-28 | TACR2 | P21452 | Neurokinin 2 receptor |
| PG-28 | GRB2 | P62993 | Growth factor receptor-bound protein 2 |
| PG-28 | AMY2A | P04746 | Pancreatic alpha-amylase |
| PG-28 | PSEN2 | P49810 | Presenilin-2 |
| PG-28 | PSENEN | Q9NZ42 | Gamma-secretase subunit PEN-2 |
| PG-28 | NCSTN | Q92542 | Nicastrin |
| PG-28 | APH1A | Q96BI3 | Gamma-secretase subunit APH-1A |
| PG-28 | PSEN1 | P49768 | Presenilin-1 |
| PG-28 | APH1B | Q8WW43 | Gamma-secretase subunit APH-1B |
| PG-29 | STAT3 | P40763 | Signal transducer and activator of transcription 3 |
| PG-29 | PTAFR | P25105 | Platelet activating factor receptor |
| PG-29 | IL2 | P60568 | Interleukin-2 |
| PG-29 | VEGFA | P15692 | Vascular endothelial growth factor A |
| PG-29 | FGF1 | P05230 | Acidic fibroblast growth factor |
| PG-29 | HPSE | Q9Y251 | Heparanase |
| PG-29 | RORC | P51449 | Nuclear receptor ROR-gamma |
| PG-29 | ATP1A1 | P05023 | Sodium/potassium-transporting ATPase alpha-1 chain |
| PG-29 | LGALS4 | P56470 | Galectin-4 |
| PG-29 | LGALS3 | P17931 | Galectin-3 |
| PG-29 | LGALS8 | O00214 | Galectin-8 |
| PG-29 | CDK1 | P06493 | Cyclin-dependent kinase 1 |
| PG-29 | BCL2L1 | Q07817 | Apoptosis regulator Bcl-X |
| PG-29 | HSD11B2 | P80365 | 11-beta-hydroxysteroid dehydrogenase 2 |
| PG-29 | HSD11B1 | P28845 | 11-beta-hydroxysteroid dehydrogenase 1 |
| PG-29 | PSEN2 | P49810 | Presenilin-2 |
| PG-29 | PSENEN | Q9NZ42 | Gamma-secretase subunit PEN-2 |
| PG-29 | NCSTN | Q92542 | Nicastrin |
| PG-29 | APH1A | Q96BI3 | Gamma-secretase subunit APH-1A |
| PG-29 | PSEN1 | P49768 | Presenilin-1 |
| PG-29 | APH1B | Q8WW43 | Gamma-secretase subunit APH-1B |
| PG-29 | FGF2 | P09038 | Fibroblast growth factor 2 |
| PG-29 | LGALS9 | O00182 | Galectin-9 |
| PG-30 | ACHE | P22303 | Acetylcholinesterase |
| PG-30 | ADK | P55263 | Adenosine kinase (by homology) |
| PG-30 | ADORA1 | P30542 | Adenosine A1 receptor (by homology) |
| PG-30 | ADORA2A | P29274 | Adenosine A2a receptor |
| PG-30 | ADORA2B | P29275 | Adenosine A2b receptor |
| PG-30 | ADRA1D | P25100 | Alpha-1d adrenergic receptor |
| PG-30 | ALOX5 | P09917 | Arachidonate 5-lipoxygenase |
| PG-30 | APP | P05067 | Beta amyloid A4 protein |
| PG-30 | AR | P10275 | Androgen Receptor |
| PG-30 | ATM | Q13315 | Serine-protein kinase ATM |
| PG-30 | BRD2 | P25440 | Bromodomain-containing protein 2 |
| PG-30 | BRD3 | Q15059 | Bromodomain-containing protein 3 |
| PG-30 | BRD4 | O60885 | Bromodomain-containing protein 4 |
| PG-30 | CA1 | P00915 | Carbonic anhydrase I |
| PG-30 | CA12 | O43570 | Carbonic anhydrase XII |
| PG-30 | CA13 | Q8N1Q1 | Carbonic anhydrase XIII |
| PG-30 | CA14 | Q9ULX7 | Carbonic anhydrase XIV |
| PG-30 | CA2 | P00918 | Carbonic anhydrase II |
| PG-30 | CA3 | P07451 | Carbonic anhydrase III |
| PG-30 | CA4 | P22748 | Carbonic anhydrase IV |
| PG-30 | CA5A | P35218 | Carbonic anhydrase VA |
| PG-30 | CA5B | Q9Y2D0 | Carbonic anhydrase VB |
| PG-30 | CA6 | P23280 | Carbonic anhydrase VI |
| PG-30 | CA7 | P43166 | Carbonic anhydrase VII |
| PG-30 | CA9 | Q16790 | Carbonic anhydrase IX |
| PG-30 | CASR | P41180 | Calcium sensing receptor |
| PG-30 | CCKBR | P32239 | Cholecystokinin B receptor |
| PG-30 | CCND3 | P30281 | G1/S-specific cyclin-D3 |
| PG-30 | CCND1 | P24385 | G1/S-specific cyclin-D1 |
| PG-30 | CDK4 | P11802 | Cyclin-dependent kinase 4 |
| PG-30 | CCND2 | P30279 | G1/S-specific cyclin-D2 |
| PG-30 | CDC25A | P30304 | Dual specificity phosphatase Cdc25A |
| PG-30 | CDC25B | P30305 | Dual specificity phosphatase Cdc25B |
| PG-30 | CHRM1 | P11229 | Muscarinic acetylcholine receptor M1 |
| PG-30 | CHRM2 | P08172 | Muscarinic acetylcholine receptor M2 |
| PG-30 | CHRM3 | P20309 | Muscarinic acetylcholine receptor M3 |
| PG-30 | CHRM4 | P08173 | Muscarinic acetylcholine receptor M4 |
| PG-30 | CHRM5 | P08912 | Muscarinic acetylcholine receptor M5 |
| PG-30 | CTSS | P25774 | Cathepsin S |
| PG-30 | CYP11B1 | P15538 | Cytochrome P450 11B1 |
| PG-30 | CYP11B2 | P19099 | Cytochrome P450 11B2 |
| PG-30 | CYP19A1 | P11511 | Cytochrome P450 19A1 |
| PG-30 | CYP1A1 | P04798 | Cytochrome P450 1A1 |
| PG-30 | CYP1A2 | P05177 | Cytochrome P450 1A2 |
| PG-30 | CYP1B1 | Q16678 | Cytochrome P450 1B1 |
| PG-30 | DRD1 | P21728 | Dopamine D1 receptor |
| PG-30 | DRD5 | P21918 | Dopamine D5 receptor |
| PG-30 | EDNRA | P25101 | Endothelin receptor ET-A (by homology) |
| PG-30 | EGFR | P00533 | Epidermal growth factor receptor erbB1 |
| PG-30 | EPHX1 | P07099 | Epoxide hydrolase 1 |
| PG-30 | ESR2 | Q92731 | Estrogen receptor beta |
| PG-30 | GABRA1 | P14867 | GABA receptor alpha-1 subunit |
| PG-30 | GABRG2 | P18507 | Gamma-aminobutyric acid receptor subunit gamma-2 |
| PG-30 | GABRA2 | P47869 | Gamma-aminobutyric acid receptor subunit alpha-2 |
| PG-30 | GABRB3 | P28472 | Gamma-aminobutyric acid receptor subunit beta-3 |
| PG-30 | GABRA5 | P31644 | GABA receptor alpha-5 subunit |
| PG-30 | GABRA6 | Q16445 | GABA receptor alpha-6 subunit |
| PG-30 | GABRA3 | P34903 | Gamma-aminobutyric acid receptor subunit alpha-3 |
| PG-30 | GABRA4 | P48169 | Gamma-aminobutyric acid receptor subunit alpha-4 |
| PG-30 | GAK | O14976 | Serine/threonine-protein kinase GAK |
| PG-30 | GRM1 | Q13255 | Metabotropic glutamate receptor 1 |
| PG-30 | GRM5 | P41594 | Metabotropic glutamate receptor 5 |
| PG-30 | GSK3B | P49841 | Glycogen synthase kinase-3 beta |
| PG-30 | HSD11B1 | P28845 | 11-beta-hydroxysteroid dehydrogenase 1 |
| PG-30 | HSD17B2 | P37059 | Estradiol 17-beta-dehydrogenase 2 |
| PG-30 | HTR2C | P28335 | Serotonin 2c (5-HT2c) receptor |
| PG-30 | JAK2 | O60674 | Tyrosine-protein kinase JAK2 |
| PG-30 | JAK3 | P52333 | Tyrosine-protein kinase JAK3 |
| PG-30 | KCNA5 | P22460 | Voltage-gated potassium channel subunit Kv1.5 |
| PG-30 | KCNMA1 | Q12791 | Calcium-activated potassium channel subunit alpha-1 |
| PG-30 | LIPE | Q05469 | Hormone sensitive lipase |
| PG-30 | MAPK14 | Q16539 | MAP kinase p38 alpha |
| PG-30 | MCHR1 | Q99705 | Melanin-concentrating hormone receptor 1 |
| PG-30 | MIF | P14174 | Macrophage migration inhibitory factor |
| PG-30 | MKNK1 | Q9BUB5 | MAP kinase-interacting serine/threonine-protein kinase MNK1 |
| PG-30 | NTRK1 | P04629 | Nerve growth factor receptor Trk-A |
| PG-30 | NTRK2 | Q16620 | Neurotrophic tyrosine kinase receptor type 2 |
| PG-30 | NTRK3 | Q16288 | NT-3 growth factor receptor |
| PG-30 | P2RX7 | Q99572 | P2X purinoceptor 7 |
| PG-30 | PABPC1 | P11940 | Polyadenylate-binding protein 1 |
| PG-30 | PDE10A | Q9Y233 | Phosphodiesterase 10A |
| PG-30 | PDE4B | Q07343 | Phosphodiesterase 4B |
| PG-30 | PDE4D | Q08499 | Phosphodiesterase 4D |
| PG-30 | PIK3CA | P42336 | PI3-kinase p110-alpha subunit |
| PG-30 | PIK3CB | P42338 | PI3-kinase p110-beta subunit |
| PG-30 | PIK3CD | O00329 | PI3-kinase p110-delta subunit |
| PG-30 | PIK3CG | P48736 | PI3-kinase p110-gamma subunit |
| PG-30 | PIM1 | P11309 | Serine/threonine-protein kinase PIM1 |
| PG-30 | PIM2 | Q9P1W9 | Serine/threonine-protein kinase PIM2 |
| PG-30 | PLA2G7 | Q13093 | LDL-associated phospholipase A2 |
| PG-30 | PLEC | Q15149 | Plectin |
| PG-30 | PPARA | Q07869 | Peroxisome proliferator-activated receptor alpha |
| PG-30 | PRKDC | P78527 | DNA-dependent protein kinase |
| PG-30 | PSEN1 | P49768 | Presenilin 1 |
| PG-30 | PSEN2 | P49810 | Presenilin 2 |
| PG-30 | PTGER3 | P43115 | Prostanoid EP3 receptor |
| PG-30 | RAPGEF4 | Q8WZA2 | Rap guanine nucleotide exchange factor 4 |
| PG-30 | SCN5A | Q14524 | Sodium channel protein type V alpha subunit |
| PG-30 | SCN9A | Q15858 | Sodium channel protein type IX alpha subunit |
| PG-30 | SLC1A3 | P43003 | Excitatory amino acid transporter 1 |
| PG-30 | TERT | O14746 | Telomerase reverse transcriptase |
| PG-30 | TGFBR1 | P36897 | TGF-beta receptor type I |
| PG-30 | TLR9 | Q9NR96 | Toll-like receptor (TLR7/TLR9) |
| PG-30 | HTR3A | P46098 | 5-hydroxytryptamine receptor 3A |
| PG-30 | SLC18A2 | Q05940 | Synaptic vesicular amine transporter |
| PG-30 | LGALS9 | O00182 | Galectin-9 |
| PG-30 | LGALS1 | P09382 | Galectin-1 |
| PG-30 | CHRNA7 | P36544 | Neuronal acetylcholine receptor subunit alpha-7 |
| PG-31 | AR | P10275 | Androgen Receptor |
| PG-31 | NR1H3 | Q13133 | LXR-alpha |
| PG-31 | HMGCR | P04035 | HMG-CoA reductase |
| PG-31 | CYP51A1 | Q16850 | Cytochrome P450 51 (by homology) |
| PG-31 | NPC1L1 | Q9UHC9 | Niemann-Pick C1-like protein 1 |
| PG-31 | CYP17A1 | P05093 | Cytochrome P450 17A1 |
| PG-31 | RORC | P51449 | Nuclear receptor ROR-gamma |
| PG-31 | CYP19A1 | P11511 | Cytochrome P450 19A1 |
| PG-31 | ESR1 | P03372 | Estrogen receptor alpha |
| PG-31 | SHBG | P04278 | Testis-specific androgen-binding protein |
| PG-31 | ESR2 | Q92731 | Estrogen receptor beta |
| PG-31 | SREBF2 | Q12772 | Sterol regulatory element-binding protein 2 |
| PG-31 | SLC6A2 | P23975 | Norepinephrine transporter |
| PG-31 | CHRM2 | P08172 | Muscarinic acetylcholine receptor M2 |
| PG-31 | CYP2C19 | P33261 | Cytochrome P450 2C19 |
| PG-31 | RORA | P35398 | Nuclear receptor ROR-alpha |
| PG-31 | PTPN1 | P18031 | Protein-tyrosine phosphatase 1B |
| PG-31 | SERPINA6 | P08185 | Corticosteroid binding globulin |
| PG-31 | BCHE | P06276 | Butyrylcholinesterase |
| PG-31 | ABCB11 | O95342 | Bile salt export pump |
| PG-31 | CA1 | P00915 | Carbonic anhydrase 1 |
| PG-31 | CA2 | P00918 | Carbonic anhydrase 2 |
| PG-31 | CD4 | P01730 | T-cell surface glycoprotein CD4 |
| PG-31 | CDC45 | O75419 | Cell division control protein 45 homolog |
| PG-31 | CYP24A1 | Q07973 | 1,25-dihydroxyvitamin D(3) 24-hydroxylase, mitochondrial |
| PG-31 | CYP27B1 | O15528 | 25-hydroxyvitamin D-1 alpha hydroxylase, mitochondrial |
| PG-31 | POLA1 | P09884 | DNA polymerase alpha catalytic subunit |
| PG-31 | EBP | Q15125 | 3-beta-hydroxysteroid-Delta(8),Delta(7)-isomerase |
| PG-31 | ENPP2 | Q13822 | Ectonucleotide pyrophosphatase/phosphodiesterase family member 2 |
| PG-31 | EPHA1 | P21709 | Ephrin type-A receptor 1 |
| PG-31 | EPHA2 | P29317 | Ephrin type-A receptor 2 |
| PG-31 | EPHA4 | P54764 | Ephrin type-A receptor 4 |
| PG-31 | EPHA5 | P54756 | Ephrin type-A receptor 5 |
| PG-31 | EPHA6 | Q9UF33 | Ephrin type-A receptor 6 |
| PG-31 | EPHA7 | Q15375 | Ephrin type-A receptor 7 |
| PG-31 | EPHA8 | P29322 | Ephrin type-A receptor 8 |
| PG-31 | EPHB1 | P54762 | Ephrin type-B receptor 1 |
| PG-31 | EPHB2 | P29323 | Ephrin type-B receptor 2 |
| PG-31 | EPHB3 | P54753 | Ephrin type-B receptor 3 |
| PG-31 | FGF1 | P05230 | Fibroblast growth factor 1 |
| PG-31 | G6PD | P11413 | Glucose-6-phosphate 1-dehydrogenase |
| PG-31 | GBA2 | Q9HCG7 | Non-lysosomal glucosylceramidase |
| PG-31 | GPBAR1 | Q8TDU6 | G-protein coupled bile acid receptor 1 |
| PG-31 | IL2 | P60568 | Interleukin-2 |
| PG-31 | CDC25A | P30304 | M-phase inducer phosphatase 1 |
| PG-31 | CDC25B | P30305 | M-phase inducer phosphatase 2 |
| PG-31 | ABCC4 | O15439 | Multidrug resistance-associated protein 4 |
| PG-31 | NR1H2 | P55055 | Oxysterols receptor LXR-beta |
| PG-31 | NR1H4 | Q96RI1 | Bile acid receptor |
| PG-31 | SLC10A2 | Q12908 | Ileal sodium/bile acid cotransporter |
| PG-31 | SLC10A1 | Q14973 | Sodium/bile acid cotransporter |
| PG-31 | ATIC | P31939 | Bifunctional purine biosynthesis protein PURH |
| PG-31 | SLC22A3 | O75751 | Solute carrier family 22 member 3 |
| PG-31 | SRD5A1 | P18405 | 3-oxo-5-alpha-steroid 4-dehydrogenase 1 |
| PG-31 | SRD5A2 | P31213 | 3-oxo-5-alpha-steroid 4-dehydrogenase 2 |
| PG-31 | SHH | Q15465 | Sonic hedgehog protein |
| PG-31 | ST3GAL1 | Q11201 | CMP-N-acetylneuraminate-beta-galactosamide-alpha-2,3-sialyltransferase 1 |
| PG-31 | VDR | P11473 | Vitamin D3 receptor |
| PG-31 | GC | P02774 | Vitamin D-binding protein |
| PG-32 | AMY2A | P04746 | Pancreatic alpha-amylase |
| PG-32 | PTAFR | P25105 | Platelet activating factor receptor |
| PG-32 | IL2 | P60568 | Interleukin-2 |
| PG-32 | STAT3 | P40763 | Signal transducer and activator of transcription 3 |
| PG-32 | VEGFA | P15692 | Vascular endothelial growth factor A |
| PG-32 | FGF1 | P05230 | Acidic fibroblast growth factor |
| PG-32 | FGF2 | P09038 | Basic fibroblast growth factor |
| PG-32 | HPSE | Q9Y251 | Heparanase |
| PG-32 | HSP90AA1 | P07900 | Heat shock protein HSP 90-alpha |
| PG-32 | LGALS4 | P56470 | Galectin-4 |
| PG-32 | LGALS3 | P17931 | Galectin-3 |
| PG-32 | LGALS8 | O00214 | Galectin-8 |
| PG-32 | BCL2L1 | Q07817 | Apoptosis regulator Bcl-X |
| PG-32 | CDK1 | P06493 | Cyclin-dependent kinase 1 |
| PG-32 | HSD11B2 | P80365 | 11-beta-hydroxysteroid dehydrogenase 2 |
| PG-32 | HSD11B1 | P28845 | 11-beta-hydroxysteroid dehydrogenase 1 |
| PG-32 | ATP1A1 | P05023 | Sodium/potassium-transporting ATPase alpha-1 chain |
| PG-32 | PSEN2 | P49810 | Presenilin-2 |
| PG-32 | PSENEN | Q9NZ42 | Gamma-secretase subunit PEN-2 |
| PG-32 | NCSTN | Q92542 | Nicastrin |
| PG-32 | APH1A | Q96BI3 | Gamma-secretase subunit APH-1A |
| PG-32 | PSEN1 | P49768 | Presenilin-1 |
| PG-32 | APH1B | Q8WW43 | Gamma-secretase subunit APH-1B |
| PG-33 | STAT3 | P40763 | Signal transducer and activator of transcription 3 |
| PG-33 | IL2 | P60568 | Interleukin-2 |
| PG-33 | PTAFR | P25105 | Platelet activating factor receptor |
| PG-33 | VEGFA | P15692 | Vascular endothelial growth factor A |
| PG-33 | FGF1 | P05230 | Acidic fibroblast growth factor |
| PG-33 | FGF2 | P09038 | Basic fibroblast growth factor |
| PG-33 | HPSE | Q9Y251 | Heparanase |
| PG-33 | ATP1A1 | P05023 | Sodium/potassium-transporting ATPase alpha-1 chain |
| PG-33 | HSP90AA1 | P07900 | Heat shock protein HSP 90-alpha |
| PG-33 | TYMS | P04818 | Thymidylate synthase (by homology) |
| PG-33 | TACR2 | P21452 | Neurokinin 2 receptor |
| PG-33 | ITGAV | P06756 | Integrin alpha-V |
| PG-33 | ITGB3 | P05106 | Integrin beta-3 |
| PG-33 | ITGA2B ITGB3 | P08514 P05106 | Integrin alpha-IIb/beta-3 |
| PG-33 | LGALS4 | P56470 | Galectin-4 |
| PG-33 | LGALS3 | P17931 | Galectin-3 |
| PG-33 | LGALS8 | O00214 | Galectin-8 |
| PG-34 | NDUFA4 | O00483 | Cytochrome c oxidase subunit NDUFA4 |
| PG-34 | NR0B1 | P51843 | Nuclear receptor subfamily 0 group B member 1 |
| PG-34 | GSTP1 | P09211 | Glutathione S-transferase P |
| PG-34 | GSTA4 | O15217 | Glutathione S-transferase A4 |
| PG-34 | GSTA3 | Q16772 | Glutathione S-transferase A3 |
| PG-34 | GSTA5 | Q7RTV2 | Glutathione S-transferase A5 |
| PG-34 | GSTA2 | P09210 | Glutathione S-transferase A2 |
| PG-34 | CASP3 | P42574 | Caspase-3 |
| PG-34 | GSTA1 | P08263 | Glutathione S-transferase A1 |
| PG-34 | HPGDS | O60760 | Hematopoietic prostaglandin D synthase |
| PG-34 | ABCB1 | P08183 | P-glycoprotein 1 |
| PG-34 | ABCC9 | O60706 | Sulfonylurea receptor 2 |
| PG-34 | ADAM10 | O14672 | ADAM10 |
| PG-34 | ADAM17 | P78536 | ADAM17 |
| PG-34 | ADORA2A | P29274 | Adenosine A2a receptor |
| PG-34 | ADORA3 | P0DMS8 | Adenosine A3 receptor |
| PG-34 | ALOX5 | P09917 | Arachidonate 5-lipoxygenase |
| PG-34 | ALOX5AP | P20292 | 5-lipoxygenase activating protein |
| PG-34 | AR | P10275 | Androgen Receptor |
| PG-34 | ATR | Q13535 | Serine-protein kinase ATR |
| PG-34 | AURKA | O14965 | Serine/threonine-protein kinase Aurora-A |
| PG-34 | AXL | P30530 | Tyrosine-protein kinase receptor UFO |
| PG-34 | BRPF1 | P55201 | Peregrin |
| PG-34 | CCNA2 | P20248 | Cyclin-A2 |
| PG-34 | CDK2 | P24941 | Cyclin-dependent kinase 2 |
| PG-34 | CCND1 | P24385 | G1/S-specific cyclin-D1 |
| PG-34 | CDK4 | P11802 | Cyclin-dependent kinase 4 |
| PG-34 | CCNE1 | P24864 | G1/S-specific cyclin-E1 |
| PG-34 | CCNE2 | O96020 | G1/S-specific cyclin-E2 |
| PG-34 | CDK1 | P06493 | Cyclin-dependent kinase 1 |
| PG-34 | CCNA1 | P78396 | Cyclin-A1 |
| PG-34 | CDK5R1 | Q15078 | Cyclin-dependent kinase 5 activator 1 |
| PG-34 | CDK5 | Q00535 | Cyclin-dependent-like kinase 5 |
| PG-34 | CFD | P00746 | Complement factor D |
| PG-34 | CHEK1 | O14757 | Serine/threonine-protein kinase Chk1 |
| PG-34 | CXCR2 | P25025 | Interleukin-8 receptor B |
| PG-34 | DUT | P33316 | dUTP pyrophosphatase |
| PG-34 | EGFR | P00533 | Epidermal growth factor receptor erbB1 |
| PG-34 | ERBB2 | P04626 | Receptor protein-tyrosine kinase erbB-2 |
| PG-34 | F10 | P00742 | Thrombin and coagulation factor X |
| PG-34 | F2 | P00734 | Thrombin |
| PG-34 | FKBP1A | P62942 | FK506-binding protein 1A |
| PG-34 | FLT3 | P36888 | Tyrosine-protein kinase receptor FLT3 |
| PG-34 | GABRA2 | P47869 | Gamma-aminobutyric acid receptor subunit alpha-2 |
| PG-34 | GABRB3 | P28472 | Gamma-aminobutyric acid receptor subunit beta-3 |
| PG-34 | GABRG2 | P18507 | Gamma-aminobutyric acid receptor subunit gamma-2 |
| PG-34 | GABRA3 | P34903 | Gamma-aminobutyric acid receptor subunit alpha-3 |
| PG-34 | GABRA1 | P14867 | Gamma-aminobutyric acid receptor subunit alpha-1 |
| PG-34 | GABRA5 | P31644 | Gamma-aminobutyric acid receptor subunit alpha-5 |
| PG-34 | GCK | P35557 | Hexokinase type IV |
| PG-34 | GPR139 | Q6DWJ6 | Probable G-protein coupled receptor 139 |
| PG-34 | GPR88 | Q9GZN0 | Probable G-protein coupled receptor 88 |
| PG-34 | GSK3B | P49841 | Glycogen synthase kinase-3 beta |
| PG-34 | HCRTR2 | O43614 | Orexin receptor 2 |
| PG-34 | HDAC4 | P56524 | Histone deacetylase 4 |
| PG-34 | HMGCR | P04035 | HMG-CoA reductase (by homology) |
| PG-34 | HSD17B2 | P37059 | Estradiol 17-beta-dehydrogenase 2 |
| PG-34 | IGF1R | P08069 | Insulin-like growth factor I receptor |
| PG-34 | JAK1 | P23458 | Tyrosine-protein kinase JAK1 |
| PG-34 | JAK2 | O60674 | Tyrosine-protein kinase JAK2 |
| PG-34 | JAK3 | P52333 | Tyrosine-protein kinase JAK3 |
| PG-34 | KCNH2 | Q12809 | HERG |
| PG-34 | KDR | P35968 | Vascular endothelial growth factor receptor 2 |
| PG-34 | KIF11 | P52732 | Kinesin-like protein 1 |
| PG-34 | LIMK2 | P53671 | LIM domain kinase 2 |
| PG-34 | LRRK2 | Q5S007 | Leucine-rich repeat serine/threonine-protein kinase 2 |
| PG-34 | LTB4R | Q15722 | Leukotriene B4 receptor 1 |
| PG-34 | MAP3K20 | Q9NYL2 | Mixed lineage kinase 7 |
| PG-34 | MAPK1 | P28482 | MAP kinase ERK2 |
| PG-34 | MAPK11 | Q15759 | MAP kinase p38 beta |
| PG-34 | MAPK14 | Q16539 | MAP kinase p38 alpha |
| PG-34 | MAPK8 | P45983 | c-Jun N-terminal kinase 1 |
| PG-34 | MDM2 | Q00987 | p53-binding protein Mdm-2 |
| PG-34 | MERTK | Q12866 | Proto-oncogene tyrosine-protein kinase MER |
| PG-34 | MET | P08581 | Hepatocyte growth factor receptor |
| PG-34 | MMP13 | P45452 | Matrix metalloproteinase 13 |
| PG-34 | MMP14 | P50281 | Matrix metalloproteinase 14 |
| PG-34 | MMP2 | P08253 | Matrix metalloproteinase 2 |
| PG-34 | MMP7 | P09237 | Matrix metalloproteinase 7 |
| PG-34 | MTOR | P42345 | Serine/threonine-protein kinase mTOR |
| PG-34 | NPY5R | Q15761 | Neuropeptide Y receptor type 5 |
| PG-34 | NQO1 | P15559 | Quinone reductase 1 |
| PG-34 | NQO2 | P16083 | Quinone reductase 2 |
| PG-34 | NTRK1 | P04629 | Nerve growth factor receptor Trk-A |
| PG-34 | OPRL1 | P41146 | Nociceptin receptor |
| PG-34 | P2RX3 | P56373 | P2X purinoceptor 3 |
| PG-34 | P2RX7 | Q99572 | P2X purinoceptor 7 |
| PG-34 | PAK1 | Q13153 | Serine/threonine-protein kinase PAK 1 |
| PG-34 | PARP1 | P09874 | Poly [ADP-ribose] polymerase-1 |
| PG-34 | PDE10A | Q9Y233 | Phosphodiesterase 10A |
| PG-34 | PDE2A | O00408 | Phosphodiesterase 2A |
| PG-34 | PDE5A | O76074 | Phosphodiesterase 5A |
| PG-34 | PDE7A | Q13946 | Phosphodiesterase 7A |
| PG-34 | PIK3CA | P42336 | PI3-kinase p110-alpha subunit |
| PG-34 | PIK3CB | P42338 | PI3-kinase p110-beta subunit |
| PG-34 | PIK3CD | O00329 | PI3-kinase p110-delta subunit |
| PG-34 | PIK3CG | P48736 | PI3-kinase p110-gamma subunit |
| PG-34 | PRKCA | P17252 | Protein kinase C alpha |
| PG-34 | PRKCB | P05771 | Protein kinase C beta |
| PG-34 | PRKDC | P78527 | DNA-dependent protein kinase |
| PG-34 | PSEN2 | P49810 | Presenilin-2 |
| PG-34 | PSENEN | Q9NZ42 | Gamma-secretase subunit PEN-2 |
| PG-34 | NCSTN | Q92542 | Nicastrin |
| PG-34 | APH1A | Q96BI3 | Gamma-secretase subunit APH-1A |
| PG-34 | PSEN1 | P49768 | Presenilin-1 |
| PG-34 | APH1B | Q8WW43 | Gamma-secretase subunit APH-1B |
| PG-34 | PTGS1 | P23219 | Cyclooxygenase-1 |
| PG-34 | ROCK1 | Q13464 | Rho-associated protein kinase 1 |
| PG-34 | ROCK2 | O75116 | Rho-associated protein kinase 2 |
| PG-34 | RPS6KA2 | Q15349 | Ribosomal protein S6 kinase alpha 2 |
| PG-34 | SYK | P43405 | Tyrosine-protein kinase SYK |
| PG-34 | TACR1 | P25103 | Neurokinin 1 receptor |
| PG-34 | TGFBR1 | P36897 | TGF-beta receptor type I |
| PG-34 | TGFBR2 | P37173 | TGF-beta receptor type II |
| PG-34 | THRA | P10827 | Thyroid hormone receptor alpha |
| PG-34 | THRB | P10828 | Thyroid hormone receptor beta-1 |
| PG-34 | TK1 | P04183 | Thymidine kinase, cytosolic |
| PG-34 | TNF | P01375 | TNF-alpha |
| PG-34 | TYRO3 | Q06418 | Tyrosine-protein kinase receptor TYRO3 |
| AC-01 | MAPT | P10636 | Microtubule-associated protein tau |
| AC-01 | GGCX | P38435 | Vitamin K-dependent gamma-carboxylase |
| AC-01 | CDC25A | P30304 | M-phase inducer phosphatase 1 |
| AC-01 | CDC25B | P30305 | M-phase inducer phosphatase 2 |
| AC-01 | MAOA | P21397 | Amine oxidase [flavin-containing] A |
| AC-01 | MAOB | P27338 | Amine oxidase [flavin-containing] B |
| AC-01 | TDP1 | Q9NUW8 | Tyrosyl-DNA phosphodiesterase 1 |
| AC-01 | NQO1 | P15559 | NAD(P)H dehydrogenase [quinone] 1 |
| AC-01 | NQO2 | P16083 | Ribosyldihydronicotinamide dehydrogenase [quinone] |
| AC-01 | MAP2K1 | Q02750 | Dual specificity mitogen-activated protein kinase kinase 1 |
| AC-01 | MAP2K2 | P36507 | Dual specificity mitogen-activated protein kinase kinase 2 |
| AC-01 | MAP2K5 | Q13163 | Dual specificity mitogen-activated protein kinase kinase 5 |
| AC-01 | MBNL1 | Q9NR56 | Muscleblind-like protein 1 |
| AC-01 | MBNL2 | Q5VZF2 | Muscleblind-like protein 2 |
| AC-01 | MBNL3 | Q9NUK0 | Muscleblind-like protein 3 |
| AC-01 | APAF1 | O14727 | Apoptotic protease-activating factor 1 |
| AC-01 | GLRA3 | O75311 | Glycine receptor subunit alpha-3 |
| AC-01 | GSR | P00390 | Glutathione reductase, mitochondrial |
| AC-01 | APEX1 | P27695 | DNA-(apurinic or apyrimidinic site) lyase |
| AC-01 | PTPN13 | Q12923 | Tyrosine-protein phosphatase non-receptor type 13 |
| AC-02 | GBA | P04062 | Glucosylceramidase |
| AC-02 | TDP1 | Q9NUW8 | Tyrosyl-DNA phosphodiesterase 1 |
| AC-02 | CHRM1 | P11229 | Muscarinic acetylcholine receptor M1 |
| AC-02 | CHRM2 | P08172 | Muscarinic acetylcholine receptor M2 |
| AC-02 | CHRM4 | P08173 | Muscarinic acetylcholine receptor M4 |
| AC-02 | CHRM5 | P08912 | Muscarinic acetylcholine receptor M5 |
| AC-02 | CHRM3 | P20309 | Muscarinic acetylcholine receptor M3 |
| AC-02 | FGF1 | P05230 | Fibroblast growth factor 1 |
| AC-02 | FGF2 | P09038 | Fibroblast growth factor 2 |
| AC-02 | VEGFA | P15692 | Vascular endothelial growth factor A |
| AC-02 | HPSE | Q9Y251 | Heparanase 8 kDa subunit |
| AC-02 | HPSE2 | Q8WWQ2 | Inactive heparanase-2 |
| AC-02 | CA12 | O43570 | Carbonic anhydrase 12 |
| AC-02 | CA1 | P00915 | Carbonic anhydrase 1 |
| AC-02 | CA2 | P00918 | Carbonic anhydrase 2 |
| AC-02 | MGAM | O43451 | Maltase-glucoamylase, intestinal |
| AC-02 | SI | P14410 | Sucrase-isomaltase, intestinal |
| AC-02 | SLC2A5 | P22732 | Solute carrier family 2, facilitated glucose transporter member 5 |
| AC-02 | GANC | Q8TET4 | Neutral alpha-glucosidase C |
| AC-02 | HK1 | P19367 | Hexokinase-1 |
| AC-02 | KHK | P50053 | Ketohexokinase |
| AC-02 | HKDC1 | Q2TB90 | Putative hexokinase HKDC1 |
| AC-02 | HK2 | P52789 | Hexokinase-2 |
| AC-02 | HK3 | P52790 | Hexokinase-3 |
| AC-02 | GAA | P10253 | Lysosomal alpha-glucosidase |
| AC-03 | TDP1 | Q9NUW8 | Tyrosyl-DNA phosphodiesterase 1 |
| AC-03 | GBA | P04062 | Glucosylceramidase |
| AC-03 | CHRM1 | P11229 | Muscarinic acetylcholine receptor M1 |
| AC-03 | CHRM2 | P08172 | Muscarinic acetylcholine receptor M2 |
| AC-03 | CHRM4 | P08173 | Muscarinic acetylcholine receptor M4 |
| AC-03 | CHRM5 | P08912 | Muscarinic acetylcholine receptor M5 |
| AC-03 | CHRM3 | P20309 | Muscarinic acetylcholine receptor M3 |
| AC-03 | CA12 | O43570 | Carbonic anhydrase 12 |
| AC-03 | CA1 | P00915 | Carbonic anhydrase 1 |
| AC-03 | CA2 | P00918 | Carbonic anhydrase 2 |
| AC-03 | CA9 | Q16790 | Carbonic anhydrase 9 |
| AC-03 | CA14 | Q9ULX7 | Carbonic anhydrase 14 |
| AC-03 | CA3 | P07451 | Carbonic anhydrase 3 |
| AC-03 | CA5A | P35218 | Carbonic anhydrase 5A, mitochondrial |
| AC-03 | CA7 | P43166 | Carbonic anhydrase 7 |
| AC-03 | ADA | P00813 | Adenosine deaminase |
| AC-03 | ADK | P55263 | Adenosine kinase |
| AC-03 | GLA | P06280 | Alpha-galactosidase A |
| AC-03 | AMY2A | P04746 | Pancreatic alpha-amylase |
| AC-03 | GLB1 | P16278 | Beta-galactosidase |
| AC-03 | CDA | P32320 | Cytidine deaminase |
| AC-03 | FGF1 | P05230 | Fibroblast growth factor 1 |
| AC-03 | FGF2 | P09038 | Fibroblast growth factor 2 |
| AC-03 | GANC | Q8TET4 | Neutral alpha-glucosidase C |
| AC-03 | AGL | P35573 | Glycogen debranching enzyme |
| AC-03 | HSPA5 | P11021 | 78 kDa glucose-regulated protein |
| AC-03 | HEXA | P06865 | Beta-hexosaminidase subunit alpha |
| AC-03 | HEXB | P07686 | Beta-hexosaminidase subunit beta |
| AC-03 | LGALS3 | P17931 | Galectin-3 |
| AC-03 | LGALS4 | P56470 | Galectin-4 |
| AC-03 | LGALS8 | O00214 | Galectin-8 |
| AC-03 | MGAM | O43451 | Maltase-glucoamylase, intestinal |
| AC-03 | MGEA5 | O60502 | Protein O-GlcNAcase |
| AC-03 | PDCD4 | Q53EL6 | Programmed cell death protein 4 |
| AC-03 | PYGB | P11216 | Glycogen phosphorylase, brain form |
| AC-03 | PYGM | P11217 | Glycogen phosphorylase, muscle form |
| AC-03 | SLC5A2 | P31639 | Sodium/glucose cotransporter 2 |
| AC-03 | SLC5A4 | Q9NY91 | Low affinity sodium-glucose cotransporter |
| AC-03 | TYR | P14679 | Tyrosinase |
| AC-03 | VEGFA | P15692 | Vascular endothelial growth factor A |
| AC-03 | G6PC | P35575 | Glucose-6-phosphatase |
| AC-03 | GAA | P10253 | Lysosomal alpha-glucosidase |
| AC-03 | SI | P14410 | Sucrase-isomaltase, intestinal |
| AC-03 | ALB | P02768 | Serum albumin |
| AC-03 | SLC2A2 | P11168 | Solute carrier family 2, facilitated glucose transporter member 2 |
| AC-03 | GCK | P35557 | Glucokinase |
| AC-03 | IRS1 | P35568 | Insulin receptor substrate 1 |
| AC-04 | CDK1 | P06493 | Cyclin-dependent kinase 1 |
| AC-04 | CDK4 | P11802 | Cyclin-dependent kinase 4 |
| AC-04 | CDK2 | P24941 | Cyclin-dependent kinase 2 |
| AC-04 | CDK3 | Q00526 | Cyclin-dependent kinase 3 |
| AC-04 | CDK6 | Q00534 | Cyclin-dependent kinase 6 |
| AC-04 | FGF1 | P05230 | Fibroblast growth factor 1 |
| AC-04 | FGF2 | P09038 | Fibroblast growth factor 2 |
| AC-04 | CHRM1 | P11229 | Muscarinic acetylcholine receptor M1 |
| AC-04 | CHRM2 | P08172 | Muscarinic acetylcholine receptor M2 |
| AC-04 | CHRM4 | P08173 | Muscarinic acetylcholine receptor M4 |
| AC-04 | CHRM5 | P08912 | Muscarinic acetylcholine receptor M5 |
| AC-04 | CHRM3 | P20309 | Muscarinic acetylcholine receptor M3 |
| AC-04 | VEGFA | P15692 | Vascular endothelial growth factor A |
| AC-04 | HPSE | Q9Y251 | Heparanase 8 kDa subunit |
| AC-04 | HPSE2 | Q8WWQ2 | Inactive heparanase-2 |
| AC-04 | SLC3A2 | P08195 | 4F2 cell-surface antigen heavy chain |
| AC-04 | GANC | Q8TET4 | Neutral alpha-glucosidase C |
| AC-04 | GYG1 | P46976 | Glycogenin-1 |
| AC-04 | AMY2B | P19961 | Alpha-amylase 2B |
| AC-04 | SLC3A1 | Q07837 | Neutral and basic amino acid transport protein rBAT |
| AC-04 | AMY1A; AMY1B; AMY1C | P04745 | Alpha-amylase 1 |
| AC-04 | AMY2A | P04746 | Pancreatic alpha-amylase |
| AC-04 | B4GALT1 | P15291 | Beta-1,4-galactosyltransferase 1 |
| AC-04 | GLB1 | P16278 | Beta-galactosidase |
| AC-04 | CA13 | Q8N1Q1 | Carbonic anhydrase 13 |
| AC-04 | CA14 | Q9ULX7 | Carbonic anhydrase 14 |
| AC-04 | CA12 | O43570 | Carbonic anhydrase 12 |
| AC-04 | CA1 | P00915 | Carbonic anhydrase 1 |
| AC-04 | CA2 | P00918 | Carbonic anhydrase 2 |
| AC-04 | CA9 | Q16790 | Carbonic anhydrase 9 |
| AC-04 | CDA | P32320 | Cytidine deaminase |
| AC-04 | CHIA | Q9BZP6 | Acidic mammalian chitinase |
| AC-04 | HEXA | P06865 | Beta-hexosaminidase subunit alpha |
| AC-04 | HEXB | P07686 | Beta-hexosaminidase subunit beta |
| AC-04 | IL2 | P60568 | Interleukin-2 |
| AC-04 | LGALS3 | P17931 | Galectin-3 |
| AC-04 | LGALS4 | P56470 | Galectin-4 |
| AC-04 | LGALS7; LGALS7B | P47929 | Galectin-7 |
| AC-04 | LGALS9 | O00182 | Galectin-9 |
| AC-04 | LGALS8 | O00214 | Galectin-8 |
| AC-04 | GAA | P10253 | Lysosomal alpha-glucosidase |
| AC-04 | MAG | P20916 | Myelin-associated glycoprotein |
| AC-04 | MGAM | O43451 | Maltase-glucoamylase, intestinal |
| AC-04 | IGF2R | P11717 | Cation-independent mannose-6-phosphate receptor |
| AC-04 | MGEA5 | O60502 | Protein O-GlcNAcase |
| AC-04 | P2RY14 | Q15391 | P2Y purinoceptor 14 |
| AC-04 | P4HB | P07237 | Protein disulfide-isomerase |
| AC-04 | SLC28A3 | Q9HAS3 | Solute carrier family 28 member 3 |
| AC-04 | SLC5A1 | P13866 | Sodium/glucose cotransporter 1 |
| AC-04 | SLC5A2 | P31639 | Sodium/glucose cotransporter 2 |
| AC-04 | SLC5A4 | Q9NY91 | Low affinity sodium-glucose cotransporter |
| AC-04 | SI | P14410 | Sucrase-isomaltase, intestinal |
| AC-04 | TYR | P14679 | Tyrosinase |
| AC-05 | PDXK | O00764 | Pyridoxal kinase |
| AC-05 | TDP1 | Q9NUW8 | Tyrosyl-DNA phosphodiesterase 1 |
| AC-05 | MAPT | P10636 | Microtubule-associated protein tau |
| AC-05 | ADORA2A | P29274 | Adenosine receptor A2a |
| AC-05 | ADORA1 | P30542 | Adenosine receptor A1 |
| AC-05 | ADORA2B | P29275 | Adenosine receptor A2b |
| AC-05 | DYRK1A | Q13627 | Dual specificity tyrosine-phosphorylation-regulated kinase 1A |
| AC-05 | KDM4A | O75164 | Lysine-specific demethylase 4A |
| AC-05 | KDM4C | Q9H3R0 | Lysine-specific demethylase 4C |
| AC-05 | KDM4B | O94953 | Lysine-specific demethylase 4B |
| AC-05 | MBNL1 | Q9NR56 | Muscleblind-like protein 1 |
| AC-05 | MBNL2 | Q5VZF2 | Muscleblind-like protein 2 |
| AC-05 | MBNL3 | Q9NUK0 | Muscleblind-like protein 3 |
| AC-05 | CA9 | Q16790 | Carbonic anhydrase 9 |
| AC-05 | KDM4E | B2RXH2 | Lysine-specific demethylase 4E |
| AC-05 | PNPO | Q9NVS9 | Pyridoxine-5'-phosphate oxidase |
| AC-05 | P2RX1 | P51575 | P2X purinoceptor 1 |
| AC-05 | TLR8 | Q9NR97 | Toll-like receptor 8 |
| AC-05 | ALAS2 | P22557 | 5-aminolevulinate synthase, erythroid-specific, mitochondrial |
| AC-05 | ADGRE1 | Q14246 | Adhesion G protein-coupled receptor E1 |
| AC-05 | PPP1R14B | Q96C90 | Protein phosphatase 1 regulatory subunit 14B |
| AC-05 | MSR1 | P21757 | Macrophage scavenger receptor types I and II |
| AC-05 | PHOSPHO2 | Q8TCD6 | Pyridoxal phosphate phosphatase PHOSPHO2 |
| AC-05 | PDXP | Q96GD0 | Pyridoxal phosphate phosphatase |
| AC-05 | PNPO | Q9NVS9 | Pyridoxine-5'-phosphate oxidase |
| AC-05 | DDC | P20711 | Aromatic-L-amino-acid decarboxylase |
| AC-05 | GOT1 | P17174 | Aspartate aminotransferase, cytoplasmic |
| AC-06 | CHRM2 | P08172 | Muscarinic acetylcholine receptor M2 |
| AC-06 | CHRM4 | P08173 | Muscarinic acetylcholine receptor M4 |
| AC-06 | CHRM5 | P08912 | Muscarinic acetylcholine receptor M5 |
| AC-06 | CHRM1 | P11229 | Muscarinic acetylcholine receptor M1 |
| AC-06 | CHRM3 | P20309 | Muscarinic acetylcholine receptor M3 |
| AC-06 | CHRNA7 | P36544 | Neuronal acetylcholine receptor subunit alpha-7 |
| AC-06 | CHRFAM7A | Q494W8 | CHRNA7-FAM7A fusion protein |
| AC-06 | BCHE | P06276 | Cholinesterase |
| AC-06 | ACHE | P22303 | Acetylcholinesterase |
| AC-06 | DRD4 | P21917 | D(4) dopamine receptor |
| AC-06 | TDP1 | Q9NUW8 | Tyrosyl-DNA phosphodiesterase 1 |
| AC-06 | DRD2 | P14416 | D(2) dopamine receptor |
| AC-06 | CA12 | O43570 | Carbonic anhydrase 12 |
| AC-06 | CA1 | P00915 | Carbonic anhydrase 1 |
| AC-06 | CA2 | P00918 | Carbonic anhydrase 2 |
| AC-06 | GARL3 | O60755 | Galanin receptor type 3 |
| AC-06 | CHRNB2 | P17787 | Neuronal acetylcholine receptor subunit beta-2 |
| AC-06 | CHRNA4 | P43681 | Neuronal acetylcholine receptor subunit alpha-4 |
| AC-06 | BBOX1 | O75936 | Gamma-butyrobetaine dioxygenase |
| AC-06 | TBXA2R | P21731 | Thromboxane A2 receptor |
| AC-06 | CHRNA1 | P02708 | Acetylcholine receptor subunit alpha |
| AC-06 | CHAT | P28329 | Choline O-acetyltransferase |
| AC-06 | SLC18A3 | Q16572 | Vesicular acetylcholine transporter |
| AC-07 | SLC28A3 | Q9HAS3 | Solute carrier family 28 member 3 |
| AC-07 | TYR | P14679 | Tyrosinase |
| AC-07 | AMY2A | P04746 | Pancreatic alpha-amylase |
| AC-07 | SLC5A2 | P31639 | Sodium/glucose cotransporter 2 |
| AC-07 | HEXA | P06865 | Beta-hexosaminidase subunit alpha |
| AC-07 | HEXB | P07686 | Beta-hexosaminidase subunit beta |
| AC-07 | B4GALT1 | P15291 | Beta-1,4-galactosyltransferase 1 |
| AC-07 | IL2 | P60568 | Interleukin-2 |
| AC-07 | ALDH1A2 | O94788 | Retinal dehydrogenase 2 |
| AC-07 | ALDH1B1 | P30837 | Aldehyde dehydrogenase X, mitochondrial |
| AC-07 | ERAP1 | Q9NZ08 | Endoplasmic reticulum aminopeptidase 1 |
| AC-07 | SLC5A4 | Q9NY91 | Solute carrier family 5 member 4 |
| AC-07 | SLC5A1 | P13866 | Sodium/glucose cotransporter 1 |
| AC-07 | P4HB | P07237 | Protein disulfide-isomerase |
| AC-07 | HRAS | P01112 | GTPase HRas |
| AC-07 | HSPA5 | P11021 | Endoplasmic reticulum chaperone BiP |
| AC-07 | LGALS9 | O00182 | Galectin-9 |
| AC-07 | CDK1 | P06493 | Cyclin-dependent kinase 1 |
| AC-07 | HSP90AA1 | P07900 | Heat shock protein HSP 90-alpha |
| AC-07 | VEGFA | P15692 | Vascular endothelial growth factor A |
| AC-07 | PSEN2 | P49810 | Presenilin-2 |
| AC-07 | PSENEN | Q9NZ42 | Gamma-secretase subunit PEN-2 |
| AC-07 | NCSTN | Q92542 | Nicastrin |
| AC-07 | APH1A | Q96BI3 | Gamma-secretase subunit APH-1A |
| AC-07 | PSEN1 | P49768 | Presenilin-1 |
| AC-07 | APH1B | Q8WW43 | Gamma-secretase subunit APH-1B |
| AC-07 | FGF1 | P05230 | Acidic fibroblast growth factor |
| AC-07 | HPSE | Q9Y251 | Heparanase |
| AC-07 | FGF2 | P09038 | Basic fibroblast growth factor |
| AC-07 | LGALS4 | P56470 | Galectin-4 |
| AC-07 | LGALS3 | P17931 | Galectin-3 |
| AC-07 | LGALS8 | O00214 | Galectin-8 |
| AC-07 | HTR2B | P41595 | Serotonin 2b (5-HT2b) receptor |
| AC-07 | ADRA2A | P08913 | Alpha-2a adrenergic receptor |
| AC-07 | ADRA2C | P18825 | Adrenergic receptor alpha-2 |
| AC-07 | ADRA2B | P18089 | Alpha-2b adrenergic receptor |
| AC-07 | DRD1 | P21728 | Dopamine D1 receptor |
| AC-07 | DRD2 | P14416 | Dopamine D2 receptor |
| AC-07 | ADRA1D | P25100 | Alpha-1d adrenergic receptor |
| AC-07 | HTR2A | P28223 | Serotonin 2a (5-HT2a) receptor |
| AC-07 | HTR2C | P28335 | Serotonin 2c (5-HT2c) receptor |
| AC-07 | DRD3 | P35462 | Dopamine D3 receptor |
| AC-07 | CYP2D6 | P10635 | Cytochrome P450 2D6 |
| AC-07 | HTR6 | P50406 | Serotonin 6 (5-HT6) receptor |
| AC-07 | ADRA1A | P35348 | Alpha-1a adrenergic receptor (by homology) |
| AC-07 | HTR1B | P28222 | Serotonin 1b (5-HT1b) receptor (by homology) |
| AC-07 | FOLH1 | Q04609 | Glutamate carboxypeptidase II |
| AC-07 | RORC | P51449 | Nuclear receptor ROR-gamma |
| AC-07 | TRPV1 | Q8NER1 | Vanilloid receptor |
| AC-07 | STAT3 | P40763 | Signal transducer and activator of transcription 3 |
| AC-07 | PYGL | P06737 | Liver glycogen phosphorylase |
| AC-07 | PYGM | P11217 | Muscle glycogen phosphorylase |
| AC-07 | PYGB | P11216 | Brain glycogen phosphorylase |
